# Novel glycine amides, semicarbazides and fluoroallylamines as inhibitors of the amine oxidase vascular adhesion protein-1 (VAP-1)

**DOI:** 10.1039/d5md01008j

**Published:** 2026-01-28

**Authors:** Timo Pöstges, Jan Kampschulze, Walburga Hanekamp, Marcel Bermúdez, Matthias Lehr

**Affiliations:** a Institute of Pharmaceutical and Medicinal Chemistry, University of Münster Corrensstrasse 48 48149 Münster Germany lehrm@uni-muenster.de

## Abstract

Vascular adhesion protein-1 (VAP-1), also known as copper-containing amine oxidase 3 (AOC3), is an enzyme implicated in the pathogenesis of various diseases. Increasing evidence highlights VAP-1 as a promising therapeutic target, particularly for the treatment of inflammatory disorders and diabetic complications. We have synthesised a series of compounds in which a heterocycle or a benzene-fused heterocycle is connected *via* a hydrocarbon spacer to a glycine amide, semicarbazide, or fluoroallylamine moiety. These functional groups are believed to act as reactive “warheads”, forming covalent bonds with the topaquinone cofactor at the enzyme's active site. Screening was initially conducted using bovine plasma amine oxidase (AOC4), an enzyme structurally closely related to VAP-1 (AOC3) and also referred to as secretory VAP-1 (sVAP-1). Selected compounds were subsequently evaluated for their ability to inhibit VAP-1 activity in human plasma. The results showed that glycine amide and semicarbazide analogs generally exhibited stronger inhibition of the bovine AOC4 than of the human AOC3. In contrast, fluoroallylamines displayed comparable or even greater inhibitory potency toward the human enzyme. Overall, fluoroallylamines with nanomolar IC_50_ values were identified as the most potent inhibitors of human VAP-1, whereas glycine amides, which act as substrate inhibitors, were the least effective. In assays evaluating inhibition of the related enzyme diamine oxidase (AOC1) as well as monoamine oxidases A and B (MAO A and MAO B), the glycine amides displayed relatively high selectivity for human VAP-1. The semicarbazides, however, also showed strong inhibitory activity against AOC1. Several of the fluorinated allylamines tested were identified as highly potent, well-balanced dual inhibitors of human VAP-1 and MAO B, with (*Z*)-2-({3-[(1*H*-benzotriazol-1-yl)methyl]phenoxy}methyl)-3-fluoroprop-2-en-1-amine (94) being the most effective. Compounds with this dual inhibitory profile are thought to exert particularly beneficial effects in the treatment of inflammatory conditions.

## Abstract 2

Vascular adhesion protein-1 (VAP-1), also known as copper-containing amine oxidase 3 (AOC3), is an enzyme implicated in the pathogenesis of various diseases.

## Graphical abstract

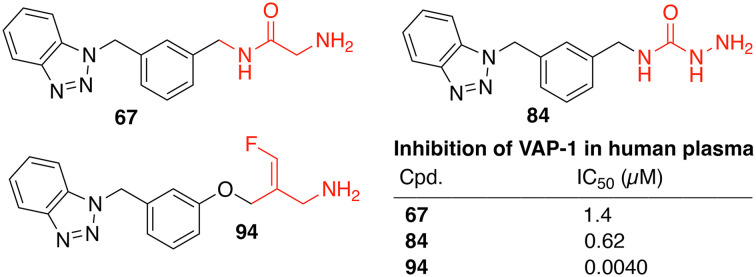

## Introduction

Amine oxidases (AOs) are widespread enzymes that play essential roles in the metabolism of monoamines, diamines, and polyamines.^1^ In humans, two main classes of amine oxidases are found: flavin adenine dinucleotide (FAD)-dependent and copper-dependent enzymes. The group that utilizes FAD as a cofactor includes monoamine oxidases (MAOs), lysine-specific demethylases (LSDs) and polyamine oxidases (PAOs).^2^ Copper-dependent amine oxidases require not only copper but also a tyrosine-derived quinone cofactor. Based on the structure of this cofactor, they are further classified into two subtypes: lysine tyrosylquinone (LTQ)-containing and topaquinone (TPQ)-containing amine oxidases.^3^

Copper-dependent amine oxidases catalyse the oxidative deamination of primary amines, producing the corresponding aldehyde along with hydrogen peroxide and ammonia as byproducts.^3^ FAD-dependent MAOs oxidatively degrade not only primary amines in the same manner, but also secondary and tertiary amines.^4–6^ LSDs and PAOs are involved in the demethylation of mono- and dimethylated lysine residues on histones, and the cleavage of secondary amines, respectively.^7,8^

In humans, four genes encode members of the copper- and topaquinone-dependent amine oxidase family, also known as amine oxidases copper-containing (AOC).^9^ Of these, three – AOC1, AOC2, and AOC3 – codify functional enzymes, whereas AOC4 is a pseudogene that lacks protein-coding capacity. In contrast, certain other mammals, such as cattle and pigs, possess a functional AOC4 gene that encodes an active amine oxidase enzyme. AOC1, also referred to as diamine oxidase (DAO) or histaminase, works in concert with histamine *N*-methyltransferase to metabolise the biogenic diamine histamine.^10^ AOC2 enzyme activity has been detected in the retina, leading to its alternative name, retina-specific amine oxidase.^11^

The best-characterised topaquinone-containing amine oxidase is AOC3, commonly known by several names, including benzylamine oxidase, clorgiline-resistant amine oxidase, plasma amine oxidase, primary amine oxidase, semicarbazide-sensitive amine oxidase (SSAO), serum monoamine oxidase, and vascular adhesion protein-1 (VAP-1).^12^ In the following text, the enzyme will be referred to as VAP-1. VAP-1 exists both as a membrane-bound form and as a soluble, circulating form in plasma. The latter is believed to arise through proteolytic cleavage of the membrane-associated protein, primarily from vascular endothelial cells.^13^ For a long time, the physiological and pathophysiological roles of VAP-1 remained poorly understood.^14^ Moreover, its endogenous substrates *in vivo* have not yet been clearly identified.

Elevated plasma concentrations of VAP-1 have been observed in various diseases, including liver fibrosis and both type 1 and type 2 diabetes.^15–20^ In type 2 diabetes, it is hypothesised that increased VAP-1 levels serve a compensatory role in regulating blood glucose levels. The hydrogen peroxide generated by VAP-1 activity may exert insulin-mimetic effects by promoting the translocation of glucose transporters GLUT1 and GLUT4 to the plasma membrane. However, this same hydrogen peroxide, along with the concomitant produced aldehydes, contributes to vascular damage, leading to diabetic complications such as nephropathy and retinopathy.^16,19^ Beyond diabetes, VAP-1 plays a significant role in cerebrovascular and cardiovascular diseases,^21,22^ as well as in inflammatory processes.^23–27^ It facilitates the transmigration of leukocytes from the bloodstream into inflamed tissues, where these immune cells intensify the local inflammatory response. Consequently, VAP-1 inhibitors are being explored as promising therapeutic agents for inflammatory and vascular-related disorders.

**Fig. 1:**
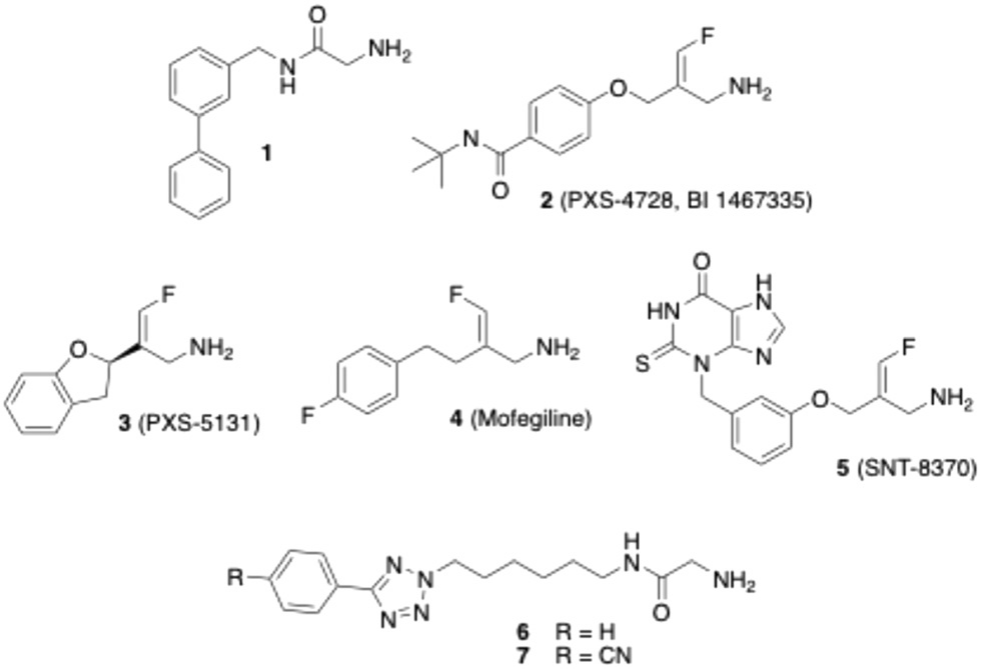
Structures of known VAP-1 inhibitors.

Several inhibitors of VAP-1 have already been described in the literature,^28–31^ such as the glycine amide 1^32^ (Fig. 1), the carbamimidoyl carbamate ASP8232^33^ and fluoroallylamines like PXS-4728 (2).^34^ Although some of these substances showed promising effects in animal trials, clinical studies in humans have so far been rather disappointing.^30,31^ For example, Astellas' compound ASP8232 was evaluated in phase II studies for its efficacy in treating diabetic macular edema and diabetic nephropathy. Despite effectively inhibiting VAP-1 activity in plasma following oral application, ASP8232 failed to show any therapeutic benefit in patients with macular edema.^35^ In contrast, ASP8232 was significantly effective in inhibiting albuminuria in diabetic patients, indicating that diabetic kidney damage may be delayed.^36^ PXS-4728, developed by Pharmaxis, has been tested by Boehringer Ingelheim under the designation BI 1467335 in phase II clinical trials for the treatment of diabetic retinopathy and non-alcoholic steatohepatitis (NASH).^37,38^ However, these were discontinued due to a high risk of dose-dependent drug interactions.^30^ A trial with the monoclonal antibody timolumab (BTT1023) for therapy of primary sclerosing cholangitis, which is characterised by biliary and liver sclerosis, was stopped due to lack of efficacy.^39^

Actually, there is growing interest in compounds that, beyond inhibiting VAP-1, also modulate a second molecular target, as such agents may offer enhanced therapeutic efficacy. For example, the dual VAP-1/MAO B inhibitor PXS-5131 (3) – derived from the MAO B inhibitor mofegiline (4), which was clinically investigated for the treatment of Parkinson's disease – has demonstrated anti-inflammatory activity in models of both acute inflammation and neuroinflammation.^40,41^ Moreover, the dual inhibition of VAP-1 and myeloperoxidase (MPO) is being investigated as a promising strategy for anti-inflammatory therapy. A representative compound exhibiting this dual activity is SNT-8370 (5).^42^ Taken together, these findings underscore that VAP-1 remains an attractive target for the development of novel therapeutic agents.^31^

As mentioned above, several VAP-1 inhibitors with a reactive glycine amide head group have been described in the literature, including compound 1 (Fig. 1).^32^ Building on these findings, we recently synthesised a series of related compounds incorporating an aryltetrazolylalkyl substituent on the amide nitrogen of the glycine amide moiety, such as compounds 6 and 7.^43,44^ Initial inhibition assays were performed using bovine AOC4, an amine oxidase secreted into the plasma that is structurally very similar to VAP-1 and also referred to as secretory VAP-1 (sVAP-1).^45,46^ Further investigations revealed that compounds 1, 6, and 7 undergo oxidative deamination by bovine sVAP-1 resulting in the formation of a glyoxamide derivative. These findings indicate that glycine amide-based compounds do not act as covalent inhibitors, as initially proposed for compound 1, but rather function as substrate inhibitors – competing with the enzyme's natural substrate for binding and undergoing enzymatic oxidation.

In this study, we report the results of further structural modifications of glycine amide-based inhibitors 6 and 7. Initially, the phenyltetrazole moiety was replaced with benzannelated azoles, including indole, benzimidazole, and benzotriazole. Subsequently, a series of benzotriazole derivatives was synthesised in which the glycine amide moiety was replaced by a semicarbazide group. Additionally, various fluoroallylamine derivatives bearing terminal phenyltetrazole or benzotriazole residues were prepared. The compounds were evaluated for their ability to inhibit isolated bovine sVAP-1, their stability toward degradation by this VAP-1-related enzyme, and their potential mechanism of inhibition. Selected compounds were also tested for their ability to inhibit VAP-1 activity in human plasma and their inhibitory activity against other amine oxidases.

## Results and discussion

### Chemistry

**Scheme 1:**
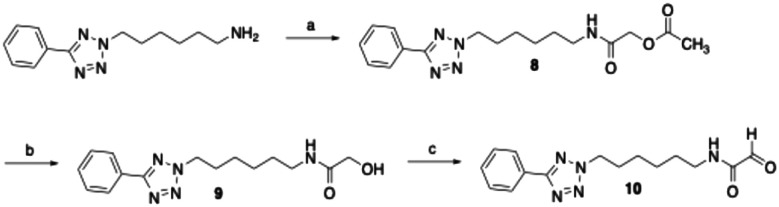
Reagents and conditions: (a) acetoxyacetic acid, EDC, 1-hydroxybenzotriazole, DMF, room temperature, 15 h, 88%; (b) K_2_CO_3_, methanol/water (1 : 1), 50 °C, 2 h, 75%; (c) Dess–Martin periodinane, dichloromethane, 0–20 °C, 2 h, 49%.

To verify that glycine amide 6 can be oxidised by bovine sVAP-1 to the aldehyde 10, the latter was independently synthesised as a reference compound. For this purpose, 6-(5-phenyl-2*H*-tetrazol-2-yl)hexan-1-amine^47^ was first acylated with acetoxyacetic acid following activation with EDC and hydroxybenzotriazole (Scheme 1). The acetoxy group of the resulting compound 8 was saponified with potassium carbonate in aqueous methanol, yielding 9. Finally, the terminal hydroxy group of 9 was oxidised using Dess–Martin periodinane to afford the target glyoxamide 10.

**Scheme 2:**
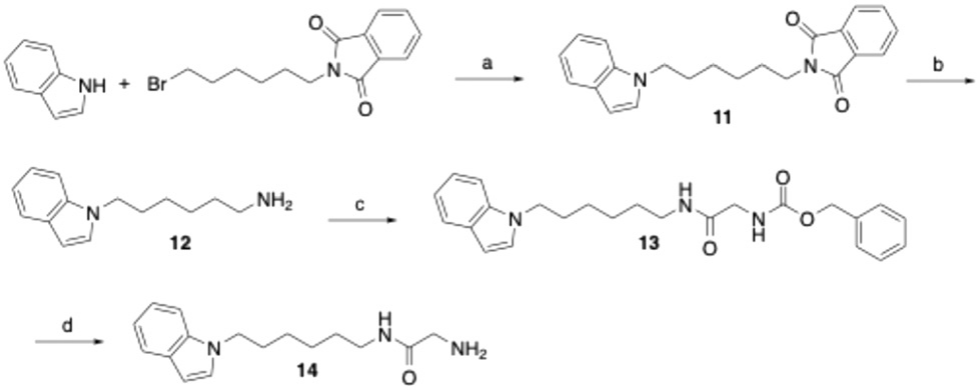
Reagents and conditions: (a) NaH, DMF, room temperature, 16 h followed by 60 °C, 3 h, 29%; (b) hydrazine monohydrate, ethanol, reflux, 5 h, quantitative; (c) *N*-(benzyloxycarbonyl)glycine, EDC, 1-hydroxybenzotriazole, DMF, room temperature, 20 h, 76%; (d) H_2_, Pd/C, THF, methanol, room temperature, 4 h, 94%.

The derivative of compound 6, in which the phenyltetrazole moiety is replaced by an indole residue, was prepared as outlined in Scheme 2. Thus, indole was alkylated at the nitrogen position in DMF using *N*-(6-bromohexyl)phthalimide following deprotonation with sodium hydride. The phthalimide protecting group in the resulting compound 11 was then removed by hydrazinolysis to yield amine 12. Subsequent coupling with glycine, protected on the nitrogen with a benzyloxycarbonyl (Cbz) group and activated by EDC/1-hydroxybenzotriazole, afforded the glycine amide derivative 13. Final hydrogenolytic removal of the Cbz group led to the desired target glycine amide 14.

**Scheme 3:**
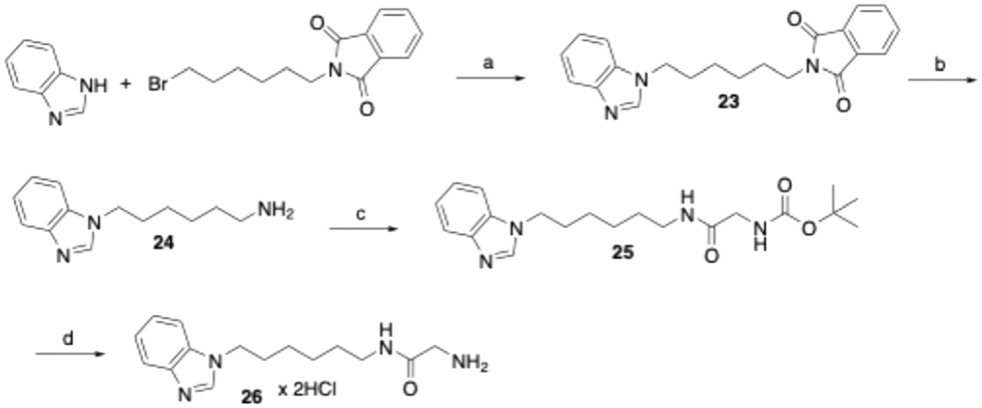
Reagents and conditions: (a) NaH, DMF, room temperature, 60 °C, 4 h, 75%; (b) hydrazine monohydrate, ethanol, reflux, 4.5 h, 67%; (c) *N*-(*tert*-butoxycarbonyl)glycine, EDC, 1-hydroxybenzotriazole, DMF, room temperature, 18 h, 80%; (d) 4 M HCl in cyclopentyl methyl ether, ethyl acetate, room temperature, 3 h, 58%.

The synthesis of the corresponding indazole, benzimidazole, and benzotriazole derivatives did not proceed *via* a benzyloxycarbonyl-protected glycine amide intermediate, but through a BOC-protected glycine amide. Using the synthesis of the benzimidazole derivative 26 as an example, the benzannelated heterocycle benzimidazole was first alkylated with *N*-(6-bromohexyl)phthalimide using NaH for deprotonation of the nitrogen (Scheme 3). Subsequent hydrazinolysis of the resulting compound 23 afforded the hexanamine derivative 24, which was then coupled with *N*-(*tert*-butoxycarbonyl)glycine to yield the BOC-protected glycine amide 25. Deprotection using hydrochloric acid in cyclopentyl methyl ether afforded the target compound 26 as the dihydrochloride salt. The formation of the dihydrochloride salt was confirmed by CHN elemental analysis and ^1^H NMR spectroscopy in DMSO-d_6_. In the NMR spectra, a consistent downfield shift of the solvent water signal was observed for glycine amides isolated as dihydrochlorides. Additionally, the methylene protons vicinal to the amine group displayed quartet splitting, further supporting the structure.

In the analogous reactions of indazole and benzotriazole with *N*-(6-bromohexyl)phthalimide also isomers alkylated in position 2 of the heterocycle were formed. These N2-alkylated byproducts were isolated and separately converted into glycine amide target compounds. ^1^H NMR and CHN analyses indicated that the 2*H*-benzotriazole derivative 34 was isolated as a monohydrochloride salt, whereas the 1*H*-benzotriazole 31 and both indazole derivatives (19 and 22) were obtained as dihydrochloride salts. Notably, in the synthesis of the benzotriazole derivatives 31 and 34, in the first step potassium carbonate was used instead of NaH to deprotonate the heterocycle, and the reaction was carried out in acetonitrile.

Derivatives of compound 31 bearing a nitrile substituent at position 5 or 6 on the benzotriazole ring (39 and 42), or having alkyl chains of varying lengths (46, 50, 54 and 58), were prepared accordingly. In the initial reaction step, only the 1*H* isomers were isolated and subsequently subjected to further transformations. CHN analysis revealed that the nitrile-containing derivatives were obtained as monohydrochloride salts, whereas compounds lacking a nitrile group in the heterocycle were isolated as dihydrochloride salts.

**Scheme 4:**
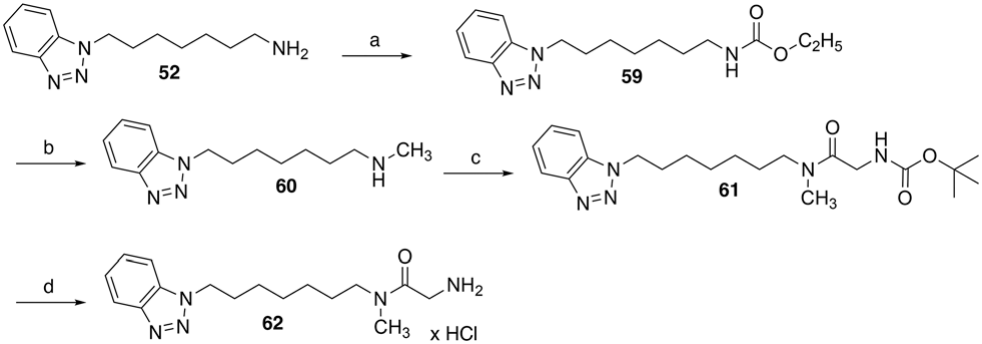
Reagents and conditions: (a) ethyl chloroformate, triethylamine, dichloromethane, 0 °C, 30 min followed by room temperature, 2.5 h, 76%; (b) LiAlH_4_, THF, 0 °C followed by reflux, 3 h, 81%; (c) *N*-(*tert*-butoxycarbonyl)glycine, EDC, 1-hydroxybenzotriazole, DMF, room temperature, 22 h, 75%; (d) 4 M HCl in cyclopentyl methyl ether, ethyl acetate, room temperature, 4 h, 48%.

The synthesis of a derivative of 54, which is additionally methylated at the glycine amide group, was carried out starting from benzotriazole-substituted heptan-1-amine 52 (see SI) (Scheme 4). This was reacted with ethyl chloroformate and triethylamine in dichloromethane to yield ethyl carbamate 59. Subsequent reduction with lithium aluminium hydride in dry THF produced the secondary amine 60. EDC/1-hydroxybenzotriazole-mediated coupling of 60 with Boc-glycine amide and subsequent cleavage of the Boc protecting group using 4 M HCl in cyclopentyl methyl ether led to the test compound 62, which was obtained as a monohydrochloride salt.

**Scheme 5:**
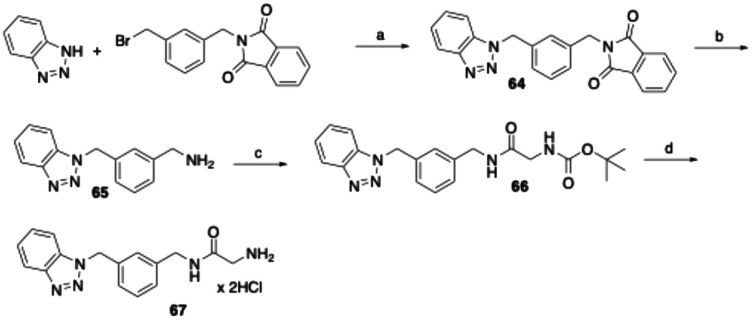
Reagents and conditions: (a) K_2_CO_3_, acetonitrile, reflux, 16 h, 65%; (b) hydrazine monohydrate, ethanol, reflux, 4 h, 92%; (c) *N*-(*tert*-butoxycarbonyl)glycine, EDC, 1-hydroxybenzotriazole, DMF, room temperature, 24 h, 84%; (d) 4 M HCl in cyclopentyl methyl ether, ethyl acetate, room temperature, 3 h, 61%.

To synthesize the derivative of 54, in which the carbon atoms 2–6 of the heptyl spacer between the amide and benzotriazole are replaced by a *meta*-substituted phenyl ring, benzotriazole was alkylated at the N1 nitrogen with 2-[3-(bromomethyl)benzyl]isoindoline-1,3-dione^48^ in a nucleophilic substitution reaction (Scheme 5). After hydrazinolytic cleavage of the phthalimide protecting group, the released primary amine moiety was acylated with *N*-(*tert*-butoxycarbonyl)glycine as described above. Finally, the Boc protecting group was cleaved with HCl in cyclopentyl methyl ether to yield the target compound 67.

**Scheme 6:**
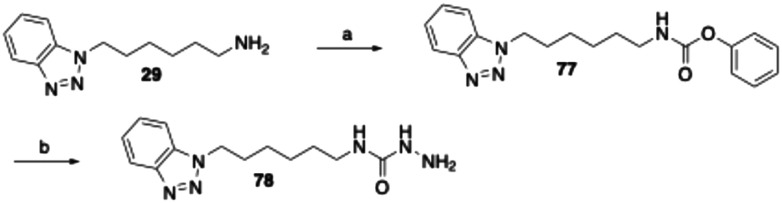
Reagents and conditions: (a) diphenyl carbonate, THF/water, 40 °C, 2 h, 87%; (b) hydrazine monohydrate, 1,2-dimethoxyethane, 80 °C, 6 h, 51%.

The synthesis of the phenyltetrazolyl and benzotriazolyl derivatives bearing a semicarbazide warhead was carried out as exemplified by the preparation of compound 78 (Scheme 6). In this case, the semicarbazide moiety is substituted with a benzotriazol-1-ylhexyl residue. The corresponding amine precursor (29), was first converted into phenyl carbamate 77 using diphenyl carbonate. Subsequent treatment of 77 with hydrazine hydrate in 1,2-dimethoxyethane afforded the target semicarbazide 78.

**Scheme 7:**
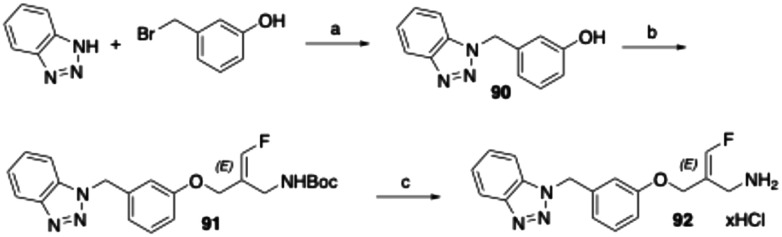
Reagents and conditions: (a) K_2_CO_3_, DMF, room temperature, overnight, 65%; (b) *tert*-butyl (*E*)-[2-(bromomethyl)-3-fluoroallyl]carbamate, Cs_2_CO_3_, acetonitrile, room temperature, overnight, 42%; (c) 4 M HCl in cyclopentyl methyl ether, ethyl acetate, room temperature, 2 h followed by 50 °C, 2 h, 10%.

The compounds with fluoroallylamine warheads were synthesised as shown for the corresponding *E*-configured benzotriazole-substituted derivative 92 (Scheme 7). Benzotriazole was first alkylated at the N1 position with 3-(bromomethyl)phenol^49^ in the presence of potassium carbonate, affording intermediate 90. This was subsequently reacted with *tert*-butyl (*E*)-[2-(bromomethyl)-3-fluoroallyl]carbamate^50,51^ in acetonitrile using cesium carbonate for deprotonation of the phenol group to yield the Boc-protected fluoroallylamine 91. Final Boc deprotection with HCl in cyclopentyl methyl ether afforded the desired *E*-configured fluoroallylamine 92 as its hydrochloride salt.

### Biological evaluation

For the initial screening of VAP-1 inhibitors, we used amine oxidase isolated from bovine plasma.^47,52^ This enzyme is composed primarily of AOC4, which is predominantly expressed in the bovine liver and secreted into the bloodstream, where it accounts for most of the plasma amine oxidase activity.^45,46^ Due to its high sequence homology with VAP-1 (AOC3), AOC4 is also referred to as secretory VAP-1 (sVAP-1).^9,45,46^ Given this similarity and the commercial availability of bovine sVAP-1 in large quantities at a reasonable cost, we considered it a suitable enzyme source for the initial development of VAP-1 inhibitors. The tests were performed twice: once without pre-incubation and once with a 15 minute pre-incubation of the enzyme with the inhibitor. This approach was chosen to provide insight into the possible mechanism of action of the compounds. A decrease in inhibitory effect after 15 min of pre-incubation suggests that the substance is degraded by the enzyme and therefore acts as a substrate. If the inhibition remains unchanged, this indicates a competitive inhibitor. An increase in inhibitory activity after pre-incubation points to a covalent inhibitor, as such compounds typically require time to form a covalent bond with the enzyme. In addition to evaluating their inhibitory effect on bovine sVAP-1 activity, the new compounds were also tested for their stability against enzymatic metabolism by this enzyme. Furthermore, selected derivatives were evaluated for their ability to inhibit VAP-1 activity in human plasma and for their selectivity against other amine oxidases, namely diamine oxidase (DAO) and monoamine oxidases A and B (MAO A and MAO B).^52^

**Scheme 8:**
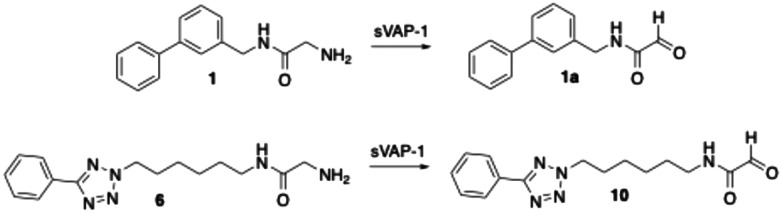
Deamination of the glycine amides 1 and 6 by bovine sVAP-1 (AOC4) to glyoxamides.

Previous studies had demonstrated that the phenyltetrazolylalkyl-substituted glycine amides investigated are substrates of bovine sVAP-1.^44^ Their inhibitory effect on the enzyme arises from the fact that they are converted more slowly by the enzyme than the substrate used, making them substrate inhibitors. The deamination of these glycine amides by the enzyme was confirmed by LC/MS analysis through detection of the expected glyoxamide. For the reference inhibitor 1, the corresponding glyoxamide 1a (Scheme 8) was also synthesised and spiked into a reaction sample. The LC/MS chromatogram obtained showed only one peak having the same retention time and mass spectrum as the product formed during the reaction of 1 with bovine sVAP-1, which confirmed the assumed structure of 1a.

In this study, first the putative glyoxamide product 10 (Scheme 8), generated from glycine amide 6 by bovine sVAP-1, was synthesised and compared directly with the enzymatic reaction product. LC/MS again revealed co-elution and identical mass spectra, verifying that bovine sVAP-1 indeed produces glyoxamide 10 from glycine amide 6.

Interestingly, inhibition assays with glyoxamide 10 gave unexpected results. Whereas the glyoxamide 1a derived from compound 1 showed no effect at 10 μM, glyoxamide 10 inhibited bovine sVAP-1 by 27% without pre-incubation and by 47% after 15 min pre-incubation of the enzyme with the inhibitor. The enhanced inhibition following pre-incubation suggests that the formyl group of glyoxamide 10 reacts covalently with the enzyme. However, its inhibitory potency remained lower than that of the parent glycine amide 6 (about 60% inhibition under both conditions at 10 μM^43^), which shows that the phenyltetrazolylalkyl-substituted glycine amides themselves, and not their degradation products, are primarily responsible for the measured inhibitory effect.

In structure–activity relationship (SAR) studies described in the present work, the phenyltetrazole moiety of compound 6 was replaced with benzene-fused azoles, including indole, indazole, benzimidazole, and benzotriazole. In the initial assays, the test compounds were evaluated without pre-incubation, meaning the enzyme was added directly to a solution containing both inhibitor and substrate. Under these conditions, the benzotriazole derivative 31 was the most potent, exhibiting an IC_50_ of 1.1 μM, comparable to that of the nitrile-substituted tetrazole 7 (Table 1). However, upon 15 minutes of preincubation, the IC_50_ values of compound 31 and the slightly less potent benzimidazole 26 increased from 1.1 and 2.0 μM to 3.0 and 4.6 μM, respectively. This loss of activity suggests that both compounds – like the phenyl-tetrazolyl-substituted glycine amides investigated – act as substrates of bovine sVAP-1 and undergo enzymatic degradation to less active or inactive aldehyde products.

**Table 1:** Inhibition of isolated bovine sVAP-1 by several glycine amide derivatives evaluated without and with a 15 min pre-incubation of enzyme and inhibitor

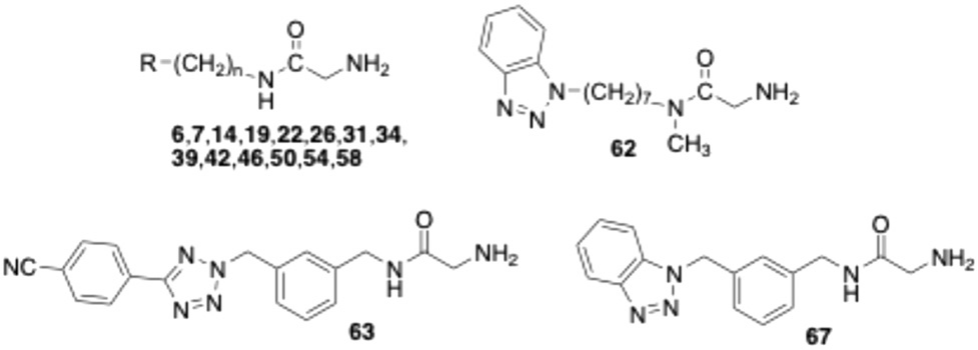				
Cpd.	R	*n*	Inhibition of bovine sVAP-1	
			IC_50_^a^ (μM)	
			Without pre-incubation	With pre-incubation
6	5-Phenyl-2*H*-tetrazol-2-yl	6	6.0	6.3
7	5-(4-CN-phenyl)-2*H*-tetrazol-2-yl	6	1.5	2.3
14	Indol-1-yl	6	3.8	n.d.
19	Indazol-1-yl	6	2.1	n.d.
22	Indazol-2-yl	6	>10^b^	n.d.
26	Benzimidazol-1-yl	6	2.0	4.6
31	Benzotriazol-1-yl	6	1.1	3.0
34	Benzotriazol-2-yl	6	8.7	n.d.
39	5-CN-benzotriazol-1-yl	6	1.8	4.0
42	6-CN-benzotriazol-1-yl	6	>10^c^	n.d.
46	Benzotriazol-1-yl	4	n.a.	n.d.
50	Benzotriazol-1-yl	5	>10^d^	n.d.
54	Benzotriazol-1-yl	7	0.45	1.7
58	Benzotriazol-1-yl	8	0.53	1.4
62			3.5	6.2
63			0.24	0.13
67			0.35	0.36
1			0.72 ± 0.14	0.37 ± 0.14
2 (PXS-4728)			1.0 ± 0.3	0.027 ± 0.003

a IC_50_-values of the target compounds are the means of two independently performed determinations, errors are within ±20%; IC_50_-values of references 1 and 2: mean ± standard deviation, *n* = 5 (1) or 4 (2); n.a.: not active at 10 μM; n.d.: inhibition not determined.

b 44% inhibition at 10 μM.

c 30% inhibition at 10 μM.

d 41% inhibition at 10 μM.

Introduction of a nitrile group at the *para*-position of the phenyl ring in phenyltetrazole 6 (yielding compound 7) enhanced inhibitory potency by 3–4 fold (Table 1).^44^ This prompted evaluation of analogous nitrile substitutions on the phenyl moiety of the benzotriazole heterocycle in glycine amide 31. However, inhibition data for 39 and 42 demonstrated that nitrile groups at the 5- or 6-position of the benzotriazole ring did not improve activity. The benzotriazole-5-nitrile derivative 39 displayed activity similar to the parent compound 31, whereas substitution at the 6-position (42) resulted in a pronounced loss of activity.

For the most effective compound initially identified, benzotriazole 31, the length of the alkyl spacer between the glycine amide nitrogen and the heterocycle was systematically varied. Shortening the chain from six to five or four carbon atoms caused a marked loss of activity (Table 1). The derivative with a pentyl chain (50) retained only a slight effect at 10 μM (without pre-incubation), while the compound with a butyl spacer (46) was completely inactive at this concentration. In contrast, extending the hexyl chain by one or two additional carbons (54, 58) approximately doubled the inhibitory activity in both assay formats. Nonetheless, activity in the pre-incubation experiments was consistently lower than in the corresponding assays without pre-incubation, similar to the behavior measured for the analogous glycine amides bearing phenyltetrazole substituents.^44^ This observation further supports the notion that the compounds are degraded by bovine sVAP-1. This was further confirmed by stability measurements. After incubation of compound 54 with the bovine enzyme, only 31 ± 11% (mean ± SD, *n* = 4) of the parent compound remained after 60 min at a concentration of 10 μM, while a peak corresponding to the mass/charge ratio of the expected glyoxamide simultaneously appeared in the LC/MS chromatogram. For phenyltetrazoles 6 and 7, the remaining amounts at the same concentration were 27 ± 15% and 60 ± 4% (mean ± SD, *n* = 3), respectively.

Next, the amide nitrogen of benzotriazole 54 was methylated, yielding compound 62, which displayed markedly reduced activity compared with the unmethylated glycine amide. A similar loss of activity upon amide methylation has also been reported for other glycine amide derivatives in the literature.^32,44^

In our earlier studies on glycine amides bearing terminal phenyltetrazole groups,^44^ we synthesised compound 63 (Table 1), in which the flexible hexyl spacer was replaced by a more rigid *meta*-dimethylphenyl scaffold. This structural modification resulted in a pronounced enhancement of biological activity accompanied by increased resistance to enzymatic degradation. Applying the same structural modification to compound 31 likewise enhanced inhibitory potency significantly, as demonstrated by the submicromolar IC_50_ values of the corresponding benzotriazole derivative 67 (Table 1), while the stability toward the enzyme was still high (82 ± 3% after 60 min incubation at 10 μM, mean ± SD, *n* = 4). However, these effects were somewhat less marked than those observed for the analogous phenyltetrazole-containing glycine amide 63. Thus, the IC_50_ values of 63 (with and without pre-incubation) were approximately 2–3 times lower than those of 67, and after 60 min of incubation at a concentration of 10 μM with the enzyme, even 90 ± 4% of the parent compound 63 was detectable. The pronounced stability of the compounds containing the *meta*-substituted dimethylphenyl scaffold against degradation by bovine sVAP-1 also explains why their IC_50_ values do not change significantly after pre-incubation of the substances with the enzyme. It should be noted, however, that 63 and 67 are ultimately also converted by the enzyme. At a concentration of 1 μM, the residual amount of parent substance after 60 min was less than 5%.

Although semicarbazide has long been recognised as an inhibitor of VAP-1 – hence the alternative name semicarbazide-sensitive amine oxidase – surprisingly, only few semicarbazide derivatives have been developed as VAP-1 inhibitors.^31,53^ One of these is compound 68 (Table 2), which we synthesised.^43^ In the assay without pre-incubation of bovine sVAP-1 and inhibitor, this substance showed only weak inhibitory activity (IC_50_ > 10 μM). However, when the enzyme was treated with the inhibitor for 15 min prior to substrate addition, the potency increased markedly, yielding an IC_50_ of 0.18 μM. This inhibitory behaviour indicates that, unlike glycine amides, the semicarbazide acts as a covalent inhibitor of the enzyme. Because the covalent bond with the topaquinone cofactor takes time to form, inhibition is stronger when the inhibitor has time to bind before the substrate is added.

**Table 2:** Inhibition of isolated bovine sVAP-1 by several semicarbazide derivatives evaluated without and with a 15 min pre-incubation of enzyme and inhibitor

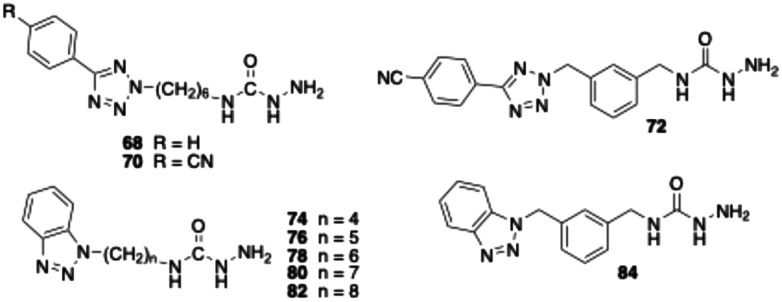		
Cpd.	Inhibition of bovine sVAP-1	
	IC_50_^a^ (μM)	
	Without pre-incubation	With pre-incubation
68	>10^b^	0.18
70	4.2	0.067
72	2.1	0.022
74	>10^c^	1.0
76	>10^d^	0.21
78	0.85	0.032
80	0.75	0.026
82	1.1	0.035
84	6.9	0.066
1	0.72 ± 0.14	0.37 ± 0.14
2 (PXS-4728)	1.0 ± 0.3	0.027 ± 0.003

a IC_50_-values of the target compounds are the means of two independently performed determinations, errors are within ±20%; IC_50_-values of references 1 and 2: mean ± standard deviation, *n* = 5 (1) or 4 (2).

b 46% inhibition at 10 μM.

c 22% inhibition at 10 μM.

d 41% inhibition at 10 μM.

As part of this study, we first examined how the inhibitory activity of semicarbazide 68 is affected by introducing a nitrile group at the *para*-position of the terminal phenyl ring. In a second step, we replaced the hexyl spacer with a *meta*-dimethylphenyl moiety. The test results for target compounds 70 and 72 demonstrate that both modifications substantially enhanced inhibitory potency (Table 2). The IC_50_ values, measured with pre-incubation, decreased by factors of 3 and 8, respectively. Moreover, the data indicate that compound 72 exhibits inhibitory activity comparable to that of the reference inhibitor PXS-4728 (2).

Replacing the 4-cyanophenyltetrazolyl residue of compound 70 with a benzotriazol-1-yl substituent yielded compound 78, which displayed significantly improved inhibitory potency – approximately fourfold without pre-incubation and twofold with pre-incubation compared to the parent compound. Shortening the alkyl spacer of 78 from six to five or four carbon atoms resulted in a pronounced loss of activity, as reflected by the IC_50_ values of compounds 74 and 76. In contrast, elongation of the alkyl chain from six (78) to seven or eight carbon atoms (80 and 82) did not significantly affect enzyme inhibition. Nevertheless, all three analogues exhibited inhibitory potencies comparable to that of the reference PXS-4728 (2). Surprisingly, replacement of the hexyl chain with a *meta*-dimethylphenyl substituent (84) caused a marked reduction in potency (by factors of seven and two, respectively), whereas the same modification had increased inhibition in the phenyl tetrazole series. Finally, stability studies revealed that, unlike glycine amides, the semicarbazide derivatives tested are not degraded by bovine sVAP-1.

Next, phenyltetrazole and benzotriazole derivatives were prepared which, like the *E*-configured PXS-4728, carry a reactive fluoroallylamine warhead.^54,55^ The synthetic routes afforded isomers that predominantly or entirely exhibited the *E* or *Z* configuration. The generally accepted mechanism of action of this type of compounds is that the amino group of the fluoroallylamine initially forms a Schiff base with the carbonyl group of the cofactor topaquinone. This produces a highly reactive intermediate which, by eliminating the fluoride, covalently alkylates a nucleophilic amino acid residue or the topaquinone cofactor in the active centre, thereby irreversibly inhibiting the enzyme.^30,40,42,55,56^

Testing against bovine sVAP-1 revealed that, unlike PXS-4728 and the semicarbazides, these fluoroallylamines (87, 89, 92 and 94) showed only small differences in IC_50_ values in the assays with and without pre-incubation (factors of 2–3 compared to 20–40 for PXS-4728 and the semicarbazides) (Table 3). This indicates that the fluoroallylamines react with the cofactor of the enzyme more rapidly than PXS-4728 or the semicarbazides leading to a fast onset of maximum inhibition. After 15 min of pre-incubation, however, the maximal inhibitory potency of all these compounds was comparable: the most active semicarbazides, PXS-4728, and the newly synthesised fluoroallylamines all exhibited IC_50_ values of about 0.030 μM. Stability assays further demonstrated that the fluoroallylamines were largely resistant to degradation by bovine sVAP-1.

**Table 3:** Inhibition of isolated bovine sVAP-1 by several fluoroallylamine derivatives evaluated without and with a 15 min pre-incubation of enzyme and inhibitor

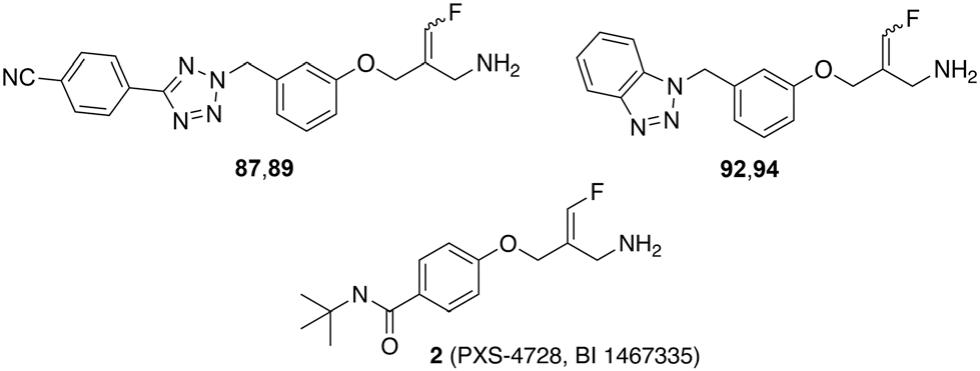		
Cpd.	Inhibition of bovine sVAP-1	
	IC_50_^a^ (μM)	
	Without pre-incubation	With pre-incubation
87 (*E* : *Z* 95 : 5)	0.12	0.035
89 (*E* : *Z* 0 : 100)	0.11	0.040
92 (*E* : *Z* 80 : 20)	0.092	0.030
94 (*E* : *Z* 8 : 92)	0.076	0.033
2 (PXS-4728) (*E*)	1.0 ± 0.3	0.027 ± 0.003

a IC_50_-values of the target compounds are the means of two independently performed determinations, errors are within ±20%; IC_50_-values of reference 2: mean ± standard deviation, *n* = 4.

Selected compounds were evaluated for their ability to inhibit the soluble form of VAP-1 present in human plasma. Following a 15 minute pre-incubation of the inhibitor with the enzyme, it was observed that the glycine amides and semicarbazides tested exhibited markedly lower inhibitory activity against the human enzyme compared to bovine sVAP-1 (Table 4). Specifically, their IC_50_ values were approximately 7 to 23 times higher for the human enzyme. Notable exceptions were the two glycine amides with a *meta*-dimethylphenyl spacer (compounds 63 and 67). For these compounds, the difference in inhibitory potency between the human and bovine enzymes was only 2- and 4-fold, respectively. In contrast to the glycine amides and semicarbazides, the fluoroallylamines exhibited a distinct profile. The two tetrazole derivatives 87 and 89 demonstrated similar IC_50_ values in both inhibition assays. However, the benzotriazoles 92 and 94, along with PXS-4728 (2), were even markedly more potent against the human plasma enzyme than against the bovine counterpart. Among the newly synthesised compounds, the *Z*-configured fluoroallylamine 94 emerged as the most potent inhibitor of human VAP-1, with an IC_50_ value of 4 nM. This represents approximately three times the potency of mofegiline and one-third the potency of PXS-4728.

**Table 4:** Inhibition of VAP-1 activity in human plasma and sVAP-1 isolated from bovine plasma by selected compounds evaluated with a 15 min pre-incubation of enzyme and inhibitor

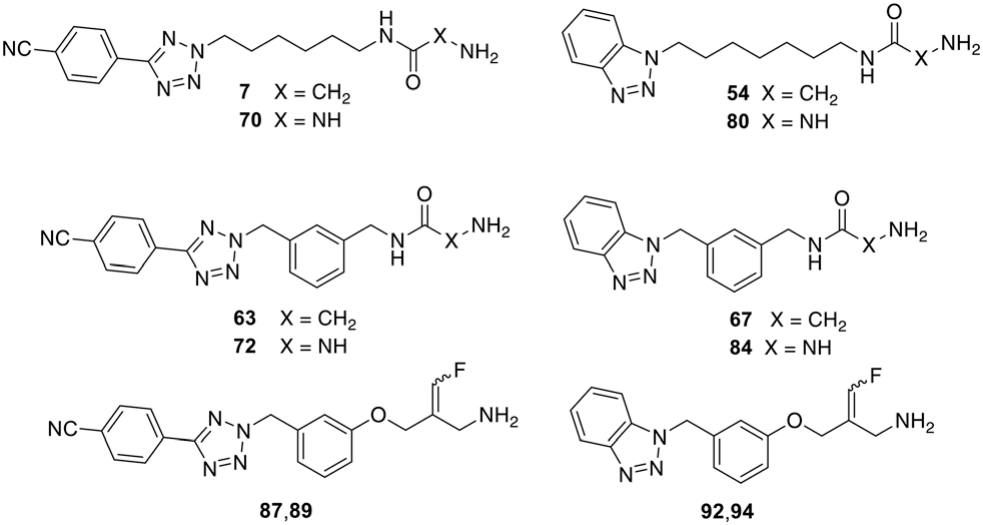		
Cpd.	Inhibition IC_50_^a^ (μM)	
	VAP-1 in human plasma	sVAP-1 from bovine plasma
Glycine amides		
7	39	2.3
54	39	1.7
63	0.32	0.13
67	1.4	0.36
Semicarbazides		
70	1.3	0.067
80	0.40	0.024
72	0.17	0.022
84	0.62	0.066
Fluoroallylamines		
87 (*E* : *Z* 95 : 5)	0.050	0.045
89 (*E* : *Z* 0 : 100)	0.021	0.040
92 (*E* : *Z* 80 : 20)	0.0074	0.030
94 (*E* : *Z* 8 : 92)	0.0040	0.033
References		
1	2.6	0.37
2 (PXS-4728)	0.0016	0.027
4 (Mofegiline)	0.012	n.d.

a IC_50_-values of the compounds are the means of two independently performed determinations, errors are within ±20%. For the standard deviations of the means see SI.

**Fig. 2:**
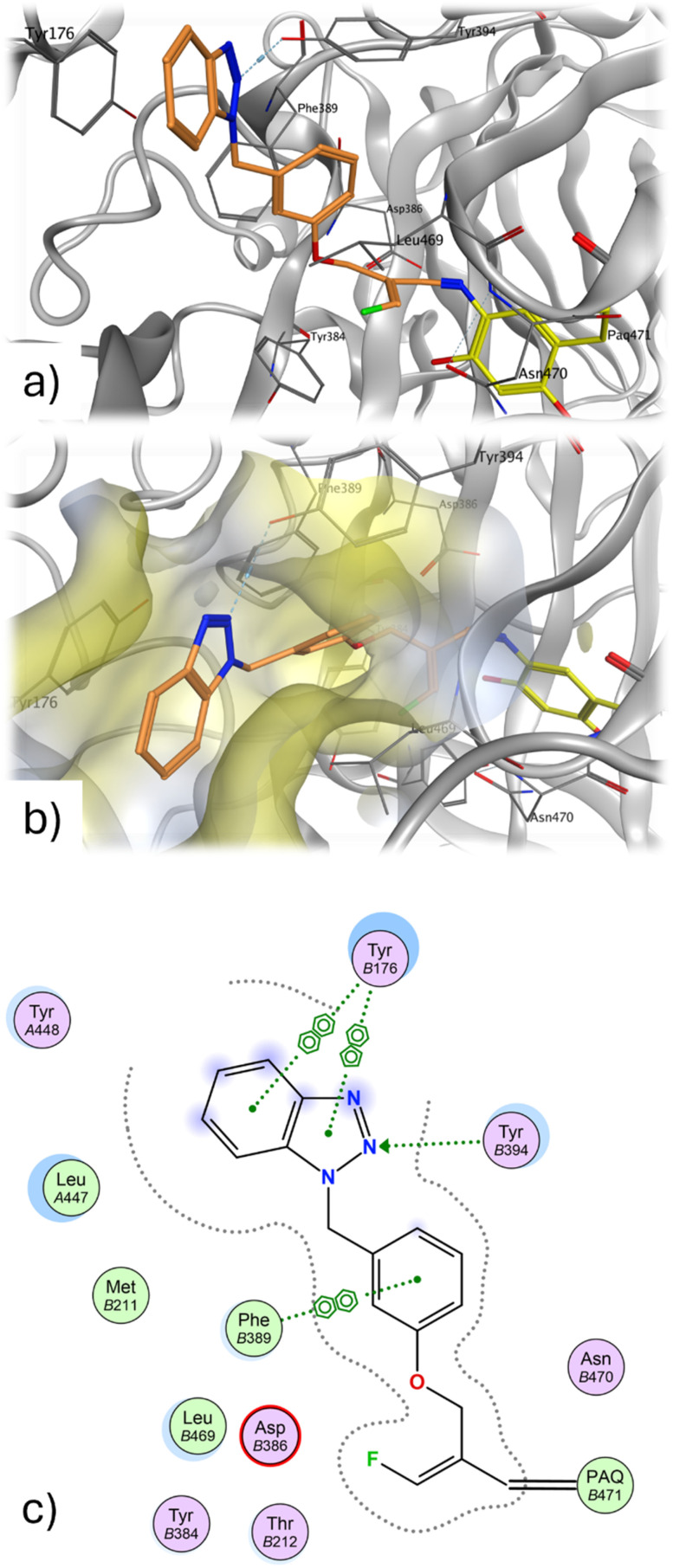
(a) Proposed binding mode of 94 (orange) at the topaquinone within the substrate binding site of human VAP-1. (b) The liophilic cavity of the benzotriazole is shown as yellow surface. (c) Two dimensional interaction diagram prepared in MOE; green dots represent lipophilic amino acids, polar amino acids are colored purple.

The docking analysis of the most potent fluoroallylamine, compound 94, with the human VAP-1 model is shown in Fig. 2. The illustrated binding represents the stage in which the amino group of the inhibitor has already formed a Schiff-base intermediate with topaquinone, but the electrophilic attack – leading to formation of the second covalent bond after fluoride elimination – has not yet occurred. The phenyl ring attached to the reactive fluoroallyl group *via* an oxygen atom occupies a lipophilic pocket with limited steric tolerance. π–π Interactions may occur between this ring and the phenyl group of Phe389. In contrast, the benzotriazole moiety, linked to the central phenyl ring through a methylene bridge, resides in a larger pocket. This region may stabilize the inhibitor through a hydrogen bond with Tyr394 and additional π–π interactions with Tyr176. The existence of a slightly larger binding pocket in this part of the enzyme is further supported by the observation that the bulkier 4-cyanophenyl-tetrazole group in compounds 87 and 89 results only in a moderate reduction in activity (Table 4). Overall, the binding model shows good agreement with the model described by Yamaki *et al.* for structurally related *N*-benzylglycine amides.^32^

In the case of the semicarbazide derivatives, it can likewise be assumed that the terminal amino group of the semicarbazide moiety undergoes covalent attachment to topaquinone, resulting in the formation of a semicarbazone. Owing to the structural similarity between compounds 84 and 94, it is plausible that the phenyl ring and benzotriazole heterocycle of 84 binds to the enzyme in a manner analogous to the corresponding groups in 94. Further analysis of the structure–activity relationships indicates that the central *meta*-substituted phenyl ring of 84 is not essential for inhibitory activity and can be replaced by a linear alkyl chain of appropriate length. Accordingly, the derivative containing a heptyl spacer between the benzotriazole heterocycle and the semicarbazide group (80) inhibits human VAP-1 with comparable potency (Table 4).

An analogous binding model can be proposed for the glycine amide derivatives. However, unlike the fluoroallylamines and semicarbazides, these compounds do not form irreversible bonds with the enzyme; instead, they are released as glyoxamides following oxidative cleavage. They therefore act as substrates that exhibit inhibitory effects. Notably, in contrast to the semicarbazides, the two glycine amide derivatives containing alkyl spacers between the glycine amide headgroup and the heterocycle (7 and 54) inhibit both human VAP-1 and bovine sVAP-1 considerably less potently than the corresponding compounds bearing *meta*-dimethylphenyl substituents (63 and 67) (Table 4). This reduced activity may be explained by the longer residence time of the phenyl-containing derivatives on the enzyme, potentially due to π–π interactions with phenylalanine residues of the enzyme. Consistent with this interpretation, the derivatives bearing simple alkyl chains are metabolised much more rapidly by bovine sVAP-1 than those with a *meta*-dimethylphenyl spacer.^44^

Selectivity testing revealed distinct inhibition profiles among the compound classes investigated. The representative glycine amides did not inhibit monoamine oxidase A (MAO A) or B (MAO B), and exhibited only weak inhibition of diamine oxidase (DAO). The semicarbazides also showed no effect on MAO enzymes, but inhibited DAO significantly more strongly than human VAP-1 with nanomolar IC_50_ values (Table 5). The synthesised fluoroallylamines (compounds 87, 89, 92, 94) showed broad activity across all four amine oxidases, though with notable selectivity differences. They emerged as potent dual inhibitors of human VAP-1 and MAO B, while exhibiting moderate to weak inhibition of DAO and MAO A. Their activity profile is broadly comparable to that of mofegiline (4), albeit with a less pronounced effect on MAO A.

**Table 5:** Selectivity of selected compounds for different amine oxidases evaluated with a 15 min pre-incubation of enzyme and inhibitor

Cpd.	Inhibition IC_50_^a^ (μM)			
	VAP-1	DAO	MAO A	MAO B
Glycine amides				
7	32	n.a.	n.a.	n.a.
54	39	>10	n.a.	n.a.
63	0.32	7.0	n.a.	n.a.
67	1.4	>10	n.a.	n.a.
Semicarbazides				
70	1.3	n.d.	n.d.	n.a.
80	0.18	0.0065	n.a.	n.a.
72	0.42	0.025	n.a.	n.a.
84	0.69	0.040	n.a.	n.a.
Fluoroallylamines				
87 (*E* : *Z* 95 : 5)	0.050	0.10	>10	0.013
89 (*E* : *Z* 0 : 100)	0.021	0.44	>10	0.0060
92 (*E* : *Z* 80 : 20)	0.0074	0.22	>10	0.0059
94 (*E* : *Z* 8 : 92)	0.0040	0.45	>10	0.0054
References				
1	2.6	>10	n.a.	n.a.
2 (PXS-4728)	0.0016	3.8	n.a.	2.3
4 (Mofegiline)	0.012	0.17	0.92	0.0007

a IC_50_-values of the compounds are the means of two independently performed determinations, errors are within ±20%; n.a.: not active at 10 μM; n.d.: not determined; enzyme sources: VAP-1 in human plasma, porcine kidney DAO, human recombinant MAO A and MAO B.

In contrast, the fluoroallylamine PXS-4728 (2) displayed a markedly higher selectivity for human VAP-1. Its high potency in human plasma at low concentrations – demonstrated by nearly complete inhibition of product formation at 0.010 μM – together with its poor inhibitory activity against DAO, MAO A, and MAO B, indicates that the amine oxidase activity in human blood plasma originates exclusively, or almost exclusively, from VAP-1. Thus, contrary to assumptions in the literature,^57,58^ DAO does not contribute to the amine oxidase activity in human plasma, as we had previously reported.^44^

Selected compounds were evaluated for phase I metabolic stability in rat liver microsomes using NADPH as a cofactor.^59,60^ Throughout the assay, only a small proportion of each compound was metabolised – generally less than 30%. The *E*-configured fluoroallylamines 87 and 92 exhibited particularly high stability, with more than 90% of the parent compound remaining. To verify assay performance, the drug imipramine was included as a positive control. Approximately 70% of the compound was metabolised, with desmethylimipramine identified as the main product, as expected.

As mentioned above, the most effective compounds produced, the fluoroallylamine derivatives, are covalent, irreversible inhibitors of the enzyme. However, such active substances often raise safety concerns due to their lack of specificity, which can lead to off-target effects and potential immunogenicity from protein–inhibitor adducts. Despite these concerns, covalent inhibitors offer notable advantages.^61–63^ For example, their irreversible binding to the target typically means that lower doses are required. Furthermore, because the binding is irreversible, these drugs can still inhibit their targets even when mutations cause a slight reduction in binding affinity. Notably, many enzyme inhibitors currently used in therapy are covalent binding agents. The goal in developing new active substances of this type is to design “targeted covalent inhibitors” that selectively react with specific nucleophilic residues near the binding site, thereby minimizing off-target effects. The high activity of the fluoroallylamines in the human VAP-1 assay, which uses human plasma containing the full complement of plasma proteins, together with their high stability in the rat liver S9 fraction (Table 6), suggests that these compounds exhibit no significant reactivity toward off-target bionucleophiles.

**Table 6:** Metabolic stability of selected compounds in rat liver S9 fraction

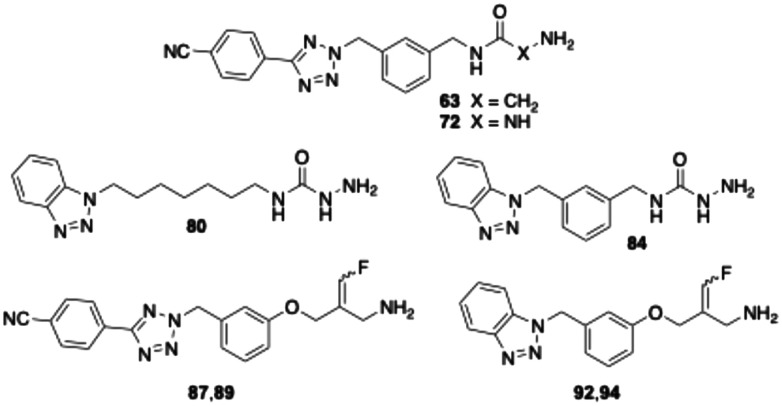	
Cpd.	Metabolic stability^a^ (%)
	IC_50_^a^ (μM)
63	75 ± 4
72	87 ± 10
80	78 ± 7
84	86 ± 1
87	94 ± 1
89	83 ± 1
92	90 ± 1
94	82 ± 6
Imipramine	28 ± 4

a Percent of parent remaining after incubation with rat liver S9 fraction for 30 min in presence of the cofactor NADPH; values are means ± standard deviations of independent determinations (*n* = 2), in case of imipramine: *n* = 4.

## Experimental

### Chemistry

#### General

Column chromatography was performed on silica gel 60, particle size 0.040-0.063 mm (Macherey & Nagel Düren, Germany) or on a Reveleris medium-pressure liquid chromatography system (Grace Davison Discovery Sciences, Deerfield, USA) applying pre-packed silica gel columns from Grace or Büchi (Büchi, Essen, Germany). Melting points were determined on a Büchi B-540 apparatus and are uncorrected. ^1^H and ^13^C NMR spectra were recorded an DD2 spectrometer (400 MHz) or an DD2 spectrometer (600 MHz) (Agilent, Santa Clara, USA). The high resolution mass spectra (HRMS) were recorded on a micrOTOF-Q II spectrometer (Bruker, Bremen, Germany) applying atmospheric pressure chemical ionization (APCI) with direct probe injection or electrospray ionization (ESI) of samples dissolved in a mixture of acetonitrile, aqueous ammonium formate (10 mM), and formic acid (90 : 10 : 0.1, v/v/v). Preparative reversed-phase HPLC was conducted using a Knauer Azura P2.1L pump, equipped with a Knauer RP18 Eurospher II 5 μm column (20 mm (I.D.) × 250 mm) and protected by a RP18 Eurospher II 5 μm guard column (20 mm (I.D.) × 30 mm) (Knauer, Berlin, Germany). The elution was carried out at a flow rate of 25 mL min^−1^. Detection was performed using a SPD-6A UV detector at 254 nm (Shimadzu Corporation, Tokyo, Japan). Chromatograms were recorded with MacDAcq32 Control Software (Bischoff, Leonberg, Germany). The compounds were dissolved in DMSO, and the sample volume injected was 500 μL. The substances were isolated by evaporating the organic solvent and freeze-drying the remaining aqueous phase using a Christ alpha 1-2 LD plus apparatus (Christ, Osterode am Harz, Germany). Purity of the target compounds was determined by reversed phase HPLC with UV detection. The samples were prepared by mixing 20 μL of a 5 mM solution of the compounds in DMSO with 180 μL acetonitrile. 5–20 μL of the solutions were injected into the HPLC system. Separation was performed using a Nucleosil 100 RP18 3 μm column (3 mm (I.D.) × 125 mm) (Macherey & Nagel, Düren, Germany) protected with a SecurityGuard™ cartridge C18 (3 mm (I.D.) × 4 mm) (Phenomenex, Aschaffenburg, Germany) at a flow rate of 0.4 mL min^−1^ with a gradient consisting of acetonitrile/water/trifluoroacetic acid (18 : 82 : 0.1 to 86 : 14 : 0.1, v/v/v) (method 1), acetonitrile/TRIS buffer (0.05 M, adjusted to pH 8.5 with 1 M HCl at 20 °C) (18 : 82 to 86 : 14, v/v) (method 2) or acetonitrile/water/trifluoroacetic acid (10 : 90 : 0.1 to 90 : 10 : 0.1, v/v/v) (method 3). UV-absorbance was measured at 254 nm.

For the syntheses of the target compounds not described below: see SI.

#### 2-Oxo-2-{[6-(5-phenyl-2*H*-tetrazol-2-yl)hexyl]amino}ethyl acetate 8

To a stirred solution of 6-(5-phenyl-2*H*-tetrazol-2-yl)hexan-1-amine^47^ (233 mg, 0.95 mmol), acetoxyacetic acid (124 mg, 1.05 mmol), and 1-hydroxybenzotriazole (88% (w/w), 161 mg, 1.05 mmol) in dry DMF (7 mL), *N*-(3-dimethylaminopropyl)-*N*′-ethylcarbodiimide hydrochloride (EDC-HCl) (201 mg, 1.05 mmol) was added. After stirring for further 15 h at room temperature, the reaction mixture was diluted with half-saturated sodium chloride solution (30 mL) and extracted exhaustively with ethyl acetate. The combined organic phases were washed twice with brine, dried over anhydrous sodium sulfate, and concentrated under reduced pressure. Purification by chromatography on silica gel (gradient elution: cyclohexane/ethyl acetate 5 : 5 to 2 : 8) yielded 8 (290 mg, 88%) as a solid. C_17_H_23_N_5_O_3_ (345.4); mp 44–45 °C; ^1^H NMR (400 MHz, CDCl_3_): *δ* (ppm) = 1.37–1.43 (m, 4H), 1.50–1.59 (m, 2H), 2.03–2.12 (m, 2H), 2.16 (s, 3H), 3.30 (td, *J* = 7.1 and 6.0 Hz, 2H), 4.54 (s, 2H), 4.66 (t, *J* = 7.0 Hz, 2H), 6.12 (s, 1H), 7.44–7.54 (m, 3H), 8.11–8.18 (m, 2H); HRMS (APCI, direct probe) *m*/*z* [M + H]^+^ calc.: 346.1874, found 346.1895.

#### 2-Hydroxy-*N*-[6-(5-phenyl-2*H*-tetrazol-2-yl)hexyl]acetamide 9

A solution of 8 (282 mg, 0.82 mmol) in methanol (5 mL) was treated with a solution of potassium carbonate (227 mg, 1.64 mmol) in water (5 mL) and stirred at 50 °C for 2 h. After removal of methanol under reduced pressure, the aqueous residue was exhaustively extracted with ethyl acetate. The combined organic layers were dried over anhydrous sodium sulfate, filtered, and concentrated *in vacuo*. Purification of the residue by chromatography on silica gel (gradient elution: cyclohexane/ethyl acetate 4 : 6 to ethyl acetate) afforded 9 as a solid (185 mg, 75%). C_15_H_21_N_5_O_2_ (303.4); mp 66–68 °C; ^1^H NMR (600 MHz, CDCl_3_): *δ* (ppm) = 1.35–1.43 (m, 4H), 1.51–1.58 (m, 2H), 2.04–2.11 (m, 2H), 3.31 (q, *J* = 6.7 Hz, 2H), 4.12 (s, 2H), 4.66 (t, *J* = 7.0 Hz, 2H), 6.47–6.59 (m, 1H), 7.45–7.51 (m, 3H,), 8.12–8.15 (m, 2H); HRMS (APCI, direct probe) *m*/*z* [M + H]^+^ calc.: 304.1768, found: 304.1770.

#### 2-Oxo-*N*-[6-(5-phenyl-2*H*-tetrazol-2-yl)hexyl]acetamide 10

A solution of 9 (175 mg, 0.58 mmol) in dry dichloromethane (15 mL) was slowly treated with Dess–Martin periodinane (369 mg, 0.87 mmol) at 0 °C and stirred at room temperature for 2 h. Saturated aqueous sodium bicarbonate solution (15 mL) and sodium thiosulfate pentahydrate (72 mg) were then added, and the mixture was stirred for an additional 30 min at room temperature. The organic phase was separated, and the aqueous layer was exhaustively extracted with dichloromethane. The combined organic layers were dried over anhydrous sodium sulfate, filtered, and concentrated under reduced pressure. The crude product was purified by chromatography on silica gel (gradient elution: cyclohexane/ethyl acetate 5 : 5 to ethyl acetate), affording 10 as an oil (86 mg, 49%). C_15_H_19_N_5_O_2_ (301.4); HPLC purity (method 1): 93%; ^1^H NMR (600 MHz, [D_6_]DMSO): *δ* (ppm) = 1.25–1.33 (m, 4H), 1.36–1.49 (m, 2H), 1.91–1.99 (m, 2H), 3.01–3.07 (m, 1H), 3.13 (td, *J* = 7.1 and 6.1 Hz, 1H), 4.69–4.74 (m, 2H), 4.88 (t, *J* = 6.7 Hz, 0.5H, HOCH̲OH), 6.29 (d, *J* = 6.8 Hz, 1H), 7.52–7.58 (m, 3H), 7.74 (t, *J* = 5.9 Hz, 0.5H, H̲OCHOH), 8.04–8.07 (m, 2H), 8.75 (t, *J* = 5.7 Hz, 0.5H, HOCHOH̲), 9.19 (s, 0.5H, CH̲O), about half of the compound was present as a hydrate. ^13^C NMR (151 MHz, [D_6_]DMSO): *δ* (ppm) = 25.4, 25.6, 28.3, 28.6, 28.7, 28.8, 38.0, 38.3, 52.7, 87.5 (COC̲OHOH, hydrate), 126.3, 127.0, 129.3, 130.5, 160.7 (C̲OCHO), 164.0, 170.7 (C̲OCOHOH, hydrate), 189.3 (COC̲HO); HRMS (APCI, direct probe) *m*/*z* [M + H]^+^ calc.: 302.1612, found: 302.1631.

#### 2-[6-(Indol-1-yl)hexyl]isoindoline-1,3-dione 11

A stirred solution of indole (400 mg, 3.41 mmol) in dry DMF (10 mL) was treated with sodium hydride (60%, dispersion in mineral oil, 164 mg, 4.10 mmol). After 30 min, 2-(6-bromohexyl)isoindoline-1,3-dione (1.05 g, 3.39 mmol) was added and stirring was continued for 16 h at room temperature. The mixture was then heated to 60 °C for 3 h. Upon cooling to room temperature, the mixture was diluted with water (50 mL) and exhaustively extracted with ethyl acetate. The combined organic phases were washed with water, dried over anhydrous sodium sulfate, and concentrated under reduced pressure. Purification by silica gel chromatography (gradient elution: cyclohexane to cyclohexane/ethyl acetate 9 : 1) afforded 11 as an oil (347 mg, 29%). C_22_H_22_N_2_O_2_ (346.4); ^1^H NMR (400 MHz, CDCl_3_): *δ* (ppm) = 1.33–1.42 (m, 4H), 1.60–1.70 (m, 2H), 1.80–1.88 (m, 2H), 3.66 (t, *J* = 7.1 Hz, 2H), 4.11 (t, *J* = 7.1 Hz, 2H), 6.47 (d, *J* = 3.1 Hz, 1H), 7.06–7.10 (m, 2H), 7.18 (ddd, *J* = 8.2, 7.0 and 1.2 Hz, 1H), 7.32 (dt, *J* = 8.2 and 1.0 Hz, 1H), 7.61 (dt, *J* = 7.9 and 1.0 Hz, 1H), 7.68–7.73 (m, 2H), 7.81–7.86 (m, 2H); HRMS (APCI, direct probe) *m*/*z* [M + H]^+^ calc.: 347.1754, found: 347.1722.

#### 6-(Indol-1-yl)hexan-1-amine 12

A solution of 11 (347 mg, 1.00 mmol) in ethanol (80 mL) was treated with hydrazine monohydrate (300 mg, 5.99 mmol) under stirring, and the mixture was heated under reflux for 5 h. The solvent was then removed under reduced pressure, and the resulting residue was treated with aqueous sodium hydroxide solution (50 mL, pH > 10). The mixture was exhaustively extracted with ethyl acetate. The combined organic phases were washed with aqueous sodium hydroxide solution, dried over anhydrous sodium sulfate, and concentrated under reduced pressure to yield 12 (216 mg, quantitative) as an oil. C_14_H_20_N_2_ (216.3); ^1^H NMR (400 MHz, CDCl_3_): *δ* (ppm) = 1.28–1.49 (m, 6H), 1.81–1.88 (m, 2H), 2.68 (t, *J* = 6.9 Hz, 2H), 4.12 (t, *J* = 7.1 Hz, 2H), 6.48 (dd, *J* = 3.1 and 0.9 Hz, 1H), 7.07–7.12 (m, 2H), 7.20 (ddd, *J* = 8.3, 7.0 and 1.2 Hz, 1H), 7.34 (dq, *J* = 8.3 and 0.9 Hz, 1H), 7.63 (dt, *J* = 7.9 and 1.0 Hz, 1H); HRMS (APCI, direct probe) *m*/*z* [M + H]^+^ calc.: 217.1699, found: 217.1723.

#### Benzyl (2-{[6-(indol-1-yl)hexyl]amino}-2-oxoethyl)carbamate 13

A solution of 12 (216 mg, 1.00 mmol), *N*-[(benzyloxy)carbonyl]glycine (230 mg, 1.10 mmol) and 1-hydroxybenzotriazole (88% (m/m), 169 mg, 1.10 mmol) in dry DMF (10 mL) was treated with EDC-HCl (211 mg, 1.10 mmol) and stirred at room temperature for 20 h. After completion, semi-saturated sodium bicarbonate solution (50 mL) was added, and the reaction mixture was exhaustively extracted with ethyl acetate. The combined organic phases were washed twice with saturated sodium bicarbonate solution, dried over anhydrous sodium sulfate, and concentrated *in vacuo*. The residue was purified by chromatography on silica gel (gradient elution: cyclohexane/ethyl acetate 7 : 3 to cyclohexane/ethyl acetate 2 : 8) to afford 13 (309 mg, 76%) as a solid. C_24_H_29_N_3_O_3_ (407.5); mp 79–80 °C; ^1^H NMR (400 MHz, CDCl_3_): *δ* (ppm) = 1.26–1.36 (m, 4H), 1.40–1.50 (m, 2H), 1.83 (p, *J* = 7.6 Hz, 2H), 3.20 (q, *J* = 6.6 Hz, 2H), 3.81 (d, *J* = 4.6 Hz, 2H), 4.11 (t, *J* = 7.1 Hz, 2H), 5.12 (s, 2H), 5.39 (s, 1H), 5.95 (s, 1H), 6.48 (d, *J* = 3.1 Hz, 1H), 7.06–7.12 (m, 2H), 7.20 (ddd, *J* = 8.2, 7.0 and 1.2 Hz, 1H), 7.28–7.38 (m, 6H), 7.63 (dt, *J* = 7.8, 1.0 Hz, 1H); HRMS (APCI, direct probe) *m*/*z* [M + H]^+^ calc.: 408.2282, found: 408.2303.

#### *N*-[6-(Indol-1-yl)hexyl]-2-aminoacetamide 14

A solution of 13 (290 mg, 0.71 mmol) in dry THF (10 mL) and methanol (10 mL) was treated with 10% palladium on carbon (32 mg) and stirred under a balloon filled with hydrogen at room temperature for 4 h. The resulting suspension was filtered through Celite® 545, and the filtrate was concentrated under reduced pressure to afford 14 (183 mg, 94%) as an oil. C_16_H_23_N_3_O (273.4); HPLC purity (method 1): 100%; ^1^H NMR (400 MHz, CDCl_3_): *δ* (ppm) = 1.28–1.36 (m, 4H), 1.43–1.50 (m, 2H), 1.78–1.86 (m, 2H), 3.21 (q, *J* = 6.8 Hz, 2H), 3.36 (s, 2H), 4.10 (t, *J* = 7.0 Hz, 2H), 6.48 (dd, *J* = 3.1 and 0.9 Hz, 1H), 7.06–7.12 (m, 2H), 7.20 (ddd, *J* = 8.3, 7.0 and 1.2 Hz, 1H), 7.27 (t, *J* = 5.6 Hz, 1H), 7.33 (dq, *J* = 8.2 and 0.9 Hz, 1H), 7.62 (dt, *J* = 7.9 and 1.1 Hz, 1H); ^13^C NMR (101 MHz, CDCl_3_): *δ* (ppm) = 26.72, 26.83, 29.6, 30.3, 39.0, 44.5, 46.4, 101.1, 109.5, 119.3, 121.1, 121.5, 127.9, 128.7, 136.1, 172.1; HRMS (APCI, direct probe) *m*/*z* [M + H]^+^ calc.: 274.1914, found: 274.1925.

#### 2-[6-(Benzimidazol-1-yl)hexyl]isoindoline-1,3-dione 23

To a stirred solution of benzimidazole (295 mg, 2.50 mmol) in dry DMF (5 mL), sodium hydride (60% dispersion in mineral oil, 120 mg, 3.00 mmol) was added portionwise at room temperature. The mixture was then heated to 60 °C for 30 min. Subsequently, 2-(6-bromohexyl)isoindoline-1,3-dione (775 mg, 2.50 mmol) was added, and the mixture was heated at 60 °C for an additional 4 h. After completion, the reaction mixture was diluted with water (30 mL) and exhaustively extracted with ethyl acetate. The combined organic phases were washed with water, dried over anhydrous sodium sulfate, and concentrated *in vacuo*. Chromatography on silica gel (gradient elution: cyclohexane/ethyl acetate 7 : 3 + 1% triethylamine to cyclohexane/ethyl acetate 4 : 6 + 1% triethylamine) afforded 23 (649 mg, 75%) as an oil. C_21_H_21_N_3_O_2_ (347.4); ^1^H NMR (400 MHz, CDCl_3_): *δ* (ppm) = 1.39 (p, *J* = 3.7 Hz, 4H), 1.67 (p, *J* = 7.1 Hz, 2H), 1.89 (p, *J* = 7.1 Hz, 2H), 3.66 (t, *J* = 7.1 Hz, 2H), 4.17 (t, *J* = 7.2 Hz, 2H), 7.25–7.32 (m, 2H), 7.36–7.41 (m, 1H), 7.67–7.73 (m, 2H), 7.78–7.86 (m, 3H), 7.97 (s, 1H); HRMS (APCI, direct probe) *m*/*z* [M + H]^+^ calc.: 348.1707, found: 348.1720.

#### 6-(Benzimidazol-1-yl)hexan-1-amine 24

Compound 23 (629 mg, 1.81 mmol) was reacted with hydrazine monohydrate (544 mg, 10.9 mmol) following the same procedure described for the synthesis of 12, affording 24 (262 mg, 67%) as an oil. C_13_H_19_N_3_ (217.3); ^1^H NMR (400 MHz, CDCl_3_): *δ* (ppm) = 1.27–1.48 (m, 6H), 1.85–1.92 (m, 2H), 2.66 (t, *J* = 6.8 Hz, 2H), 4.15 (t, *J* = 7.1 Hz, 2H), 7.23–7.30 (m, 2H), 7.35–7.39 (m, 1H), 7.75–7.81 (m, 1H), 7.89 (s, 1H); HRMS (APCI, direct probe) *m*/*z* [M + H]^+^ calc.: 218.1652, found: 218.1668.

#### *tert*-Butyl (2-{[6-(benzimidazol-1-yl)hexyl]amino}-2-oxoethyl)carbamate 25

A solution of 24 (105 mg, 0.48 mmol), *N*-(*tert*-butoxycarbonyl)glycine (93 mg, 0.53 mmol) and 1-hydroxybenzotriazole (88% (m/m), 81 mg, 0.53 mmol) in dry DMF (5 mL) was treated with EDC-HCl (102 mg, 0.53 mmol) and stirred at room temperature for 18 h. The reaction mixture was diluted with semi-saturated aqueous sodium bicarbonate solution and exhaustively extracted with ethyl acetate. The combined organic phases were washed twice with saturated aqueous sodium bicarbonate solution, dried over anhydrous sodium sulfate, and concentrated under reduced pressure. The residue was chromatographed on silica gel (ethyl acetate/methanol 92.5 : 7.5 + 1% triethylamine) to afford 25 (144 mg, 80%) as an oil. C_20_H_30_N_4_O_3_ (374.5); ^1^H NMR (400 MHz, [D_6_]DMSO): *δ* (ppm) = 1.20–1.40 (m, 15H), 1.77 (p, *J* = 7.2 Hz, 2H), 3.02 (q, *J* = 6.5 Hz, 2H), 3.47 (d, *J* = 6.4 Hz, 2H), 4.23 (t, *J* = 7.1 Hz, 2H), 6.86 (t, *J* = 6.1 Hz, 1H), 7.17–7.27 (m, 2H), 7.57–7.69 (m, 3H), 8.21 (s, 1H); HRMS (APCI, direct probe) *m*/*z* [M + H]^+^ calc.: 375.2391, found: 375.2377.

#### *N*-[6-(Benzimidazol-1-yl)hexyl]-2-aminoacetamide dihydrochloride 26

A solution of 25 (132 mg, 0.35 mmol) in ethyl acetate (4 mL) was treated with 4 M HCl in cyclopentyl methyl ether (3 mL) and stirred at room temperature for 3 h. The solvent was then removed *in vacuo*, and the resulting residue was dispersed in ethyl acetate. After filtration, the filter cake was dried under reduced pressure to give 26 as a solid (71 mg, 58%). C_15_H_24_Cl_2_N_4_O (347.3); HPLC purity (method 2): 100%; ^1^H NMR (600 MHz, [D_6_]DMSO): *δ* (ppm) = 1.27–1.37 (m, 4H), 1.42 (p, *J* = 7.0 Hz, 2H), 1.90 (p, *J* = 7.0 Hz, 2H), 3.09 (td, *J* = 6.8 Hz, and 5.4 Hz, 2H), 3.50 (q, *J* = 5.7 Hz, 2H), 4.48 (t, *J* = 7.2 Hz, 2H), 7.56–7.65 (m, 2H), 7.85–7.89 (m, 1H), 8.01–8.05 (m, 1H), 8.15 (s, 3H), 8.49 (t, *J* = 5.6 Hz, 1H), 9.65 (s, 1H); ^13^C NMR (151 MHz, [D_6_]DMSO): *δ* (ppm) = 25.4, 25.7, 28.5, 28.6, 38.5, 40.0, 46.2, 113.2, 115.1, 125.9, 126.2, 131.1, 131.3, 141.4, 165.6; HRMS (APCI, direct probe) *m*/*z* [C_15_H_22_N_4_O + H]^+^ calc.: 275.1866, found: 275.1885; elemental analysis calcd. for C_15_H_24_Cl_2_N_4_O: C 51.88, H 6.97, N 16.13, found: C 51.66, H 6.93, N 15.96.

#### 2-[6-(1*H*-Benzotriazol-1-yl)hexyl]isoindoline-1,3-dione 27, 2-[6-(2*H*-benzotriazol-2-yl)hexyl]isoindoline-1,3-dione 28

A mixture of benzotriazole (298 mg, 2.50 mmol), 2-(6-bromohexyl)isoindoline-1,3-dione (775 mg, 2.50 mmol), potassium carbonate (691 mg, 5.00 mmol) and acetonitrile (30 mL) was heated under reflux for 6.5 h. After cooling to room temperature, the mixture was filtered and the filtrate concentrated under reduced pressure. The residue was purified by chromatography on silica gel (gradient elution: cyclohexane to cyclohexane/ethyl acetate 7 : 3), yielding the regio isomers 27 (455 mg, 52%) and 28 (337 mg, 39%) as solids. 27: C_20_H_20_N_4_O_2_ (348.4); mp 81–82 °C; ^1^H NMR (400 MHz, CDCl_3_): *δ* (ppm) = 1.35–1.45 (m, 4H), 1.61–1.70 (m, 2H), 1.97–2.06 (m, 2H), 3.66 (t, *J* = 7.2 Hz, 2H), 4.63 (t, *J* = 7.2 Hz, 2H), 7.36 (ddd, *J* = 8.1, 6.6 and 1.3 Hz, 1H), 7.44–7.53 (m, 2H), 7.67–7.73 (m, 2H), 7.80–7.85 (m, 2H), 8.05 (dt, *J* = 8.4 and 1.0 Hz, 1H); HRMS (APCI, direct probe) *m*/*z* [M + H]^+^ calc.: 349.1659, found: 349.1681. 28: C_20_H_20_N_4_O_2_ (348.4); mp 102–103 °C; ^1^H NMR (600 MHz, CDCl_3_): *δ* (ppm) = 1.37–1.44 (m, 4H), 1.63–1.70 (m, 2H), 2.09–2.15 (m, 2H), 3.66 (t, *J* = 7.2 Hz, 2H), 4.71 (t, *J* = 7.1 Hz, 2H), 7.34–7.38 (m, 2H), 7.68–7.71 (m, 2H), 7.81–7.86 (m, 4H); HRMS (APCI, direct probe) *m*/*z* [M + H]^+^ calc.: 349.1659, found: 349.1667.

#### 6-(1*H*-Benzotriazol-1-yl)hexan-1-amine 29

Compound 27 (430 mg, 1.23 mmol) was reacted with hydrazine monohydrate (370 mg, 7.39 mmol) following the same procedure described for the synthesis of compound 12, affording 29 (225 mg, 84%) as an oil. C_12_H_18_N_4_ (218.3); ^1^H NMR (400 MHz, CDCl_3_): *δ* (ppm) = 1.31–1.49 (m, 6H), 2.02 (p, *J* = 7.1 Hz, 2H), 2.64–2.69 (m, 2H), 4.63 (t, *J* = 7.1 Hz, 2H), 7.36 (ddd, *J* = 8.0, 6.6 and 1.3 Hz, 1H), 7.45–7.54 (m, 2H), 8.05 (dt, *J* = 8.3 and 1.0 Hz, 1H); HRMS (APCI, direct probe) *m*/*z* [M + H]^+^ calc.: 219.1604, found: 219.1619.

#### *tert*-Butyl (2-{[6-(1*H*-benzotriazol-1-yl)hexyl]amino}-2-oxoethyl)carbamate 30

A solution of 29 (210 mg, 0.96 mmol), *N*-(*tert*-butoxycarbonyl)glycine (186 mg, 1.06 mmol), and 1-hydroxybenzotriazole (88% purity, 163 mg, 1.06 mmol) in dry DMF (7 mL) was treated with EDC-HCl (203 mg, 1.06 mmol) and stirred at room temperature for 19 h. The reaction mixture was then diluted with a semi-saturated aqueous solution of sodium bicarbonate (30 mL) and exhaustively extracted with ethyl acetate. The combined organic layers were washed twice with saturated aqueous sodium bicarbonate solution, dried over anhydrous sodium sulfate, and concentrated under reduced pressure. Purification by chromatography on silica gel (gradient elution: cyclohexane/ethyl acetate 5 : 5 to 8 : 2) afforded compound 30 as an oil (315 mg, 87%). C_19_H_29_N_5_O_3_ (375.5); ^1^H NMR (400 MHz, CDCl_3_): *δ* (ppm) = 1.33–1.38 (m, 4H), 1.42–1.52 (m, 11H), 2.02 (p, *J* = 7.0 Hz, 2H), 3.23 (q, *J* = 6.6 Hz, 2H), 3.77 (s, 2H), 4.65 (t, *J* = 7.1 Hz, 2H), 5.20 (s, 1H), 6.20 (s, 1H), 7.39 (ddd, *J* = 8.4, 6.5 and 2.3 Hz, 1H), 7.48–7.56 (m, 2H), 8.08 (dt, *J* = 8.4 and 1.0 Hz, 1H); HRMS (APCI, direct probe) *m*/*z* [M + H]^+^ calc.: 376.2343, found: 376.2350.

#### *N*-[6-(1*H*-Benzotriazol-1-yl)hexyl]-2-aminoacetamide dihydrochloride 31

Compound 30 (125 mg, 0.33 mmol) was subjected to the same reaction conditions used for the synthesis of 26, resulting in the formation of 31 (75 mg, 65%) as a solid. C_14_H_23_Cl_2_N_5_O (348.3); HPLC purity (method 1): 100%; ^1^H NMR (400 MHz, [D_6_]DMSO): *δ* (ppm) = 1.20–1.42 (m, 6H), 1.91 (p, *J* = 7.1 Hz, 2H), 3.07 (q, *J* = 6.4 Hz, 2H), 3.49 (q, *J* = 5.8 Hz, 2H), 4.71 (t, *J* = 7.0 Hz, 2H), 7.40 (ddd, *J* = 8.1, 6.9 and 1.0 Hz, 1H), 7.55 (ddd, *J* = 8.2, 6.9 and 1.0 Hz, 1H), 7.90 (dt, *J* = 8.4 and 1.0 Hz, 1H), 8.03 (dt, *J* = 8.4 and 1.0 Hz, 1H), 8.13 (s, 3H), 8.42 (t, *J* = 5.6 Hz, 1H); ^13^C NMR (101 MHz, [D_6_]DMSO): *δ* (ppm) = 25.67, 25.69, 28.7, 29.1, 38.5, 40.0, 47.3, 110.6, 119.1, 123.9, 127.1, 132.8, 145.1, 165.6; HRMS (APCI, direct probe) *m*/*z* [C_14_H_21_N_5_O + H]^+^ calc.: 276.1819, found: 276.1823.

#### 6-(2*H*-Benzotriazol-2-yl)hexan-1-amine 32

Compound 28 (470 mg, 1.35 mmol) was reacted with hydrazine monohydrate (273 mg, 5.45 mmol) following the same procedure described for the synthesis of 12, affording 32 (182 mg, 62%) as an oil. C_12_H_18_N_4_ (218.3); ^1^H NMR (400 MHz, CDCl_3_): *δ* (ppm) = 1.32–1.49 (m, 6H), 2.12 (p, *J* = 7.3 Hz, 2H), 2.62–2.73 (m, 2H), 4.72 (t, *J* = 7.1 Hz, 2H), 7.34–7.40 (m, 2H), 7.82–7.88 (m, 2H); HRMS (APCI, direct probe) *m*/*z* [M + H]^+^ calc.: 219.1604, found: 219.1615.

#### *tert*-Butyl (2-{[6-(2*H*-benzotriazol-2-yl)hexyl]amino}-2-oxoethyl)carbamate 33

Compound 32 (172 mg, 0.79 mmol) was treated with *N*-(*tert*-butoxycarbonyl)glycine (152 mg, 0.87 mmol) under conditions analogous to those employed in the synthesis of 25. Purification by chromatography on silica gel (gradient elution: cyclohexane/ethyl acetate 6 : 4 to 3 : 7) afforded 33 (225 mg, 76%) as an oil. C_19_H_29_N_5_O_3_ (375.5); ^1^H NMR (400 MHz, CDCl_3_): *δ* (ppm) = 1.32–1.54 (m, 15H), 2.11 (p, *J* = 7.2 Hz, 2H_2_), 3.24 (q, *J* = 6.6 Hz, 2H), 3.76 (s, 2H), 4.72 (t, *J* = 7.0 Hz, 2H), 5.15 (s, 1H), 6.18 (s, 1H), 7.34–7.40 (m, 2H), 7.82–7.88 (m, 2H); HRMS (APCI, direct probe) *m*/*z* [M + H]^+^ calc.: 376.2343, found: 376.2361.

#### *N*-[6-(2*H*-Benzotriazol-2-yl)hexyl]-2-aminoacetamide hydrochloride 34

Compound 33 (112 mg, 0.30 mmol) was subjected to the same reaction conditions used for the synthesis of 26, resulting in the formation of 34 (56 mg, 60%) as a solid. C_14_H_22_ClN_5_O (311.8); HPLC purity (method 1): 100%; ^1^H NMR (600 MHz, [D_6_]DMSO): *δ* (ppm) = 1.23–1.35 (m, 4H), 1.39 (p, *J* = 7.1 Hz, 2H), 2.02 (p, *J* = 7.1 Hz, 2H), 3.08 (q, *J* = 6.5 Hz, 2H), 3.49 (s, 2H), 4.74 (t, *J* = 7.0 Hz, 2H), 7.41–7.45 (m, 2H), 7.90–7.93 (m, 2H), 8.08 (s, 3H), 8.38 (t, *J* = 5.6 Hz, 1H); ^13^C NMR (151 MHz, [D_6_]DMSO): *δ* (ppm) = 25.60, 25.68, 28.6, 29.3, 38.5, 40.1, 55.9, 117.8, 126.2, 143.6, 165.6; HRMS (APCI, direct probe) *m*/*z* [C_14_H_21_N_5_O + H]^+^ calc.: 276.1819, found: 276.1822. Elemental analysis calcd. for C_14_H_22_ClN_5_O: C 53.93, H 7.11, N 22.46, found: C 53.60, H 7.11, N 22.43.

#### Ethyl [7-(1*H*-benzotriazol-1-yl)heptyl]carbamate 59

A solution of 52 (234 mg, 1.01 mmol) (see SI) in dichloromethane (6 mL) and triethylamine (1 mL) was treated dropwise with ethyl chloroformate (124 μL, 1.30 mmol) at 0 °C. The mixture was stirred at 0 °C for 30 min and then at room temperature for 2 h. The crude product was purified by chromatography on silica gel (gradient elution: cyclohexane to cyclohexane/ethyl acetate 1 : 1) to afford 59 (234 mg, 76%) as a solid. C_16_H_24_N_4_O_2_ (304.4); mp 47–49 °C; ^1^H NMR (400 MHz, CDCl_3_): *δ* (ppm) = 1.19–1.38 (m, 9H), 1.45 (p, *J* = 7.2 Hz, 2H), 2.01 (p, *J* = 7.0 Hz, 2H), 3.12 (q, *J* = 6.3 Hz, 2H), 4.09 (q, *J* = 7.1 Hz, 2H), 4.55–4.67 (m, 3H), 7.37 (ddd, *J* = 8.4, 6.9 and 1.4 Hz, 1H), 7.46–7.54 (m, 2H), 8.07 (dt, *J* = 8.4 and 0.9 Hz, 1H); HRMS (APCI, direct probe) *m*/*z* [M + H]^+^ calc.: 305.1972, found: 305.1956.

#### 7-(1*H*-Benzotriazol-1-yl)-*N*-methylheptan-1-amine 60

A solution of 59 (230 mg, 0.76 mmol) in dry THF (10 mL) was treated dropwise with 2 M lithium aluminium hydride in THF (1.60 mL, 3.20 mmol) at 0 °C and then heated under reflux for 3 h. The reaction mixture was cooled to 0 °C, and water (500 μL), aqueous sodium hydroxide solution (15% (m/V), 500 μL) and water (1500 μL) were added dropwise at 5 min intervals. After addition of anhydrous sodium sulfate, the mixture was stirred for a further 15 min and filtered. The filtrate was concentrated under reduced pressure and the residue was cromatographed on silica gel (dichloromethane/methanol 9 : 1 + 1% triethylamine) to afford 60 (151 mg, 81%) as an oil. C_14_H_22_N_4_ (246.4); ^1^H NMR (400 MHz, CDCl_3_): *δ* (ppm) = 1.29–1.39 (m, 6H), 1.63 (p, *J* = 7.4 Hz, 2H), 2.00 (p, *J* = 7.1 Hz, 2H), 2.52 (s, 3H), 2.71 (t, *J* = 7.4 Hz, 2H), 4.62 (t, *J* = 7.1 Hz, 2H), 7.36 (ddd, *J* = 8.4, 6.6 and 1.3 Hz, 1H), 7.44–7.55 (m, 2H,), 8.05 (dt, *J* = 8.4 and 1.0 Hz, 1H); HRMS (APCI, direct probe) *m*/*z* [M + H]^+^ calc.: 247.1917, found: 247.1985.

#### *tert*-Butyl (2-{[7-(1*H*-benzotriazol-1-yl)heptyl](methyl)amino}-2-oxoethyl)carbamate 61

Compound 60 (145 mg, 0.59 mmol) was treated with *N*-(*tert*-butoxycarbonyl)glycine (114 mg, 0.65 mmol) under conditions analogous to those employed in the synthesis of 25. Purification by chromatography on silica gel (gradient elution: cyclohexane/ethyl acetate 6 : 4 to 2 : 8) afforded 61 (179 mg, 75%) as an oil. C_21_H_33_N_5_O_3_ (403.5); ^1^H NMR (400 MHz, CDCl_3_): *δ* (ppm) = 1.22–1.39 (m, 6H), 1.44 (m, 11H), 1.96–2.05 (m, 2H), 2.89 (s, 1H, NCH̲_3minor_), 2.91 (s, 2H, NCH̲_3major_), 3.14 (t, *J* = 7.6 Hz, 0.67H, heptyl 1-CH̲_2minor_), 3.34 (t, *J* = 7.5 Hz, 1.33H, heptyl 1-CH̲_2major_), 3.92 (d, *J* = 8.3 Hz, 2H), 4.64 (td, *J* = 7.1 and 5.0 Hz, 2H), 5.52 (s, 1H), 7.37 (dddd, *J* = 8.3, 6.6, 1.8 and 1.4 Hz, 1H), 7.46–7.55 (m, 2H), 8.07 (ddt, *J* = 8.4, 2.0 and 1.0 Hz, 1H); HRMS (APCI, direct probe) *m*/*z* [M + H]^+^ calc.: 404.2656, found: 404.2670.

#### *N*-[7-(1*H*-Benzotriazol-1-yl)heptyl]-2-amino-*N*-methylacetamide hydrochloride 62

A solution of 61 (174 mg, 0.43 mmol) in ethyl acetate (2 mL) was treated with 4 M HCl in cyclopentyl methyl ether (3 mL) and stirred at room temperature for 4 h. The solvent was then removed under reduced pressure. The resulting residue was dispersed in ethyl acetate and sonicated for 5 min. After decanting the supernatant, the procedure was repeated twice. The remaining oily residue was dried under reduced pressure to yield 62 (70 mg, 48%) as a highly viscous oil. C_16_H_26_ClN_5_O (339.9); HPLC purity (method 2): 100%; ^1^H NMR (600 MHz, [D_6_]DMSO): *δ* (ppm) = 1.14–1.34 (m, 6H), 1.37–1.49 (m, 2H), 1.88–1.95 (m, 2H), 2.83 (s, 1H, NCH̲_3minor_), 2.88 (s, 2H, NCH̲_3major_), 3.17 (t, *J* = 7.7 Hz, 0.67H, heptyl 1-CH̲_2minor_), 3.28 (t, *J* = 7.2 Hz, 1.33H, heptyl 1-CH̲_2major_), 3.77 (s, 0.67H, COCH̲_2minor_), 3.79 (s, 1.33H, COCH̲_2major_), 4.71 (td, *J* = 6.9 and 5.4 Hz, 2H), 7.40 (ddd, *J* = 8.2, 6.9 and 1.0 Hz, 1H), 7.55 (ddt, *J* = 8.1, 6.9 and 1.1 Hz, 1H), 7.90 (ddt, *J* = 8.4, 4.2 and 1.0 Hz, 1H), 8.03 (dt, *J* = 8.4 and 0.9 Hz, 1H), 8.13 (s, 3H); ^13^C NMR (151 MHz, [D_6_]DMSO): *δ* (ppm) = 25.8, 25.91, 25.96, 26.5, 27.2, 28.1, 28.2, 29.06, 29.10, 32.8, 33.6, 39.1, 47.0, 47.36, 47.4, 48.0, 110.6, 119.1, 123.9, 127.1, 132.8, 145.1, 165.3, 165.7; HRMS (APCI, direct probe) *m*/*z* [C_16_H_25_N_5_O + H]^+^ calc.: 304.2132, found: 304.2145.

#### 2-{3-[(1*H*-Benzotriazol-1-yl)methyl]benzyl}isoindoline-1,3-dione 64

A mixture of benzotriazole (97 mg, 0.81 mmol), 2-[3-(bromomethyl)benzyl]isoindoline-1,3-dione^48^ (268 mg, 0.81 mmol), potassium carbonate (224 mg, 1.62 mmol) and acetonitrile (10 mL) was heated under reflux for 16 h. After cooling to room temperature, the reaction mixture was filtered and the filtrate concentrated under reduced pressure. The residue was chromatographed on silica gel (cyclohexane/ethyl acetate 7 : 3) to yield 64 (194 mg, 65%) as a solid. C_22_H_16_N_4_O_2_ (368.4); mp 152–153 °C; ^1^H NMR (400 MHz, CDCl_3_): *δ* (ppm) = 4.82 (s, 2H), 5.82 (s, 2H), 7.10 (dt, *J* = 7.8 and 1.3 Hz, 1H), 7.23–7.40 (m, 5H), 7.42–7.45 (m, 1H), 7.69–7.75 (m, 2H), 7.82–7.87 (m, 2H), 8.05 (dt, *J* = 8.1 and 1.1 Hz, 1H); HRMS (APCI, direct probe) *m*/*z* [M + H]^+^ calc.: 369.1346, found: 369.1316.

#### {3-[(1*H*-Benzotriazol-1-yl)methyl]phenyl}methanamine 65

Compound 64 (172 mg, 0.47 mmol) was reacted with hydrazine monohydrate (141 mg, 2.82 mmol) following the same procedure described for the synthesis of 12, affording 65 (102 mg, 92%) as an oil. C_14_H_14_N_4_ (238.3); ^1^H NMR (400 MHz, CDCl_3_): *δ* (ppm) = 3.84 (s, 2H), 5.83 (s, 2H), 7.13–7.17 (m, 1H), 7.26–7.43 (m, 6H), 8.06 (dt, *J* = 8.2 and 1.0 Hz, 1H); HRMS (APCI, direct probe) *m*/*z* [M + H]^+^ calc.: 239.1291, found: 239.1292.

#### *tert*-Butyl [2-({3-[(1*H*-benzotriazol-1-yl)methyl]benzyl}amino)-2-oxoethyl]carbamate 66

Compound 65 (91 mg, 0.38 mmol) was treated with *N*-(*tert*-butoxycarbonyl)glycine (74 mg, 0.42 mmol) under conditions analogous to those employed in the synthesis of 25. Purification by chromatography on silica gel (cyclohexane/ethyl acetate 3 : 7) afforded 66 (127 mg, 84%) as an oil. C_21_H_25_N_5_O_3_ (395.5); ^1^H NMR (400 MHz, CDCl_3_): *δ* (ppm) = 1.41 (s, 9H), 3.80 (d, *J* = 4.1 Hz, 2H), 4.41 (d, *J* = 5.9 Hz, 2H), 5.14 (s, 1H), 5.81 (s, 2H), 6.52 (s, 1H), 7.14 (dt, *J* = 7.6 and 1.6 Hz, 1H), 7.20–7.23 (m, 2H), 7.25–7.30 (m, 1H), 7.33–7.45 (m, 3H), 8.06 (dt, *J* = 8.3 and 1.0 Hz, 1H); HRMS (APCI, direct probe) *m*/*z* [M + H]^+^ calc.: 396.2030, found: 396.2008.

#### *N*-{3-[(1*H*-Benzotriazol-1-yl)methyl]benzyl}-2-aminoacetamide dihydrochloride 67

Compound 66 (117 mg, 0.30 mmol) was subjected to the same reaction conditions used for the synthesis of 26, resulting in the formation of 67 (66 mg, 61%) as a solid. C_16_H_19_Cl_2_N_5_O (368.3); HPLC purity (method 2): 100%; ^1^H NMR (600 MHz, [D_6_]DMSO): *δ* (ppm) = 3.57 (q, *J* = 5.8 Hz, 2H), 4.30 (d, *J* = 5.9 Hz, 2H), 5.97 (s, 2H), 7.20 (dt, *J* = 7.6 and 1.6 Hz, 1H), 7.24 (dt, *J* = 7.8 and 1.5 Hz, 1H), 7.31 (t, *J* = 7.6 Hz, 1H), 7.34 (t, *J* = 1.9 Hz, 1H), 7.40 (ddd, *J* = 8.1, 6.9 and 1.0 Hz, 1H), 7.54 (ddd, *J* = 8.2, 6.9 and 0.9 Hz, 1H), 7.87 (dt, *J* = 8.4 and 1.0 Hz, 1H), 8.05 (dt, *J* = 8.4 and 1.0 Hz, 1H), 8.23 (s, 3H), 8.98 (t, *J* = 6.0 Hz, 1H); ^13^C NMR (151 MHz, [D_6_]DMSO): *δ* (ppm) = 40.1, 42.0, 50.9, 110.8, 119.2, 124.1, 126.5, 126.9, 127.2, 127.5, 128.9, 132.7, 136.0, 139.4, 145.3, 166.0; HRMS (APCI, direct probe) *m*/*z* [C_16_H_18_N_5_O + H]^+^ calc.: 296.1506, found: 296.1520.

#### Phenyl [6-(1*H*-benzotriazol-1-yl)hexyl]carbamate 77

Diphenyl carbonate (253 mg, 1.18 mmol) was ground and suspended in a mixture of THF and water (1 : 9, 3 mL). A suspension of 29 (244 mg, 1.12 mmol) in THF/water (1 : 9, 3 mL) was added dropwise under stirring, and the mixture was further stirred at 40 °C for 2 h. Upon completion of the reaction, the mixture was diluted with water and exhaustively extracted with ethyl acetate. The combined organic layers were dried over anhydrous sodium sulfate and concentrated under reduced pressure. The crude product was purified by chromatography on silica gel (gradient elution: cyclohexane to cyclohexane/ethyl acetate 1 : 1), affording 77 (328 mg, 87%) as a solid. C_19_H_22_N_4_O_2_ (338.4); mp 84–86 °C; ^1^H NMR (400 MHz, CDCl_3_): *δ* (ppm) = 1.34–1.47 (m, 4H), 1.55 (p, *J* = 7.0 Hz, 2H), 2.04 (p, *J* = 7.2 Hz, 2H), 3.24 (q, *J* = 6.6 Hz, 2H), 4.66 (t, *J* = 7.1 Hz, 2H), 5.04 (s, 1H), 7.08–7.13 (m, 2H), 7.15–7.21 (m, 1H), 7.31–7.42 (m, 3H), 7.47–7.56 (m, 2H), 8.08 (dt, *J* = 8.4 and 0.9 Hz, 1H); HRMS (APCI, direct probe) *m*/*z* [M + H]^+^ calc.: 339.1816, found: 339.1810.

#### *N*-[6-(1*H*-Benzotriazol-1-yl)hexyl]hydrazinecarboxamide 78

A solution of 77 (308 mg, 0.91 mmol) in 1,2-dimethoxyethane (2.5 mL) was treated with hydrazine monohydrate (456 mg, 9.11 mmol) and stirred at 80 °C for 6 h. After completion, the crude product was purified by chromatography on silica gel (gradient elution: dichloromethane to dichloromethane/methanol 9 : 1). The product-containing fractions were combined and concentrated to a few mL under reduced pressure. By adding ethyl acetate, the desired product 78 (128 mg, 51%) precipitated. C_13_H_20_N_6_O (276.3); mp 118–120 °C; HPLC purity (method 1): 98%; ^1^H NMR (600 MHz, [D_6_]DMSO): *δ* (ppm) = 1.21–1.36 (m, 6H), 1.90 (p, *J* = 7.1 Hz, 2H), 2.97 (td, *J* = 7.0 and 6.3 Hz, 2H), 4.03 (s, 2H), 4.70 (t, *J* = 7.0 Hz, 2H), 6.26 (s, 1H), 6.82 (s, 1H), 7.40 (ddd, *J* = 8.3, 6.9 and 1.0 Hz, 1H), 7.55 (ddd, *J* = 8.3, 6.9 and 1.1 Hz, 1H), 7.89 (dt, *J* = 8.4 and 1.0 Hz, 1H), 8.03 (dt, *J* = 8.4 and 1.0 Hz, 1H); ^13^C NMR (151 MHz, [D_6_]DMSO): *δ* (ppm) = 25.76, 25.83, 29.2, 29.9, 38.7, 47.4, 110.6, 119.1, 123.89, 127.1, 132.8, 145.1, 160.2; HRMS (APCI, direct probe) *m*/*z* [M + H]^+^ calc.: 277.1771, found: 277.1772.

#### 4-[2-(3-Hydroxybenzyl)-2*H*-tetrazol-5-yl]benzonitrile 85

To a solution of 4-(tetrazol-5-yl)benzonitrile^44^ (170 mg, 0.99 mmol) and 3-(bromomethyl)phenol^49^ (205 mg, 1.10 mmol) in dry DMF (10 mL) was added potassium carbonate (474 mg, 3.43 mmol). The resulting suspension was heated at 80 °C for 4 h. After cooling, the mixture was diluted with water and exhaustively extracted with ethyl acetate. The combined organic layers were washed with brine, dried over sodium sulfate, and concentrated under reduced pressure. Chromatography on silica gel (cyclohexane/ethyl acetate 6 : 4) yielded 85 (183 mg, 66%) as a solid. C_15_H_11_N_5_O (277.3); mp 110–113 °C; ^1^H NMR (400 MHz, [D_6_]DMSO): *δ* (ppm) = 5.94 (s, 2H), 6.72–6.84 (m, 3H), 7.19 (t, *J* = 7.8 Hz, 1H), 8.00–8.05 (m, 2H), 8.18–8.25 (m, 2H); HRMS (APCI, direct probe) *m*/*z* [M + H]^+^ calc.: 278.1036, found: 278.1039.

#### *tert*-Butyl (*E*)-{2-[(3-{[5-(4-cyanophenyl)-2*H*-tetrazol-2-yl]methyl}phenoxy)methyl]-3-fluoroallyl}carbamate 86

Cesium carbonate (240 mg, 0.74 mmol) was added to a solution of 85 (104 mg, 0.38 mmol) and *tert*-butyl (*E*)-[2-(bromomethyl)-3-fluoroallyl]carbamate^50,51^ (78 mg, 0.29 mmol) in dry acetonitrile (10 mL). The mixture was stirred overnight at room temperature. The solvent was then removed under reduced pressure. To the residue water was added and the resulting mixture was extracted exhaustively with ethyl acetate. The combined organic layers were dried over sodium sulfate and concentrated under reduced pressure. Purification by chromatography on silica gel (gradient elution: cyclohexane to ethyl acetate) yielded 86 (65 mg, 48%) as an oil. C_24_H_25_FN_6_O_3_ (464.5); HRMS (ESI) *m*/*z* [M + H]^+^ calc.: 465.1941, found: 465.2074. The fluoroallyl-substituted carbamate used in this reaction was prepared from *tert*-butyl (2,3-dihydroxypropyl)carbamate before following published procedures.^50,51^ The mixture of *E* and Z isomers occurring at the *tert*-butyl [2-(hydroxymethyl)-3-fluoroallyl]carbamate stage were separated by chromatography on silica gel (gradient elution: cyclohexane to cyclohexane/ethyl acetate 7 : 3) and the isomers were each reacted separately to give the corresponding bromomethyl-substituted starting material.

#### (*E*)-4-[2-(3-{[2-(Aminomethyl)-3-fluoroallyl]oxy}benzyl)-2*H*-tetrazol-5-yl]benzonitrile 87

A solution of 86 (65 mg, 0.14 mmol) in ethyl acetate (1 mL) was treated with 4 M HCl in cyclopentyl methyl ether (6 mL). The mixture was stirred at room temperature for 2 h and at 50 °C for an additional 2 h. After completion, the solvents were removed under reduced pressure, and the residue was triturated with ethyl acetate (20 mL). The resulting precipitate was collected by filtration and washed with cold ethyl acetate (50 mL). The crude product was further purified by preparative HPLC (mobile phase: acetonitrile/water (40 : 60) containing 0.1% formic acid), affording 87 (10 mg, 20%) as a solid. C_19_H_17_FN_6_O (364.4); mp 196–198 °C; HPLC purity (method 1): 93% *E*, 5% *Z*; ^1^H NMR (600 MHz, [D_6_]DMSO): *δ* (ppm) = 3.57 (d, *J* = 1.9 Hz, 2H), 4.61 (d, *J* = 3.3 Hz, 2H), 6.01 (s, 2H), 7.01–7.06 (m, 3H), 7.22 (s, 0.5H), 7.35 (t, *J* = 7.9 Hz, 1.5H), 8.01–8.07 (m, 2H), 8.15 (s, 2H), 8.20–8.25 (m, 2H); ^13^C NMR (151 MHz, [D_6_]DMSO): *δ* (ppm) = 32.7, 56. 2, 64.0, 113.0, 113.1, 115.0, 118.3, 121.1, 127.1, 130.1, 130.9, 133.4, 135.3, 151.0, 152.8, 158.1, 163.1; HRMS (ESI) *m*/*z* [M + H]^+^ calc.: 365.1521, found: 365.1524.

#### *tert*-Butyl (*Z*)-{2-[(3-{[5-(4-cyanophenyl)-2*H*-tetrazol-2-yl]methyl}phenoxy)methyl]-3-fluoroallyl}carbamate 88

Compound 85 (119 mg, 0.43 mmol) was reacted with *tert*-butyl (*Z*)-[2-(bromomethyl)-3-fluoroallyl]carbamate^50,51^ (130 mg, 0.48 mmol) following the procedure described for the synthesis of 86, affording 88 (142 mg, 71%) as an oil. The fluoroallyl-substituted carbamate used in this synthesis was prepared before according to the procedure outlined for the synthesis of 86. C_24_H_25_FN_6_O_3_ (464.5); HRMS (ESI) *m*/*z* [M + H]^+^ calc.: 465.1941, found: 465.2045.

#### (*Z*)-4-[2-(3-{[2-(Aminomethyl)-3-fluoroallyl]oxy}benzyl)-2*H*-tetrazol-5-yl]benzonitrile 89

Compound 88 (135 mg, 0.29 mmol) was subjected to the same procedure as described for the preparation of compound 87, affording 89 as a solid (28 mg, 26%). C_19_H_17_FN_6_O (364.4); mp 158–161; HPLC purity (method 3): 100% *Z*; ^1^H NMR (600 MHz, [D_6_]DMSO): *δ* (ppm) = 3.47 (d, *J* = 3.3 Hz, 2H), 4.72 (d, *J* = 2.4 Hz, 2H), 6.01 (s, 2H), 6.99–7.06 (m, 3H), 7.16 (d, *J* = 82.5 Hz, 1H), 7.35 (t, *J* = 7.9 Hz, 1H), 8.01–8.06 (m, 2H), 8.20–8.25 (m, 2H); ^13^C NMR (151 MHz, [D_6_]DMSO): *δ* (ppm) = 36.3, 56.2, 60.1, 113.0, 113.8, 114.9, 118.3, 121.1, 127.1, 130.1, 130.9, 133.4, 135.3, 150.4, 152.2, 158.1, 163.1; HRMS (ESI) *m*/*z* [M + H]^+^ calc.: 365.1521, found: 365.1536.

#### 3-[(1*H*-Benzotriazol-1-yl)methyl]phenol 90

Potassium carbonate (552 mg, 3.99 mmol) was added to a solution of benzotriazole (150 mg, 1.26 mmol) and 3-(bromomethyl)phenol^49^ (360 mg, 1.93 mmol) in dry DMF (10 mL). The resulting suspension was stirred at room temperature overnight. Water (25 mL) was then added, and the mixture was exhaustively extracted with ethyl acetate. The combined organic layers were washed with brine, dried over sodium sulfate, and concentrated under reduced pressure. Purification by chromatography on silica gel (gradient elution: cyclohexane to cyclohexane/ethyl acetate 6 : 4) afforded 90 as a solid (184 mg, 65%). C_13_H_11_N_3_O (225.3); mp 150–152 °C; ^1^H NMR (400 MHz, CDCl_3_): *δ* (ppm) = 5.79 (s, 2H), 6.81–6.93 (m, 3H), 7.23 (t, *J* = 7.7 Hz, 1H), 7.30–7.45 (m, 3H), 7.97 (dt, *J* = 8.4 and 0.9 Hz, 1H); HRMS (APCI, direct probe) *m*/*z* [M + H]^+^ calc.: 226.0975, found: 226.0953.

#### *tert*-Butyl (*E*)-[2-({3-[(1*H*-benzotriazol-1-yl)methyl]phenoxy}methyl)-3-fluoroallyl]carbamate 91

Compound 90 (78 mg, 0.35 mmol) was treated with *tert*-butyl (*E*)-[2-(bromomethyl)-3-fluoroallyl]carbamate (92 mg, 0.29 mmol) under conditions analogous to those employed in the synthesis of 86. Purification by chromatography on silica gel (gradient elution: cyclohexane to cyclohexane/ethyl acetate 7 : 3) afforded 91 (59 mg, 42%) as a solid. C_22_H_25_N_4_O_3_ (412.5); ^1^H NMR (600 MHz, CDCl_3_): *δ* (ppm) = 1.39 (s, 9H), 3.95 (s, 2H), 4.36 (s, 2H), 4.72 (s, 1H), 5.81 (s, 2H), 6.66 (d, *J* = 82.8 Hz, 1H), 6.78–6.91 (m, 3H), 7.25 (t, *J* = 7.9 Hz, 1H), 7.33–7.44 (m, 3H), 8.07 (dt, *J* = 8.3 and 1.0 Hz, 1H).

#### (*E*)-2-({3-[(1*H*-Benzotriazol-1-yl)methyl]phenoxy}methyl)-3-fluoroprop-2-en-1-amine hydrochloride 92

A solution of 91 (59 mg, 0.14 mmol) in ethyl acetate (2 mL) was treated with 4 M HCl in cyclopentyl methyl ether (5 mL). The mixture was stirred at room temperature for 2 h and at 50 °C for an additional 2 h. After completion, the solvents were removed under reduced pressure, and the residue was triturated with ethyl acetate (20 mL) and cyclohexane (5 mL). The resulting precipitate was collected by filtration and washed with cold ethyl acetate (50 mL). Drying under reduced pressure yielded 92 (5 mg, 10%) as a solid. C_17_H_18_ClFN_4_O (348.8); HPLC purity (method 3): 76% *E*, 19% *Z*; ^1^H NMR (600 MHz, [D_6_]DMSO): *δ* (ppm) = 3.46–3.50 (s, 0.4H), 3.53–3.57 (s, 1.6H), 4.58 (s, 1.6H), 4.69 (s, 0.4H), 5.95 (s, 2H), 6.92–6.96 (m, 2H), 6.97–6.99 (m, 1H), 7.18 (d, *J* = 82.3 Hz, 0.2H), 7.27 (d, *J* = 82.0 Hz, 0.8H), 7.29 (t, *J* = 7.9 Hz, 1H), 7.40 (ddd, *J* = 8.2, 6.9 and 1.0 Hz, 1H), 7.54 (ddd, *J* = 8.2, 6.9 and 1.0 Hz, 1H), 7.86 (dt, *J* = 8.3 and 1.0 Hz, 1H), 8.05 (dt, *J* = 8.4 and 0.9 Hz, 1H), 8.27 (s, 3H); ^13^C NMR (151 MHz, [D_6_]DMSO): *δ* (ppm) = 32.5, 50.8, 63.9 (d, *J* = 10.9 Hz), 110.7, 112.9 (d, *J* = 4.9 Hz), 114.3, 114.5, 119.2, 120.5, 124.1, 127.5, 130.0, 132.7, 145.3, 151.1, 152.8, 158.1; HRMS (ESI) *m*/*z* [C_17_H_17_FN_4_O + H]^+^ calc.: 313.1459, found: 313.1450.

#### *tert*-Butyl (*Z*)-[2-({3-[(1*H*-benzotriazol-1-yl)methyl]phenoxy}methyl)-3-fluoroallyl]carbamate 93

Compound 90 (97 mg, 0.43 mmol) was treated with *tert*-butyl (*Z*)-[2-(bromomethyl)-3-fluoroallyl]carbamate (130 mg, 0.48 mmol) under conditions analogous to those employed in the synthesis of 86. Purification by chromatography on silica gel (gradient elution: cyclohexane to cyclohexane/ethyl acetate 7 : 3) afforded 93 (102 mg, 57%) as a solid. C_22_H_25_N_4_O_3_ (412.5); ^1^H NMR (600 MHz, CDCl_3_): *δ* (ppm) = 1.39 (s, 9H), 3.70 (s, 2H), 4.62 (s, 2H), 4.72 (s, 1H), 5.81 (s, 2H), 6.64 (d, *J* = 82.8 Hz, 1H), 6.79–6.90 (m, 3H), 7.25 (t, *J* = 7.9 Hz, 1H), 7.32–7.45 (m, 3H), 8.07 (dt, *J* = 8.3 and 1.0 Hz, 1H).

#### (*Z*)-2-({3-[(1*H*-Benzotriazol-1-yl)methyl]phenoxy}methyl)-3-fluoroprop-2-en-1-amine hydrochloride 94

Compound 93 (102 mg, 0.25 mmol) was subjected to the same procedure as described for the preparation of 92, affording 94 as a solid (55 mg, 64%). C_17_H_18_ClFN_4_O (348.8); HPLC purity (method 3): 89% *Z*, 8% *E*; ^1^H NMR (600 MHz, [D_6_]DMSO): *δ* (ppm) = 3.46–3.50 (s, 2H), 4.69 (s, 2H), 5.95 (s, 2H), 6.92–6.96 (m, 2H), 6.98–7.00 (m, 1H), 7.18 (d, *J* = 82.2 Hz, 1H), 7.28 (t, *J* = 7.9 Hz, 1H), 7.40 (ddd, *J* = 8.1, 6.9 and 1.0 Hz, 1H), 7.54 (ddd, *J* = 8.2, 6.9 and 0.8 Hz, 1H), 7.86 (dt, *J* = 8.4 and 1.0 Hz, 1H), 8.05 (dt, *J* = 8.4 and 1.0 Hz), 8.29 (s, 3H); ^13^C NMR (151 MHz, [D_6_]DMSO): *δ* (ppm) = 35.9 (d, *J* = 10.4 Hz), 50.8, 59.9 (d, *J* = 6.6 Hz), 110.7, 113.2, 114.2, 114.4, 119.2, 120.5, 124.1, 127.5, 130.0, 132.7, 145.3, 150.8, 152.6, 158.1; HRMS (ESI) *m*/*z* [C_17_H_17_FN_4_O + H]^+^ calc.: 313.1459, found: 313.1444.

### Biological evaluation

#### Inhibition of secretory vascular adhesion protein-1 (sVAP-1) isolated from bovine plasma

Determination of sVAP-1 inhibition with or without pre-incubation of enzyme and test compound was performed as recently described.^47,52^ 6-(5-Phenyl-2*H*-tetrazol-2-yl)hexan-1-amine was used as substrate and commercially purified sVAP-1 from bovine plasma as enzyme. The aldehyde formed as the product was derivatised with TRIS to give an oxazolidine derivative, which was quantified by HPLC and UV detection at 238 nm. Inhibition of sVAP-1 was calculated from the amount of oxazolidine formed in the absence and presence of a test compound (corrected for blank). The IC_50_ values were determined by Probit transformation.^64^

#### Degradation of test substances by sVAP-1 isolated from bovine plasma

Preparation of the enzyme solution: 0.035 I.U. (150 Tabor Units) (5.7 mg) of sVAP-1 from bovine plasma (Worthington, delivered by CellSystems, Troisdorf, Germany) were dissolved in phosphate buffered saline (PBS) (1000 μL) (PBS: prepared from phosphate buffered saline tablets (Sigma-Aldrich, Steinheim, Germany); one tablet dissolved in 200 mL of deionised water yields 0.01 M phosphate buffer, 0.0027 M potassium chloride and 0.137 M sodium chloride, pH 7.4, at 25 °C). The obtained solution was diluted with PBS by 1 : 25 (v/v).

Incubation procedure: To a mixture of DMSO (2.5 μL) and a solution of the test compound (0.40 mM) in DMSO (2.5 μL) was added a solution of bovine sVAP-1 in PBS (95 μL). After incubation at 37 °C for 15 min, 30 min, 60 min or 120 min, the enzyme activity was destroyed by the addition of acetonitrile (100 μL). The samples were cooled in an ice bath for 10 min and centrifuged at 12000 × *g* and 10 °C for 5 min. In parallel, controls were prepared by treating mixtures of the test compound solution (0.40 mM) in DMSO (2.5 μL), DMSO (2.5 μL) and PBS (95 μL) in the same way. The extent of degradation was evaluated by reversed-phase HPLC with MS-detection. The HPLC-MS system from Shimadzu (Kyoto, Japan) consisted of two LC-20ADXR HPLC-pumps, a SIL-30AC autosampler, and a LCMS-2020 single quad detector. Aliquots of 2 μL were injected onto a HICHROM ACE 3 C18 column (2.1 mm (I.D.) × 100 mm, particle size 3 μm) (HiChrom, Berkshire, UK) protected with a Phenomenex C18 guard column (3 mm (I.D.) × 4 mm) (Phenomenex, Aschaffenburg, Germany). Autosampler temperature was 10 °C, column oven temperature was set to 20 °C. The mobile phase consisted of acetonitrile/water/formic acid 10 : 90 : 0.1 (v/v/v) (A) and acetonitrile/water/formic acid 90 : 10 : 0.1 (v/v/v) (B). The gradient run from 10% to 95% of solvent B. The flow rate was 0.3 mL min^−1^. Detection was performed in ESI+ scan mode. The stability of a test compound was calculated by comparing its peak area in the chromatogram obtained in presence of sVAP-1 with its peak area in the chromatogram of the control.

#### Inhibition of VAP-1 activity in human whole plasma

Preparation of human plasma: Human blood was collected in S-Monovette® Lithium-Heparin LH/7.5 mL (Sarstedt, Nümbrecht, Germany) during a blood donation at Münster University Hospital. Each about 5 mL of blood were pipetted into 10 mL Falcon Tubes and subsequently centrifuged at 1500 × *g* at 20 °C for 15 min. Aliquots of 800 μL plasma were transferred into 2 mL Eppendorf tubes and stored at −80 °C until further use.

Incubation procedure: A solution of the appropriate inhibitor in DMSO (concentration variable) (1.25 μL) was treated with human whole plasma (47.5 μL) and pre-incubated at 37 °C for 15 min. Subsequently, the enzyme reaction was started by the addition of a solution of the substrate 6-(5-phenyl-2*H*-tetrazol-2-yl)hexan-1-amine^47^ (10 mM) in DMSO (1.25 μL). After 2 h of incubation, acetonitrile (50 μL) was added. The samples were cooled in an ice bath for 10 min and centrifuged at 12 000 × *g* and 10 °C for 10 min. An aliquot of the supernatant (75 μL) was diluted with aqueous TRIS buffer (100 mM, pH 8.5 at 20 °C) (75 μL), allowed to stand at room temperature for 30 min, and analysed by HPLC with UV detection at 238 nm as described recently.^47^ Controls with DMSO (1.25 μL) instead of a DMSO solution of the inhibitor (1.25 μL) were prepared in the same manner in parallel (*n* = 3). Blanks were run by incubating a mixture of the substrate solution in DMSO and DMSO (1.25 μL each) and 47.5 μL of PBS in the same way. Comparative chromatograms can be found in the SI of ref. 44. The peak ratios of enzyme product and internal standard obtained in the absence and presence of a test compound were compared, corrected for the blank value. From these data, the IC_50_ values were calculated *via* Probit-log concentration graphs.^64^

#### Inhibition of diamine oxidase (DAO)

Inhibition of DAO was studied as recently published.^52^ Briefly, the commercial enzyme isolated from porcine kidney was pre-incubated with the test compound for 15 min in potassium phosphate buffer. Subsequently, the enzymatic reaction was started by addition of the substrate 6-(5-phenyltetrazol-2-yl)hexan-1-amine. Determination of the produced aldehyde and calculation of the inhibition data were performed in the same manner as for the sVAP-1 assay described above.

#### Inhibition of monoamine oxidase A (MAO A)

Inhibition of MAO A was evaluated as recently described.^52^ Briefly, the commercial human recombinant enzyme was pre-incubated with the test compound for 15 min in PBS containing 0.2% of the detergent Brij35. Subsequently, the enzymatic reaction was started by addition of the substrate 4-(5-phenyl-2*H*-tetrazol-2-yl)butan-1-amine. Determination of the produced aldehyde and calculation of the inhibition data were performed in the same manner as for the sVAP-1 assay described above.

#### Inhibition of monoamine oxidase B (MAO B)

Inhibition of MAO B was determined as recently described.^52^ Briefly, the commercial human recombinant enzyme was pre-incubated with the test compound for 15 min in PBS containing 0.2% of the detergent Triton X-100. Subsequently, the enzymatic reaction was started by addition of the substrate 4-(5-phenyl-2*H*-tetrazol-2-yl)butan-1-amine. Determination of the produced aldehyde and calculation of the inhibition data were performed in the same manner as for the sVAP-1 assay described above.

#### Metabolic stability in rat liver S9 fraction

Metabolic stability was assessed using rat liver S9 fractions as previously described.^59,60^ In brief, test compounds (final concentration: 20 μM) were incubated with the liver preparation under aerobic conditions, both in the presence and absence of the cofactor NADPH (final concentration: 1 mM). After 30 min, the metabolic reactions were stopped, and the extent of compound metabolism was determined by reversed-phase HPLC with MS detection.^60^

#### Molecular modeling

Modeling of enzyme-ligand complexes was based on the crystal structure of the human VAP-1 (PDB entry 4BTW).^65^ Structural preparation was performed by using Molecular Operating Environment 2022.02 (MOE, Chemical Computing Group, Montreal, Canada). All docking experiments were performed with GOLD 5.2^66^ by using covalent settings and CHEMPLP as primary scoring function. Residues within a distance of 10 Å around residue 471 were defined as substrate binding site. Side chain flexibilities were individually assessed and iteratively evaluated during pose selection. The most plausible docking poses have been selected based on enzyme-ligand interactions and visualised in MOE.

## Conclusions

In summary, as part of these studies, we developed VAP-1 inhibitors featuring different head groups designed to form covalent bonds with either the enzyme or its cofactor, topaquinone. Initial testing was conducted with amine oxidase sVAP-1 (AOC4) derived from bovine plasma, which is structurally closely related to VAP-1 (AOC3).

Structure–activity studies previously performed with glycine amides bearing terminal phenyl tetrazole substituents^44^ were extended to include related compounds containing benzannelated azole heterocycles. These analogues were also found to act as enzyme substrates: after covalent binding to the cofactor, they were cleaved to form glyoxamides, classifying them as substrate inhibitors. By contrast, compounds containing semicarbazide or fluoroallylamine groups functioned as covalent inhibitors that were not further processed by the enzyme.

Selected compounds were also tested for their ability to inhibit VAP-1 activity in human plasma. They demonstrated inhibitory activity, although the values partly differed from those measured with the bovine sVAP-1 enzyme. The most pronounced differences were observed with glycine amides containing an aliphatic spacer between the glycine amide and the heterocycle, as well as with semicarbazides: these compounds inhibited the bovine enzyme approximately 8- to 20-fold more strongly than the human enzyme. In contrast, fluoroallylamines were comparably or even more effective against the human enzyme.

In selectivity experiments with other amine oxidases, glycine amides were found to be weak inhibitors of diamine oxidase (DAO), whereas semicarbazides acted as strong inhibitors of this enzyme. Monoamine oxidases A and B (MAO A and MAO B), by contrast, were not inhibited by either substance group. Fluoroallylamines inhibited DAO with much lower potency than human VAP-1 and showed only marginal inhibition of MAO A. Inhibition of MAO B, however, was comparable in strength to that observed for VAP-1.

The literature indicates that dual inhibitors of VAP-1 and MAO-B, such as mofegiline and PXS-5131, may have beneficial effects in the treatment of inflammation.^40,41^ In this context, the benzotriazole-substituted fluoroallylamine 94 emerges as a particularly promising candidate for further evaluation. Unlike mofegiline and PXS-5131,^40^ compound 94 displays more closely aligned IC_50_ values for VAP-1 and MAO B, making it a well-balanced potent dual inhibitor of these two enzymes.

## Author contributions

Conceptualization: TP, JK, ML. Investigation: TP, JK, WH. Methodology: TK, JK, WH, MB, ML. Project administration: MB, ML. Resources: MB, ML. Supervision: MB, ML. Validation: TP, JK, MB, ML. Visualization: TP, JK, MB, ML. Writing – original draft: ML.

## Conflicts of interest

There are no conflicts to declare.

## Supplementary Material

MD-OLF-D5MD01008J-s001

## Data Availability

The data supporting this article have been included as part of the supplementary information (SI). Supplementary information: it comprises synthetic procedures not described in the Experimental section as well as ^1^H NMR, ^13^C NMR and HRMS spectra of all compounds tested. See DOI: https://doi.org/10.1039/d5md01008j.
