# Supplementary material for: Novel glycine amides, semicarbazides and fluoroallylamines as inhibitors of the amine oxidase vascular adhesion protein-1 (VAP-1)
Source: RSC Med Chem. 2026 Jan 28;17(3):1709–28. doi: 10.1039/d5md01008j (PMC12914920; doi:10.1039/d5md01008j)
Supplement: MD-017-D5MD01008J-s001 [file MD-017-D5MD01008J-s001.pdf]

Supplementary Information (SI) for RSC Medicinal Chemistry.
This journal is © The Royal Society of Chemistry 2026

Supplementary Information

Novel glycine amides, semicarbazides and fluoroallylamines as inhibitors of the amine oxidase vascular adhesion protein-1 (VAP-1)

*Timo Pöstges, Jan Kampschulze, Walburga Hanekamp, Marcel Bermúdez, and Matthias Lehr**

Institute of Pharmaceutical and Medicinal Chemistry, University of Münster, Corrensstrasse 48, D-48149 Münster, Germany

* Corresponding author. Tel.: +49251 83 33331; fax: +49251 83 32144;

E-mail address: lehrm@uni-muenster.de

Supplementary Information content

		Page
1.	Synthetic procedures	2
2.	References	22
3.	^{1}H NMR, ^{13}C NMR and HRMS spectra of the target compounds	23
4.	Means and standard deviations of the inhibition data listed in Table 4	77

1. Synthetic procedures

2-[6-(1*H*-Indazol-1-yl)hexyl]isoindoline-1,3-dione (15), 2-[6-(2*H*-Indazol-2-yl)hexyl]isoindoline-1,3-dione (16)

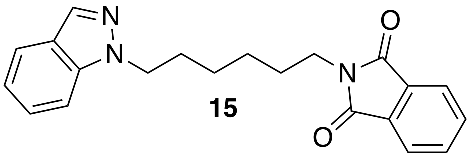

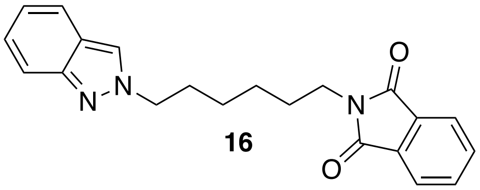

Sodium hydride (60 %, dispersion in mineral oil, 166 mg, 4.15 mmol) was added to a solution of indazole (411 mg, 3.48 mmol) in dry DMF (10 mL) with stirring. The mixture was then heated to 60 °C. After 30 min, 2-(6-bromohexyl)isoindoline-1,3-dione (1.16 g, 3.74 mmol) was added and the mixture was further stirred at 60 °C for 4 h. After cooling to room temperature, the reaction mixture was diluted with water and exhaustively extracted with ethyl acetate. The combined organic phases were washed with water, dried over anhydrous sodium sulfate, and concentrated under reduced pressure. Chromatography on silica gel (gradient elution: cyclohexane to cyclohexane/ethyl acetate 6:4) afforded **15** (321 mg, 27%) as oil and **16** (247 mg, 20%) as solid. **15**: $C_{21}H_{21}N_3O_2$ (347.4); ^{1}H NMR (600 MHz, $CDCl_3$): δ (ppm) = 1.32 – 1.45 (m, 4H), 1.60 – 1.69 (m, 2H), 1.89 – 1.97 (m, 2H), 3.63 – 3.67 (m, 2H), 4.36 – 4.40 (m, 2H), 7.13 (td, *J* = 7.4 and 3.3 Hz, 1H), 7.34 – 7.42 (m, 2H), 7.67 – 7.74 (m, 3H), 7.80 – 7.85 (m, 2H), 7.98 (d, *J* = 3.3 Hz, 1H); HRMS (APCI, direct probe) *m/z* $[M+H]^+$ calc.: 348.1707, found: 348.1674. **16**: $C_{21}H_{21}N_3O_2$ (347.4); mp 117 – 119 °C; ^{1}H NMR (600 MHz, $CDCl_3$): δ (ppm) = 1.35 – 1.42 (m, 4H), 1.64 – 1.69 (m, 2H), 2.00 – 2.06 (m, 2H), 3.66 (t, *J* = 7.2 Hz, 2H), 4.42 (t, *J* = 7.2 Hz, 2H), 7.08 (ddd, *J* = 8.5, 6.6 and 1.0 Hz, 1H), 7.28 (ddd, *J* = 8.7, 6.6 and 1.2 Hz, 1H), 7.64 (dt, *J* = 8.4 and 1.0 Hz, 1H), 7.68 – 7.72 (m, 3H), 7.81 – 7.85 (m, 2H), 7.91 (s, 1H); HRMS (APCI, direct probe) *m/z* $[M+H]^+$ calc.: 348.1707, found: 348.1702.

6-(1*H*-Indazol-1-yl)hexan-1-amine (17)

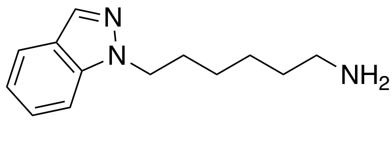

A solution of **15** (301 mg, 0.87 mmol) in ethanol (50 mL) was treated with hydrazine monohydrate (261 mg, 5.21 mmol) under stirring, and the mixture was heated under reflux for 3.5 h. The solvent was then removed under reduced pressure, and the resulting residue was treated with aqueous sodium hydroxide solution (50 mL, pH > 10). The aqueous layer was exhaustively extracted with ethyl acetate. The combined organic phases were washed with aqueous sodium hydroxide solution, dried over anhydrous sodium sulfate, and concentrated under reduced pressure to yield **17** (174 mg, 92%) as an oil. $C_{13}H_{19}N_3$ (217.3); ^{1}H NMR (400 MHz, $CDCl_3$): δ (ppm) = 1.27 – 1.49 (m, 6H), 1.90 – 1.97 (m, 2H), 2.64 – 2.69 (m, 2H), 4.38

(t, *J* = 7.1 Hz, 2H), 7.13 (ddd, *J* = 7.9, 6.4 and 1.3 Hz, 1H), 7.34 – 7.42 (m, 2H), 7.73 (dt, *J* = 8.1 and 1.0 Hz, 1H), 7.98 (d, *J* = 0.9 Hz, 1H); HRMS (APCI, direct probe) *m/z* $[M+H]^+$ calc.: 218.1652, found: 218.1659.

tert-Butyl (2-{[6-(1*H*-indazol-1-yl)hexyl]amino}-2-oxoethyl)carbamate (18)

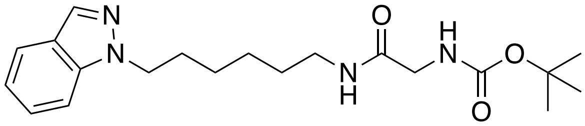

A solution of **17** (164 mg, 0.75 mmol), *N*-(*tert*-butoxycarbonyl)glycine (145 mg, 0.83 mmol) and 1-hydroxybenzotriazole (88 % (m/m), 127 mg, 0.83 mmol) in dry DMF (6 mL) was treated with *N*-(3-dimethylaminopropyl)-*N'*-ethylcarbodiimide hydrochloride (EDC-HCl) (159 mg, 0.83 mmol) and stirred at room temperature for 16 h. After completion, semisaturated aqueous sodium bicarbonate solution was added, and the reaction mixture was exhaustively extracted with ethyl acetate. The combined organic phases were washed twice with saturated sodium bicarbonate solution, dried over anhydrous sodium sulfate, and concentrated under reduced pressure. The residue was purified by chromatography on silica gel (gradient elution: cyclohexane/ethyl acetate 1:1 to cyclohexane/ethyl acetate 2:8) to afford **18** (181 mg, 64%) as an oil. $C_{20}H_{30}N_4O_3$ (374.5); ^{1}H NMR (400 MHz, $CDCl_3$): δ (ppm) = 1.25 – 1.40 (m, 4H), 1.42 – 1.51 (m, 11H), 1.92 (p, *J* = 7.0 Hz, 2H), 3.22 (q, *J* = 6.7 Hz, 2H), 3.76 (s, 2H), 4.40 (t, *J* = 7.0 Hz, 2H), 5.19 (s, 1H), 6.23 (s, 1H), 7.16 (ddd, *J* = 8.2, 5.5 and 2.2 Hz, 1H), 7.39 – 7.41 (m, 2H), 7.74 (dt, *J* = 8.2 and 1.0 Hz, 1H), 8.01 (d, *J* = 0.7 Hz, 1H); HRMS (APCI, direct probe) *m/z* $[M+H]^+$ calc.: 375.2391, found: 375.2410.

***N*-[6-(1*H*-Indazol-1-yl)hexyl]-2-aminoacetamide dihydrochloride (19)**

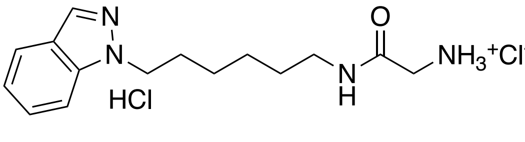

A solution of compound **18** (165 mg, 0.44 mmol) in ethyl acetate (3 mL) was treated with 4 M HCl in cyclopentyl methyl ether (3 mL) and stirred at room temperature for 4 h. The solvent was then removed under reduced pressure, and the resulting residue was dispersed in ethyl acetate. After filtration, the filter cake was dried under reduced pressure to give **19** as a solid (100 mg, 65%). $C_{15}H_{24}Cl_2N_4O$ (347.3); HPLC purity (method 1): > 99 %; ^{1}H NMR (600 MHz, [D$_6$]DMSO): δ (ppm) = 1.19 – 1.25 (m, 2H), 1.26 – 1.32 (m, 2H), 1.34 – 1.39 (m, 2H), 1.81 (p, *J* = 7.1 Hz, 2H), 3.06 (td, *J* = 6.9 and 5.5 Hz, 2H), 3.48 (q, *J* = 5.7 Hz, 2H), 4.39 (t, *J* = 6.9 Hz, 2H), 7.12 (ddd, *J* = 7.9, 6.8 and 0.9 Hz, 1H), 7.35 – 7.39 (m, 1H), 7.65 – 7.67 (m, 1H), 7.75 (dt, *J* = 8.1 and 1.0 Hz, 1H), 8.04 (d, *J* = 1.0 Hz, 1H), 8.14 (s, 3H), 8.42 (t, *J* = 5.6 Hz, 1H,); ^{13}C NMR (151 MHz, [D$_6$]DMSO): δ (ppm) = 25.9, 28.7, 29.4, 38.6, 40.0, 47.9, 109.6, 120.2,

120.8, 123.4, 125.9, 132.4, 139.2, 165.6; HRMS (APCI, direct probe) *m/z* $[C_{15}H_{22}N_4O+H]^+$ calc.: 275.1866, found: 275.1873.

6-(2*H*-Indazol-2-yl)hexan-1-amine (20)

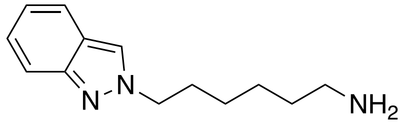

Compound **16** (165 mg, 0.47 mmol) was reacted with hydrazine monohydrate (141 mg, 2.82 mmol) following the same procedure described for the synthesis of compound **17**, affording **20** (72 mg, 70%) as an oil. $C_{13}H_{19}N_3$ (217.3); ^{1}H NMR (400 MHz, $CDCl_3$): δ (ppm) = 1.29 – 1.50 (m, 6H), 2.02 (p, *J* = 7.1 Hz, 2H), 2.69 (t, *J* = 6.8 Hz, 2H), 4.40 (t, *J* = 7.1 Hz, 2H), 7.07 (ddd, *J* = 8.4, 6.6 and 0.9 Hz, 1H), 7.27 (ddd, *J* = 8.8, 6.7 and 1.0 Hz, 1H), 7.64 (dt, *J* = 8.4, 1.1 Hz, 1H), 7.69 – 7.72 (m, 1H), 7.90 (d, *J* = 1.0 Hz, 1H); HRMS (APCI, direct probe) *m/z* $[M+H]^+$ calc.: 218.1652, found: 218.1679.

***tert*-Butyl (2-{[6-(2*H*-indazol-2-yl)hexyl]amino}-2-oxoethyl)carbamate (21)**

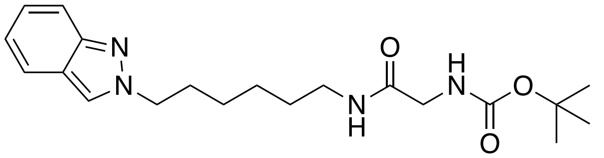

Compound **20** (64 mg, 0.29 mmol) was treated with *N*-(*tert*-butoxycarbonyl)glycine (56 mg, 0.32 mmol) under conditions analogous to those employed in the synthesis of **18**. Purification by chromatography on silica gel (gradient elution: cyclohexane/ethyl acetate 4:6 to ethyl acetate) afforded **21** (69 mg, 63%) as an oil. $C_{20}H_{30}N_4O_3$ (374.5); ^{1}H NMR (400 MHz, $CDCl_3$): δ (ppm) = 1.31 – 1.42 (m, 4H), 1.44 (s, 9H) 1.50 (p, *J* = 7.2 Hz, 2H), 2.06 (p, *J* = 7.1 Hz, 2H), 3.25 (q, *J* = 6.6 Hz, 2H), 3.77 (d, *J* = 5.7 Hz, 2H), 4.48 – 4.54 (m, 2H), 5.17 (s, 1H), 6.23 (s, 1H), 7.13 – 7.19 (m, 1H), 7.34 – 7.41 (m, 1H), 7.67 – 7.71 (m, 1H), 7.73 – 7.77 (m, 1H), 7.99 (d, *J* = 3.4 Hz, 1H); HRMS (APCI, direct probe) *m/z* $[M+H]^+$ calc.: 375.2391, found: 375.2422.

***N*-[6-(2*H*-Indazol-2-yl)hexyl]-2-aminoacetamide dihydrochloride (22)**

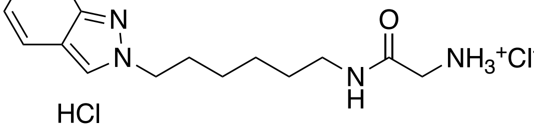

A solution of **21** (59 mg, 0.16 mmol) in a mixture of ethyl acetate and 4 M HCl in cyclopentyl methyl ether (1:1, 4 mL) was stirred at room temperature for 4 h. After removal of the solvent, the oily residue was dispersed in a small amount ethyl acetate and treated in an ultrasonic bath for 5 min before the solvent was removed again. This process was repeated two more times.

The almost solid residue was then dispersed in ethyl acetate and filtered. After vacuum drying of the filter residue, **22** (13 mg, 24%) was obtained as a solid. $C_{15}H_{24}Cl_2N_4O$ (347.3); HPLC purity (method 1): > 99 %; ^{1}H NMR (600 MHz, [D$_6$]DMSO): δ (ppm) = 1.20 – 1.26 (m, 2H), 1.28 – 1.34 (m, 2H), 1.37 – 1.42 (m, 2H), 1.91 (p, *J* = 7.1 Hz, 2H), 3.08 (q, *J* = 6.6 Hz, 2H), 3.49 (q, *J* = 5.8 Hz, 2H), 4.41 (t, *J* = 7.0 Hz, 2H), 7.02 (ddd, *J* = 8.3, 6.6 and 0.9 Hz, 1H), 7.22 (ddd, *J* = 8.7, 6.6 and 1.1 Hz, 1H), 7.57 – 7.60 (m, 1H), 7.69 (dt, *J* = 8.4 and 1.2 Hz, 1H), 8.07 (s, 3H), 8.35 – 8.38 (m, 2H); ^{13}C NMR (151 MHz, [D$_6$]DMSO): δ (ppm) = 25.66, 25.77, 28.7, 29.9, 38.5, 40.1, 52.6, 116.9, 120.5, 120.8, 121.3, 123.7, 125.2, 147.9, 165.6; HRMS (APCI, direct probe) *m/z* $[C_{15}H_{22}N_4O+H]^+$ calc.: 275.1866, found: 275.1870; Elemental analysis calcd. for $C_{15}H_{24}Cl_2N_4O$: C 51.88, H 6.97, N 16.13, found: C 53.42, H 7.24, N 15.58.

1-[6-(1,3-Dioxoisoindolin-2-yl)hexyl]-1*H*-benzotriazole-5-carbonitrile (35), 1-[6-(1,3-dioxoisoindolin-2-yl)hexyl]-1*H*-benzotriazole-6-carbonitrile (36)

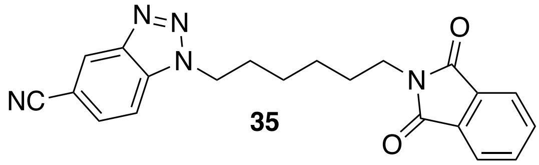

35

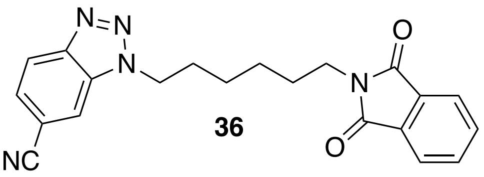

36

A solution of benzotriazole-5-carbonitrile (504 mg, 3.50 mmol) in acetonitrile (50 mL) was treated with 2-(6-bromohexyl)isoindoline-1,3-dione (1.08 g, 3.48 mmol) and potassium carbonate (967 mg, 7.00 mmol) and heated under reflux for 4 h. After cooling to room temperature, the mixture was filtered and the filtrate concentrated under reduced pressure. Chromatography on silica gel (cyclohexane/ethyl acetate 7:3) yielded the regio isomers **35** (285 mg, 22%) and **36** (185 mg, 14%) as solids. **35**: $C_{21}H_{19}N_5O_2$ (373.4); mp 142 – 144 °C; ^{1}H NMR (600 MHz, [D$_6$]DMSO): δ (ppm) = 1.24 – 1.31 (m, 4H), 1.54 (p, *J* = 7.0 Hz, 2H), 1.90 (p, *J* = 7.0 Hz, 2H), 3.52 (t, *J* = 7.1 Hz, 2H), 4.75 (t, *J* = 7.0 Hz, 2H), 7.79 – 7.86 (m, 4H), 7.90 (dd, *J* = 8.6 and 1.4 Hz, 1H), 8.12 (dd, *J* = 8.7 and 0.9 Hz, 1H), 8.75 (t, *J* = 1.1 Hz, 1H,); HRMS (APCI, direct probe) *m/z* $[M+H]^+$ calc.: 374.1612, found: 374.1630. **367**: $C_{21}H_{19}N_5O_2$ (373.4); mp 153 – 155 °C; ^{1}H NMR (600 MHz, [D$_6$]DMSO): δ (ppm) = 1.25 – 1.33 (m, 4H), 1.54 (p, *J* = 7.1 Hz, 1H), 1.91 (p, *J* = 6.9 Hz, 2H), 3.52 (t, *J* = 7.1 Hz, 2H), 4.75 (t, *J* = 7.1 Hz, 2H), 7.75 (dd, *J* = 8.6 and 1.4 Hz, 1H), 7.80 – 7.86 (m, 4H), 8.24 (dd, *J* = 8.6 and 0.9 Hz, 1H,), 8.69 (t, *J* = 1.1 Hz, 1H); HRMS (APCI, direct probe) *m/z* $[M+H]^+$ calc.: 374.1612, found: 374.1607.

1-(6-Aminohexyl)-1*H*-benzotriazole-5-carbonitrile (37)

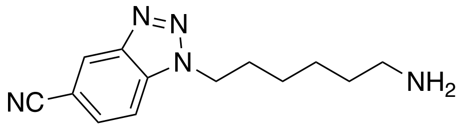

Compound **35** (270 mg, 0.72 mmol) was reacted with hydrazine monohydrate (216 mg, 4.31 mmol) following the same procedure described for the synthesis of compound **17**, affording **37** (169 mg, 96%) as a solid. $C_{13}H_{17}N_5$ (243.3); mp 46 – 48 °C; ^{1}H NMR (400 MHz, [D$_6$]DMSO): δ (ppm) = 1.18 – 1.33 (m, 6H), 1.91 (p, *J* = 7.1 Hz, 2H), 2.46 (t, *J* = 6.4 Hz, 2H), 4.77 (t, *J* = 7.0 Hz, 2H), 7.92 (dd, *J* = 8.6 and 1.4 Hz, 1H), 8.14 (dd, *J* = 8.7 and 0.9 Hz, 1H), 8.77 (dd, *J* = 1.4 and 0.8 Hz, 1H); HRMS (APCI, direct probe) *m/z* $[M+H]^+$ calc.: 244.1557, found: 244.1578.

***tert*-Butyl (2-{[6-(5-cyano-1*H*-benzotriazol-1-yl)hexyl]amino}-2-oxoethyl)carbamate (38)**

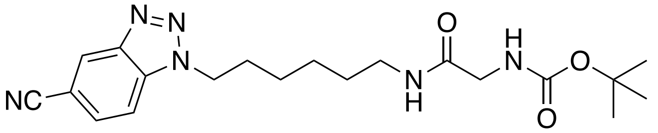

Compound **37** (161 mg, 0.66 mmol) was treated with *N*-(*tert*-butoxycarbonyl)glycine (128 mg, 0.73 mmol) under conditions analogous to those employed in the synthesis of **18**. Purification by chromatography on silica gel (gradient elution: cyclohexane/ethyl acetate 4:6 to ethyl acetate) afforded **38** (226 mg, 85%) as an oil. $C_{20}H_{28}N_6O_3$ (400.5); ^{1}H NMR (400 MHz, [D$_6$]DMSO): δ (ppm) = 1.21 – 1.38 (m, 15H), 1.87 – 1.94 (m, 2H), 3.00 (q, *J* = 6.4 Hz, 2H), 3.46 (d, *J* = 6.1 Hz, 2H), 4.76 (t, *J* = 7.0 Hz, 2H), 6.85 (t, *J* = 6.1 Hz, 1H), 7.66 (t, *J* = 5.7 Hz, 1H), 7.92 (dd, *J* = 8.6 and 1.4 Hz, 1H), 8.14 (dd, *J* = 8.7 and 0.8 Hz, 1H), 8.77 (dd, *J* = 1.4 and 0.8 Hz, 1H); HRMS (APCI, direct probe) *m/z* $[M+H]^+$ calc.: 401.2296, found: 401.2315.

2-Amino-*N*-[6-(5-cyano-1*H*-benzotriazol-1-yl)hexyl]acetamide hydrochloride (39)

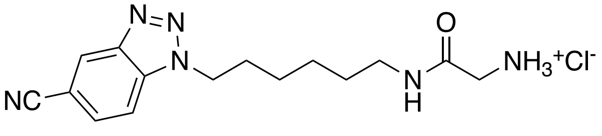

A solution of **38** (98 mg, 0.24 mmol) in ethyl acetate (2 mL) was treated with 4 M HCl in cyclopentyl methyl ether (3 mL) and stirred at room temperature for 4 h. The solvent was then removed under reduced pressure, and the resulting residue was dispersed in ethyl acetate. After filtration, the filter cake was dried under reduced pressure to give **39** as a solid (54 mg, 66%). $C_{15}H_{21}ClN_6O$ (336.8); HPLC purity (method 2): 99%; ^{1}H NMR (600 MHz, [D$_6$]DMSO): δ (ppm) = 1.20 – 1.27 (m, 2H), 1.28 – 1.34 (m, 2H), 1.35 – 1.42 (m, 2H), 1.91 (p, *J* = 7.2 Hz, 2H), 3.07 (td, *J* = 6.8 and 5.4 Hz, 2H), 3.49 (s, 2H), 4.78 (t, *J* = 7.0 Hz, 2H), 7.93 (dd, *J* = 8.6

and 1.4 Hz, 1H), 8.10 – 8.19 (m, 4H), 8.44 (t, *J* = 5.6 Hz, 1H), 8.78 (dd, *J* = 1.4 and 0.8 Hz, 1H); ^{13}C NMR (151 MHz, [D$_6$]DMSO): δ (ppm) = 25.61, 25.65, 28.6, 29.1, 38.5, 40.0, 47.8, 106.6, 112.6, 118.8, 125.9, 129.4, 134.6, 144.2, 165.6; HRMS (APCI, direct probe) *m/z* [$C_{15}H_{20}N_6O$+H]$^+$ calc.: 301.1771, found: 301.1796. Elemental analysis calcd. for $C_{15}H_{21}ClN_6O$: C 53.49, H 6.28, N 24.95, found: C 52.88, H 6.30, N 24.84.

1-(6-Aminohexyl)-1*H*-benzotriazole-6-carbonitrile (40)

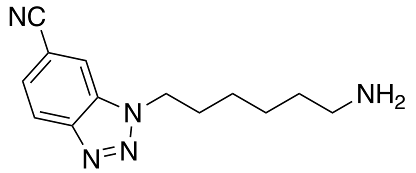

Compound **36** (172 mg, 0.46 mmol) was reacted with hydrazine monohydrate (138 mg, 2.76 mmol) following the same procedure described for the synthesis of **17**, affording **40** (103 mg, 92%) as a solid. $C_{13}H_{17}N_5$ (243.3); mp 76 – 78 °C; ^{1}H NMR (400 MHz, [D$_6$]DMSO): δ (ppm) = 1.21 – 1.32 (m, 6H), 1.93 (p, *J* = 7.2 Hz, 2H), 2.46 (t, *J* = 6.4 Hz, 2H), 4.77 (t, *J* = 7.1 Hz, 2H), 7.76 (dd, *J* = 8.6 and 1.4 Hz, 1H), 8.26 (dd, *J* = 8.6 and 0.9 Hz, 1H), 8.71 (dd, *J* = 1.4 and 0.9 Hz, 1H); HRMS (APCI, direct probe) *m/z* [M+H]$^+$ calc.: 244.1557, found: 244.1569.

***tert*-Butyl (2-{[6-(6-cyano-1*H*-benzotriazol-1-yl)hexyl]amino}-2-oxoethyl)carbamate (41)**

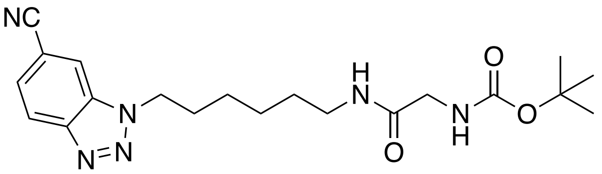

Compound **40** (93 mg, 0.38 mmol) was treated with *N*-(*tert*-butoxycarbonyl)glycine (74 mg, 0.42 mmol) under conditions analogous to those employed in the synthesis of **18**. Purification by chromatography on silica gel (gradient elution: cyclohexane/ethyl acetate 1:1 to 1:9) afforded **41** (109 mg, 71%) as an oil. $C_{20}H_{28}N_6O_3$ (400.5); ^{1}H NMR (400 MHz, [D$_6$]DMSO): δ (ppm) = 1.20 – 1.41 (s, 15H), 1.92 (p, *J* = 7.0 Hz, 2H), 3.01 (q, *J* = 6.4 Hz, 2H), 3.46 (d, *J* = 6.0 Hz, 2H), 4.77 (t, *J* = 7.1 Hz, 2H), 6.86 (t, *J* = 6.1 Hz, 1H), 7.66 (t, *J* = 5.6 Hz, 1H), 7.76 (dd, *J* = 8.6 and 1.4 Hz, 1H), 8.26 (dd, *J* = 8.6 and 0.9 Hz, 1H), 8.71 (dd, *J* = 1.4 and 0.9 Hz, 1H); HRMS (APCI, direct probe) *m/z* [M+H]$^+$ calc.: 401.2296, found: 401.2316.

2-Amino-*N*-[6-(6-cyano-1*H*-benzotriazol-1-yl)hexyl]acetamide hydrochloride (42)

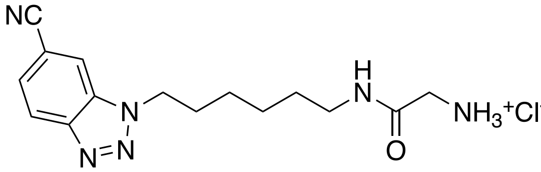

Compound **41** (103 mg, 0.26 mmol) was treated under the same reaction conditions used for the synthesis of **39**, affording **42** (65 mg, 75%) as a solid. $C_{15}H_{21}ClN_6O$ (336.8); HPLC purity (method 2): > 99%; ^{1}H NMR (600 MHz, [D$_6$]DMSO): δ (ppm) = 1.22 – 1.35 (m, 4H), 1.36 – 1.43 (m, 2H), 1.92 (p, *J* = 7.2 Hz, 2H), 3.07 (q, *J* = 6.7 Hz, 2H), 3.49 (s, 2H), 4.78 (t, *J* = 7.1 Hz, 2H), 7.77 (dd, *J* = 8.6 and 1.4 Hz, 1H), 8.13 (s, 3H), 8.26 (dd, *J* = 8.6 and 0.9 Hz, 1H), 8.42 (t, *J* = 5.6 Hz, 1H), 8.74 (t, *J* = 1.2 Hz, 1H); ^{13}C NMR (151 MHz, [D$_6$]DMSO): δ (ppm) = 25.63, 25.66, 28.7, 29.1, 38.5, 40.0, 48.0, 109.4, 117.6, 118.8, 120.8, 126.3, 132.3, 146.3, 165.6; HRMS (APCI, direct probe) *m/z* $[C_{15}H_{20}N_6O+H]^+$ calc.: 301.1771, found: 301.1796.

2-[4-(1*H*-Benzotriazol-1-yl)butyl]isoindoline-1,3-dione (43)

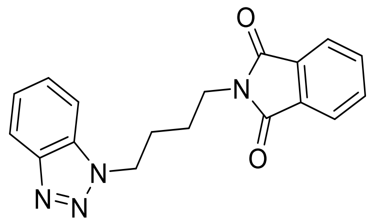

A mixture of benzotriazole (298 mg, 2.50 mmol), 2-(4-bromobutyl)isoindoline-1,3-dione (705 mg, 2.50 mmol), potassium carbonate (691 mg, 5.00 mmol) and acetonitrile (30 mL) was heated under reflux for 15 h. After cooling to room temperature, the mixture was filtered and the filtrate concentrated under reduced pressure. Chromatography on silica gel (cyclohexane/ethyl acetate 7:3) afforded **43** (342 mg, 43%) as a solid. $C_{18}H_{16}N_4O_2$ (320.4); mp 119 – 121 °C; ^{1}H NMR (400 MHz, $CDCl_3$): δ (ppm) = 1.72 – 1.80 (m, 2H), 2.03 – 2.11 (m, 2H), 3.75 (t, *J* = 6.9 Hz, 2H), 4.70 (t, *J* = 7.1 Hz, 2H), 7.36 (ddd, *J* = 8.4, 6. and 1.1 Hz, 1H), 7.48 (ddd, *J* = 8.3, 6.8 and 1.0 Hz, 1H), 7.56 (dt, *J* = 8.3 and 1.0 Hz, 1H), 7.68 – 7.74 (m, 2H), 7.80 – 7.85 (m, 2H), 8.05 (dt, *J* = 8.4 and 1.0 Hz, 1H); HRMS (APCI, direct probe) *m/z* $[M+H]^+$ calc.: 321.1346, found: 321.1334.

4-(1*H*-Benzotriazol-1-yl)butan-1-amine (44)

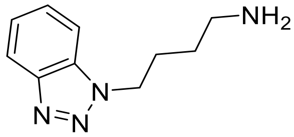

Compound **43** (312 mg, 0.97 mmol) was reacted with hydrazine monohydrate (291 mg, 5.81 mmol) following a procedure analogous to that used for the synthesis of compound **17**,

affording **44** (108 mg, 58%) as an oil. $C_{10}H_{14}N_4$ (190.3); ^{1}H NMR (400 MHz, $CDCl_3$): δ (ppm) = 1.47 – 1.58 (m, 2H), 2.03 – 2.12 (m, 2H), 2.71 – 2.80 (m, 2H), 4.67 (t, *J* = 7.0 Hz, 2H), 7.34 – 7.38 (m, 1H), 7.46 – 7.55 (m, 2H), 8.05 (dt, *J* = 8.4 and 1.0 Hz, 1H); HRMS (APCI, direct probe) *m/z* $[M+H]^+$ calc.: 191.1291, found: 191.1268.

tert-Butyl (2-{[4-(1*H*-benzotriazol-1-yl)butyl]amino}-2-oxoethyl)carbamate (45)

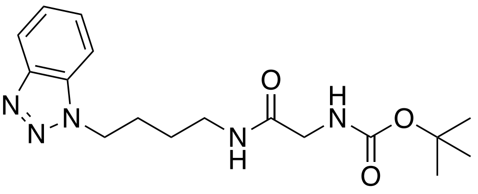

Compound **44** (101 mg, 0.53 mmol) was treated with *N*-(*tert*-butoxycarbonyl)glycine (102 mg, 0.58 mmol) under conditions analogous to those employed in the synthesis of **18**. Purification by chromatography on silica gel (ethyl acetate) afforded **45** (138 mg, 75%) as an oil. $C_{17}H_{25}N_5O_3$ (347.4); ^{1}H NMR (400 MHz, $[D_6]CDCl_3$): δ (ppm) = 1.42 (s, 9H), 1.52 – 1.60 (m, 2H), 2.02 – 2.10 (m, 2H), 3.32 (q, *J* = 6.7 Hz, 2H), 3.74 (s, 2H), 4.68 (t, *J* = 7.0, Hz, 2H), 5.10 (s, 1H), 6.24 (s, 1H), 7.39 (ddd, *J* = 8.2, 6.8 and 2.2 Hz, 1H), 7.48 – 7.58 (m, 2H), 8.04 – 8.09 (m, 1H); HRMS (APCI, direct probe) *m/z* $[M+H]^+$ calc.: 348.2030, found: 348.2011.

***N*-[4-(1*H*-Benzotriazol-1-yl)butyl]-2-aminoacetamide dihydrochloride (46)**

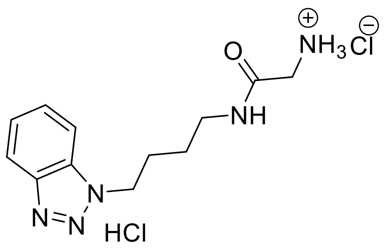

Compound **45** (128 mg, 0.37 mmol) was subjected to reaction conditions analogous to those used for the synthesis of **39**, yielding **46** as a solid (66 mg, 56%). $C_{12}H_{19}Cl_2N_5O$ (320.2); HPLC purity (method 2): > 99%; ^{1}H NMR (600 MHz, $[D_6]DMSO$): δ (ppm) = 1.38 – 1.43 (m, 2H), 1.91 – 1.96 (m, 2H), 3.14 (td, *J* = 6.9 and 5.5 Hz, 2H), 3.48 (q, *J* = 5.9 Hz, 2H), 4.73 (t, *J* = 7.0 Hz, 2H), 7.40 (ddd, *J* = 8.1, 7.1 and 1.0 Hz, 1H), 7.55 (ddd, *J* = 8.2, 6.9 and 1.0 Hz, 1H), 7.92 (dt, *J* = 8.4 and 1.0 Hz, 1H), 8.03 (dt, *J* = 8.3 and 1.0 Hz, 1H), 8.16 (s, 3H), 8.53 (t, *J* = 5.6 Hz, 1H; ^{13}C NMR (151 MHz, $[D_6]DMSO$): δ (ppm) = 26.2, 26.7, 38.0, 40.0, 47.1, 110.7, 119.1, 123.9, 127.2, 132.8, 145.1, 165.7; HRMS (APCI, direct probe) *m/z* $[C_{12}H_{17}N_5O+H]^+$ calc.: 248.1506, found: 248.1488; Elemental analysis calcd. for $C_{12}H_{19}Cl_2N_5O$: C 45.01, H 5.98, N 21.87, found: C 44.27, H 6.07, N 21.72.

2-[5-(1*H*-Benzotriazol-1-yl)pentyl]isoindoline-1,3-dione (47)

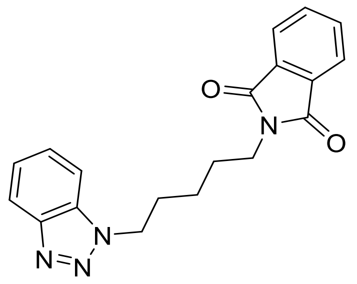

Benzotriazole (269 mg, 2.26 mmol) was reacted with 2-(5-bromopentyl)isoindoline-1,3-dione (668 mg, 2.26 mmol) following the procedure described for the synthesis of **43**. Purification by silica gel chromatography (cyclohexane/ethyl acetate 7:3) afforded **47** (341 mg, 45%) as a solid. $C_{19}H_{18}N_4O_2$ (334.4); mp 80 – 82 °C; ^{1}H NMR (400 MHz, $CDCl_3$): δ (ppm) = 1.38 – 1.47 (m, 2H), 1.74 (p, J = 7.2 Hz, 2H), 2.09 (p, J = 7.7 Hz, 2H), 3.67 (t, J = 7.2 Hz, 2H), 4.64 (t, J = 7.2 Hz, 2H), 7.36 (ddd, J = 8.4, 6.8 and 1.1 Hz, 1H), 7.46 – 7.57 (m, 2H), 7.68 – 7.74 (m, 2H), 7.79 – 7.85 (m, 2H), 8.05 (dt, J = 8.4 and 1.0 Hz, 1H); HRMS (APCI, direct probe) *m/z* $[M+H]^+$ calc.: 334.1503, found: 334.1484.

5-(1*H*-Benzotriazol-1-yl)pentan-1-amine (48)

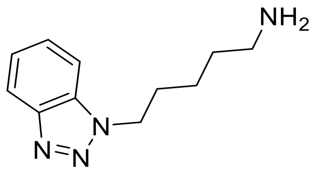

Compound **47** (325 mg, 0.97 mmol) was reacted with hydrazine monohydrate (291 mg, 5.81 mmol) following a procedure analogous to that used for the synthesis of **17**, affording **48** (142 mg, 72%) as an oil. $C_{11}H_{16}N_4$ (204.3); ^{1}H NMR (400 MHz, $CDCl_3$): δ (ppm) = 1.35 – 1.43 (m, 2H), 1.51 (p, J = 7.4 Hz, 2H), 2.04 (p, J = 7.2 Hz, 2H), 2.69 (t, J = 6.8 Hz, 2H), 4.64 (t, J = 7.0 Hz, 2H), 7.34 – 7.38 (m, 1H), 7.45 – 7.55 (m, 2H), 8.05 (dt, J = 8.4 and 0.9 Hz, 1H); HRMS (APCI, direct probe) *m/z* $[M+H]^+$ calc.: 205.1448, found: 205.1445.

***tert*-Butyl (2-{[5-(1*H*-benzotriazol-1-yl)pentyl]amino}-2-oxoethyl)carbamate (49)**

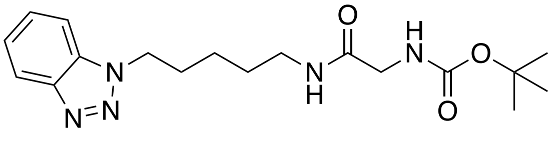

Compound **48** (135 mg, 0.66 mmol) was treated with *N*-(*tert*-butoxycarbonyl)glycine (128 mg, 0.73 mmol) under conditions analogous to those employed in the synthesis of **18**. Purification by chromatography on silica gel (ethyl acetate) afforded **49** (179 mg, 75%) as an oil. $C_{18}H_{27}N_5O_3$ (361.4); ^{1}H NMR (400 MHz, $[D_6]CDCl_3$): δ (ppm) = 1.30 – 1.39 (m, 2H), 1.44 (s, 9H), 1.53 – 1.61 (m, 2H), 2.03 (p, J = 7.0 Hz, 2H), 3.25 (q, J = 6.6 Hz, 2H), 3.75 (s, 2H), 4.65

(t, *J* = 6.9 Hz, 2H), 5.24 (s, 1H), 6.23 (t, *J* = 5.5 Hz, 1H), 7.38 (ddd, *J* = 8.3, 6.5 and 1.3 Hz, 1H), 7.48 – 7.56 (m, 2H), 8.06 (dt, *J* = 8.3 and 1.0 Hz, 1H); HRMS (APCI, direct probe) *m/z* $[M+H]^+$ calc.: 362.2187, found: 362.2156.

_N_-[5-(1_H_-Benzotriazol-1-yl)pentyl]-2-aminoacetamide dihydrochloride (50)

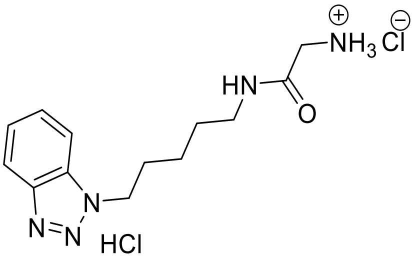

Compound **49** (169 mg, 0.47 mmol) was subjected to reaction conditions similar to those used for the synthesis of **39**, yielding **50** as a solid (104 mg, 67%). $C_{13}H_{21}Cl_2N_5O$ (334.2); HPLC purity (method 2): > 99%; ^1H NMR (600 MHz, [D$_6$]DMSO): δ (ppm) = 1.25 – 1.31 (m, 2H), 1.46 (p, *J* = 7.2 Hz, 2H), 1.92 (p, *J* = 7.2 Hz, 2H), 3.08 (td, *J* = 6.9 and 5.5 Hz, 2H), 3.48 (q, *J* = 5.8 Hz, 2H), 4.71 (t, *J* = 7.1 Hz, 2H), 7.40 (ddd, *J* = 8.2, 6.9 and 1.0 Hz, 1H), 7.55 (ddd, *J* = 8.2, 6.9 and 1.0 Hz, 1H), 7.91 (dt, *J* = 8.4 and 1.0 Hz, 1H), 8.03 (dt, *J* = 8.4 and 1.0 Hz, 1H), 8.18 (s, 3H), 8.49 (t, *J* = 5.7 Hz, 1H); ^{13}C NMR (151 MHz, [D$_6$]DMSO): δ (ppm) = 23.4, 28.2, 28.8, 38.3, 40.0, 47.4, 110.6, 119.1, 123.9, 127.2, 132.8, 145.1, 165.6; HRMS (APCI, direct probe) *m/z* $[C_{13}H_{19}N_5O+H]^+$ calc.: 262.1662, found: 262.1645.

2-[7-(1_H_-Benzotriazol-1-yl)heptyl]isoindoline-1,3-dione (51)

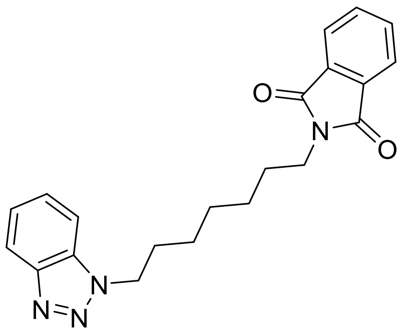

Benzotriazole (180 mg, 1.51 mmol) was reacted with 2-(7-bromoheptyl)isoindoline-1,3-dione[1] (489 mg, 1.51 mmol) following the procedure described for the synthesis of **43**. Purification by silica gel chromatography (cyclohexane/ethyl acetate 8:2) afforded **51** (258 mg, 47%) as a solid. $C_{21}H_{22}N_4O_2$ (362.4); ^1H NMR (400 MHz, $CDCl_3$): δ (ppm) = 1.29 – 1.44 (m, 6H), 1.65 (p, *J* = 7.2 Hz, 2H), 2.01 (p, *J* = 7.0 Hz, 2H), 3.66 (t, *J* = 7.4 Hz, 2H), 4.63 (t, *J* = 7.1 Hz, 2H), 7.36 (ddd, *J* = 8.4, 6.6 and 1.3 Hz, 1H), 7.45 – 7.55 (m, 2H), 7.68 – 7.73 (m, 2H), 7.80 – 7.86 (m, 2H), 8.06 (dt, *J* = 8.4 and 0.9 Hz, 1H); HRMS (APCI, direct probe) *m/z* $[M+H]^+$ calc.: 363.1816, found: 363.1834.

7-(1*H*-Benzotriazol-1-yl)heptan-1-amine (52)

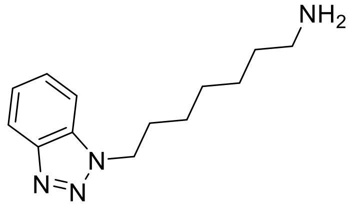

Compound **51** (250 mg, 0.69 mmol) was reacted with hydrazine monohydrate (291 mg, 5.81 mmol) following a procedure analogous to that used for the synthesis of **17**, affording **52** (158 mg, 99%) as an oil. $C_{13}H_{20}N_4$ (232.3); ^{1}H NMR (400 MHz, $CDCl_3$): δ (ppm) = 1.19 – 1.50 (m, 8H), 1.97 – 2.06 (m, 2H), 2.68 (t, *J* = 7.0 Hz, 2H), 4.63 (t, *J* = 7.1 Hz, 2H,), 7.36 (ddd, *J* = 8.4, 6.6 and 1.3 Hz, 1H), 7.46 – 7.54 (m, 2H), 8.06 (dt, *J* = 8.3 and 1.0 Hz, 1H); HRMS (APCI, direct probe) *m/z* $[M+H]^+$ calc.: 233.1761, found: 233.1767.

***tert*-Butyl (2-{[7-(1*H*-benzotriazol-1-yl)heptyl]amino}-2-oxoethyl)carbamate (53)**

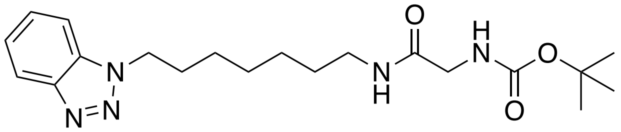

Compound **52** (154 mg, 0.66 mmol) was treated with *N*-(*tert*-butoxycarbonyl)glycine (128 mg, 0.73 mmol) under conditions analogous to those employed in the synthesis of **18**. Purification by chromatography on silica gel (gradient elution: cyclohexane/ethyl acetate 1:1 to 2:8) afforded **53** (166 mg, 64%) as an oil. $C_{20}H_{31}N_5O_3$ (389.5); ^{1}H NMR (400 MHz, $CDCl_3$): δ (ppm) = 1.21 – 1.40 (m, 6H), 1.44 (s, 11H), 1.98 – 2.06 (m, 2H), 3.24 (td, *J* = 7.1 and 5.8 Hz, 2H), 3.76 (s, 2H), 4.65 (t, *J* = 7.0 Hz, 2H), 5.14 (s, 1H), 6.11 (s, 1H), 7.40 (ddd, *J* = 8.4, 6.5 and 1.4 Hz, 1H), 7.48 – 7.56 (m, 2H), 8.08 (dt, *J* = 8.4 and 1.0 Hz, 1H); HRMS (APCI, direct probe) *m/z* $[M+H]^+$ calc.: 390.2500, found: 390.2486.

***N*-[7-(1*H*-Benzotriazol-1-yl)heptyl]-2-aminoacetamide dihydrochloride (54)**

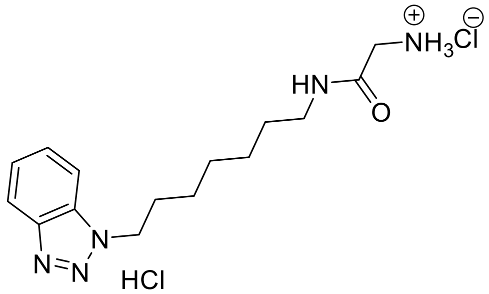

Compound **53** (160 mg, 0.41 mmol) was subjected to reaction conditions similar to those used for the synthesis of **22**, yielding **54** as a solid (93 mg, 62%). $C_{15}H_{25}Cl_2N_5O$ (362.3); HPLC purity (method 1): > 99%; ^{1}H NMR (400 MHz, [D$_6$]DMSO): δ (ppm) = 1.17 – 1.44 (m, 8H),

1.90 (p, J = 7.1 Hz, 2H), 3.06 (td, J = 6.9 and 5.5 Hz, 2H), 3.48 (q, J = 5.9 Hz, 2H), 4.70 (t, J = 6.9 Hz, 2H), 7.40 (ddd, J = 8.1, 7.0 and 1.0 Hz, 1H), 7.55 (ddd, J = 8.3, 6.9 and 1.1 Hz, 1H), 7.89 (dt, J = 8.4 and 1.0 Hz, 1H), 8.03 (dt, J = 8.4 and 1.0 Hz, 1H), 8.17 (s, 3H), 8.45 (t, J = 5.5 Hz, 1H); ^{13}C NMR (101 MHz, [D$_6$]DMSO): δ (ppm) = 26.0, 26.2, 28.1, 28.8, 29.2, 38.6, 40.0, 47.4, 110.6, 119.1, 123.9, 127.1, 132.8, 145.1, 165.6; HRMS (APCI, direct probe) *m/z* [$C_{15}H_{23}N_5O$+H]$^+$ calc.: 290.1975, found: 290.1997.

2-[8-(1*H*-Benzotriazol-1-yl)octyl]isoindoline-1,3-dione (55)

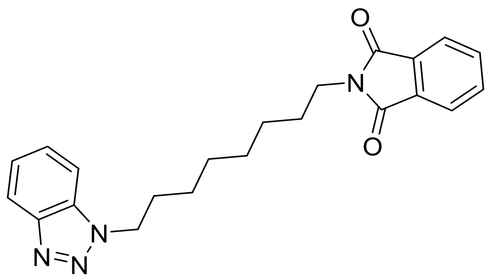

Benzotriazole (192 mg, 1.61 mmol) was reacted with 2-(8-bromooctyl)isoindoline-1,3-dione[2] (543 mg, 1.61 mmol) following the procedure described for the synthesis of **43**. Purification by silica gel chromatography (cyclohexane/erthyl acetate 3:1) afforded **55** (315 mg, 52%) as a solid. $C_{22}H_{24}N_4O_2$ (376.5); mp 54 – 56 °C; ^{1}H NMR (400 MHz, $CDCl_3$): δ (ppm) = 1.28 – 1.38 (m, 8H), 1.64 (p, J = 6.8 Hz, 2H), 2.00 (p, J = 7.1 Hz, 2H), 3.62 – 3.69 (m, 2H), 4.63 (t, J = 7.1 Hz, 2H), 7.34 – 7.40 (m, 1H), 7.45 – 7.55 (m, 2H), 7.67 – 7.73 (m, 2H), 7.79 – 7.87 (m, 2H), 8.04 – 8.09 (dq, J = 8.4 and 0.8 Hz, 1H); HRMS (APCI, direct probe) *m/z* [M+H]$^+$ calc.: 377.1972, found: 377.1915.

8-(1*H*-Benzotriazol-1-yl)octan-1-amine (56)

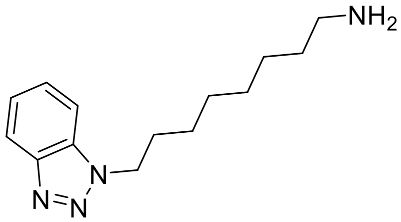

Compound **55** (300 mg, 0.80 mmol) was reacted with hydrazine monohydrate (240 mg, 4.79 mmol) following a procedure analogous to that used for the synthesis of **17**, affording **56** (173 mg, 88%) as an oil. $C_{14}H_{22}N_4$ (246.4); ^{1}H NMR (400 MHz, $CDCl_3$): δ (ppm) = 1.23 – 1.36 (m, 8H), 1.37 – 1.48 (m, 2H), 1.93 – 2.06 (m, 2H), 2.61 – 2.74 (m, 2H), 4.63 (t, J = 7.1 Hz, 2H), 7.36 (ddd, J = 8.1, 6.6 and 1.3 Hz, 1H), 7.45 – 7.54 (m, 2H), 8.06 (dt, J = 8.3 and 1.0 Hz, 1H); HRMS (APCI, direct probe) *m/z* [M+H]$^+$ calc.: 247.1917, found: 247.1990.

***tert*-Butyl (2-{[8-(1*H*-benzotriazol-1-yl)octyl]amino}-2-oxoethyl)carbamate (57)**

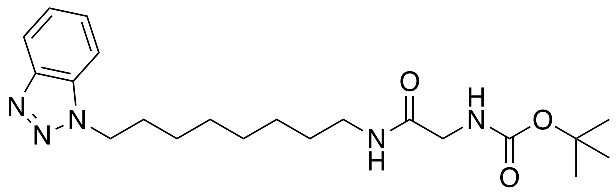

Compound **56** (163 mg, 0.66 mmol) was treated with *N*-(*tert*-butoxycarbonyl)glycine (128 mg, 0.73 mmol) under conditions analogous to those employed in the synthesis of **18**. Purification by chromatography on silica gel (gradient elution: cyclohexane/ethyl acetate 1:1 to 2:8) afforded **57** (220 mg, 82%) as an oil. $C_{21}H_{33}N_5O_3$ (403.5); ^{1}H NMR (400 MHz, $CDCl_3$): δ (ppm) = 1.26 – 1.36 (m, 8H), 1.42 – 1.51 (m, 11H), 2.02 (p, *J* = 7.0 Hz, 2H), 3.24 (q, *J* = 6.7 Hz, 2H), 3.77 (s, 2H), 4.64 (t, *J* = 7.1 Hz, 2H), 5.19 (s, 1H), 6.13 (s, 1H), 7.38 (ddd, *J* = 8.4, 6.6 and 1.4 Hz, 1H), 7.47 – 7.55 (m, 2H), 8.07 (dt, *J* = 8.4 and 0.9 Hz, 1H); HRMS (APCI, direct probe) *m/z* $[M+H]^+$ calc.: 404.2656, found: 404.2618.

***N*-[8-(1*H*-Benzotriazol-1-yl)octyl]-2-aminoacetamide dihydrochloride (58)**

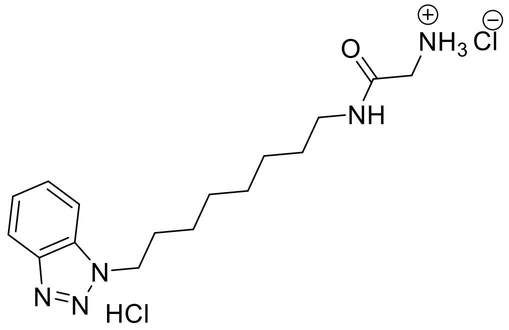

Compound **57** (208 mg, 0.52 mmol) was subjected to reaction conditions similar to those used for the synthesis of **39**, yielding **58** as a solid (153 mg, 79%). $C_{16}H_{27}Cl_2N_5O$ (376.3); HPLC purity (method 1): > 99%; ^{1}H NMR (600 MHz, [D$_6$]DMSO): δ (ppm) = 1.18 – 1.30 (m, 8H), 1.37 (p, *J* = 7.0 Hz, 2H), 1.90 (p, *J* = 7.1 Hz, 2H), 3.07 (td, *J* = 7.0 and 5.5 Hz, 2H), 3.49 (q, *J* = 5.8 Hz, 2H), 4.70 (t, *J* = 7.0 Hz, 2H), 7.40 (ddd, *J* = 8.1, 6.9 and 1.0 Hz, 1H), 7.55 (ddd, *J* = 8.2, 6.8 and 1.0 Hz, 1H), 7.89 (dt, *J* = 8.4 and 1.0 Hz, 1H), 8.03 (dt, *J* = 8.3 and 1.0 Hz, 1H), 8.16 (s, 3H), 8.43 (t, *J* = 5.6 Hz, 1H); ^{13}C NMR (151 MHz, [D$_6$]DMSO): δ (ppm) = 26.0, 26.20, 28.3, 28.5, 28.8, 29.2, 38.6, 40.0, 47.4, 110.6, 119.1, 123.9, 127.2, 132.8, 145.1, 165.6; HRMS (APCI, direct probe) *m/z* $[C_{16}H_{25}N_5O+H]^+$ calc.: 304.2132, found: 304.2169.

Phenyl [6-(5-{4-cyanophenyl}-2*H*-tetrazol-2-yl)hexyl]carbamate (69)

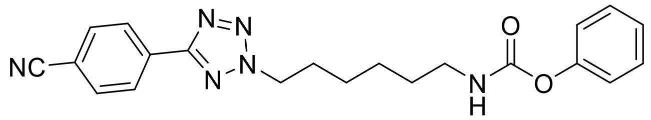

Diphenyl carbonate (121 mg, 0.56 mmol) was ground and suspended in a mixture of tetrahydrofuran (THF) and water (1:9, 2 mL). A solution of 4-[2-(6-aminohexyl)-2*H*-tetrazol-5-yl]benzonitrile [3] (151 mg, 0.56 mmol) in THF (400 µL) was added, and the suspension was stirred and heated at 40 °C for 2 h. Upon completion of the reaction, the mixture was diluted with water (20 mL) and exhaustively extracted with ethyl acetate. The combined organic extracts were dried over anhydrous sodium sulfate and concentrated under reduced pressure. The crude product was purified by column chromatography on silica gel (gradient: cyclohexane to cyclohexane/ethyl acetate 6:4), affording **69** as a solid (211 mg, 97%). $C_{21}H_{22}N_6O_2$ (390.4); mp 128 – 129 °C; ^{1}H NMR (400 MHz, $CDCl_3$): δ (ppm) = 1.24 – 1.44 (m, 4H), 1.58 (p, *J* = 7.6 Hz, 2H), 2.10 (p, *J* = 7.0 Hz, 2H), 3.26 (q, *J* = 6.7 Hz, 2H), 4.68 (t, *J* = 7.0 Hz, 2H), 5.04 (s, 1H), 7.07 – 7.12 (m, 2H), 7.16 – 7.21 (m, 1H), 7.30 – 7.39 (m, 2H), 7.74 – 7.81 (m, 2H), 8.23 – 8.30 (m, 2H); HRMS (APCI, direct probe) *m/z* $[M+H]^+$ calc.: 391.1913, found: 391.1877.

***N*-[6-(5-{4-Cyanophenyl}-2*H*-tetrazol-2-yl)hexyl]hydrazinecarboxamide (70)**

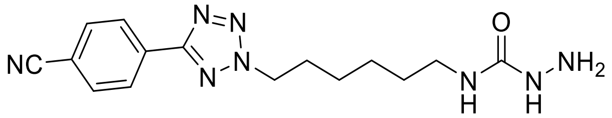

To a solution of **69** (211 mg, 0.54 mmol) in 1,2-dimethoxyethane (2 mL), hydrazine monohydrate (775 mg, 15.5 mmol) was added under stirring. The mixture was then heated at 80 °C for 4 h. After completion, the crude product was purified by chromatography on silica gel (gradient elution: dichloromethane to dichloromethane/methanol 9:1). The product-containing fractions were combined and concentrated under reduced pressure. The resulting solid was suspended in ethyl acetate, filtered, and the filter cake was dried *in vacuo* to yield **70** as a solid (150 mg, 85%). $C_{15}H_{20}N_8O$ (328.4); mp 188 – 1907 °C; HPLC purity (method 1): > 99%; ^{1}H NMR (600 MHz, [D_6]DMSO): δ (ppm) = δ (ppm) = 1.27 – 1.33 (m, 4H), 1.37 (p, *J* = 6.9 Hz, 2H), 1.97 (p, *J* = 7.6 Hz, 2H), 2.99 (q, *J* = 6.8 Hz, 2H), 4.03 (s, 2H), 4.76 (t, *J* = 7.0 Hz, 2H), 6.27 (s, 1H), 6.82 (s, 1H), 8.01 – 8.06 (m, 2H), 8.20 – 8.26 (m, 2H); ^{13}C NMR (151 MHz, [D6]DMSO): δ (ppm) = 25.5, 25.6, 28.6, 29.8, 38.7, 53.0, 112.9, 118.4, 127.1, 131.1, 133.3, 160.2, 162.7; HRMS (APCI, direct probe) *m/z* $[M+H]^+$ calc.: 329.1833, found: 329.1848.

Phenyl (3-{[5-(4-cyanophenyl)-2*H*-tetrazol-2-yl]methyl}benzyl)carbamate (71)

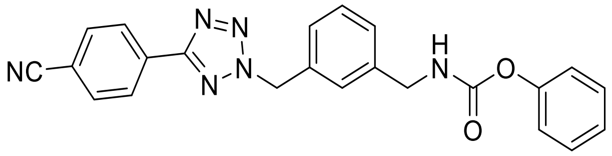

Diphenyl carbonate (83 mg, 0.39 mmol) was ground and suspended in a mixture of tetrahydrofuran (THF) and water (1:9, 2 mL). A solution of 4-{2-[3-(aminomethyl)benzyl]-2*H*-tetrazol-5-yl}benzonitrile[3] (113 mg, 0.39 mmol) in THF (400 µL) was added, and the suspension was stirred and heated at 40 °C for 2 h. Upon completion of the reaction, the mixture was exhaustively extracted with ethyl acetate. The remaining aqueous phase was diluted with water (20 mL) and extracted again thoroughly with ethyl acetate. The combined organic extracts were dried over anhydrous sodium sulfate and concentrated under reduced pressure. The crude product was purified by column chromatography on silica gel (cyclohexane/ethyl acetate 6:4), affording **71** as a solid (130 mg, 82%). $C_{23}H_{18}N_6O_2$ (410.4); mp 157 – 159 °C; ^{1}H NMR (400 MHz, $CDCl_3$): δ (ppm) = 4.47 (d, J = 6.1 Hz, 2H), 5.38 (s, 2H), 5.83 (s, 2H), 7.11 – 7.24 (m, 2H), 7.31 – 7.49 (m, 6H), 7.71 – 7.79 (m, 2H), 8.22 – 8.28 (m, 2H); HRMS (APCI, direct probe) *m/z* $[M+H]^+$ calc.: 411.1564, found: 411.1468.

***N*-(3-{[5-(4-Cyanophenyl)-2*H*-tetrazol-2-yl]methyl}benzyl)hydrazinecarboxamide (72)**

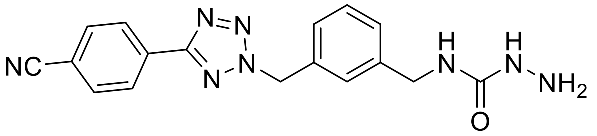

To a solution of **71** (130 mg, 0.32 mmol) in 1,2-dimethoxyethane (2 mL), hydrazine monohydrate (145 mg, 2.90 mmol) was added under stirring. The mixture was then heated at 80 °C for 6 h. After completion, the crude product was purified by chromatography on silica gel (gradient elution: dichloromethane to dichloromethane/methanol 9:1). The product-containing fractions were combined and concentrated under reduced pressure. The resulting solid was suspended in ethyl acetate, filtered, and the filter cake was dried *in vacuo* to yield **72** as a solid (45 mg, 41%). $C_{17}H_{16}N_8O$ (348.4); mp 184 – 187 °C; HPLC purity (method 1): > 99%; ^{1}H NMR (600 MHz, [D$_6$]DMSO): δ (ppm) = 4.09 (s, 2H), 4.24 (d, J = 6.3 Hz, 2H), 6.01 (s, 2H), 6.87 (s, 1H), 7.02 (s, 1H), 7.26 – 7.29 (m, 2H), 7.30 – 7.49 (m, 2H), 8.01 –8.04 (m, 2H), 8.20 – 8.24 (m, 2H); ^{13}C NMR (151 MHz, [D6]DMSO): δ (ppm) = 42.4, 56.4, 113.0, 118.3, 126.7, 127.0, 127.1, 127.4, 128.8, 130.9, 133.3, 133.7, 141.7, 159.6, 163.1; HRMS (ESI) *m/z* $[M+H]^+$ calc.: 349.1520, found: 349.1533.

Phenyl [4-(1*H*-benzotriazol-1-yl)butyl]carbamate (73)

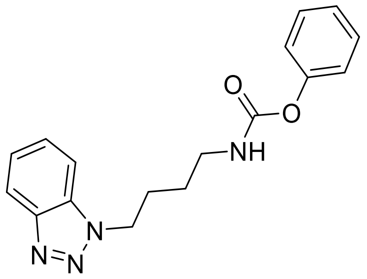

Diphenyl carbonate (161 mg, 0.75 mmol) was ground and suspended in a mixture of THF and water (1:9, 2 mL). A suspension of **44** (142 mg, 0.75 mmol) in THF/water (1:9, 2 mL) was added dropwise under stirring, and the mixture was further stirred at 40 °C for 2 h. Upon completion of the reaction, the mixture was diluted with water and exhaustively extracted with ethyl acetate. The combined organic layers were dried over anhydrous sodium sulfate and concentrated under reduced pressure. The crude product was purified by column chromatography on silica gel (gradient elution: cyclohexane/ethyl acetate 9:1 to 1:1), affording **73** as a solid (128 mg, 55%). $C_{17}H_{18}N_4O_2$ (310.4); mp 82 – 84 °C; ^{1}H NMR (400 MHz, $CDCl_3$): δ (ppm) = 1.64 (p, *J* = 7.1 Hz, 2H), 2.13 (p, *J* = 7.2 Hz, 2H), 3.33 (q, *J* = 6.5 Hz, 2H), 4.72 (t, *J* = 7.0 Hz, 2H), 5.10 (s, 1H), 7.07 – 7.12 (m, 2H), 7.16 – 7.22 (m, 1H), 7.31 – 7.39 (m, 2H), 7.41 (ddd, *J* = 8.0, 6.8 and 1.1 Hz, 1H), 7.48 – 7.60 (m, 2H), 8.10 (dt, *J* = 8.3 and 1.0 Hz, 1H); HRMS (ESI) *m/z* $[M+H]^+$ calc.: 311.1503, found: 311.1503.

***N*-[4-(1*H*-Benzotriazol-1-yl)butyl]hydrazinecarboxamide (74)**

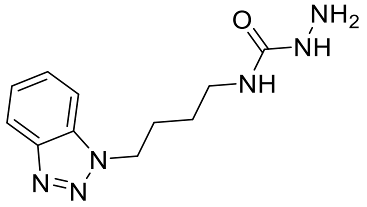

A solution of **73** (118 mg, 0.38 mmol) in 1,2-dimethoxyethane (1 mL) was treated with hydrazine monohydrate (190 mg, 3.80 mmol) and stirred at 80 °C for 6 h. After completion, the crude product was purified by chromatography on silica gel (gradient elution: dichloromethane to dichloromethane/methanol 9:1). The product-containing fractions were combined and concentrated to a few mL under reduced pressure. By adding ethyl acetate, the desired product **74** (54 mg, 57%) precipitated. $C_{11}H_{16}N_6O$ (248.3); mp 114 – 117 °C; HPLC purity (method 1): > 99%; ^{1}H NMR (600 MHz, $[D_6]$DMSO): δ (ppm) = 1.37 (p, *J* = 7.3 Hz, 2H), 1.89 (p, *J* = 7.2 Hz, 2H), 3.06 (q, *J* = 6.7 Hz, 2H), 4.03 (s, 2H), 4.72 (t, *J* = 7.1 Hz, 2H), 6.36 (s, 1H), 6.86 (s, 1H), 7.40 (ddd, *J* = 8.0, 6.9 and 1.0 Hz, 1H), 7.55 (ddd, *J* = 8.2, 6.9 and 1.0 Hz, 1H), 7.91 (dt, *J* = 8.5 and 1.0 Hz, 1H), 8.03 (dt, *J* = 8.4 and 0.9 Hz, 1H); ^{13}C NMR (151 MHz, $[D_6]$DMSO): δ (ppm) = 26.7, 27.4, 38.1, 47.2, 110.6, 119.1, 123.9, 127.1, 132.8, 145.1, 160.3; HRMS (APCI, direct probe) *m/z* $[M+H]^+$ calc.: 249.1458, found: 249.1471.

Phenyl [5-(1*H*-benzotriazol-1-yl)pentyl]carbamate (75)

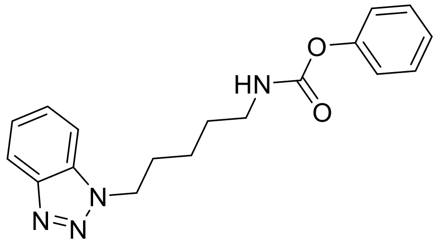

Compound **48** (139 mg, 0.68 mmol) was treated with diphenyl carbonate (146 mg, 0.68 mmol) under conditions analogous to those employed in the synthesis of **73**. Purification by chromatography on silica gel (gradient elution: cyclohexane to cyclohexane/ethyl acetate 1:1) afforded **75** (131 mg, 59%) as a solid. $C_{18}H_{20}N_4O_2$ (324.4); mp 101 – 103 °C; ^{1}H NMR (400 MHz, $CDCl_3$): δ (ppm) = 1.38 – 1.48 (m, 2H), 1.64 (p, *J* = 7.2 Hz, 2H), 2.09 (p, *J* = 7.1 Hz, 2H), 3.25 (q, *J* = 6.6 Hz, 2H), 4.68 (t, *J* = 7.0 Hz, 2H), 5.06 (s, 1H), 7.07 – 7.12 (m, 2H), 7.16 – 7.21 (m, 1H), 7.32 – 7.43 (m, 3H), 7.48 – 7.58 (m, 2H), 8.09 (dt, *J* = 8.4 and 1.0 Hz, 1H); HRMS (ESI) *m/z* $[M+H]^+$ calc.: 325.1659, found: 325.1670.

***N*-(5-(1*H*-Benzotriazol-1-yl)pentyl)hydrazinecarboxamide (76)**

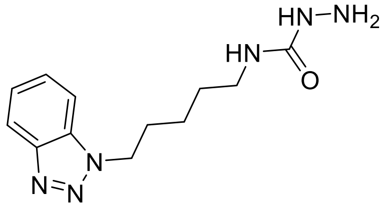

Compound **75** (121 mg, 0.37 mmol) was reacted with hydrazine monohydrate (185 mg, 3.70 mmol) following the same procedure described for the synthesis of **74**, affording **76** (56 mg, 57%) as a solid. $C_{12}H_{18}N_6O$ (262.3); mp 123 – 126 °C; HPLC purity (method 1): > 99%; ^{1}H NMR (600 MHz, [D$_6$]DMSO): δ (ppm) = 1.20 – 1.28 (m, 2H), 1.42 (p, *J* = 7.2 Hz, 2H), 1.92 (p, *J* = 7.3 Hz, 2H), 2.98 (td, *J* = 7.0 and 6.0 Hz, 2H), 4.02 (s, 2H), 4.70 (t, *J* = 7.1 Hz, 2H), 6.29 (s, 1H), 6.84 (s, 1H), 7.40 (ddd, *J* = 8.3, 6.9 and 1.0 Hz, 1H), 7.55 (ddd, *J* = 8.2, 6.9 and 1.0 Hz, 1H), 7.90 (dt, *J* = 8.4 and 1.0 Hz, 1H), 8.03 (dt, *J* = 8.4 and 1.0 Hz, 1H); ^{13}C NMR (151 MHz, [D$_6$]DMSO): δ (ppm) =23.4, 29.0, 29.5, 38.6, 47.4, 110.6, 119.1, 123.9, 127.1, 132.8, 145.1, 160.2; HRMS (APCI, direct probe) *m/z* $[M+H]^+$ calc.: 263.1615, found: 263.1633.

Phenyl [7-(1*H*-benzotriazol-1-yl)heptyl]carbamate (79)

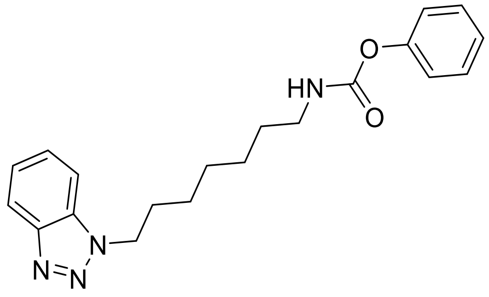

Compound **52** (151 mg, 0.65 mmol) was treated with diphenyl carbonate (139 mg, 0.65 mmol) under conditions analogous to those employed in the synthesis of **73**. Purification by chromatography on silica gel (gradient elution: cyclohexane to cyclohexane/ethyl acetate 6:4) afforded **79** (173 mg, 76%) as a solid. $C_{20}H_{24}N_4O_2$ (352.4); mp 94 – 96 °C; ^{1}H NMR (400 MHz, $CDCl_3$): δ (ppm) = 1.30 – 1.42 (m, 6H), 1.54 (p, *J* = 7.1 Hz, 2H), 2.03 (p, *J* = 6.9 Hz, 2H), 3.24 (q, *J* = 6.4 Hz, 2H), 4.65 (t, *J* = 7.1 Hz, 2H), 5.00 (s, 1H), 7.09 – 7.14 (m, 2H), 7.18 (t, *J* = 7.4 Hz, 1H), 7.31 – 7.41 (m, 3H), 7.47 – 7.56 (m, 2H), 8.08 (dt, *J* = 8.3 and 1.0 Hz, 1H); HRMS (APCI, direct probe) *m/z* $[M+H]^+$ calc.: 353.1972, found: 353.1987.

***N*-[7-(1*H*-Benzotriazol-1-yl)heptyl]hydrazinecarboxamide (80)**

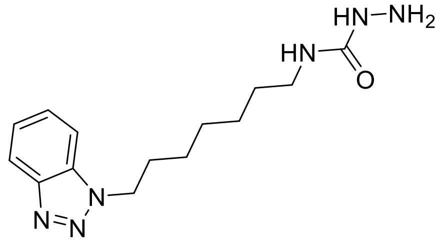

Compound **79** (154 mg, 0.44 mmol) was reacted with hydrazine monohydrate (220 mg, 4.39 mmol) following the same procedure described for the synthesis of **74**, affording **80** (28 mg, 22%) as a solid. $C_{14}H_{22}N_6O$ (290.4); mp 121 – 123 °C; HPLC purity (method 1): > 99%; ^{1}H NMR (600 MHz, [D_6]DMSO): δ (ppm) = 1.16 – 1.36 (m, 8H), 1.90 (p, *J* = 7.1 Hz, 2H), 2.97 (td, *J* = 7.0 and 6.0 Hz, 2H), 4.03 (s, 2H), 4.70 (t, *J* = 7.0 Hz, 2H), 6.24 (s, 1H), 6.81 (s, 1H), 7.40 (ddd, *J* = 8.3, 6.9 and 1.0 Hz, 1H), 7.55 (ddd, *J* = 8.3, 6.9 and 1.0 Hz, 1H), 7.89 (dt, *J* = 8.3 and 1.0 Hz, 1H), 8.03 (dt, *J* = 8.4 and 1.0 Hz, 1H); ^{13}C NMR (151 MHz, [D_6]DMSO): δ (ppm) = 26.1, 26.2, 28.2, 29.2, 30.0, 38.8, 47.4, 110.6, 119.1, 123.9, 127.1, 132.8, 145.1, 160.2; HRMS (APCI, direct probe) *m/z* $[M+H]^+$ calc.: 291.1928, found: 291.1938.

Phenyl [8-(1*H*-benzotriazol-1-yl)octyl]carbamate (81)

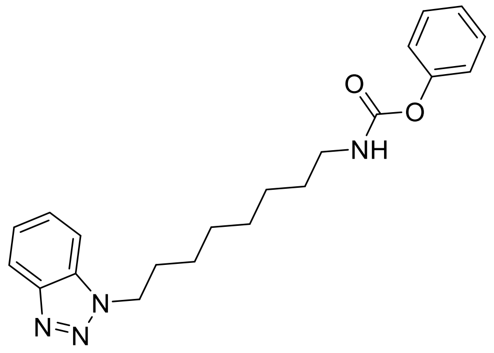

Compound **56** (206 mg, 0.84 mmol) was treated with diphenyl carbonate (180 mg, 0.84 mmol) under conditions analogous to those employed in the synthesis of **73**, affording **81** (203 mg, 66%) as a solid. $C_{21}H_{26}N_4O_2$ (366.5); mp 68 – 70 °C; ^{1}H NMR (400 MHz, $CDCl_3$): δ (ppm) = 1.28 – 1.39 (m, 8H), 1.54 (p, *J* = 6.9 Hz, 2H), 2.03 (p, *J* = 7.0 Hz, 2H), 3.24 (q, *J* = 6.9 Hz, 2H), 4.64 (t, *J* = 7.1 Hz, 2H), 5.01 (s, 1H), 7.09 – 7.15 (m, 2H), 7.15 – 7.21 (m, 1H), 7.30 – 7.41 (m, 3H), 7.46 – 7.56 (m, 2H), 8.07 (dt, *J* = 8.3 and 1.0 Hz, 1H); HRMS (APCI, direct probe) *m/z* $[M+H]^+$ calc.: 367.2129, found: 367.2139.

***N*-[8-(1*H*-Benzotriazol-1-yl)octyl]hydrazinecarboxamide (82)**

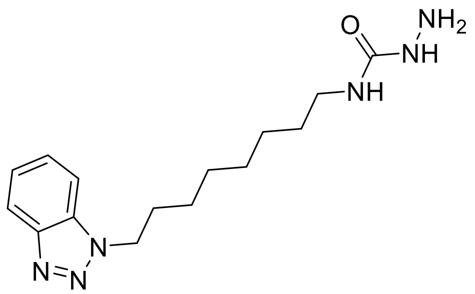

Compound **81** (185 mg, 0.50 mmol) was reacted with hydrazine monohydrate (250 mg, 4.99 mmol) following the same procedure described for the synthesis of **74**, affording **82** (82 mg, 53%) as a solid. $C_{15}H_{24}N_6O$ (304.4); mp 122 – 125 °C; HPLC purity (method 1): > 99%; ^{1}H NMR (600 MHz, [D$_6$]DMSO): δ (ppm) = 1.15 – 1.30 (m, 8H), 1.33 (p, *J* = 7.1 Hz, 2H), 1.90 (p, *J* = 7.1 Hz, 2H), 2.97 (q, *J* = 6.7 Hz, 2H), 4.03 (s, 2H), 4.70 (t, *J* = 7.0 Hz, 2H), 6.24 (s, 1H), 6.82 (s, 1H), 7.40 (ddd, *J* = 8.1, 6.9 and 1.0 Hz, 1H), 7.55 (ddd, *J* = 8.0, 7.0 and 0.9 Hz, 1H), 7.89 (dt, *J* = 8.4 and 1.0 Hz, 1H), 8.03 (dt, *J* = 8.4 and 1.0 Hz, 1H); ^{13}C NMR (151 MHz, [D$_6$]DMSO): δ (ppm) = 26.0, 26.2, 28.4, 28.6, 29.2, 30.0, 38.8, 47.4, 110.6, 119.1, 123.9, 127.1, 132.8, 145.1, 160.2; HRMS (APCI, direct probe) *m/z* $[M+H]^+$ calc.: 305.2084, found: 305.2108.

Phenyl [3-({1*H*-benzotriazol-1-yl}methyl)benzyl]carbamate (83)

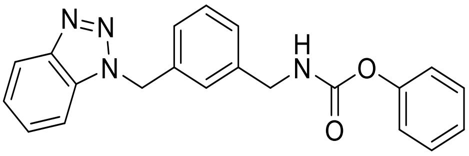

Diphenyl carbonate (56 mg, 0.26 mmol) was ground and suspended in a mixture of tetrahydrofuran (THF) and water (1:9, 2 mL). A solution of **65** (62 mg, 0.26 mmol) in THF (400 µL) was added, and the suspension was stirred and heated at 40 °C for 2 h. Upon completion of the reaction, the mixture was diluted with water and exhaustively extracted with ethyl acetate. The combined organic extracts were dried over anhydrous sodium sulfate and concentrated under reduced pressure. The crude product was purified by column chromatography on silica gel (gradient elution: cyclohexane to cyclohexane/ethyl acetate 6:4), affording **83** as a solid (63 mg, 68%). $C_{21}H_{18}N_4O_2$ (358.4); mp 130 – 131 °C; ^{1}H NMR (400 MHz, $CDCl_3$): δ (ppm) = 4.42 (d, J = 6.1 Hz, 2H), 5.33 (s, 1H), 5.85 (s, 2H), 7.09 – 7.15 (m, 2H), 7.17 – 7.44 (m, 10H), 8.08 (ddd, J = 8.2, 1.3 and 0.8 Hz, 1H); HRMS (APCI, direct probe) *m/z* $[M+H]^+$ calc.: 359.1503, found: 359.1497.

***N*-[3-({1*H*-Benzotriazol-1-yl}methyl)benzyl]hydrazinecarboxamide (84)**

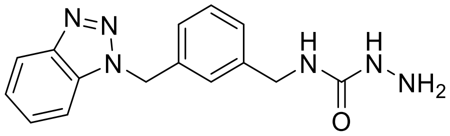

To a solution of **83** (63 mg, 0.18 mmol) in 1,2-dimethoxyethane (2 mL), hydrazine monohydrate (242 mg, 4.84 mmol) was added under stirring. The mixture was then heated at 80 °C for 4 h. After completion, the crude product was purified by column chromatography on silica gel (gradient elution: dichloromethane to dichloromethane/methanol 9:1). The product-containing fractions were combined and concentrated under reduced pressure. The resulting solid was suspended in ethyl acetate, filtered, and the filter cake was dried *in vacuo* to yield **84** as a solid (53 mg, quantitative). $C_{15}H_{16}N_6O$ (296.3); mp 131 – 132 °C; HPLC purity (method 1): > 99%; ^{1}H NMR (600 MHz, [D6]DMSO): δ (ppm) = 4.11 (s, 2H), 4.19 (d, J = 6.3 Hz, 2H), 5.95 (s, 2H), 6.82 (s, 1H), 7.01 (s, 1H), 7.15 (dt, J = 7.6 and 1.6 Hz, 1H), 7.20 (dt, J = 7.7 and 1.4 Hz, 1H), 7.25 – 7.30 (m, 2H), 7.40 (ddd, J = 8.2, 6.9 and 1.0 Hz, 1H), 7.53 (ddd, J = 6.0, 1.3 and 0.8 Hz, 1H), 7.83 (dt, J = 8.4 and 0.9 Hz, 1H), 8.05 (dt, J = 8.4 and 0.9 Hz, 1H); ^{13}C NMR (151 MHz, [D6]DMSO): δ (ppm) = 42.3, 51.0, 110.7, 119.2, 124.0, 125.9, 126.5, 126.8, 127.4, 128.6, 132.7, 135.7, 141.8, 145.3, 160.2; HRMS (ESI) *m/z* $[M+H]^+$ calc.: 297.1458, found: 297.1447.

2. References

1 X. Wang, K. Deng, C. Wang, Y. Li, T. Wang, Z. Huang, Y. Ma, P. Sun, Y. Shi, S. Yang, Y. Fan and R. Xiang, *J. Enzyme Inhib. Med. Chem.* 2020, **35**, 414-423.

2 X. Qin, Z. Ma, X. Yang, S. Hu, X. Chen, D. Liang, Y. Lin, X. Shi, L. Du and M. Li, *Anal. Chem.* 2019, **91**, 12173.

3 Pöstges, F. Galster, J. Kampschulze, W. Hanekamp and M. Lehr, *Bioorg. Med. Chem*. 2024, **98**, 117558.

3. ^{1}H NMR, ^{13}C NMR and HRMS spectra of the target compounds

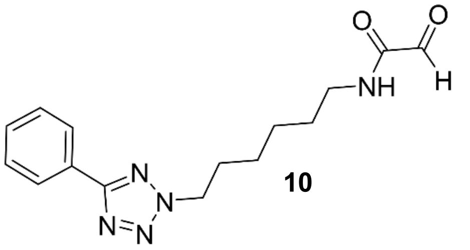

10

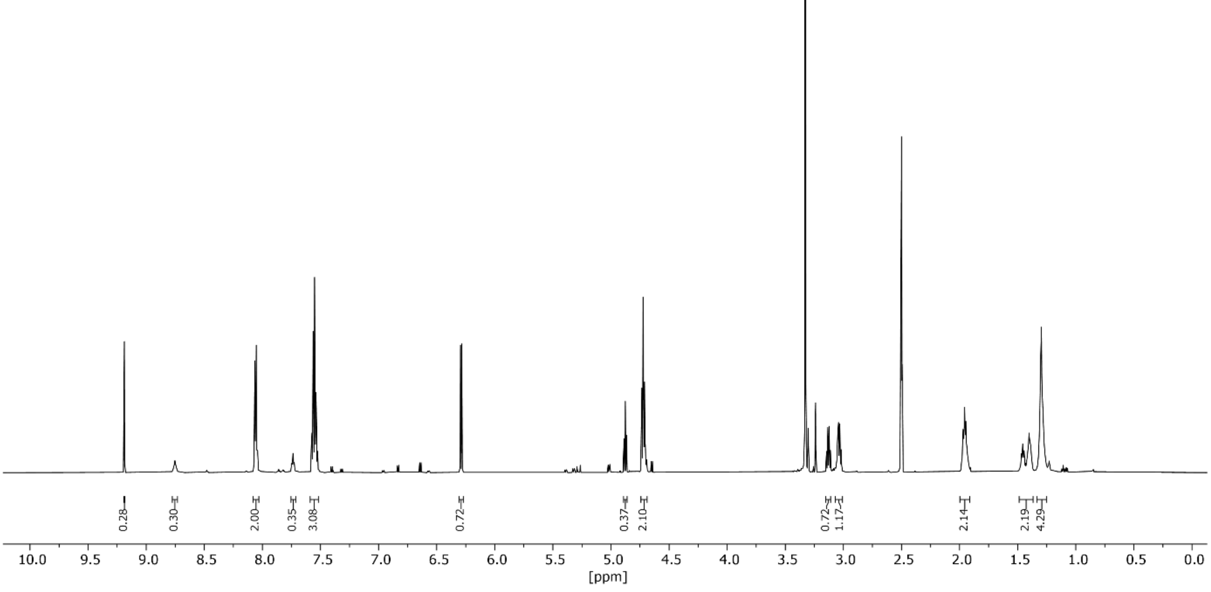

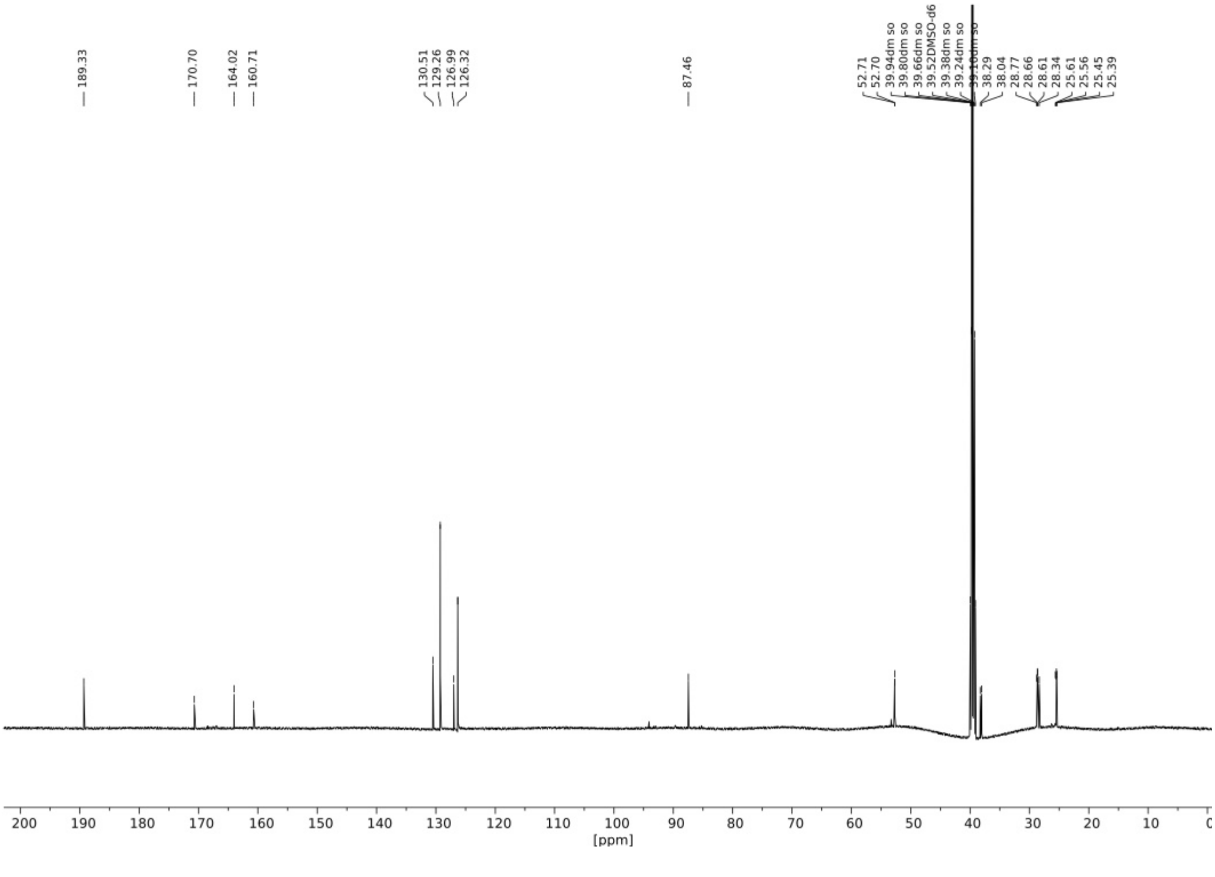

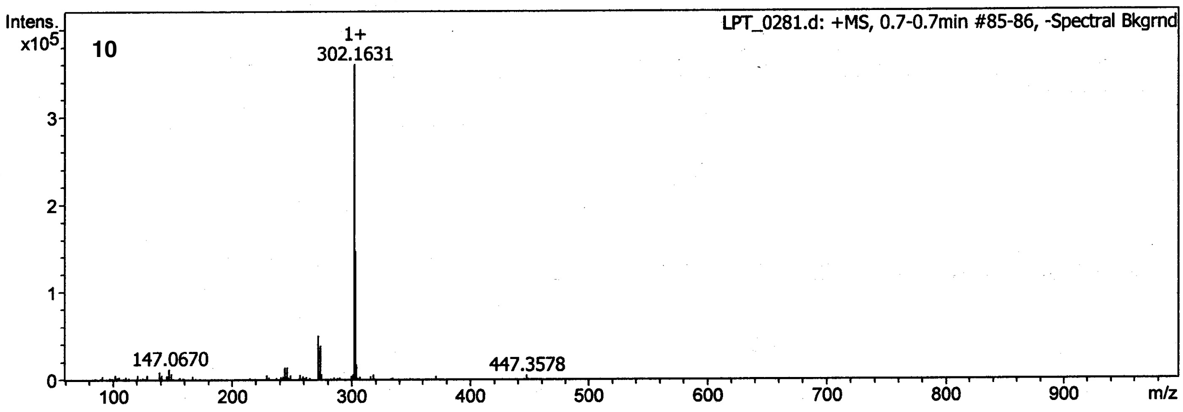
Intens.
x10^5
10
1+
302.1631
LPT_0281.d: +MS, 0.7-0.7min #85-86, -Spectral Bkgrnd
147.0670
447.3578
m/z

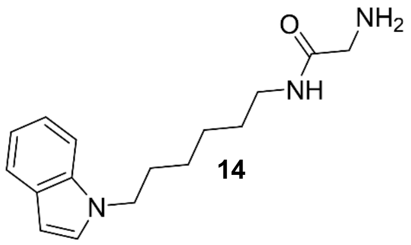
O
NH2
NH
N
14

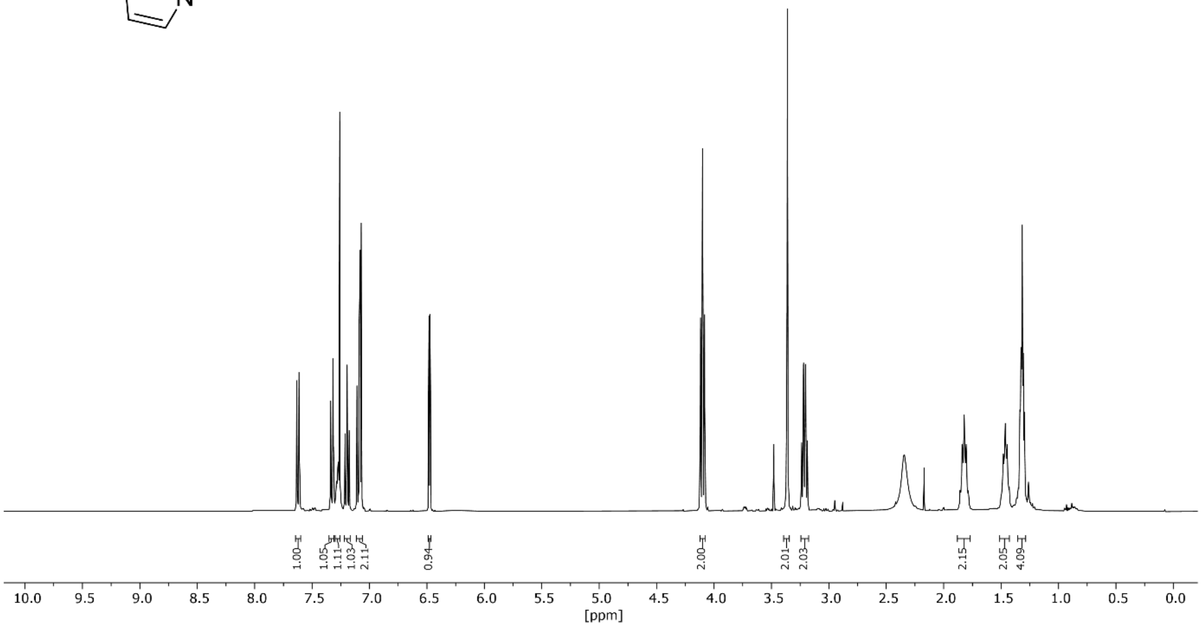
1.00
1.05
1.11
1.03
2.11
0.94
2.00
2.01
2.03
2.15
2.05
4.09
10.0 9.5 9.0 8.5 8.0 7.5 7.0 6.5 6.0 5.5 5.0 4.5 4.0 3.5 3.0 2.5 2.0 1.5 1.0 0.5 0.0
[ppm]

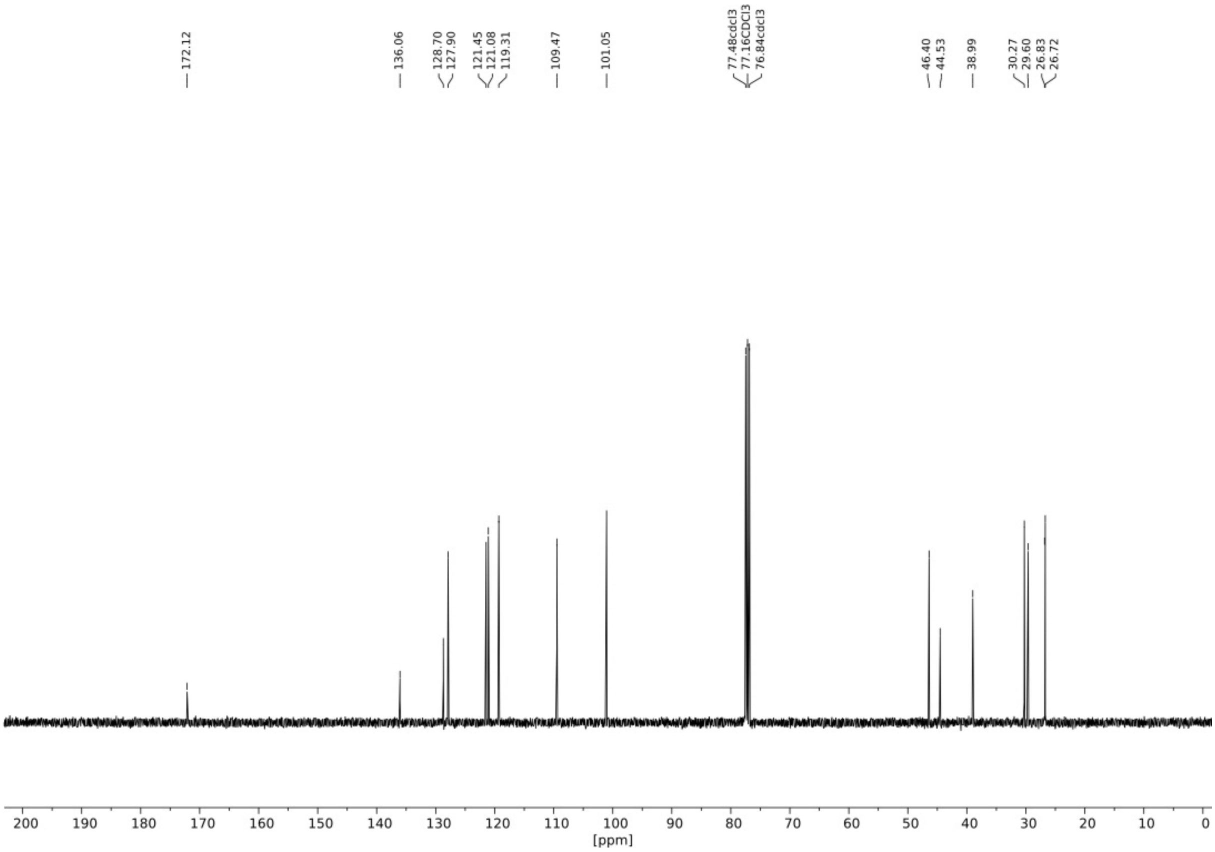
172.12
136.06
128.70
127.90
121.45
121.08
119.31
109.47
101.05
77.48cdcl3
77.16CDCl3
76.84cdcl3
46.40
44.53
38.99
30.27
29.60
26.83
26.72
200 190 180 170 160 150 140 130 120 110 100 90 80 70 60 50 40 30 20 10 0
[ppm]

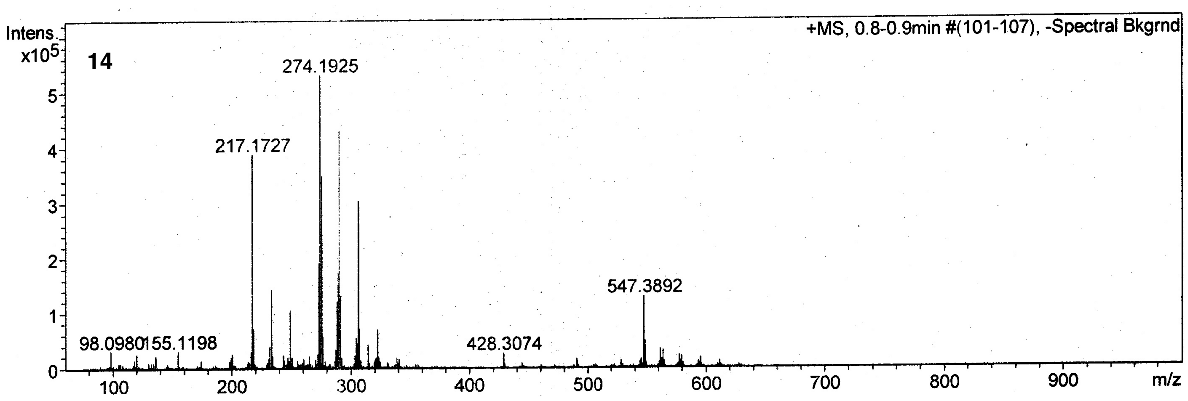
Intens.
x10^5
14
+MS, 0.8-0.9min #(101-107), -Spectral Bkgrnd
274.1925
217.1727
547.3892
98.0980
155.1198
428.3074
m/z

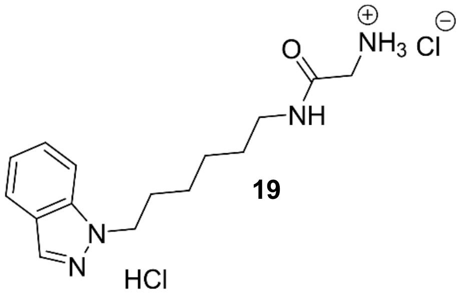
O
NH3 Cl
NH
N
N
19
HCl

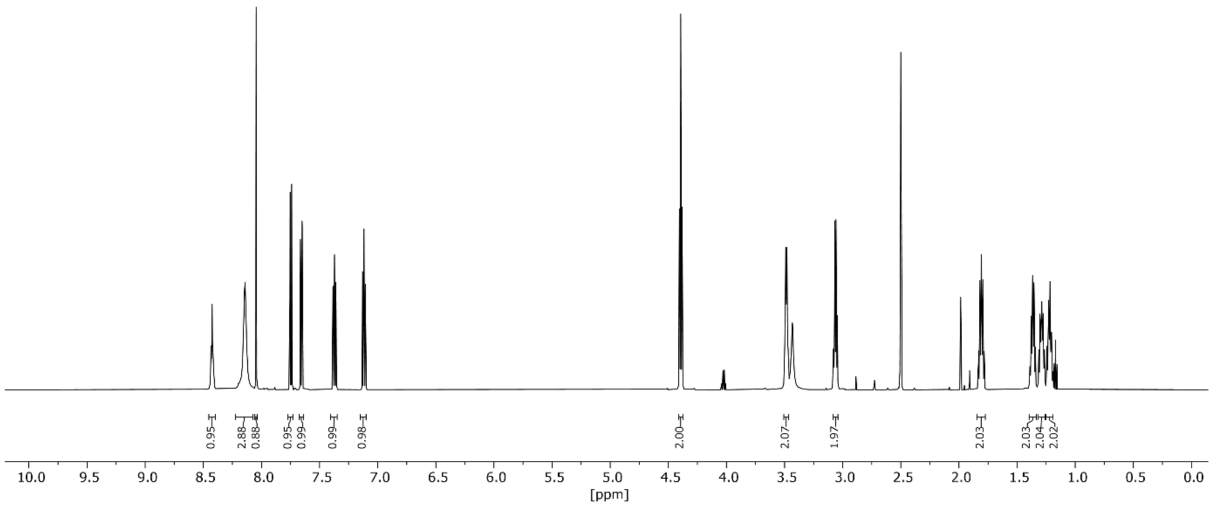
0.95
2.88
0.88
0.95
0.99
0.99
0.98
2.00
2.07
1.97
2.03
2.03
2.04
2.02
10.0 9.5 9.0 8.5 8.0 7.5 7.0 6.5 6.0 5.5 5.0 4.5 4.0 3.5 3.0 2.5 2.0 1.5 1.0 0.5 0.0
[ppm]

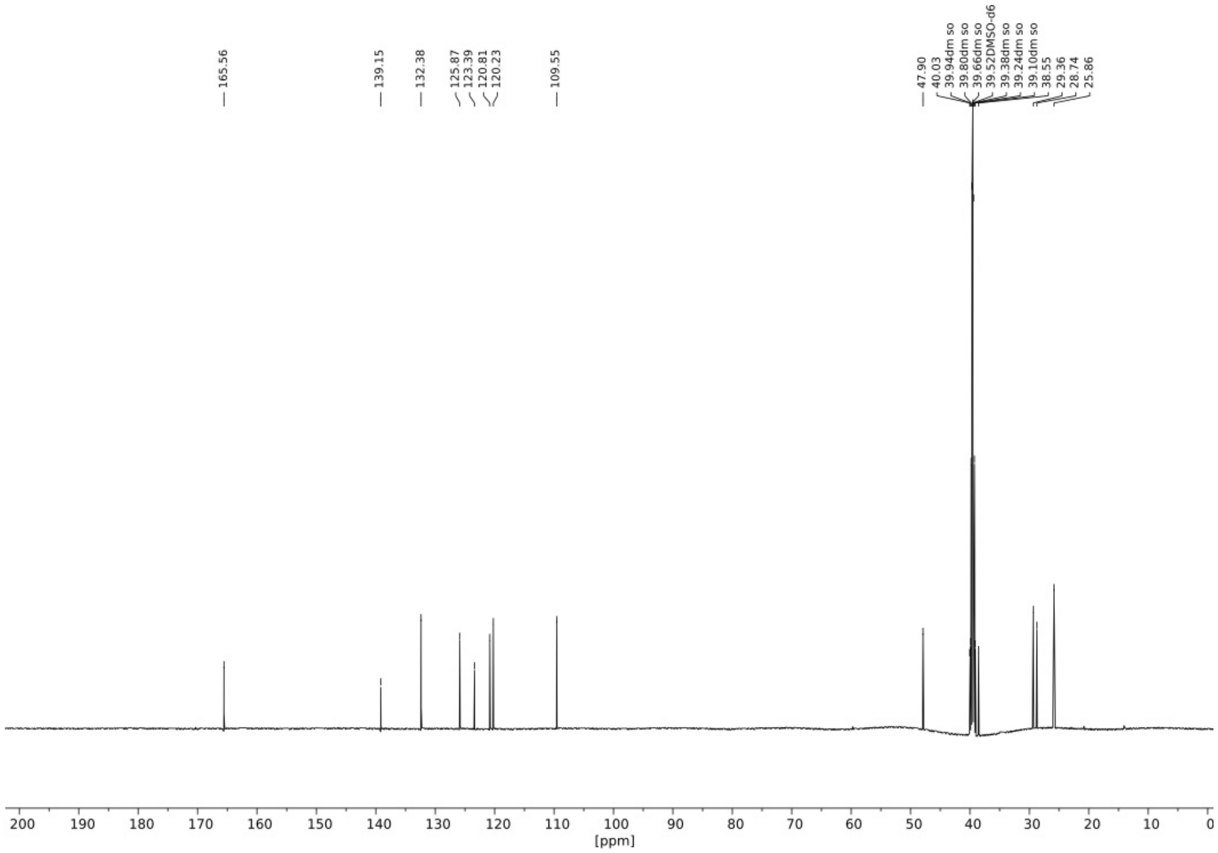
165.56
139.15
132.38
125.87
123.39
120.81
120.23
109.55
47.90
40.03
39.94dm so
39.80dm so
39.66dm so
39.52DMSO-d6
39.38dm so
39.24dm so
39.10dm so
38.55
29.36
28.74
25.86
200 190 180 170 160 150 140 130 120 110 100 90 80 70 60 50 40 30 20 10 0
[ppm]

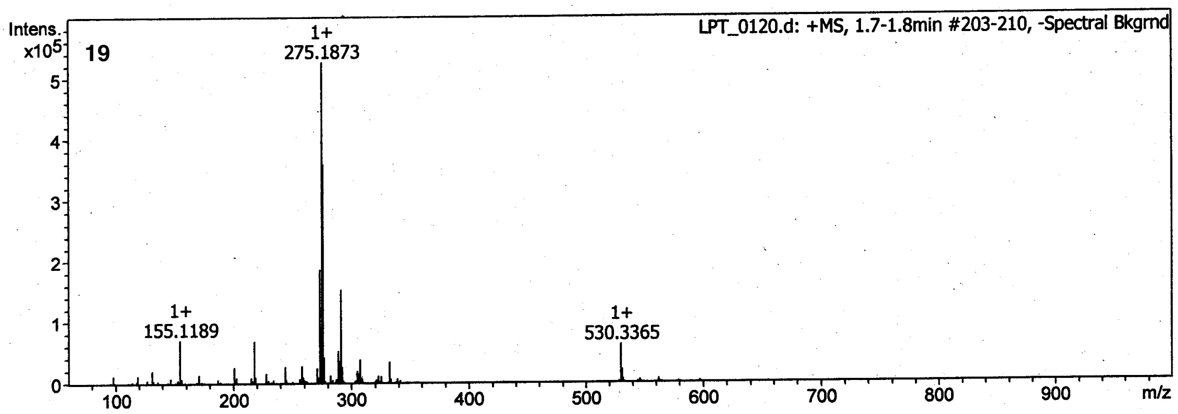
Intens.
x10^5
19
1+
275.1873
LPT_0120.d: +MS, 1.7-1.8min #203-210, -Spectral Bkgrnd
1+
155.1189
1+
530.3365
m/z

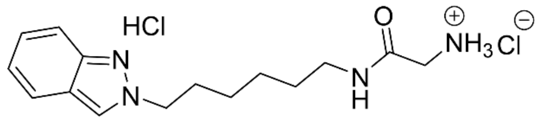
HCl
N
N
O
NH3
Cl
N
H

22

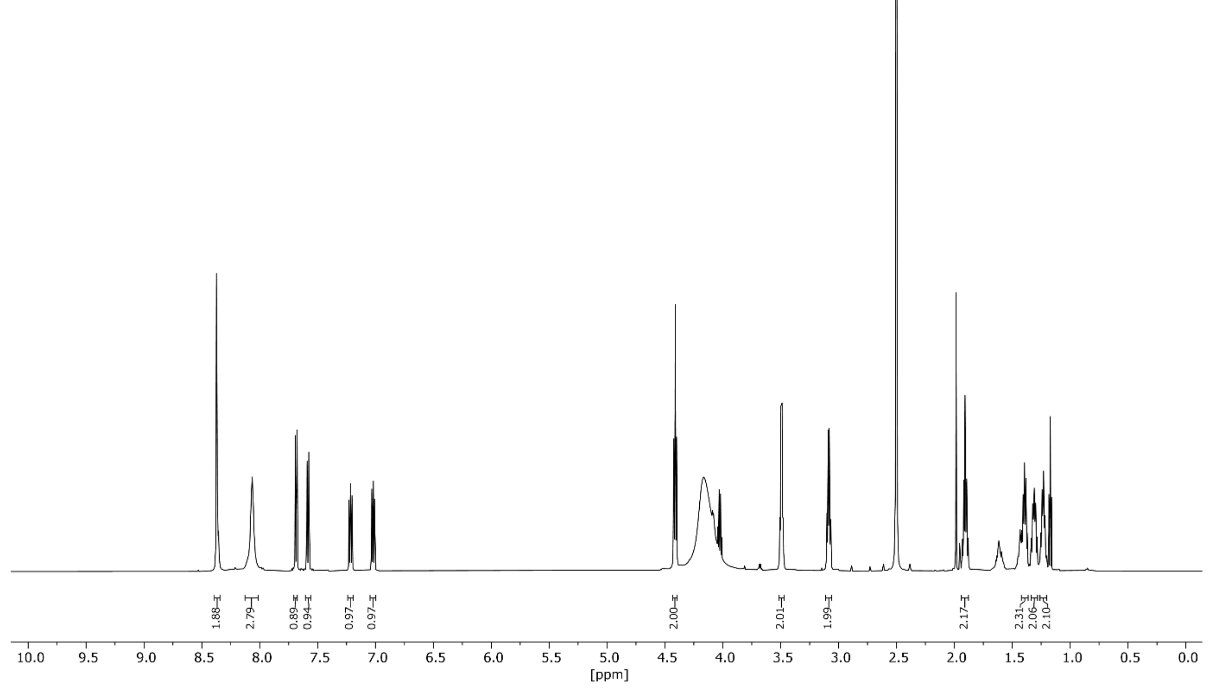
1.88
2.79
0.89
0.94
0.97
0.97
2.00
2.01
1.99
2.17
2.31
2.06
2.10
[ppm]

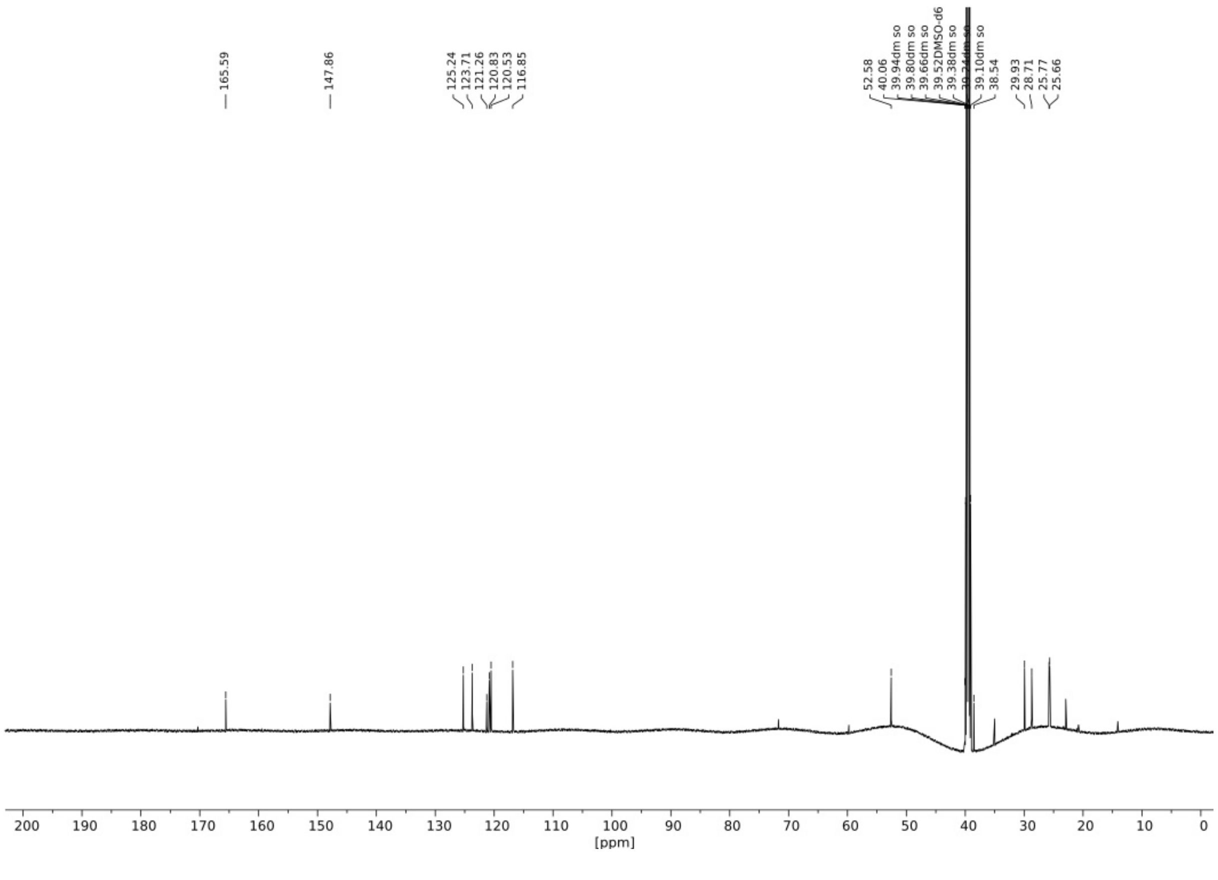
165.59
147.86
125.24
123.71
121.26
120.83
120.53
116.85
52.58
40.06
39.94dm so
39.80dm so
39.66dm so
39.52DMSO-d6
39.38dm so
39.10dm so
38.54
29.93
28.71
25.77
25.66
[ppm]

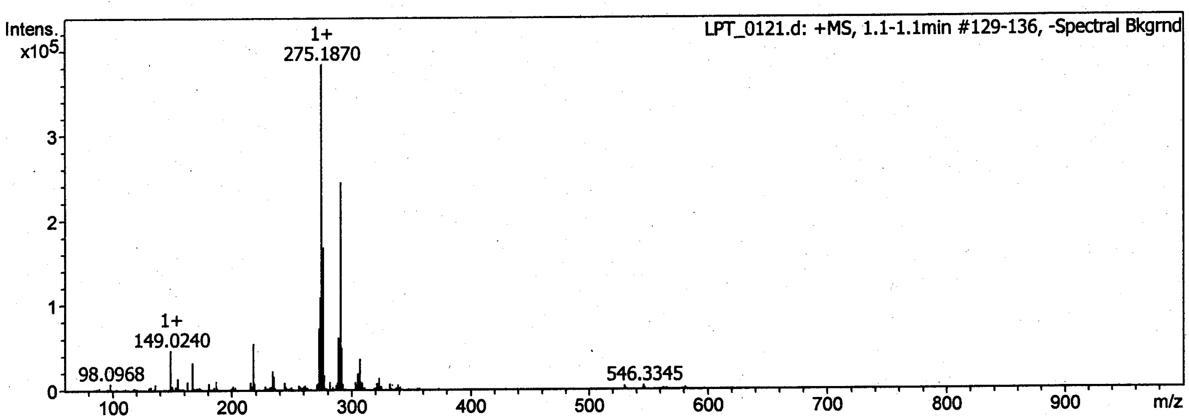
Intens.
x10^5
LPT_0121.d: +MS, 1.1-1.1min #129-136, -Spectral Bkgrnd
1+
275.1870
1+
149.0240
98.0968
546.3345
m/z

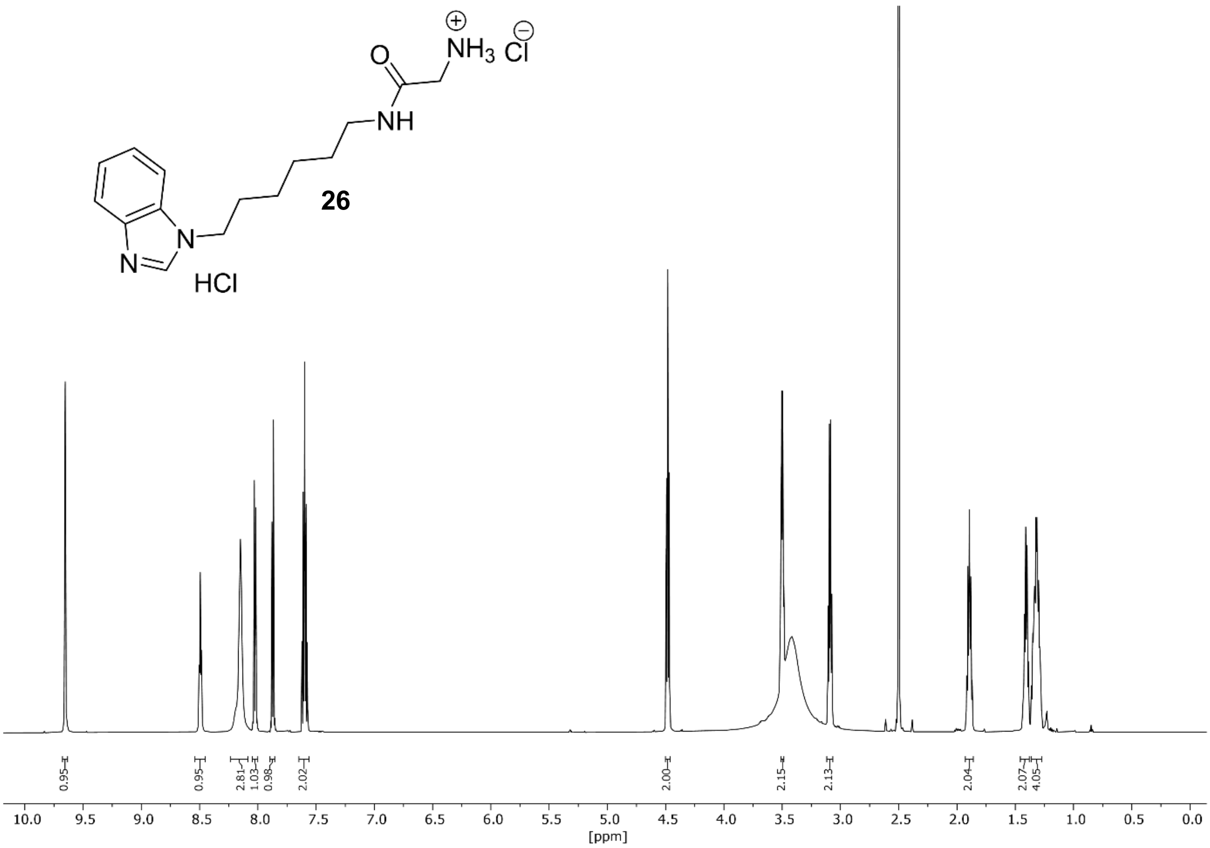
26
HCl
[ppm]

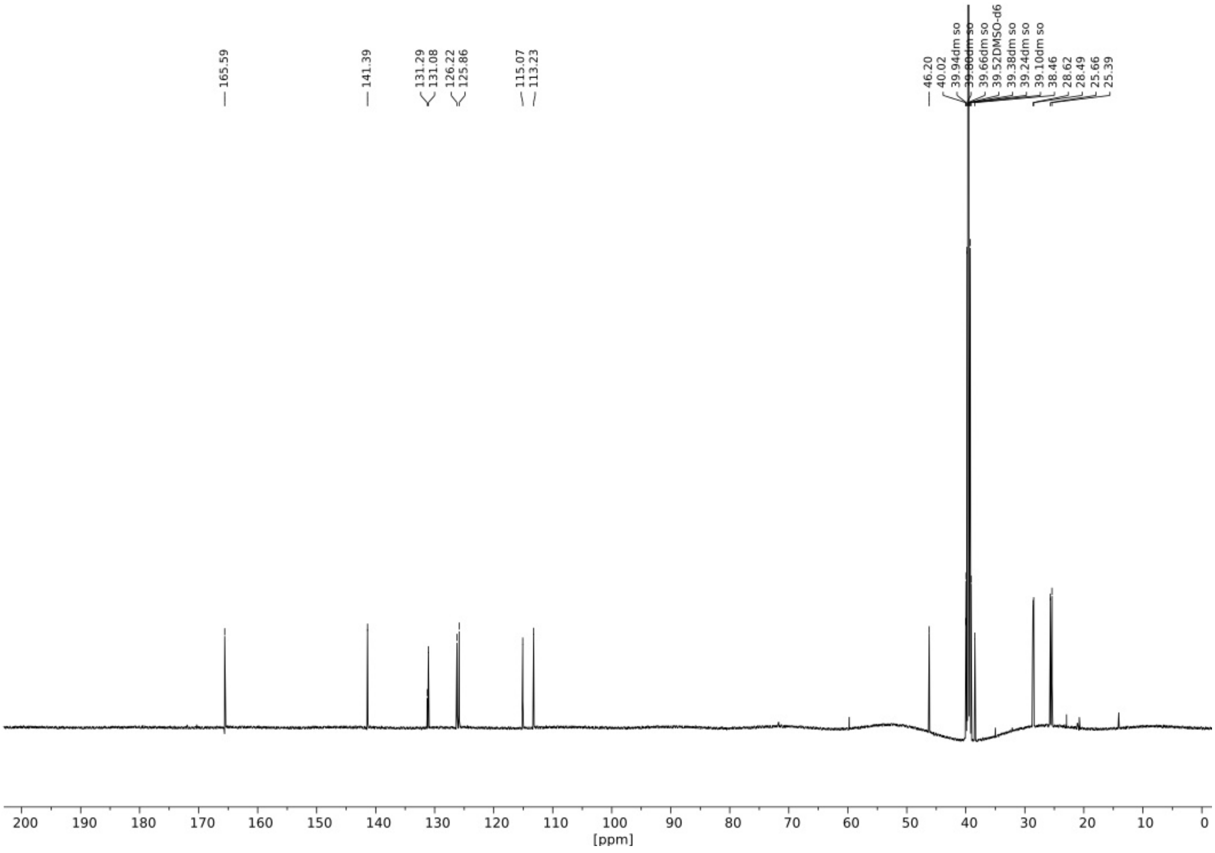
165.59
141.39
131.29
131.08
126.22
125.86
115.07
113.23
46.20
40.02
39.94dm so
39.80dm so
39.66dm so
39.52DMSO-d6
39.38dm so
39.24dm so
39.10dm so
38.46
28.62
28.49
25.66
25.39
[ppm]

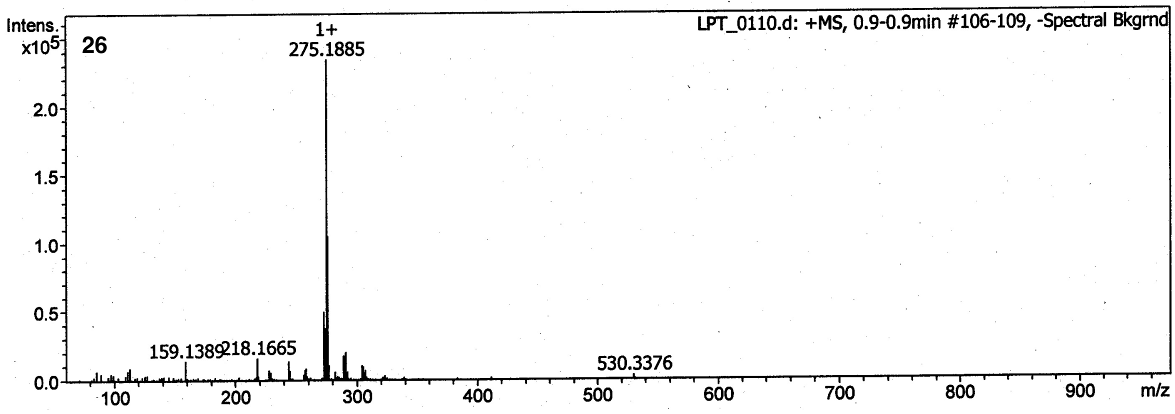
Intens.
x10^5
26
1+
275.1885
159.1389
218.1665
530.3376
LPT_0110.d: +MS, 0.9-0.9min #106-109, -Spectral Bkgrnd
2.0
1.5
1.0
0.5
0.0
100
200
300
400
500
600
700
800
900
m/z

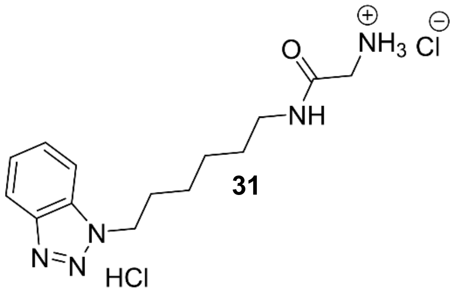
NH3
Cl
NH
O
N
N=N
HCl
31

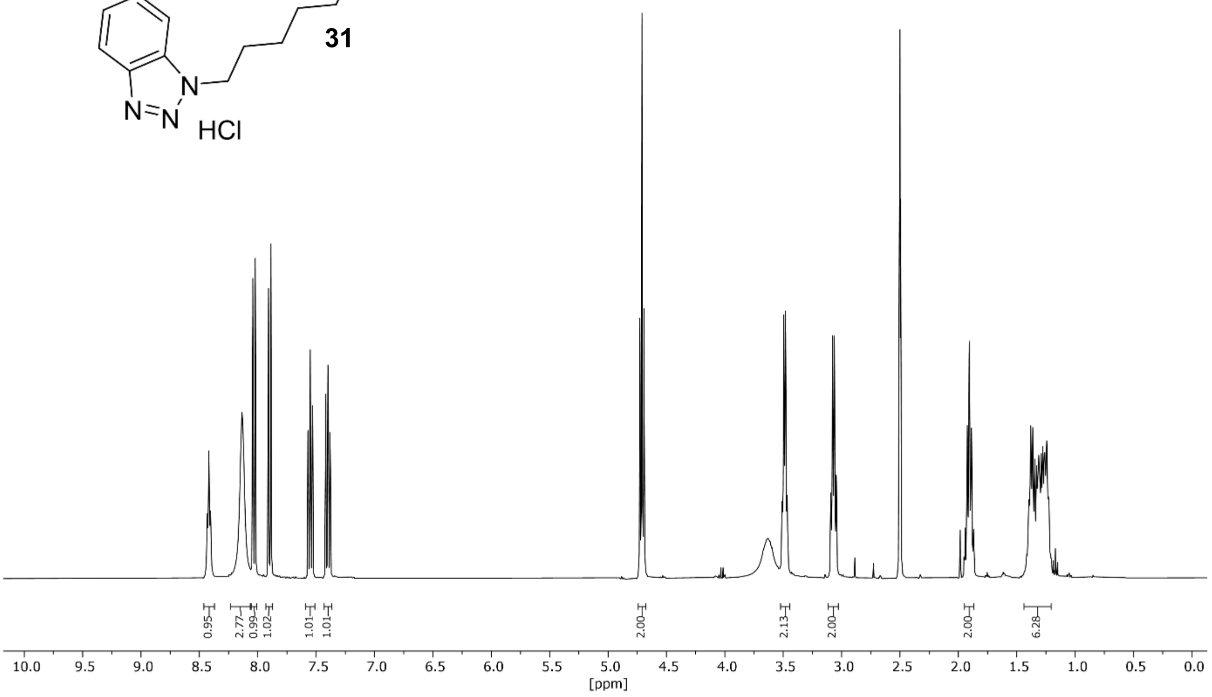
0.95
2.77
0.99
1.02
1.01
1.01
2.00
2.13
2.00
2.00
6.28
10.0 9.5 9.0 8.5 8.0 7.5 7.0 6.5 6.0 5.5 5.0 4.5 4.0 3.5 3.0 2.5 2.0 1.5 1.0 0.5 0.0
[ppm]

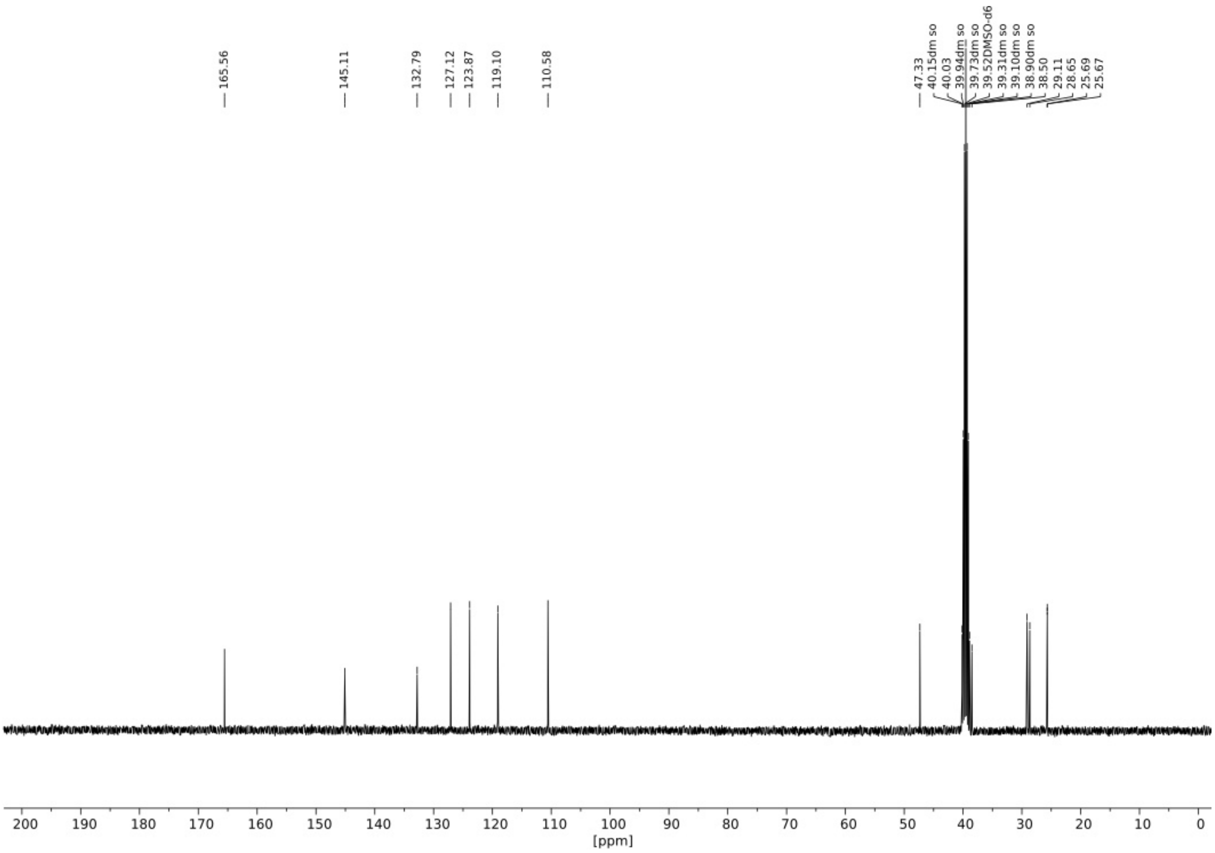
165.56
145.11
132.79
127.12
123.87
119.10
110.58
47.33
40.15dm so
40.03
39.94dm so
39.73dm so
39.52DMSO-d6
39.31dm so
39.10dm so
38.90dm so
38.50
29.11
28.65
25.69
25.67
200 190 180 170 160 150 140 130 120 110 100 90 80 70 60 50 40 30 20 10 0
[ppm]

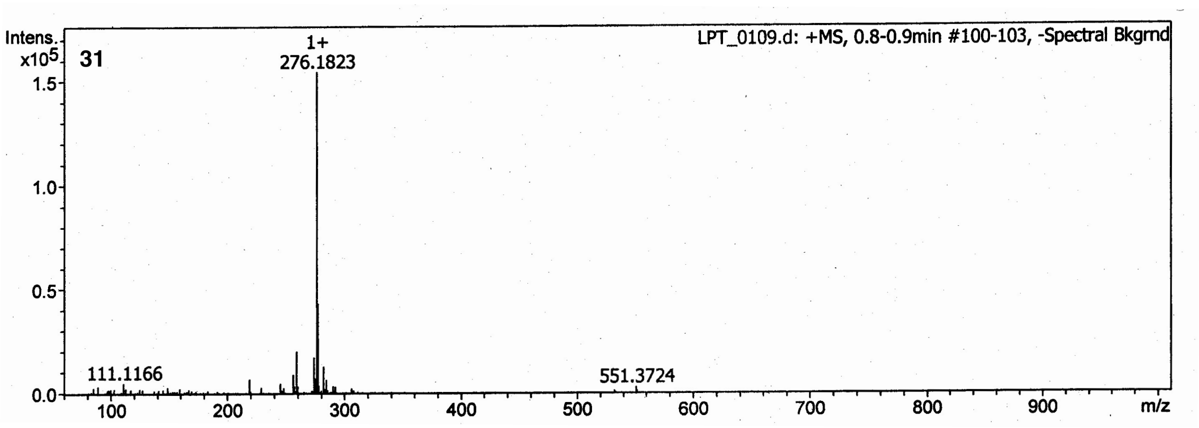
Intens.
x10^5
31
1+
276.1823
LPT_0109.d: +MS, 0.8-0.9min #100-103, -Spectral Bkgrnd
1.5
1.0
0.5
0.0
111.1166
551.3724
100
200
300
400
500
600
700
800
900
m/z

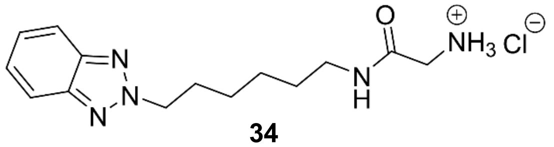

34

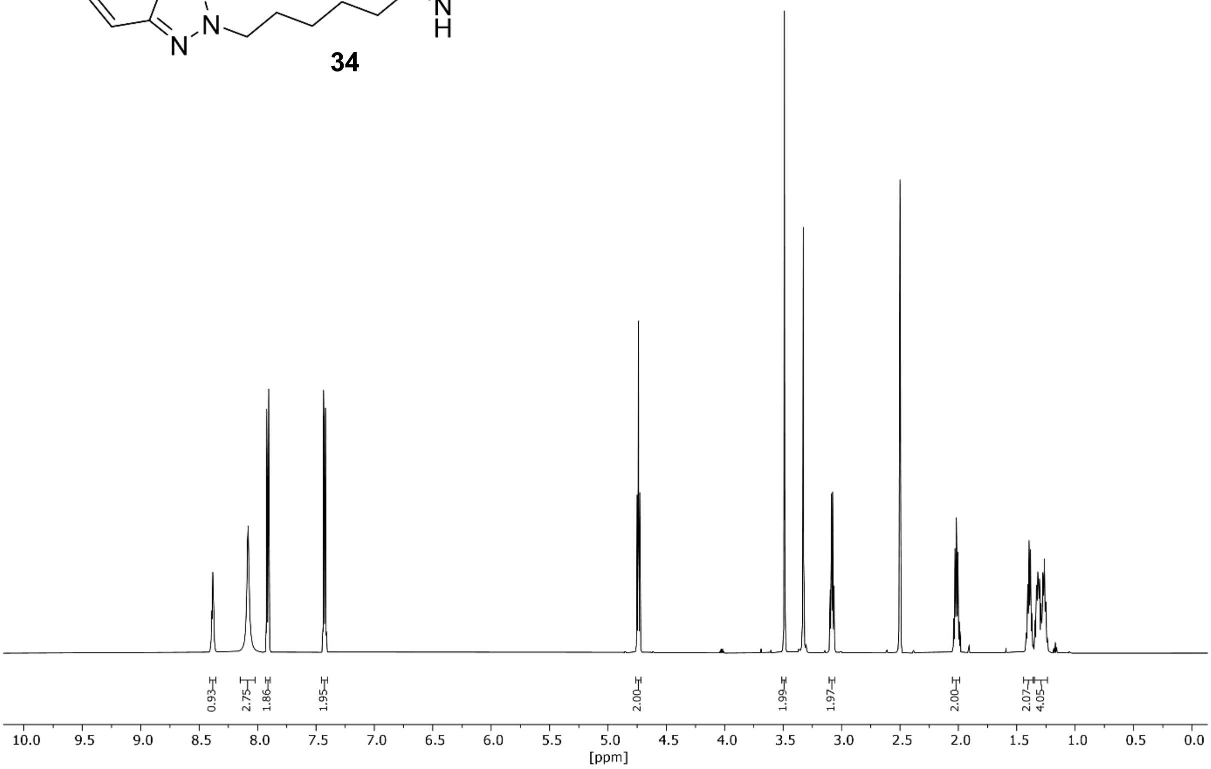

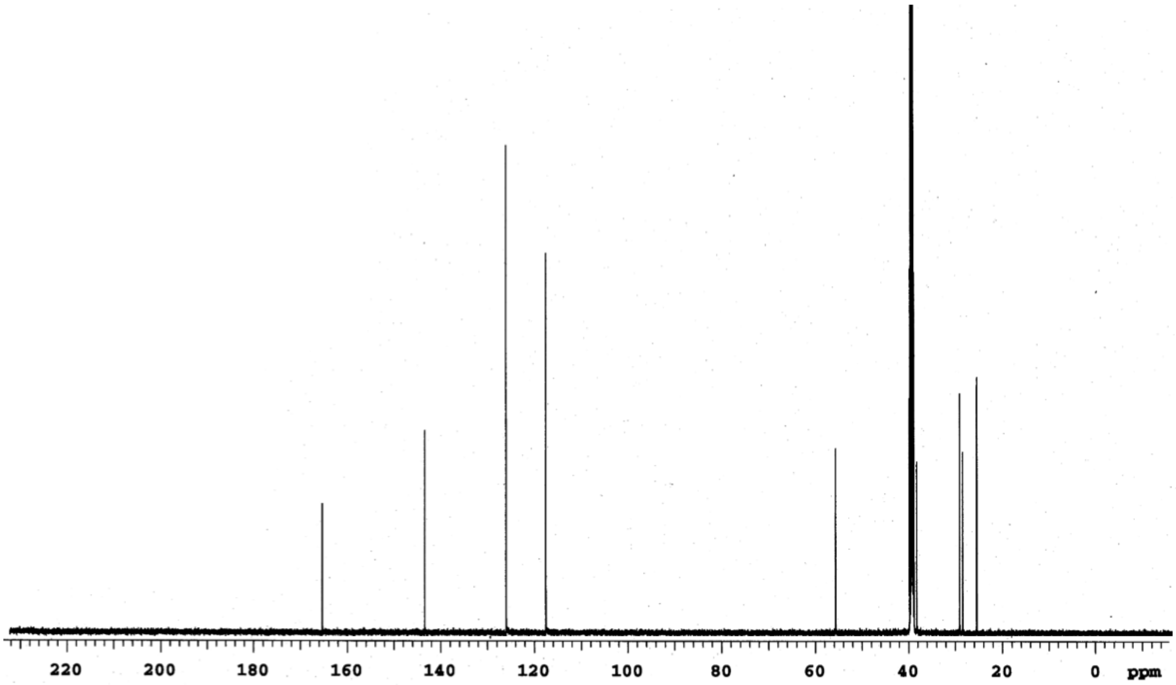

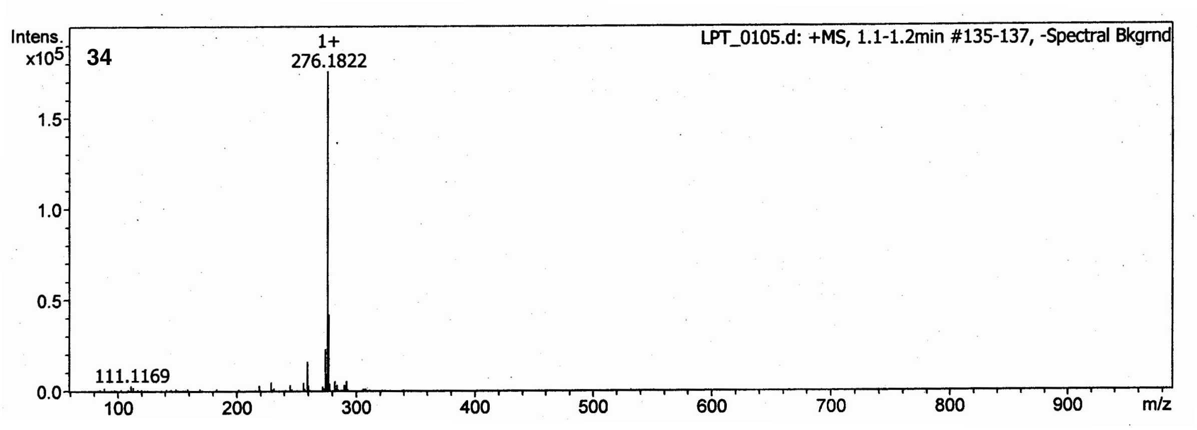
Intens.
x10^5
34
1+
276.1822
LPT_0105.d: +MS, 1.1-1.2min #135-137, -Spectral Bkgrnd
1.5
1.0
0.5
0.0
111.1169
100
200
300
400
500
600
700
800
900
m/z

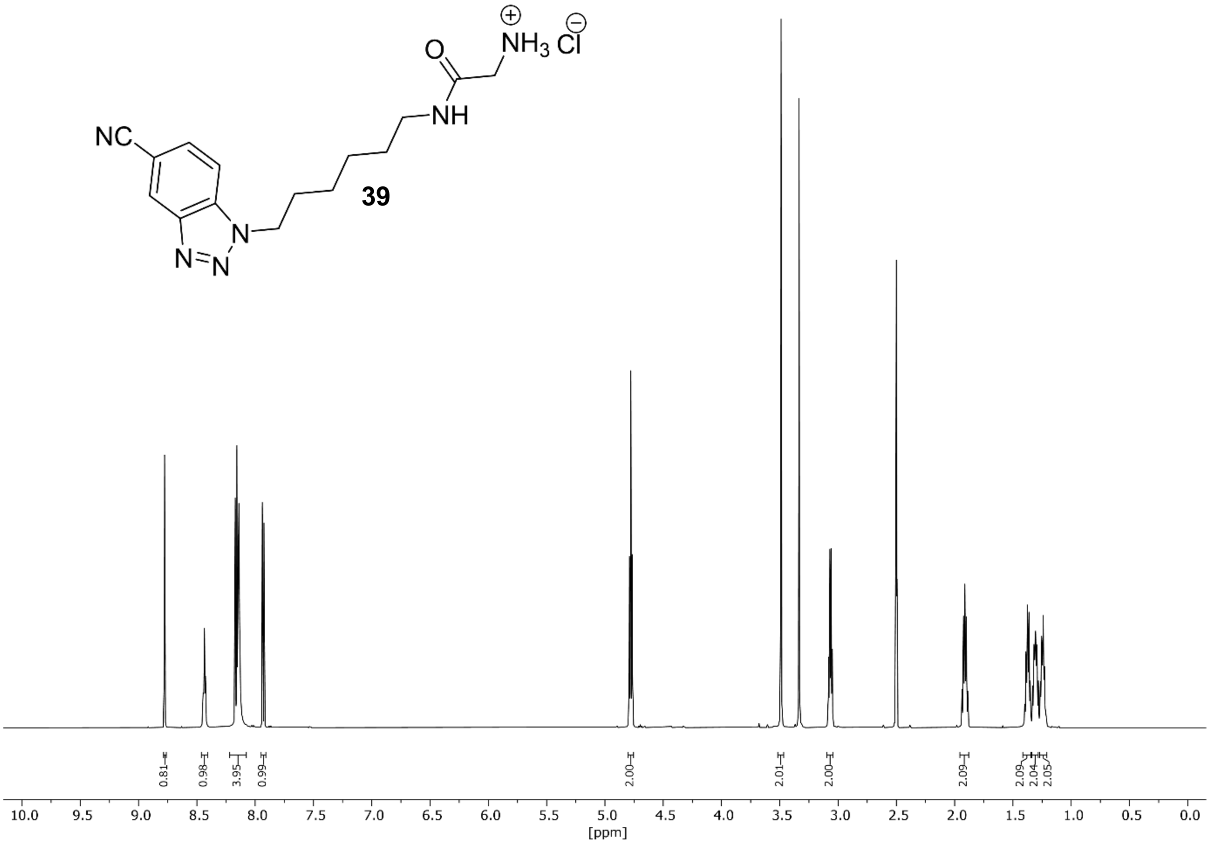
NC
N
N
N
NH
O
NH3 Cl
0.81
0.98
3.95
0.99
2.00
2.01
2.00
2.09
2.09
2.04
2.05
[ppm]

39

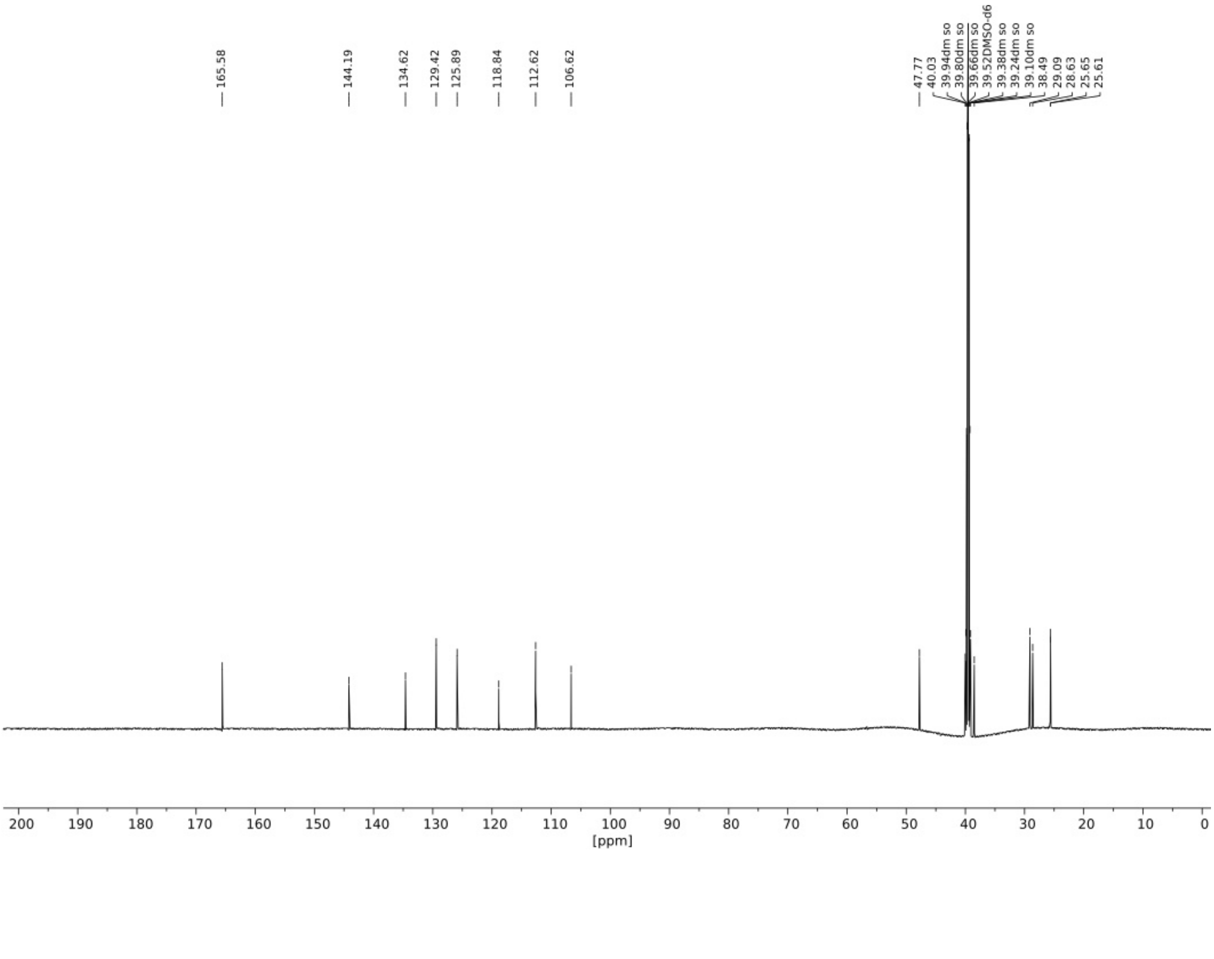
165.58
144.19
134.62
129.42
125.89
118.84
112.62
106.62
47.77
40.03
39.94dm so
39.80dm so
39.66dm so
39.52DMSO-d6
39.38dm so
39.24dm so
39.10dm so
38.49
29.09
28.63
25.65
25.61
[ppm]

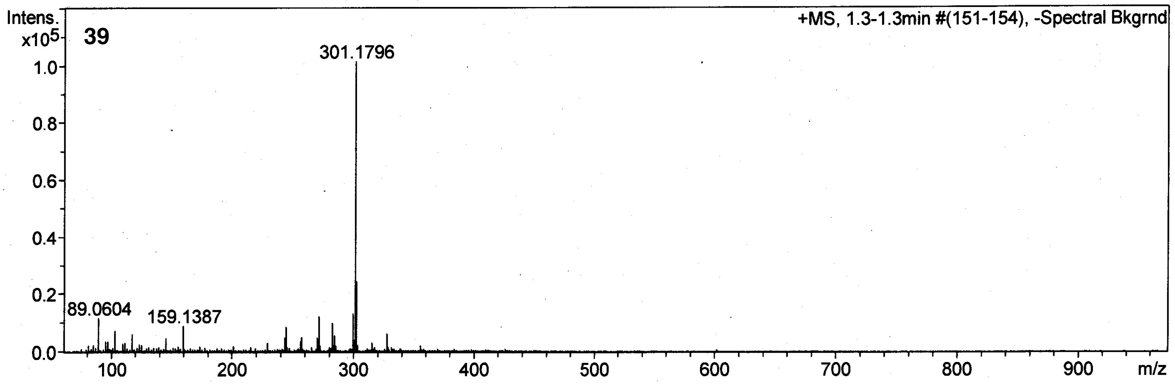
Intens.
x10^5
39
+MS, 1.3-1.3min #(151-154), -Spectral Bkgrnd
301.1796
89.0604
159.1387
1.0
0.8
0.6
0.4
0.2
0.0
100
200
300
400
500
600
700
800
900
m/z

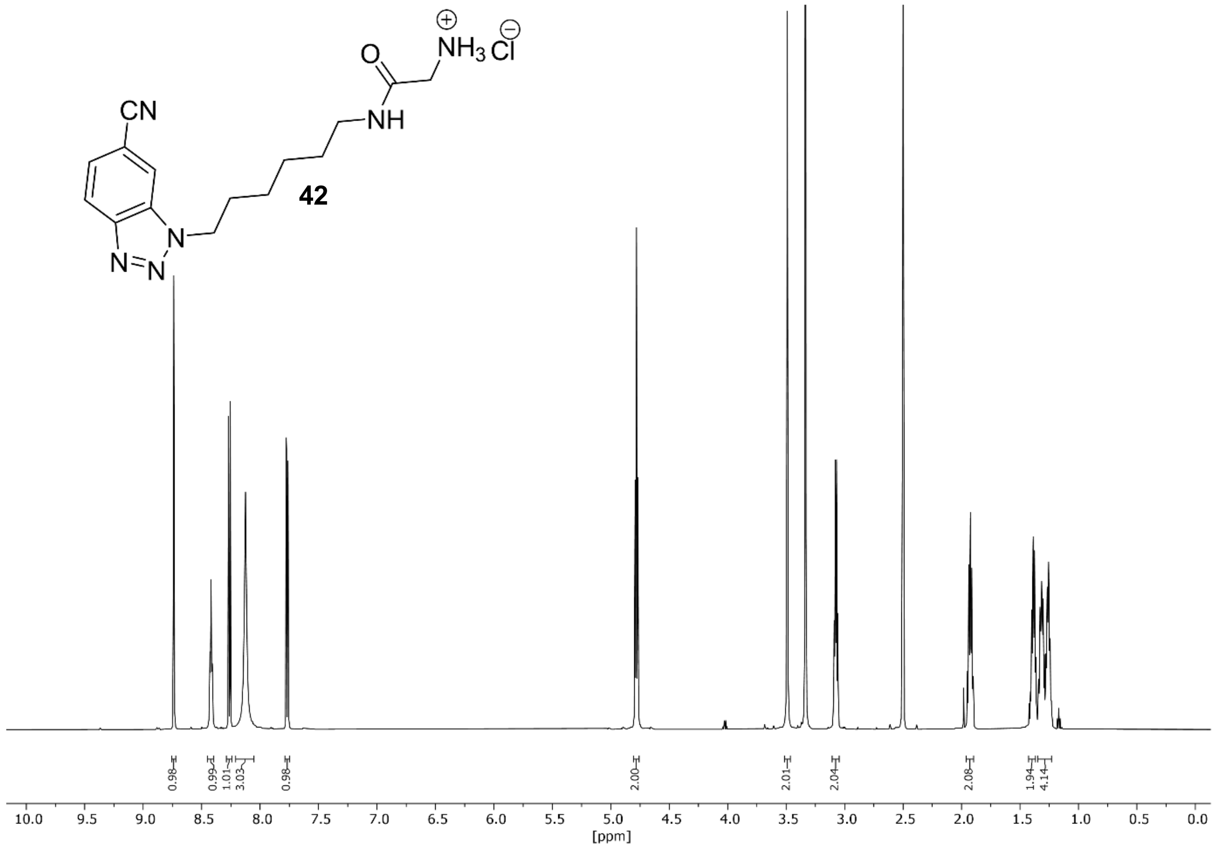
CN
N
N=N
O
NH
NH3 Cl
42
0.98
0.99
1.01
3.03
0.98
2.00
2.01
2.04
2.08
1.94
4.14
[ppm]

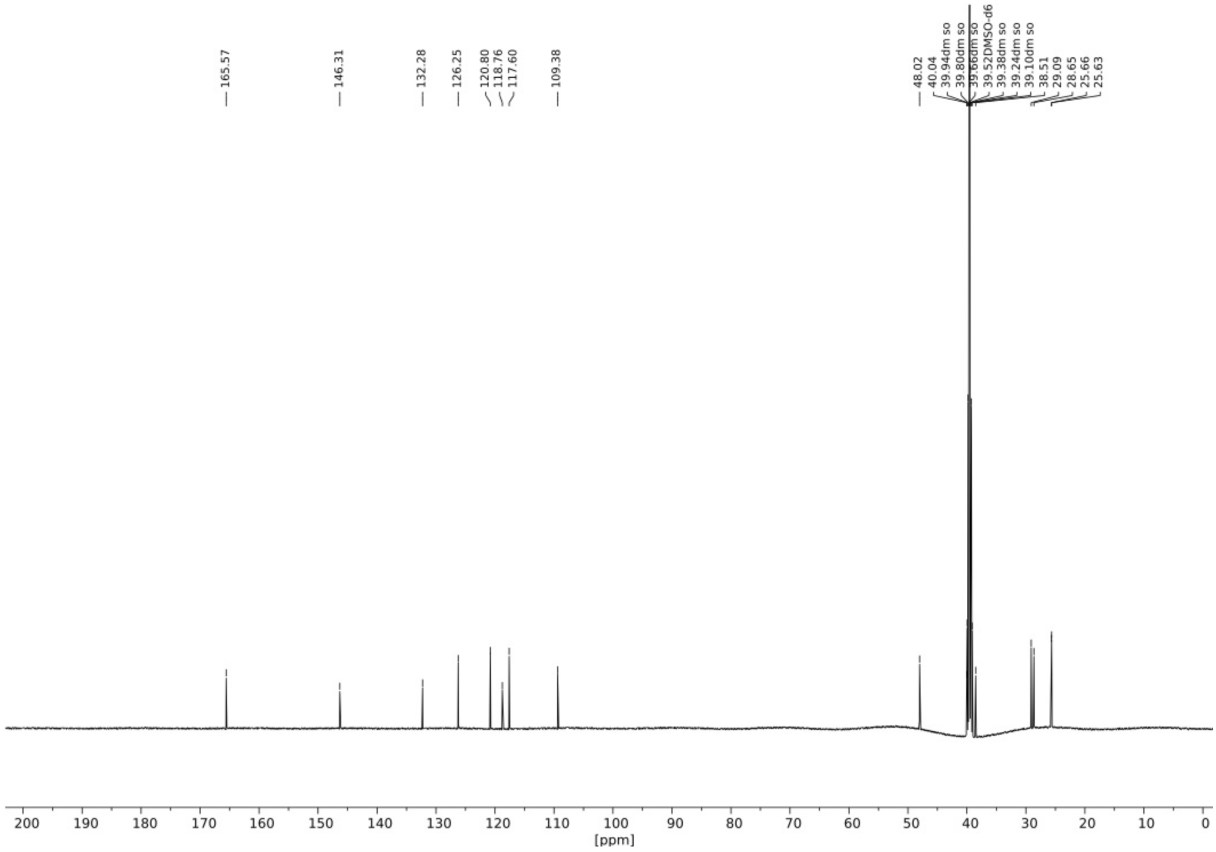
165.57
146.31
132.28
126.25
120.80
118.76
117.60
109.38
48.02
40.04
39.94dm so
39.80dm so
39.66dm so
39.52DMSO-d6
39.38dm so
39.24dm so
39.10dm so
38.51
29.09
28.65
25.66
25.63
[ppm]

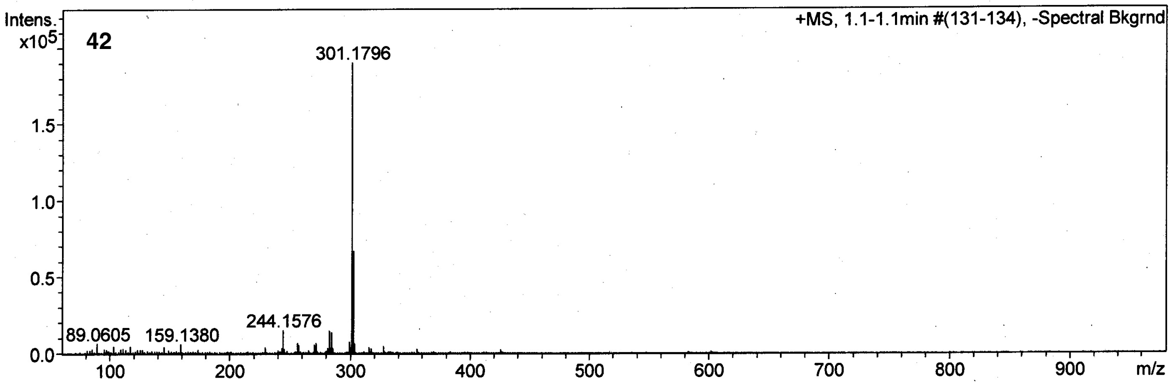
Intens.
x10^5
42
+MS, 1.1-1.1min #(131-134), -Spectral Bkgrnd
301.1796
244.1576
89.0605
159.1380
1.5
1.0
0.5
0.0
100
200
300
400
500
600
700
800
900
m/z

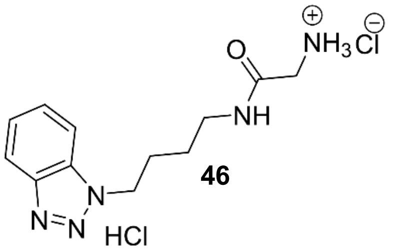

46

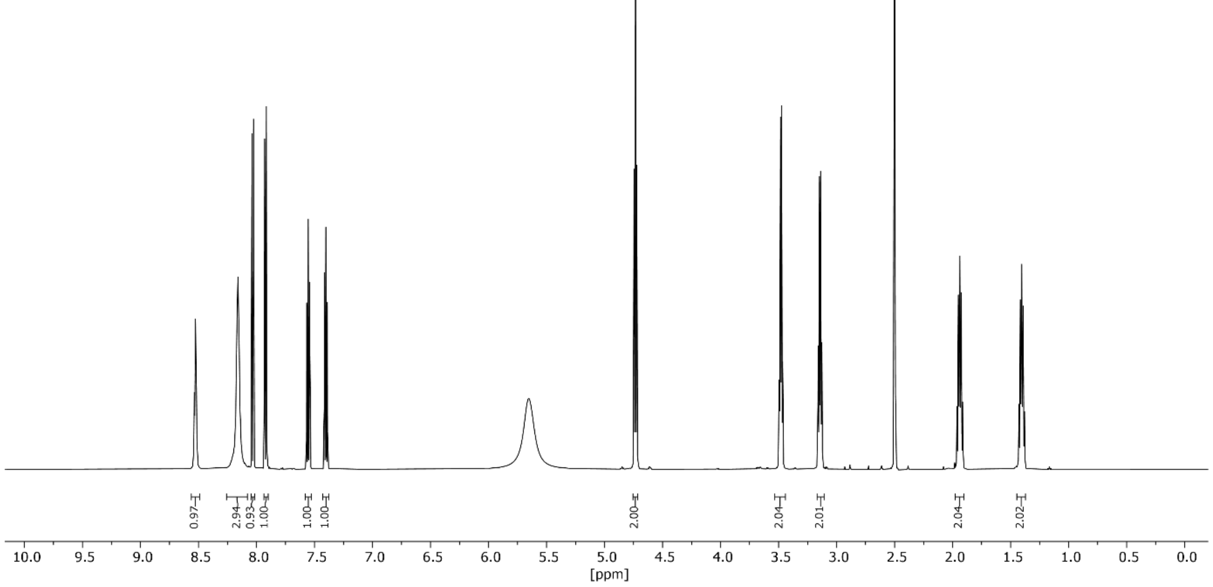
0.97
2.94
0.93
1.00
1.00
1.00
2.00
2.04
2.01
2.04
2.02
10.0 9.5 9.0 8.5 8.0 7.5 7.0 6.5 6.0 5.5 5.0 4.5 4.0 3.5 3.0 2.5 2.0 1.5 1.0 0.5 0.0
[ppm]

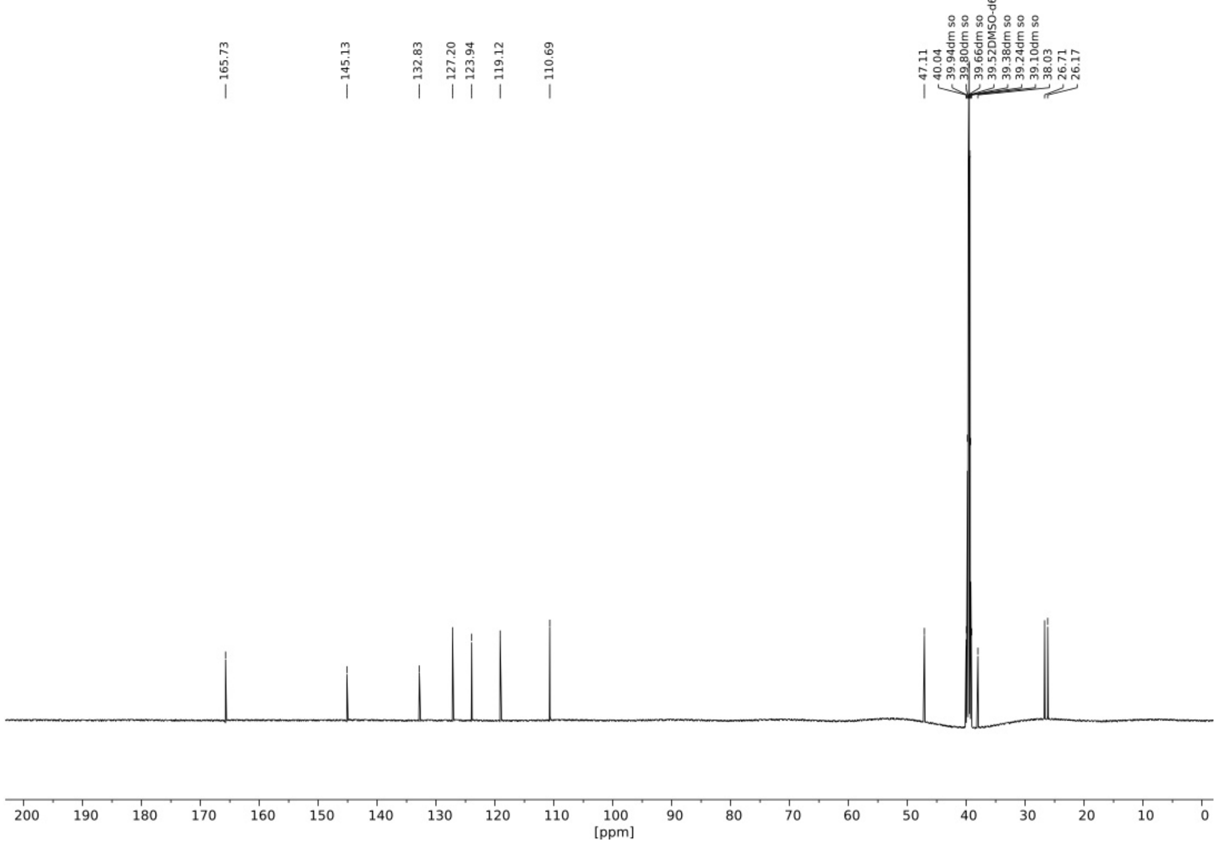
165.73
145.13
132.83
127.20
123.94
119.12
110.69
47.11
40.04
39.94dm so
39.80dm so
39.66dm so
39.52DMSO-d6
39.38dm so
39.24dm so
39.10dm so
38.03
26.71
26.17
200 190 180 170 160 150 140 130 120 110 100 90 80 70 60 50 40 30 20 10 0
[ppm]

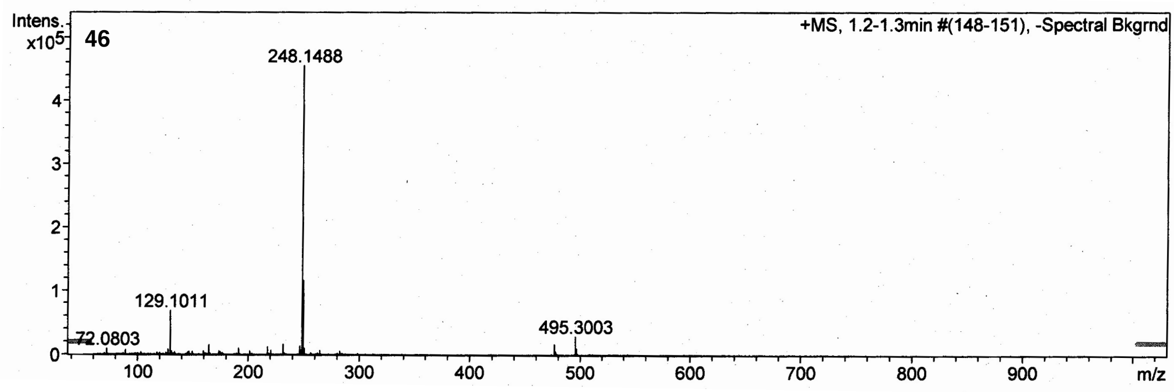
Intens.
x10^5
46
+MS, 1.2-1.3min #(148-151), -Spectral Bkgrnd
248.1488
129.1011
72.0803
495.3003
m/z

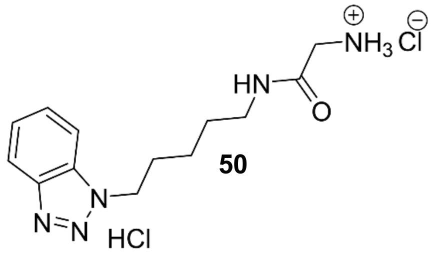
NH3 Cl
HN
O
N
N=N
HCl

50

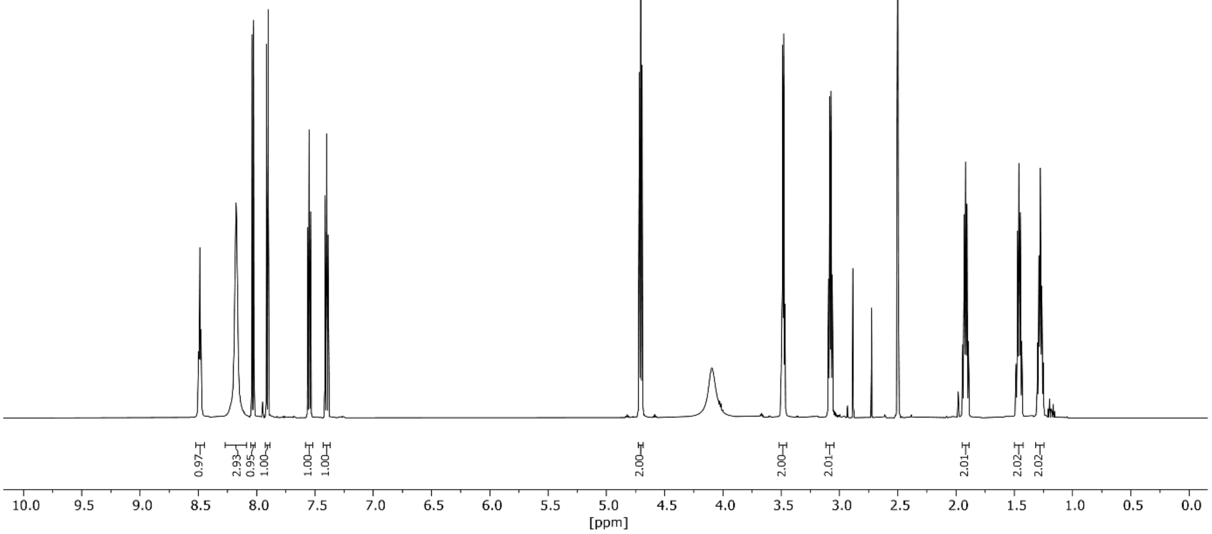
0.97
2.93
0.95
1.00
1.00
1.00
2.00
2.00
2.01
2.01
2.02
2.02
10.0 9.5 9.0 8.5 8.0 7.5 7.0 6.5 6.0 5.5 5.0 4.5 4.0 3.5 3.0 2.5 2.0 1.5 1.0 0.5 0.0
[ppm]

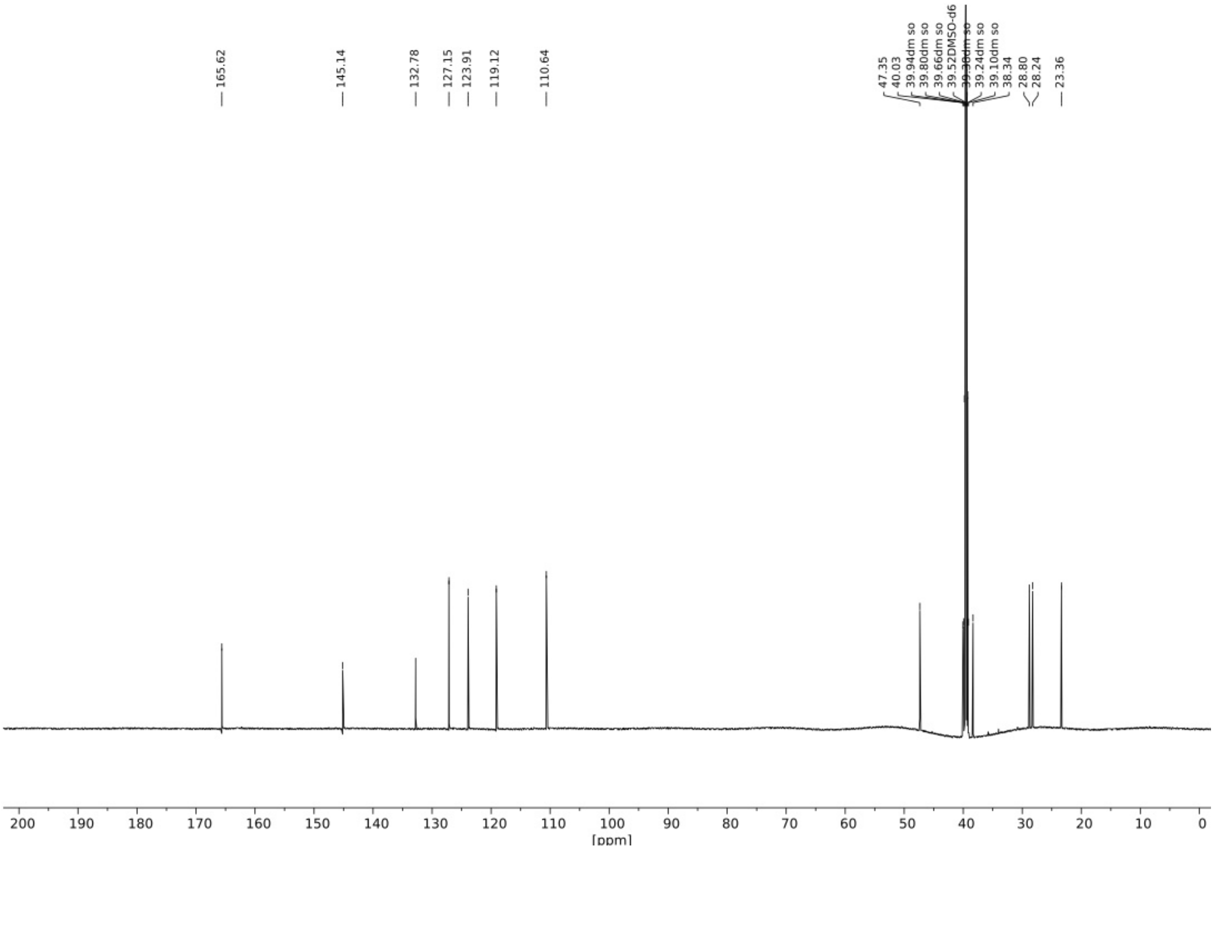
165.62
145.14
132.78
127.15
123.91
119.12
110.64
47.35
40.03
39.94dm so
39.80dm so
39.66dm so
39.52DMSO-d6
39.38dm so
39.24dm so
39.10dm so
38.34
28.80
28.24
23.36
200 190 180 170 160 150 140 130 120 110 100 90 80 70 60 50 40 30 20 10 0
[ppm]

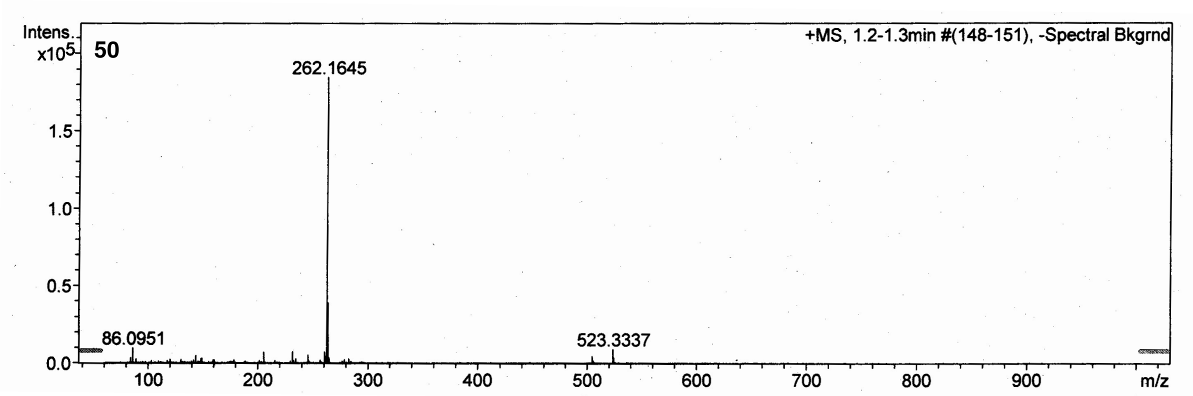
Intens.
x10^5
50
+MS, 1.2-1.3min #(148-151), -Spectral Bkgrnd
262.1645
86.0951
523.3337
1.5
1.0
0.5
0.0
100
200
300
400
500
600
700
800
900
m/z

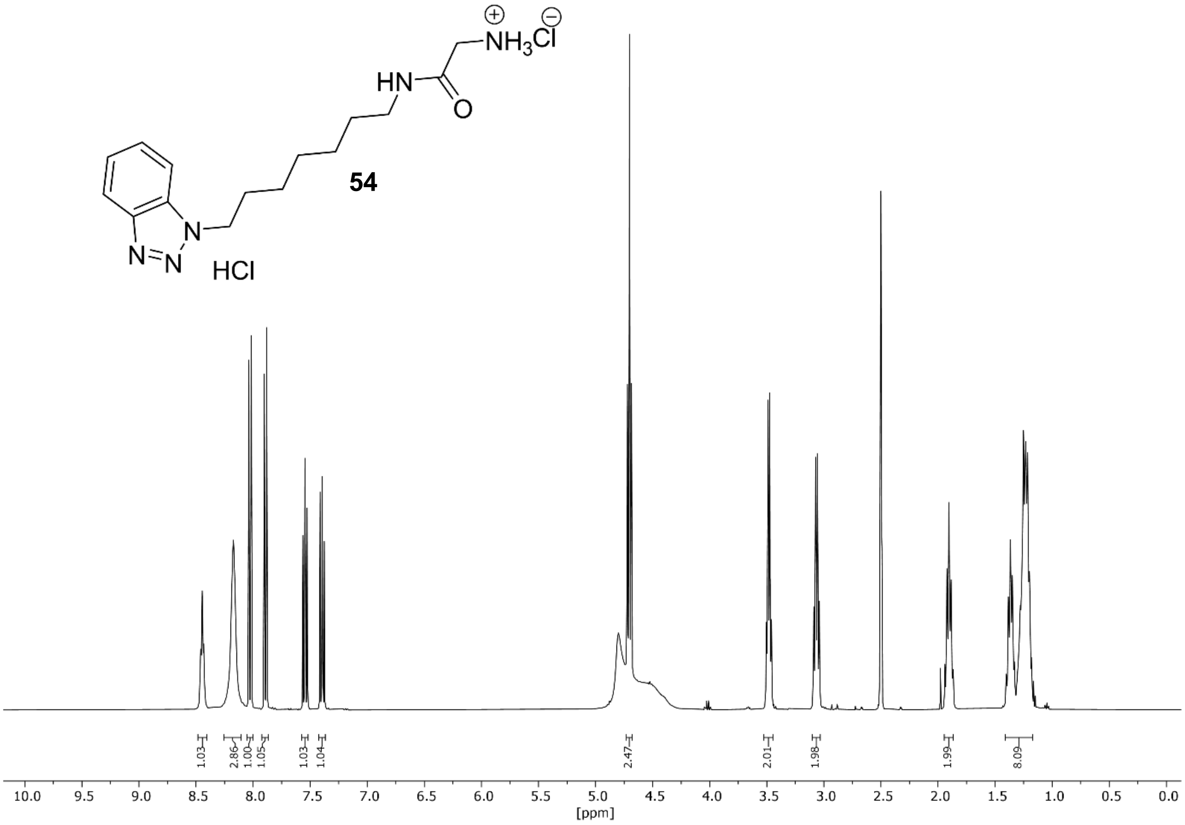
54
HCl
1.03
2.86
1.00
1.05
1.03
1.04
2.47
2.01
1.98
1.99
8.09
10.0 9.5 9.0 8.5 8.0 7.5 7.0 6.5 6.0 5.5 5.0 4.5 4.0 3.5 3.0 2.5 2.0 1.5 1.0 0.5 0.0
[ppm]

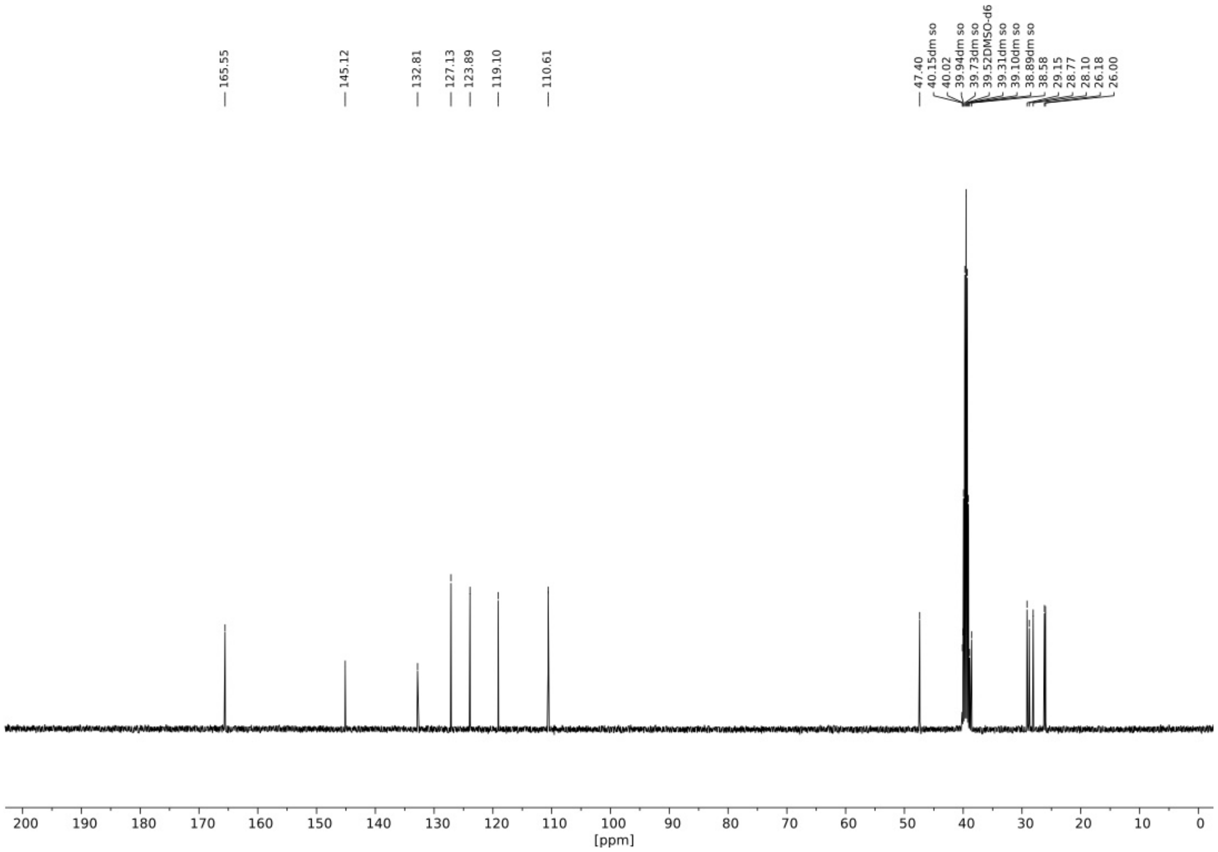
165.55
145.12
132.81
127.13
123.89
119.10
110.61
47.40
40.15dm so
40.02
39.94dm so
39.73dm so
39.52DMSO-d6
39.31dm so
39.10dm so
38.89dm so
38.58
29.15
28.77
28.10
26.18
26.00
200 190 180 170 160 150 140 130 120 110 100 90 80 70 60 50 40 30 20 10 0
[ppm]

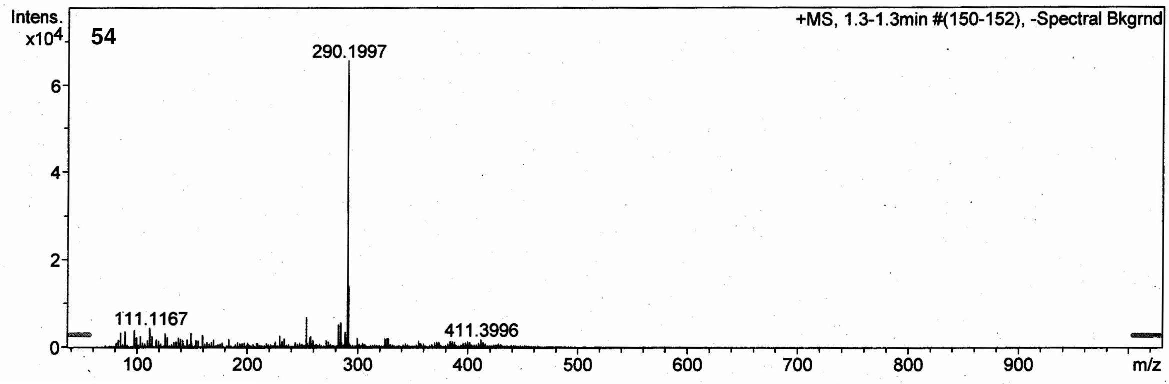
Intens.
x10^4
54
+MS, 1.3-1.3min #(150-152), -Spectral Bkgrnd
290.1997
111.1167
411.3996
0
2
4
6
100
200
300
400
500
600
700
800
900
m/z

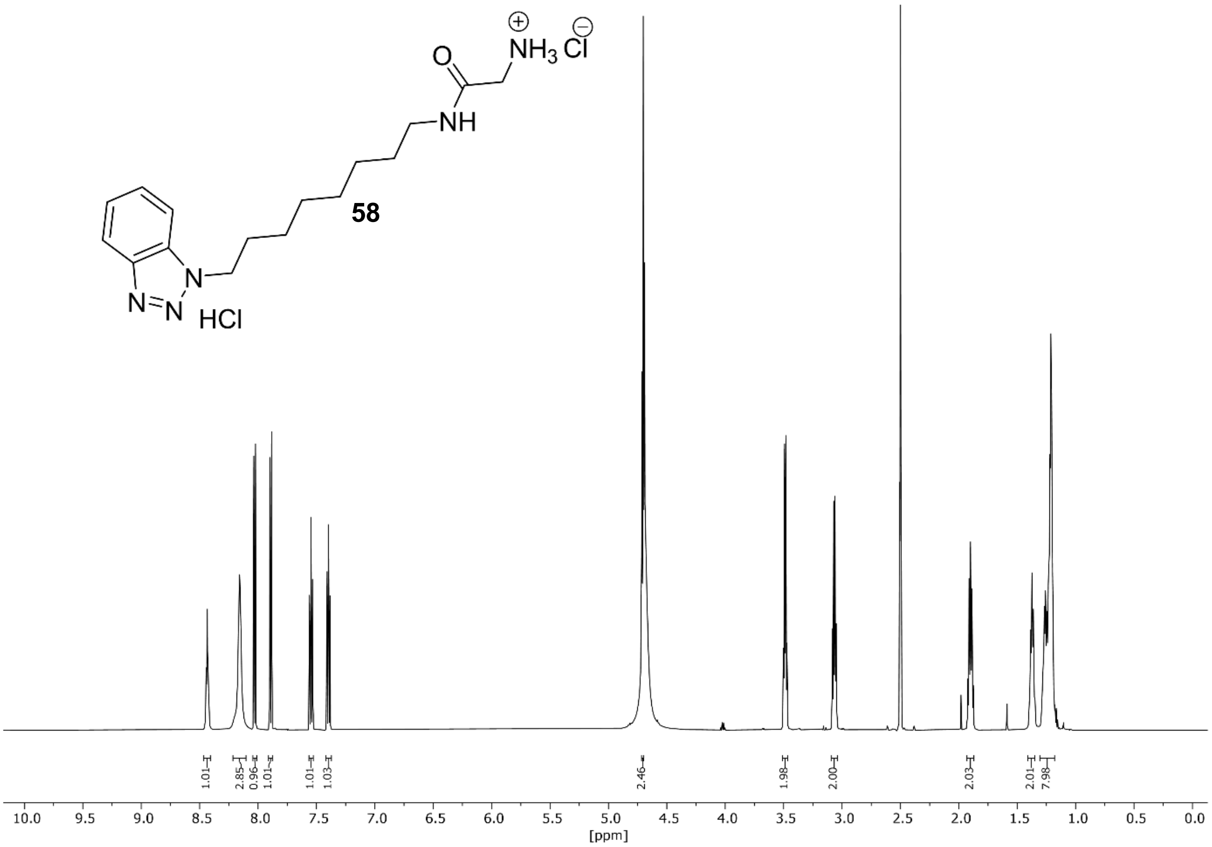
NH3 Cl
O
NH
58
N
N=N
HCl
1.01
2.85
0.96
1.01
1.01
1.03
2.46
1.98
2.00
2.03
2.01
7.98
[ppm]

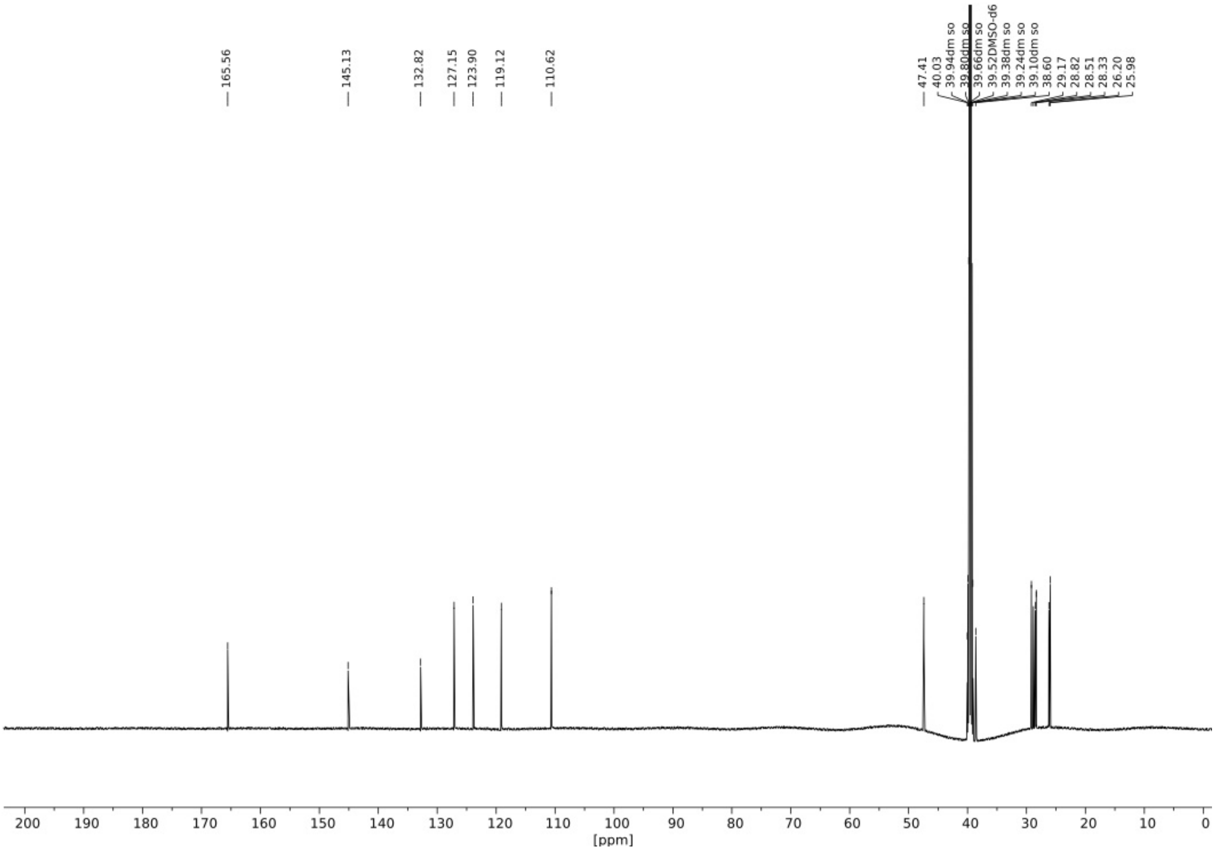
165.56
145.13
132.82
127.15
123.90
119.12
110.62
47.41
40.03
39.94dm so
39.80dm so
39.66dm so
39.52DMSO-d6
39.38dm so
39.24dm so
39.10dm so
38.60
29.17
28.82
28.51
28.33
26.20
25.98
[ppm]

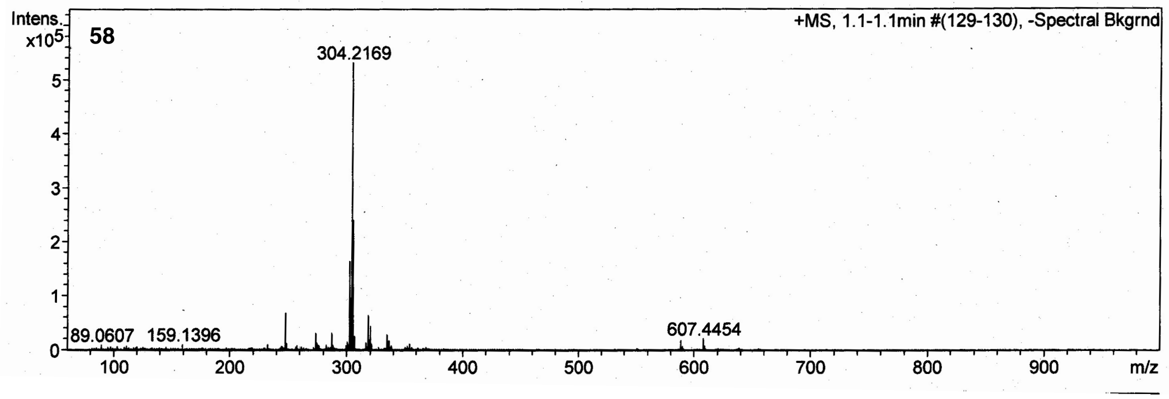
Intens.
x10^5
58
+MS, 1.1-1.1min #(129-130), -Spectral Bkgrnd
304.2169
89.0607
159.1396
607.4454
m/z

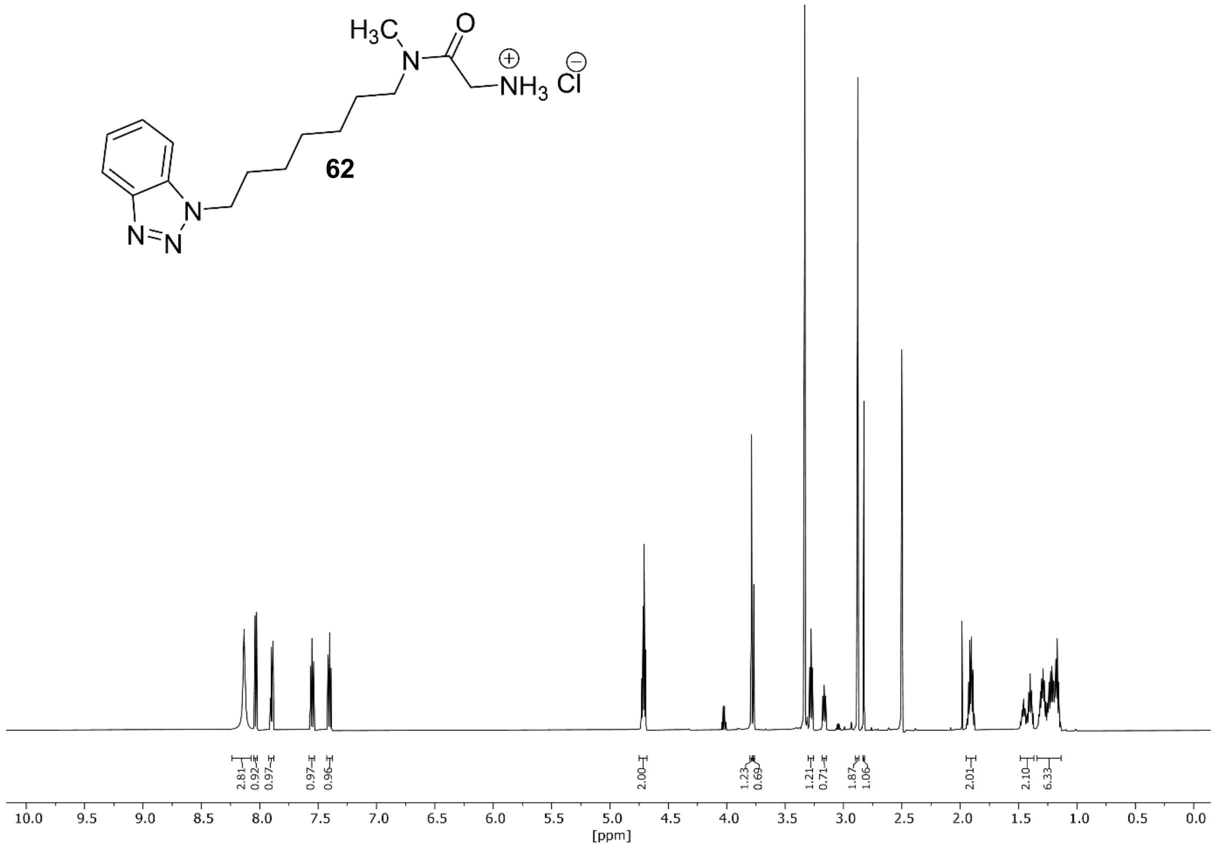
H3C
N
O
NH3
Cl
N
N=N
62
2.81
0.92
0.97
0.97
0.96
2.00
1.23
0.69
1.21
0.71
1.87
1.06
2.01
2.10
6.33
10.0 9.5 9.0 8.5 8.0 7.5 7.0 6.5 6.0 5.5 5.0 4.5 4.0 3.5 3.0 2.5 2.0 1.5 1.0 0.5 0.0
[ppm]

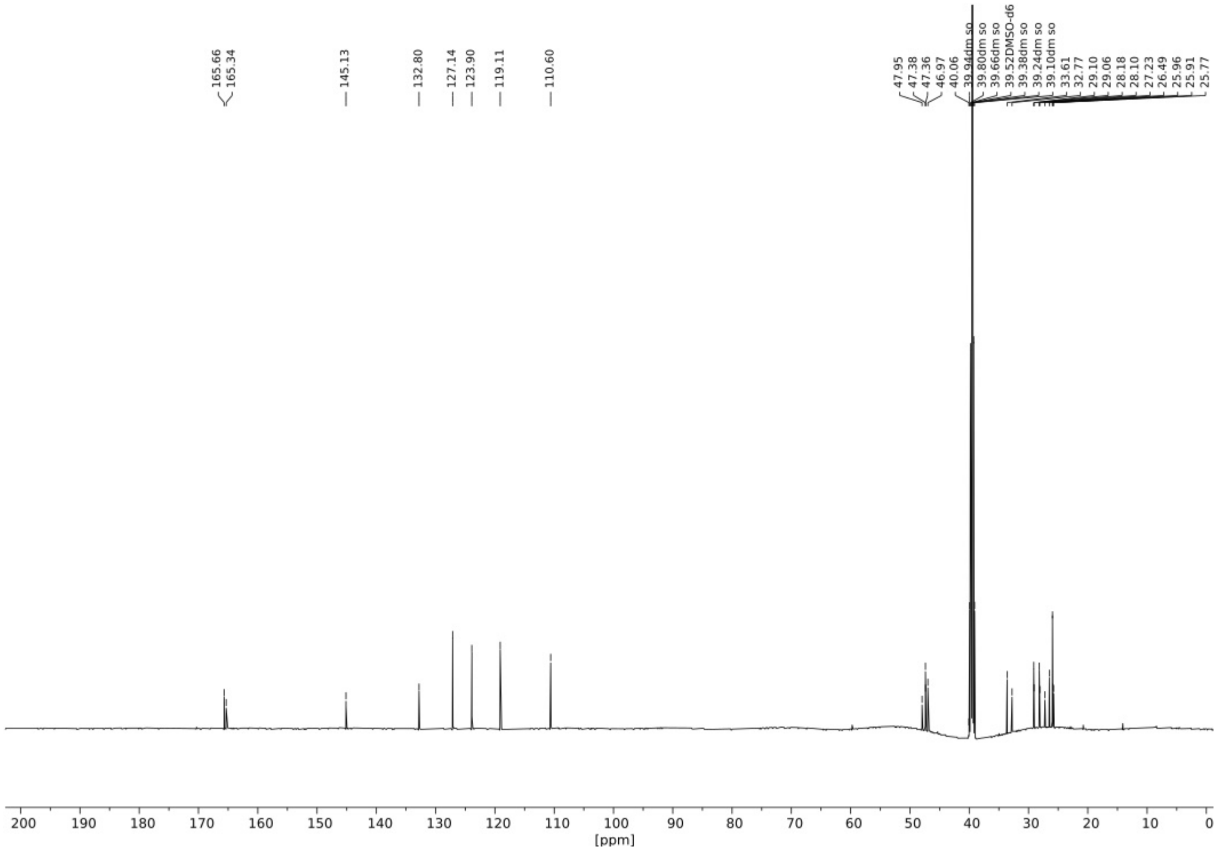
165.66
165.34
145.13
132.80
127.14
123.90
119.11
110.60
47.95
47.38
47.36
46.97
40.06
39.94dm so
39.80dm so
39.66dm so
39.52DMSO-d6
39.38dm so
39.24dm so
39.10dm so
33.61
32.77
29.10
29.06
28.18
28.10
27.23
26.49
25.96
25.91
25.77
200 190 180 170 160 150 140 130 120 110 100 90 80 70 60 50 40 30 20 10 0
[ppm]

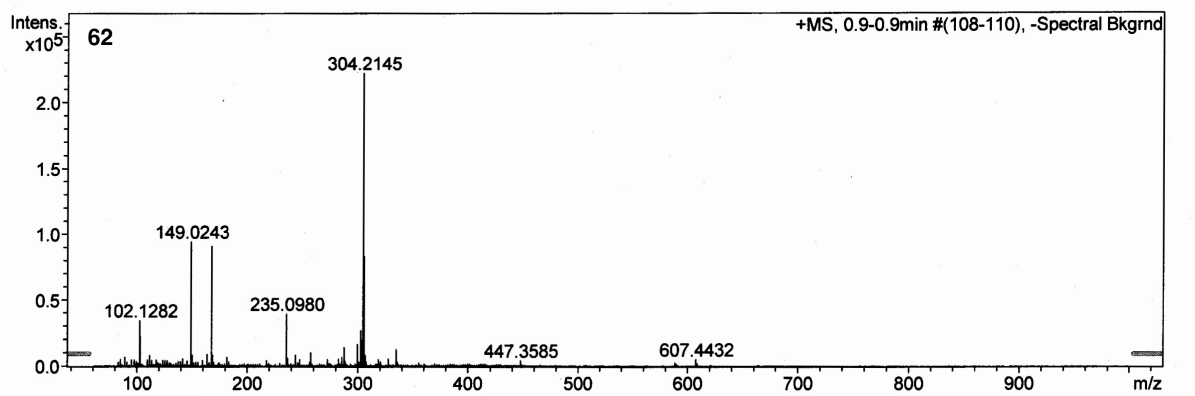
Intens.
x10^5
62
+MS, 0.9-0.9min #(108-110), -Spectral Bkgrnd
304.2145
149.0243
235.0980
102.1282
447.3585
607.4432
2.0
1.5
1.0
0.5
0.0
100
200
300
400
500
600
700
800
900
m/z

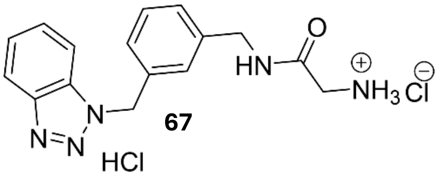
67
HCl
HN
O
NH3
Cl

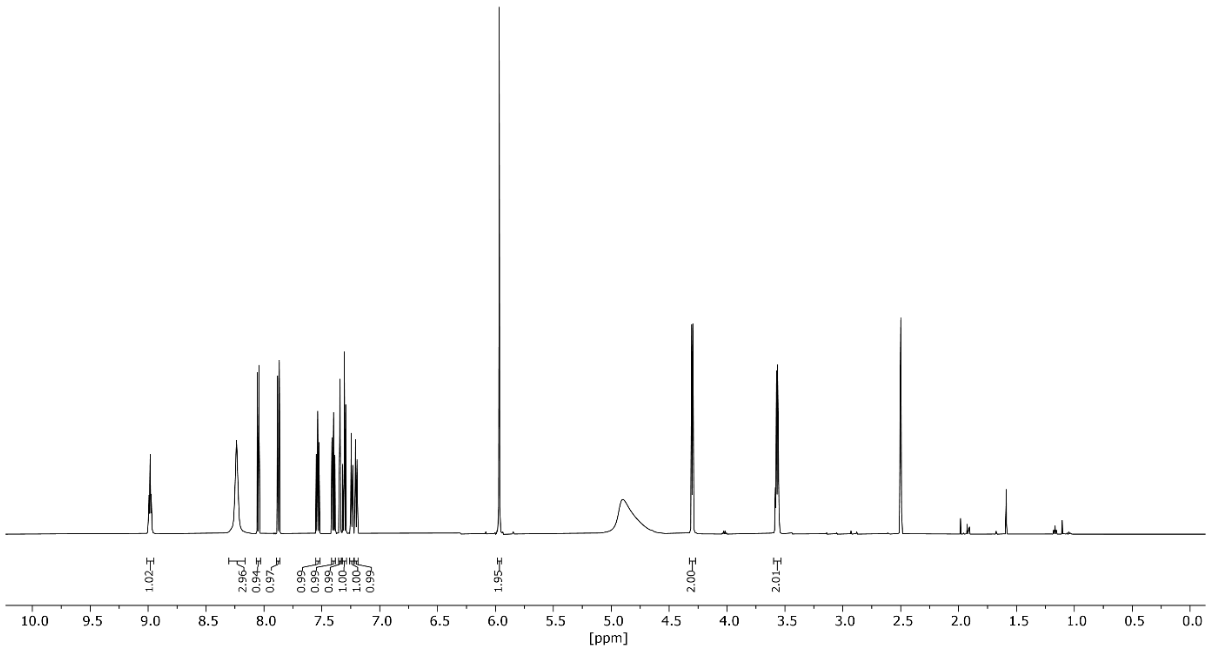
1.02
2.96
0.94
0.97
0.99
0.99
0.99
1.00
1.00
0.99
1.95
2.00
2.01
[ppm]

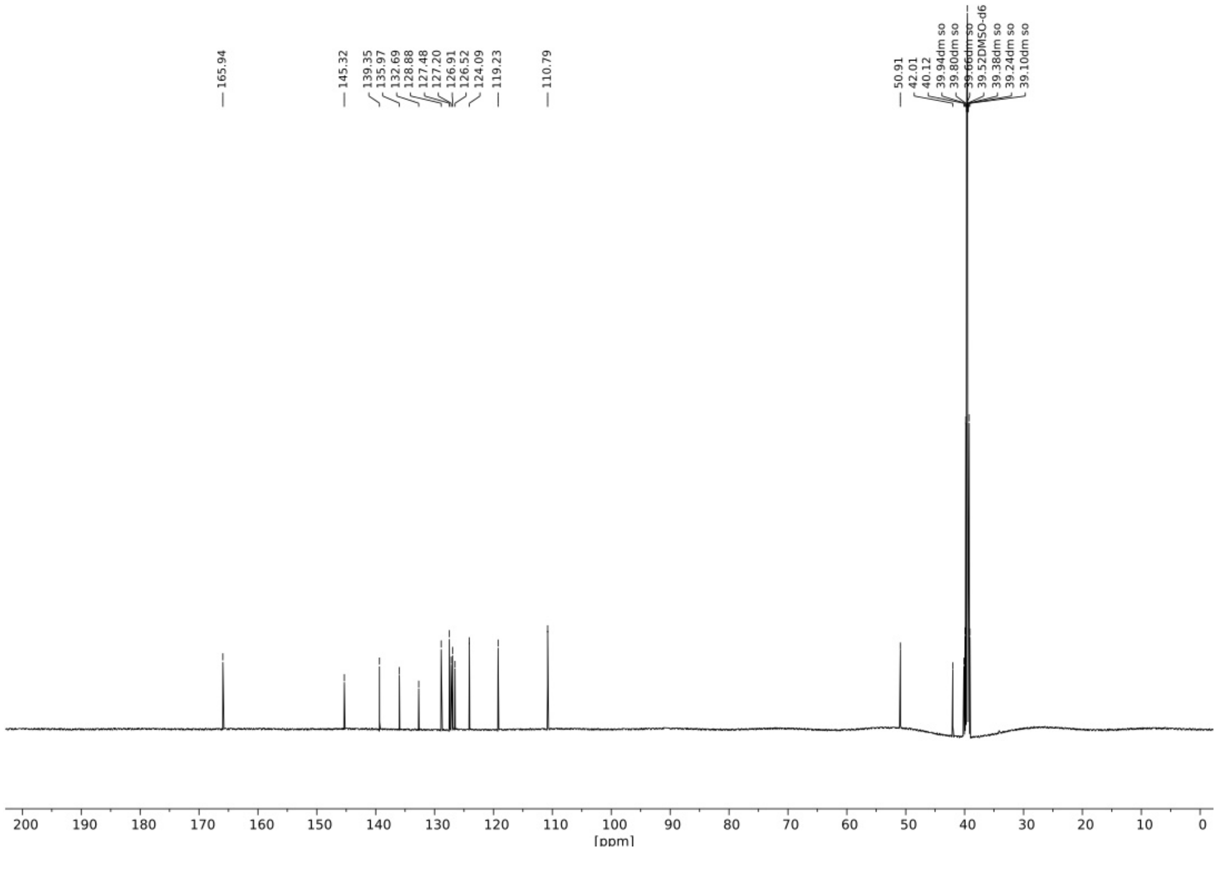
165.94
145.32
139.35
135.97
132.69
128.88
127.48
127.20
126.91
126.52
124.09
119.23
110.79
50.91
42.01
40.12
39.94dm so
39.80dm so
39.66dm so
39.52DMSO-d6
39.38dm so
39.24dm so
39.10dm so
[ppm]

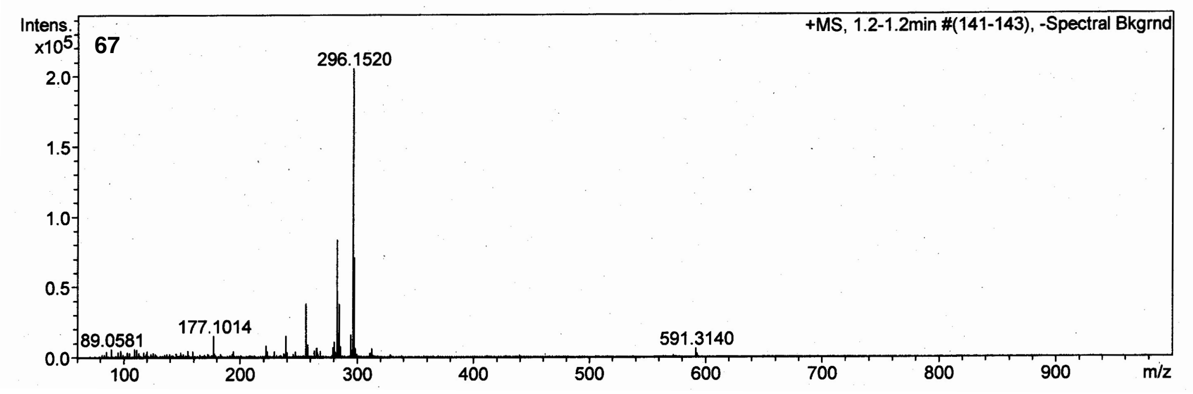
Intens.
x10^5
67
+MS, 1.2-1.2min #(141-143), -Spectral Bkgrnd
296.1520
177.1014
89.0581
591.3140
m/z

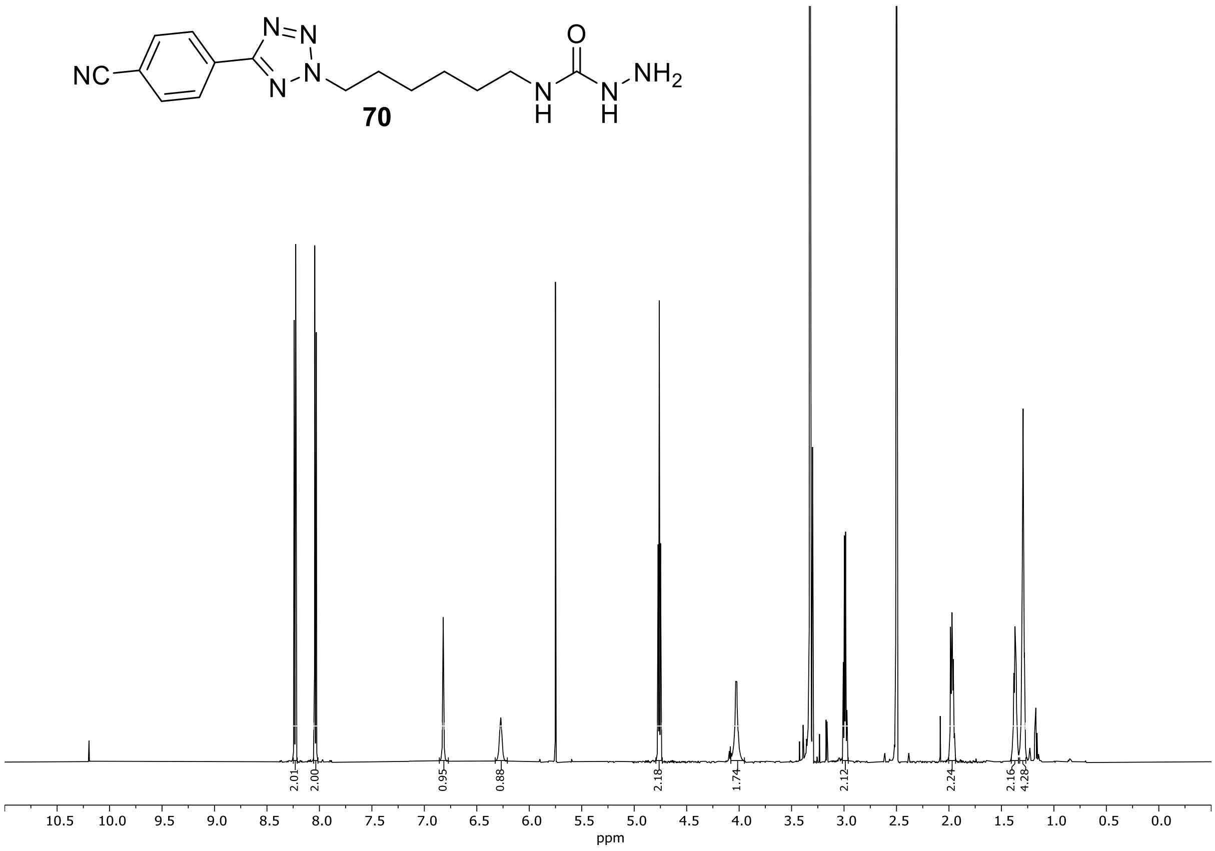
NC
N=N
N
N
NH2
O
N
H
N
H
70
2.01
2.00
0.95
0.88
2.18
1.74
2.12
2.24
2.16
4.28
10.5 10.0 9.5 9.0 8.5 8.0 7.5 7.0 6.5 6.0 5.5 5.0 4.5 4.0 3.5 3.0 2.5 2.0 1.5 1.0 0.5 0.0
ppm

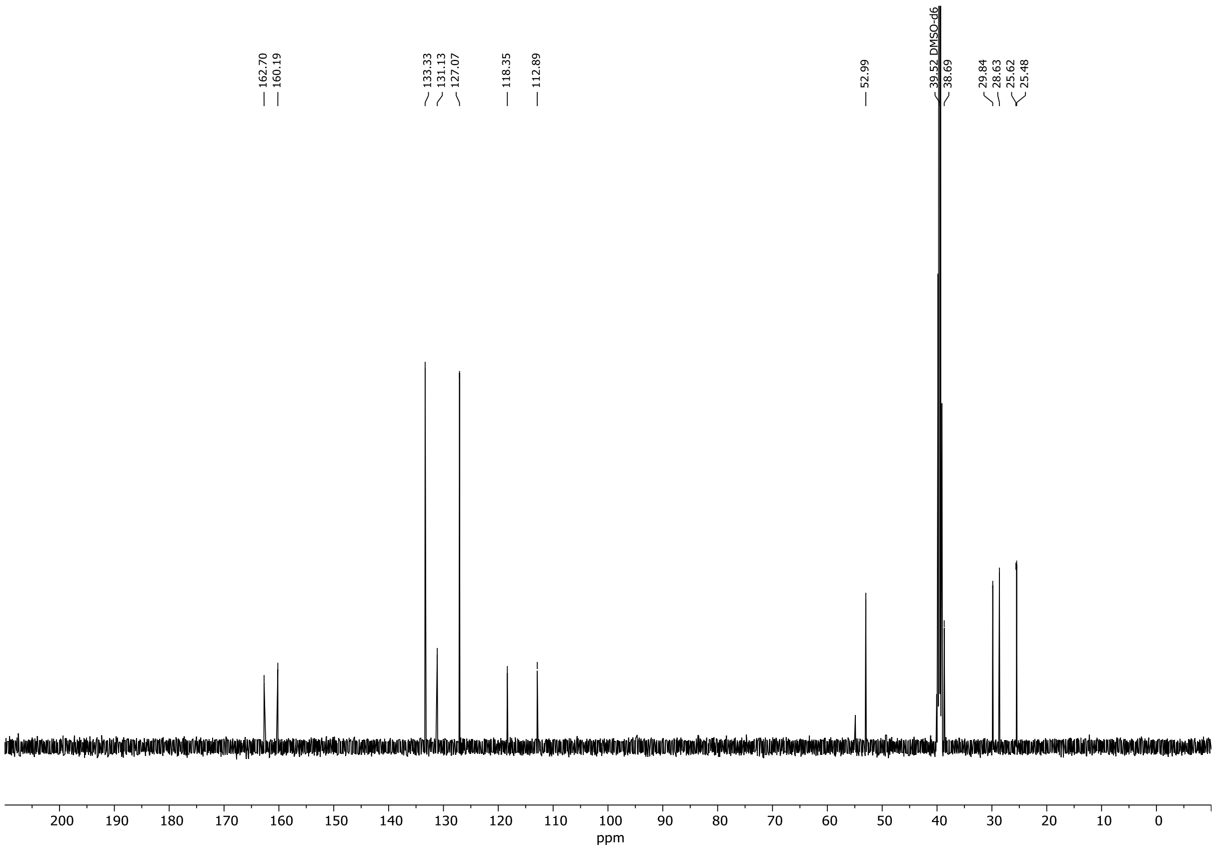
162.70
160.19
133.33
131.13
127.07
118.35
112.89
52.99
39.52 DMSO-d6
38.69
29.84
28.63
25.62
25.48
200 190 180 170 160 150 140 130 120 110 100 90 80 70 60 50 40 30 20 10 0
ppm

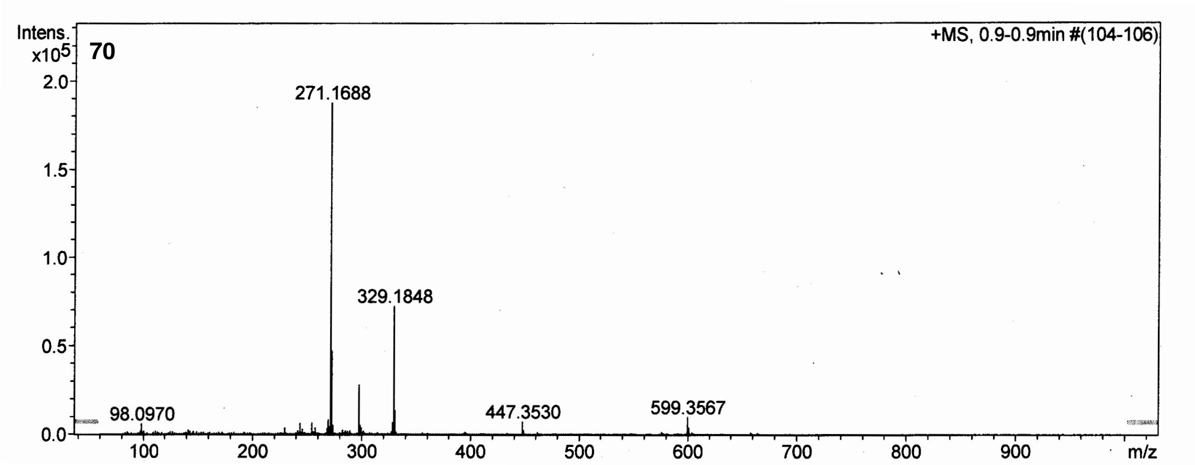
Intens.
x10^5
70
+MS, 0.9-0.9min #(104-106)
2.0
1.5
1.0
0.5
0.0
271.1688
329.1848
98.0970
447.3530
599.3567
100
200
300
400
500
600
700
800
900
m/z

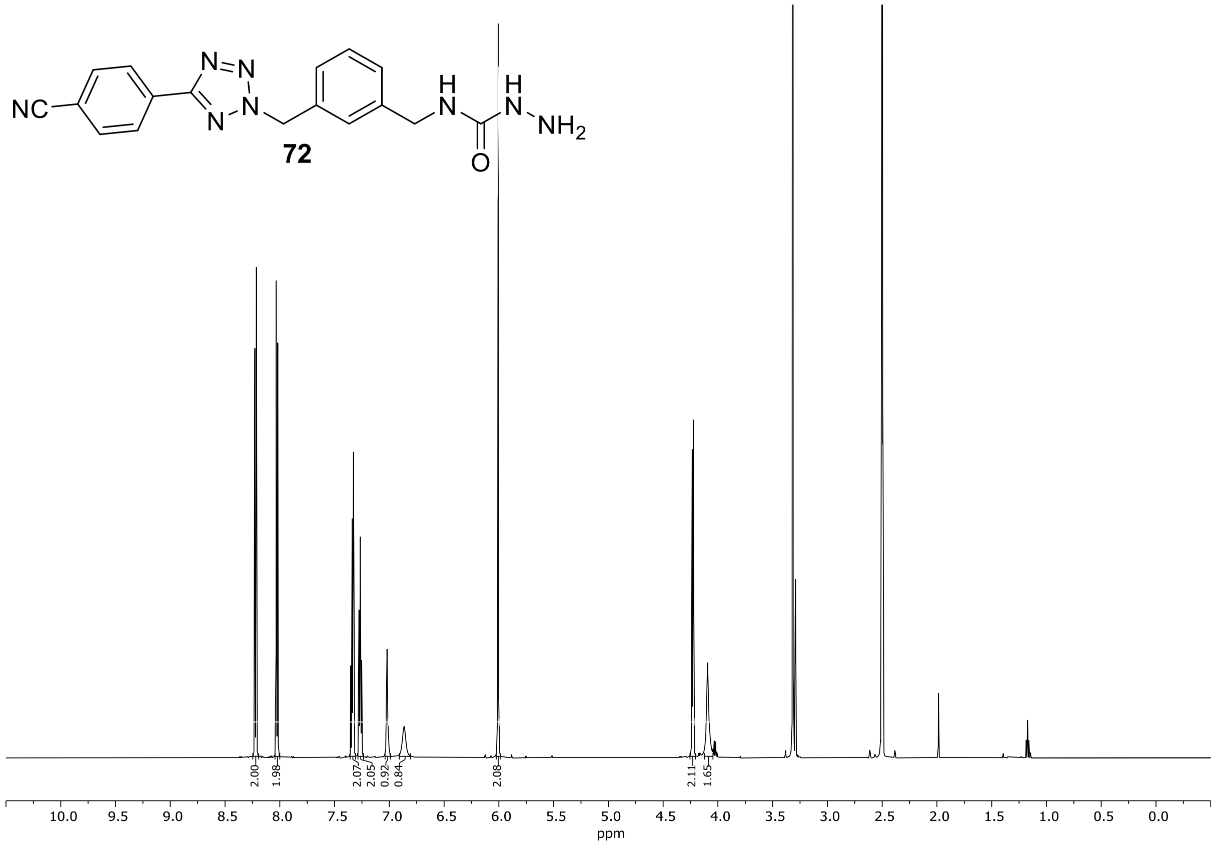
NC
N
N
N
N
H
N
H
N
NH2
O
72
2.00
1.98
2.07
2.05
0.92
0.84
2.08
2.11
1.65
10.0 9.5 9.0 8.5 8.0 7.5 7.0 6.5 6.0 5.5 5.0 4.5 4.0 3.5 3.0 2.5 2.0 1.5 1.0 0.5 0.0
ppm

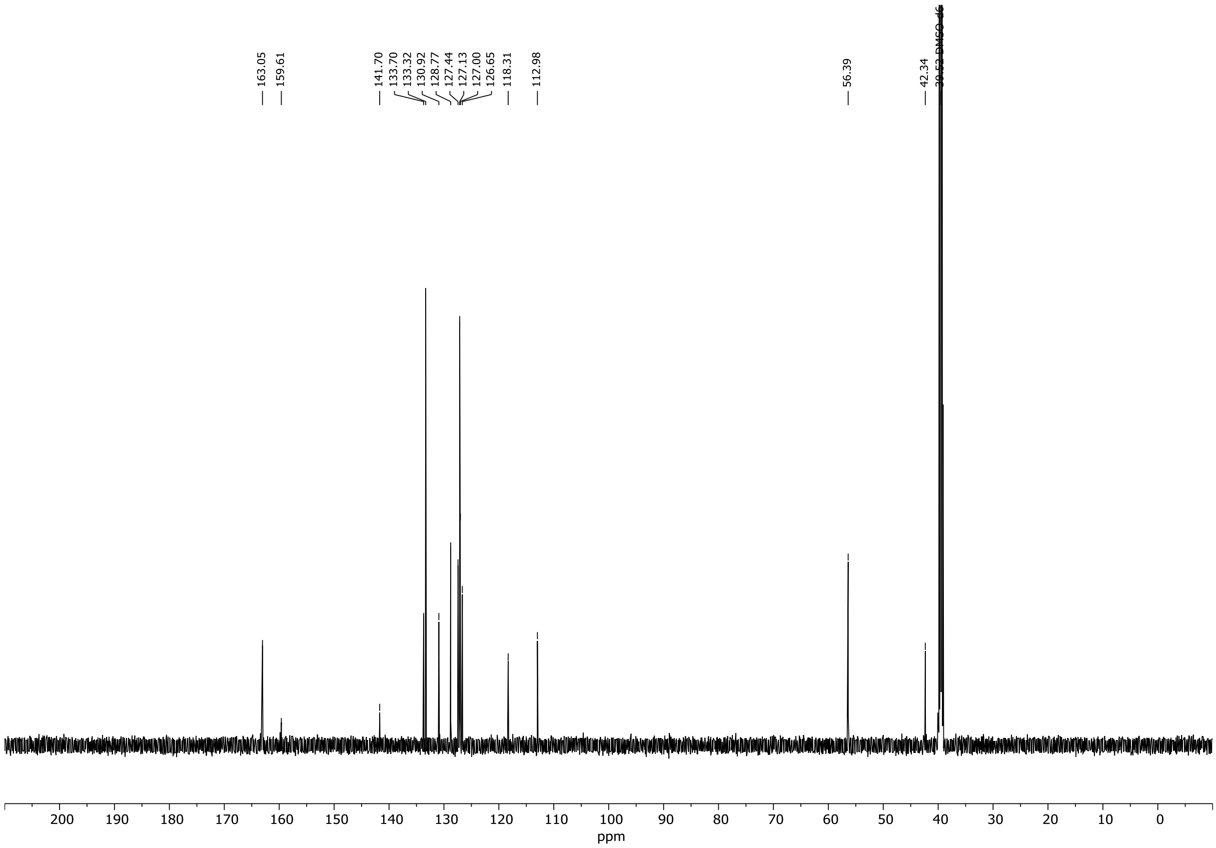
163.05
159.61
141.70
133.70
133.32
130.92
128.77
127.44
127.13
127.00
126.65
118.31
112.98
56.39
42.34
39.52 DMSO-d6
200 190 180 170 160 150 140 130 120 110 100 90 80 70 60 50 40 30 20 10 0
ppm

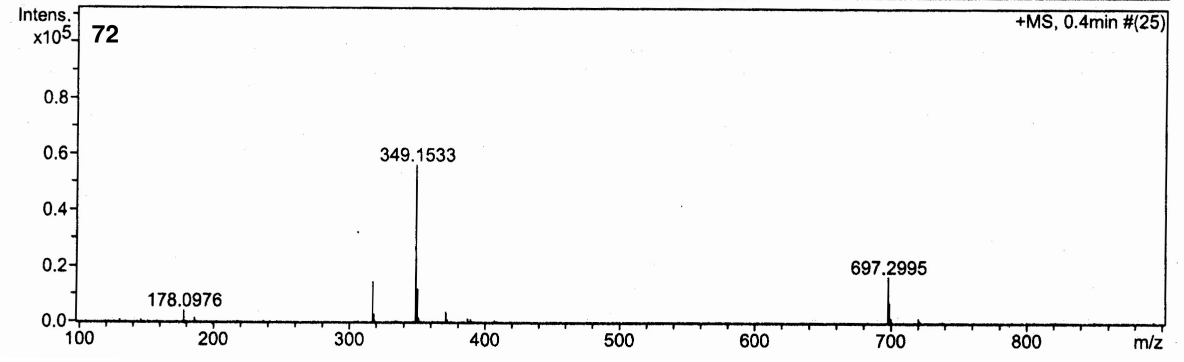
Intens.
x10^5
72
+MS, 0.4min #(25)
0.8
0.6
0.4
0.2
0.0
349.1533
697.2995
178.0976
100
200
300
400
500
600
700
800
m/z

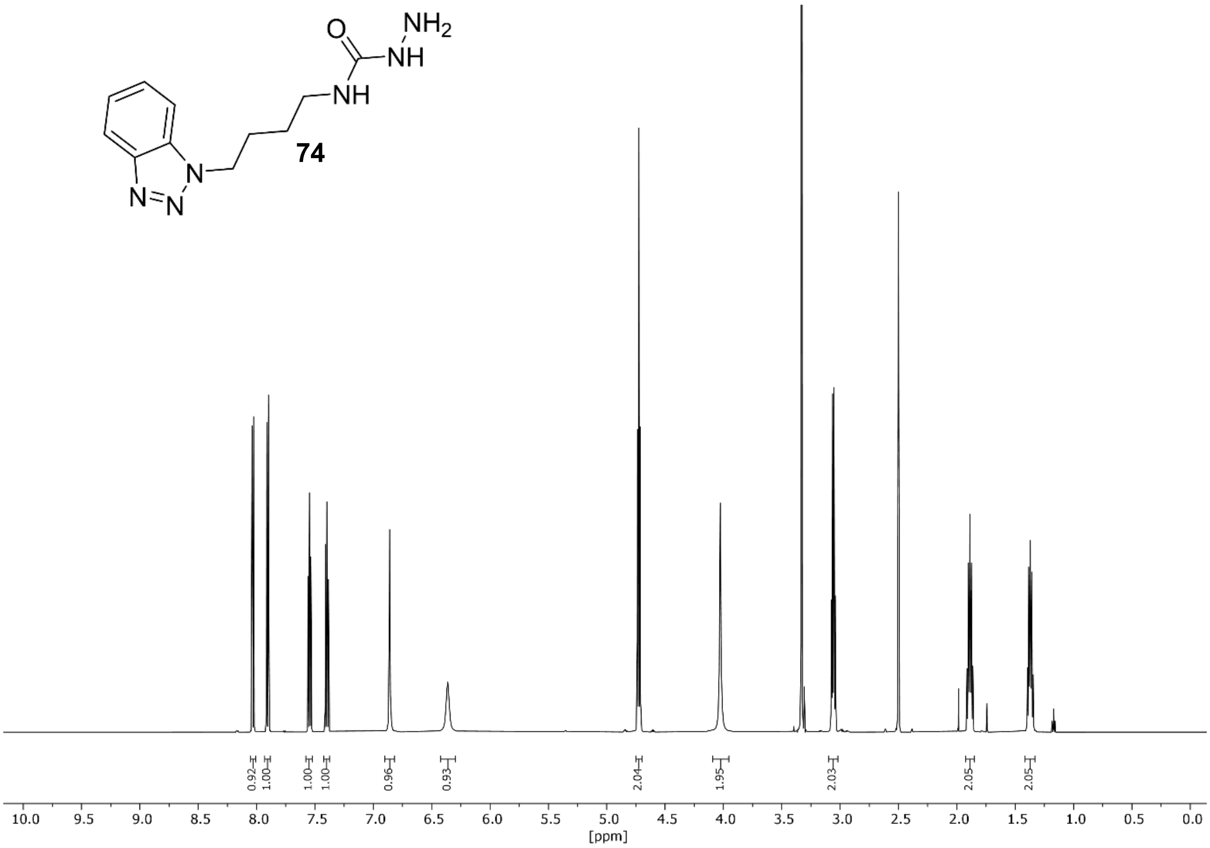
O
NH2
NH
NH
N
N
N
74
0.92
1.00
1.00
1.00
0.96
0.93
2.04
1.95
2.03
2.05
2.05
10.0 9.5 9.0 8.5 8.0 7.5 7.0 6.5 6.0 5.5 5.0 4.5 4.0 3.5 3.0 2.5 2.0 1.5 1.0 0.5 0.0
[ppm]

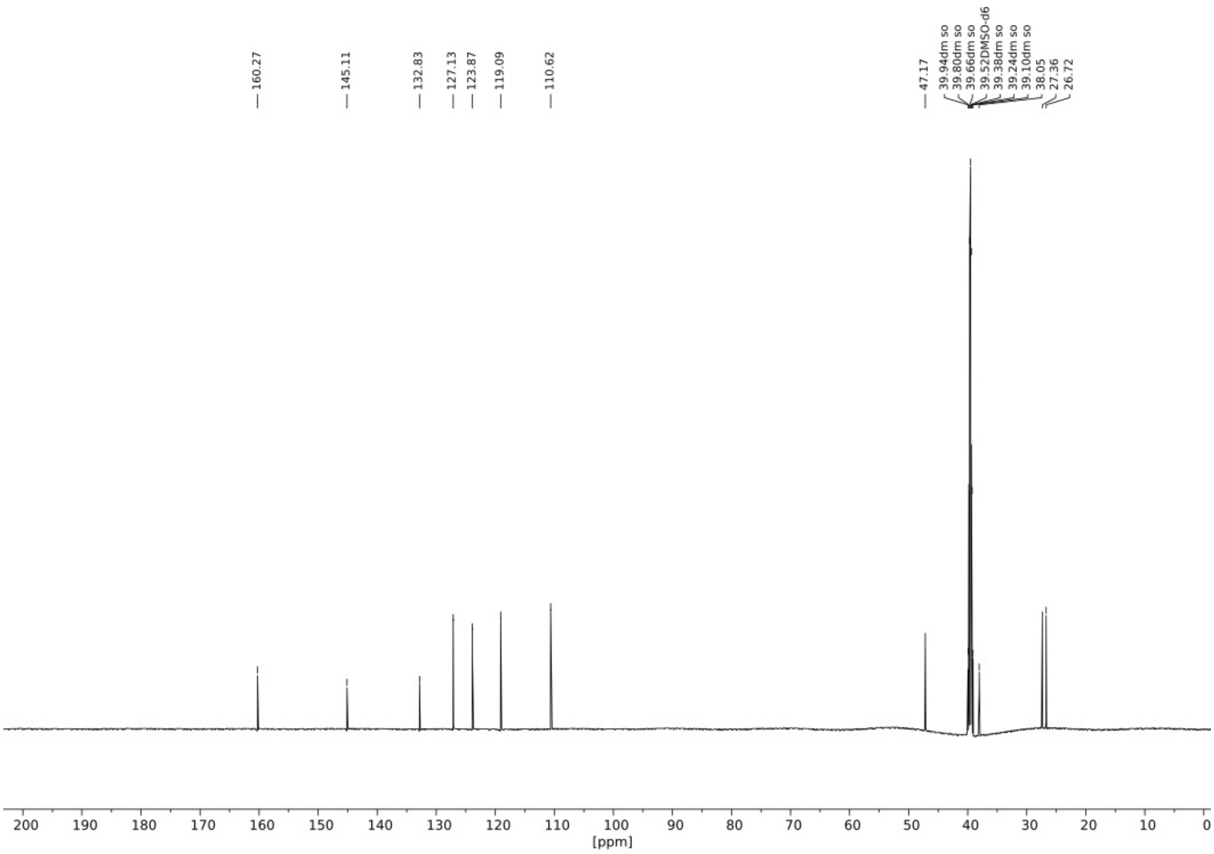
160.27
145.11
132.83
127.13
123.87
119.09
110.62
47.17
39.94dm so
39.80dm so
39.66dm so
39.52DMSO-d6
39.38dm so
39.24dm so
39.10dm so
38.05
27.36
26.72
200 190 180 170 160 150 140 130 120 110 100 90 80 70 60 50 40 30 20 10 0
[ppm]

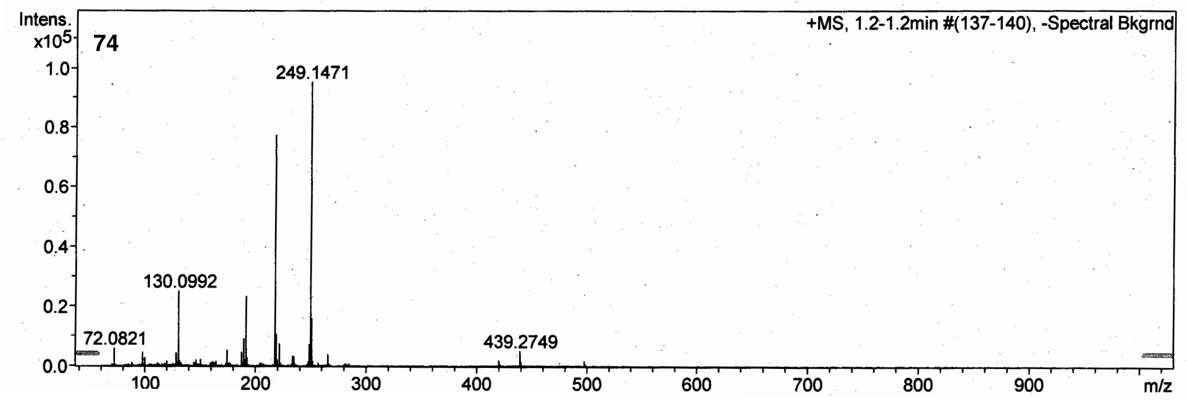
Intens.
x10^5
74
+MS, 1.2-1.2min #(137-140), -Spectral Bkgrnd
249.1471
130.0992
72.0821
439.2749
m/z

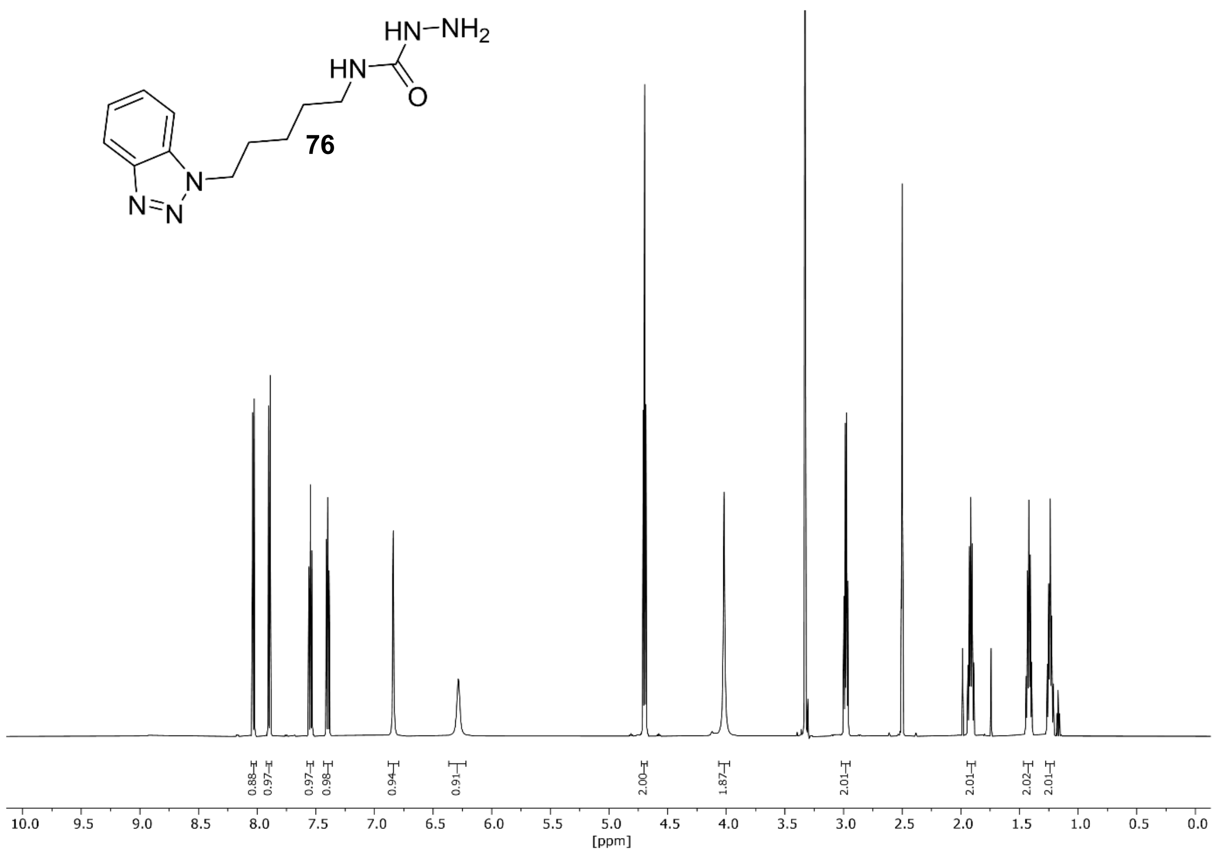
HN−NH2
HN
O
N
N=N
76
0.88
0.97
0.97
0.98
0.94
0.91
2.00
1.87
2.01
2.01
2.02
2.01
10.0 9.5 9.0 8.5 8.0 7.5 7.0 6.5 6.0 5.5 5.0 4.5 4.0 3.5 3.0 2.5 2.0 1.5 1.0 0.5 0.0
[ppm]

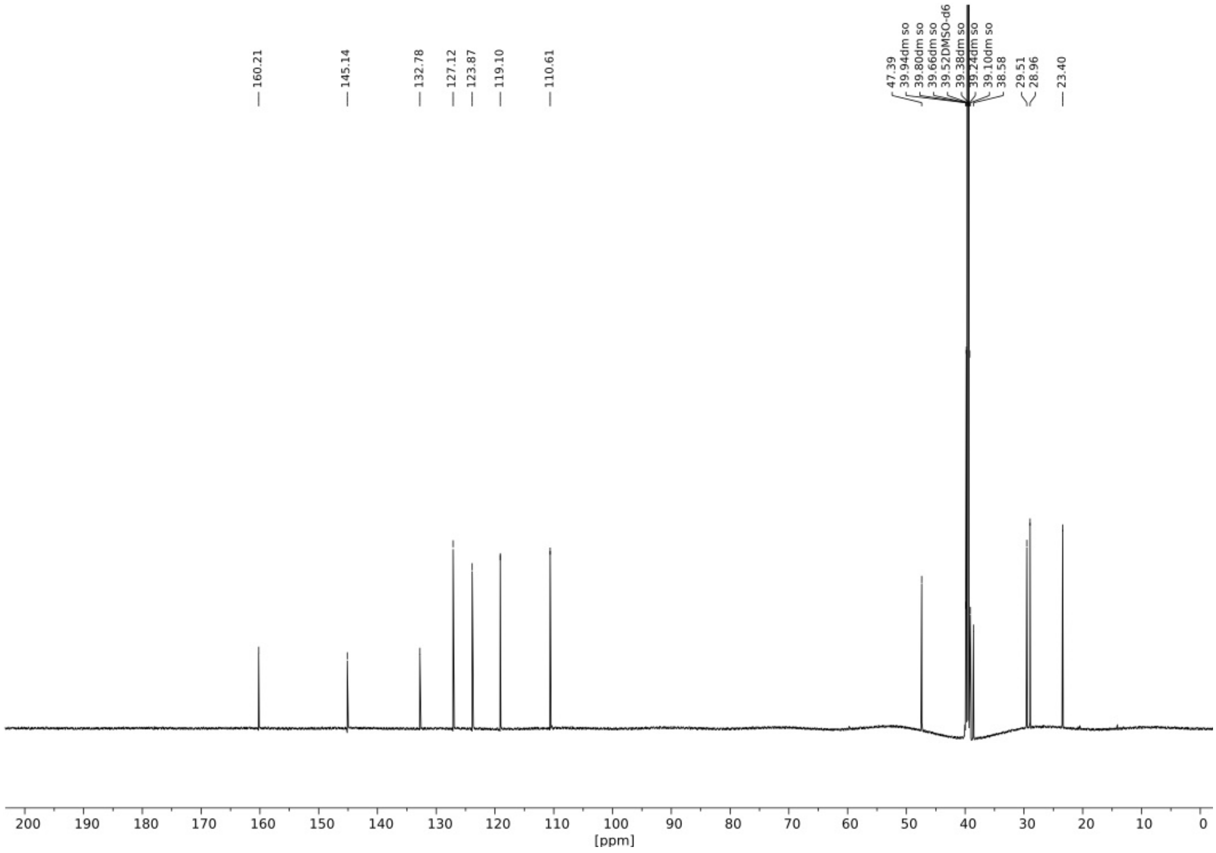
160.21
145.14
132.78
127.12
123.87
119.10
110.61
47.39
39.94dm so
39.80dm so
39.66dm so
39.52DMSO-d6
39.38dm so
39.24dm so
39.10dm so
38.58
29.51
28.96
23.40
200 190 180 170 160 150 140 130 120 110 100 90 80 70 60 50 40 30 20 10 0
[ppm]

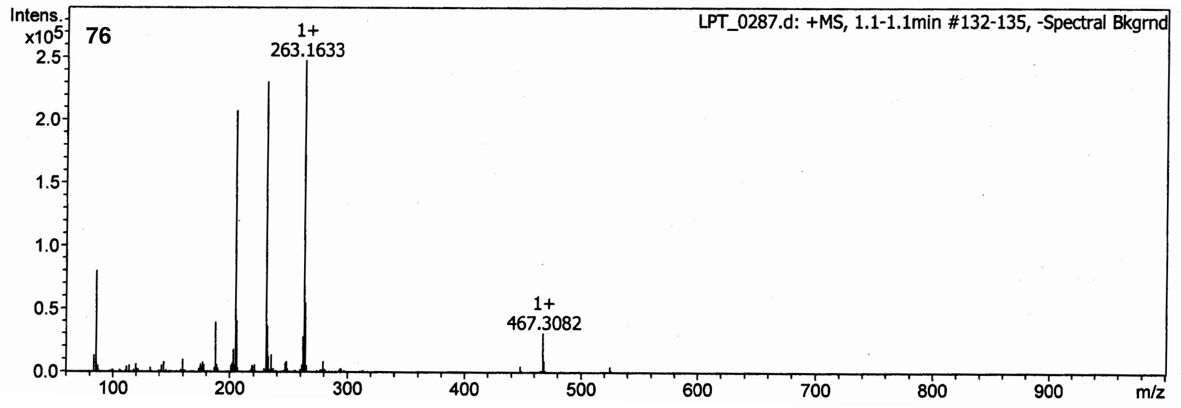
Intens.
x10^5
76
1+
263.1633
LPT_0287.d: +MS, 1.1-1.1min #132-135, -Spectral Bkgrnd
1+
467.3082
m/z

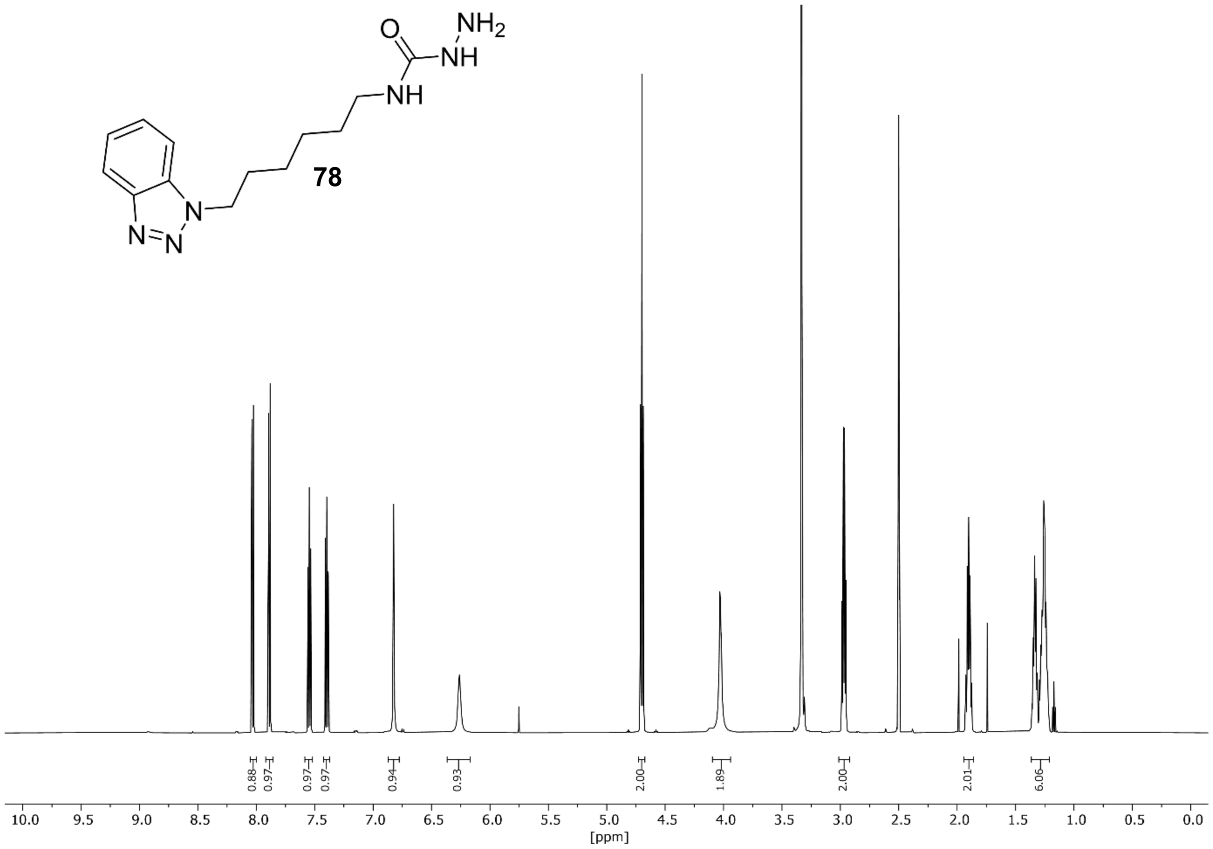
NH2
NH
O
NH
N
N
N
78
0.88
0.97
0.97
0.97
0.94
0.93
2.00
1.89
2.00
2.01
6.06
10.0 9.5 9.0 8.5 8.0 7.5 7.0 6.5 6.0 5.5 5.0 4.5 4.0 3.5 3.0 2.5 2.0 1.5 1.0 0.5 0.0
[ppm]

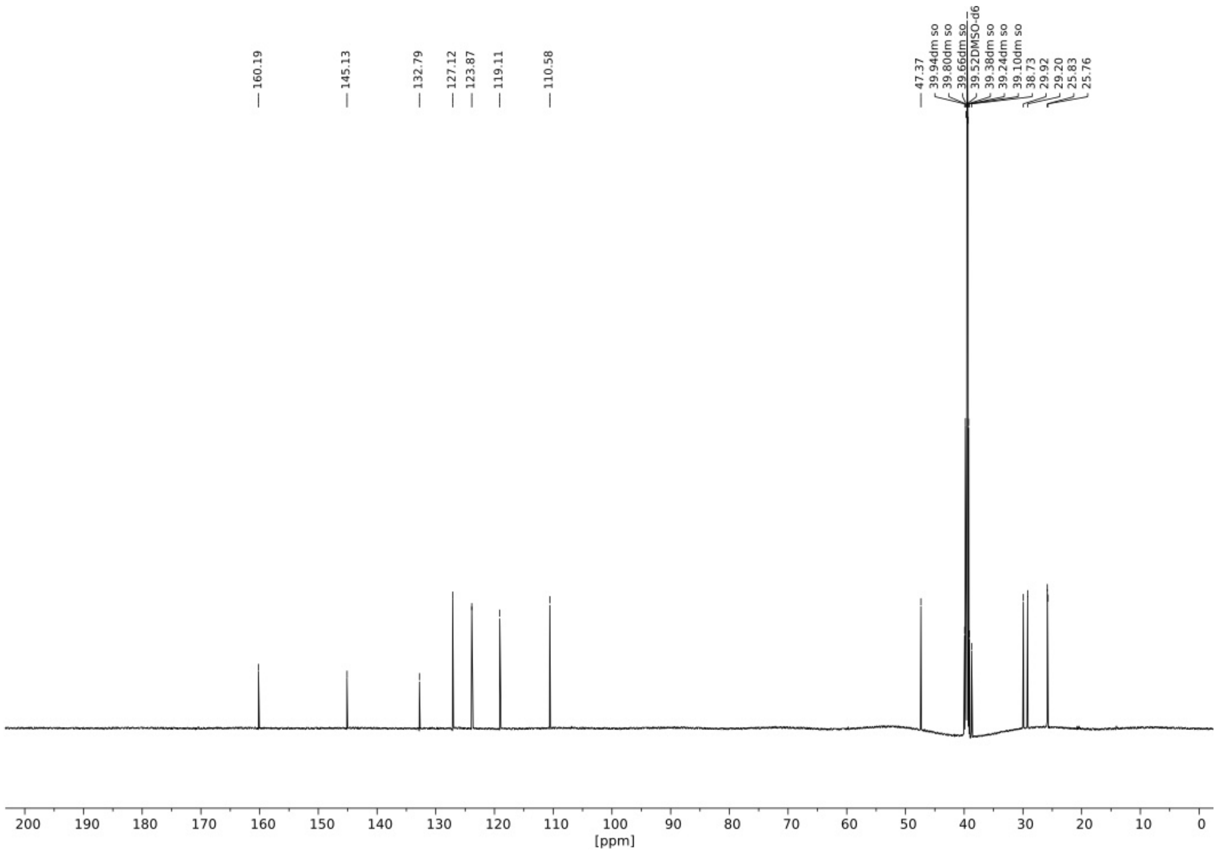
160.19
145.13
132.79
127.12
123.87
119.11
110.58
47.37
39.94dm so
39.80dm so
39.66dm so
39.52DMSO-d6
39.38dm so
39.24dm so
39.10dm so
38.73
29.92
29.20
25.83
25.76
200 190 180 170 160 150 140 130 120 110 100 90 80 70 60 50 40 30 20 10 0
[ppm]

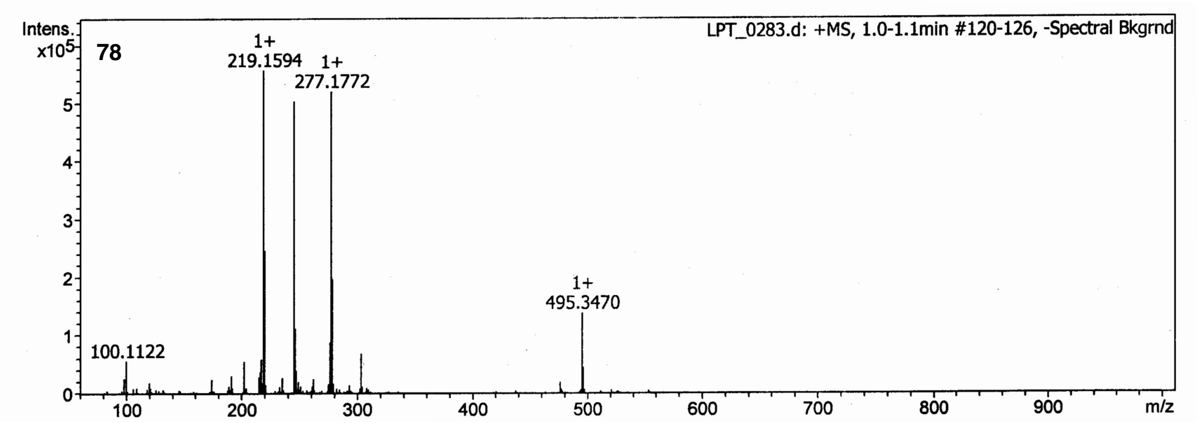
Intens.
x10^5
78
LPT_0283.d: +MS, 1.0-1.1min #120-126, -Spectral Bkgrnd
1+
219.1594
1+
277.1772
1+
495.3470
100.1122
m/z

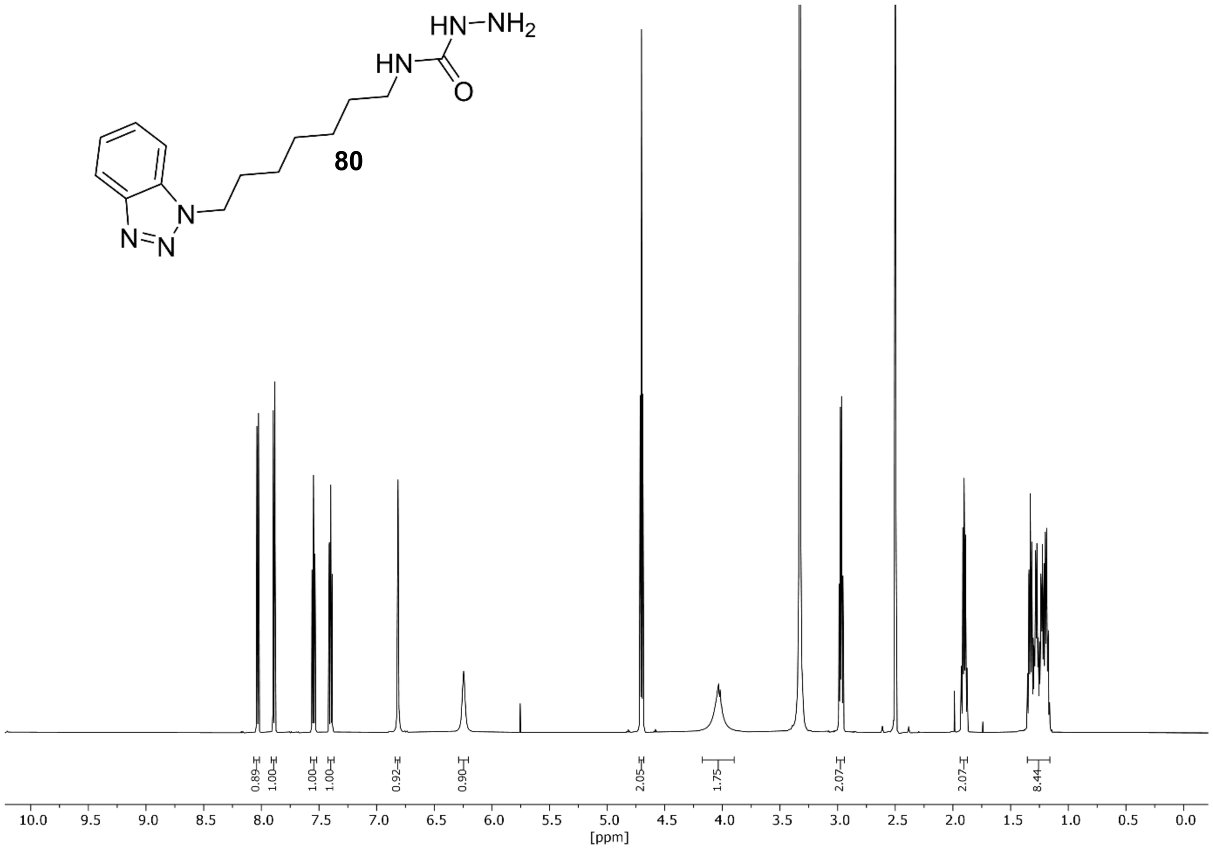
HN–NH2
HN
O
N
N=N
80
0.89
1.00
1.00
1.00
0.92
0.90
2.05
1.75
2.07
2.07
8.44
10.0 9.5 9.0 8.5 8.0 7.5 7.0 6.5 6.0 5.5 5.0 4.5 4.0 3.5 3.0 2.5 2.0 1.5 1.0 0.5 0.0
[ppm]

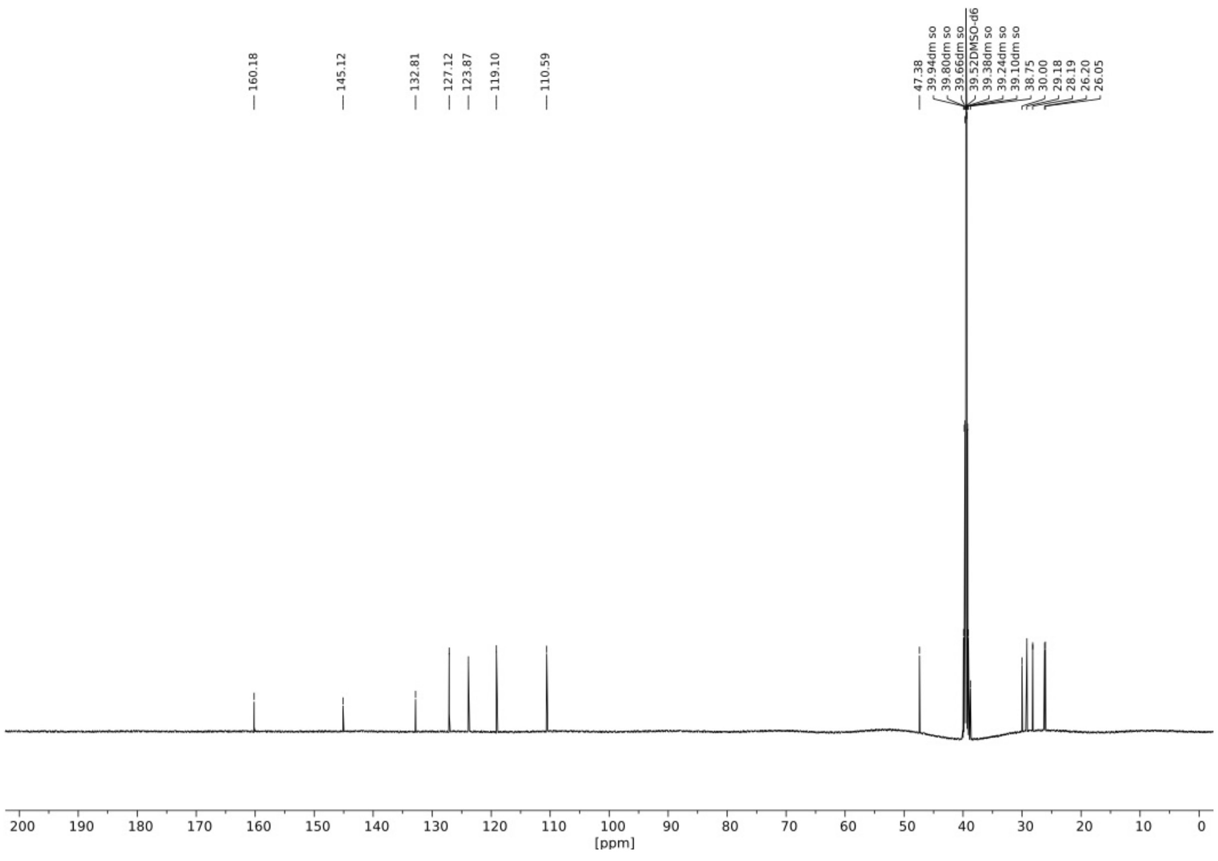
160.18
145.12
132.81
127.12
123.87
119.10
110.59
47.38
39.94dm so
39.80dm so
39.66dm so
39.52DMSO-d6
39.38dm so
39.24dm so
39.10dm so
38.75
30.00
29.18
28.19
26.20
26.05
200 190 180 170 160 150 140 130 120 110 100 90 80 70 60 50 40 30 20 10 0
[ppm]

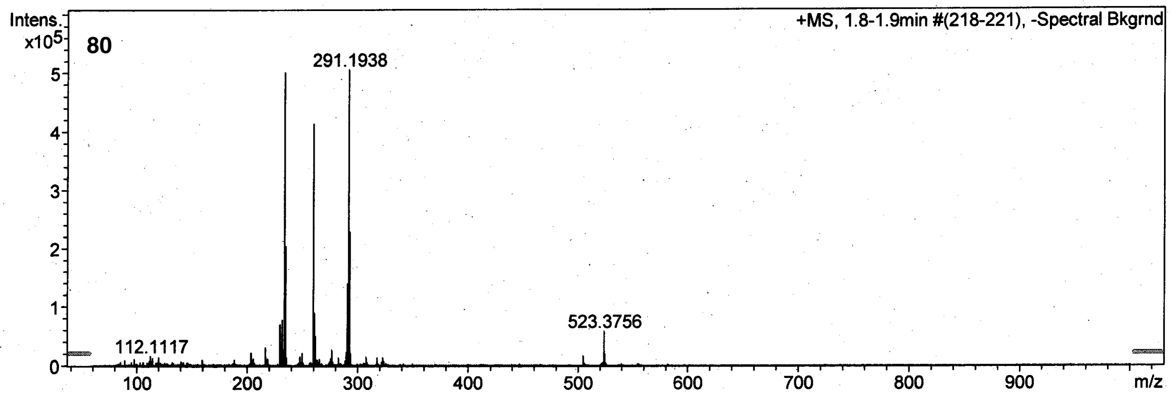
Intens.
x10^5
80
+MS, 1.8-1.9min #(218-221), -Spectral Bkgrnd
291.1938
523.3756
112.1117
m/z

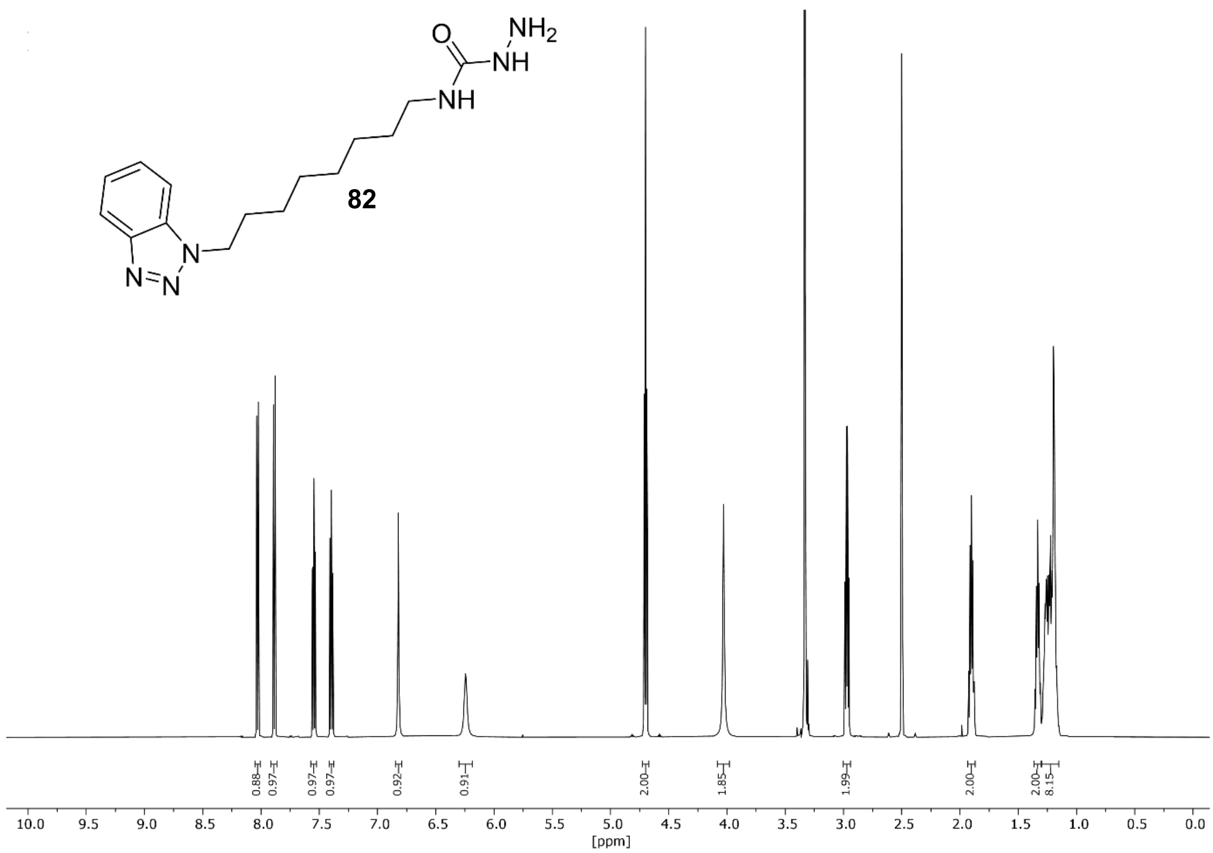
O
NH2
NH
NH
N
N
N
82
0.88
0.97
0.97
0.97
0.92
0.91
2.00
1.85
1.99
2.00
2.00
8.15
10.0 9.5 9.0 8.5 8.0 7.5 7.0 6.5 6.0 5.5 5.0 4.5 4.0 3.5 3.0 2.5 2.0 1.5 1.0 0.5 0.0
[ppm]

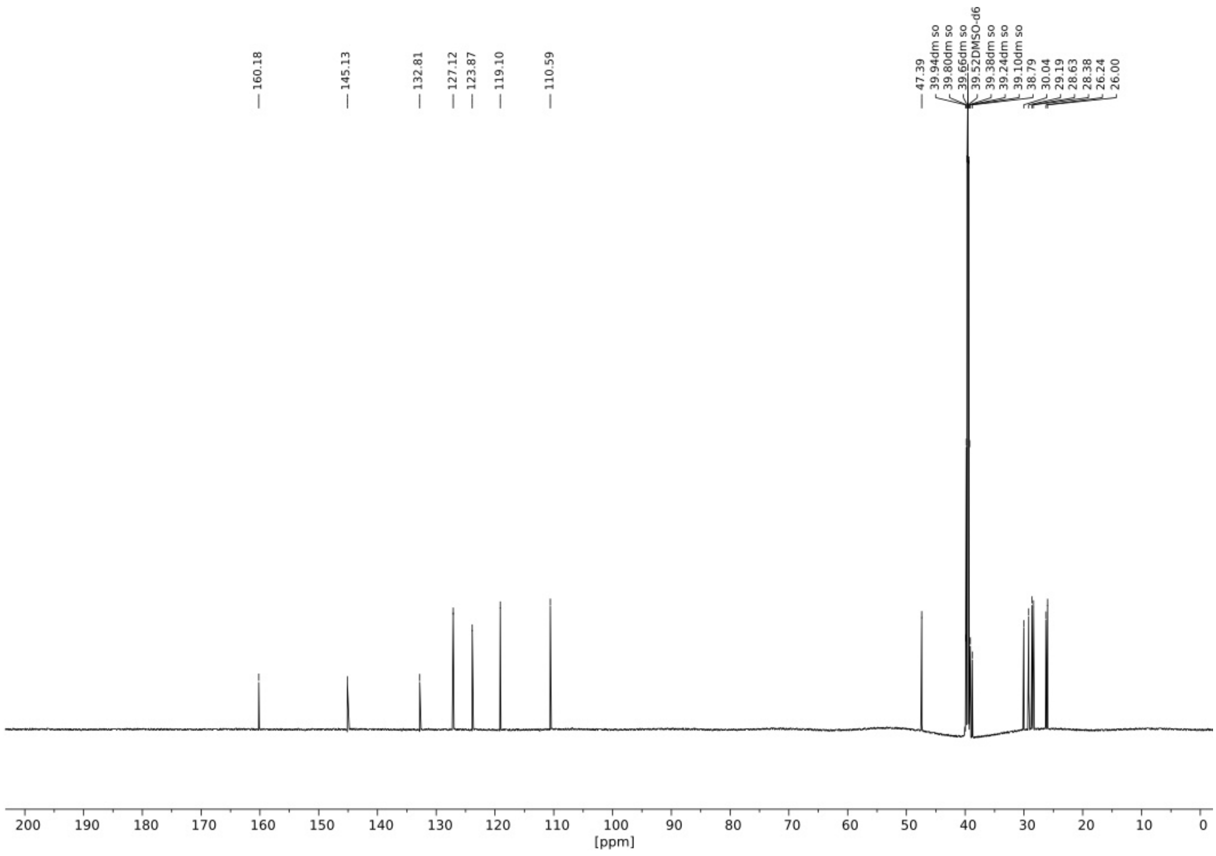
160.18
145.13
132.81
127.12
123.87
119.10
110.59
47.39
39.94dm so
39.80dm so
39.66dm so
39.52DMSO-d6
39.38dm so
39.24dm so
39.10dm so
38.79
30.04
29.19
28.63
28.38
26.24
26.00
200 190 180 170 160 150 140 130 120 110 100 90 80 70 60 50 40 30 20 10 0
[ppm]

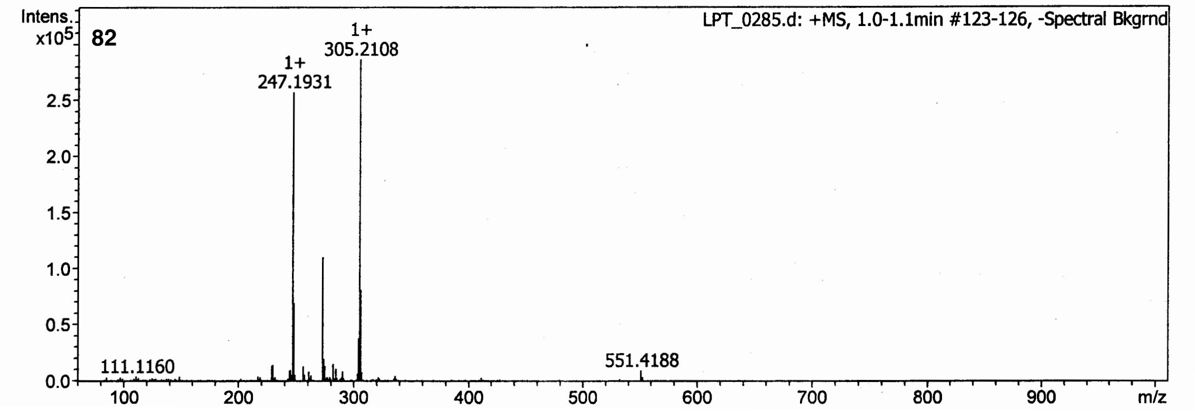
Intens.
x10^5
82
LPT_0285.d: +MS, 1.0-1.1min #123-126, -Spectral Bkgrnd
1+
305.2108
1+
247.1931
2.5
2.0
1.5
1.0
0.5
0.0
111.1160
551.4188
100
200
300
400
500
600
700
800
900
m/z

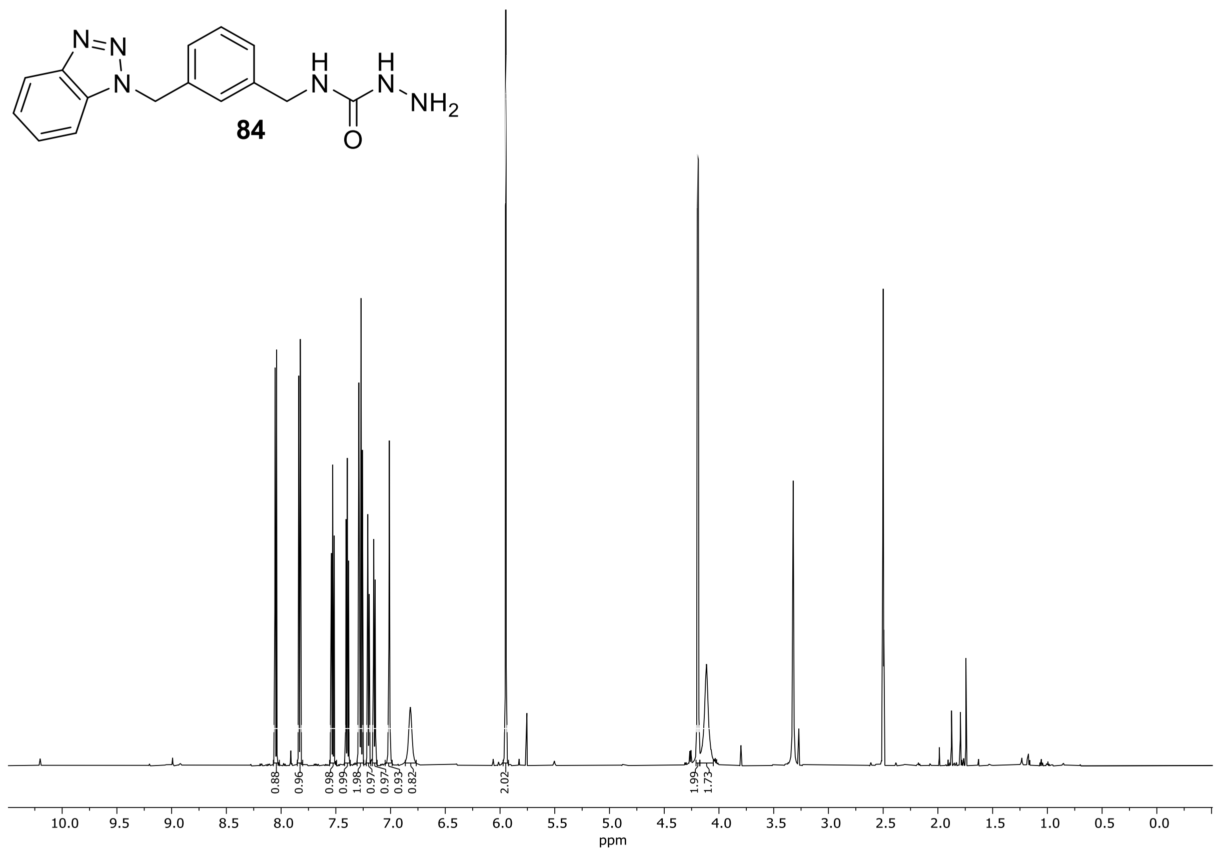
84
0.88
0.96
0.98
0.99
1.98
0.97
0.97
0.93
0.82
2.02
1.99
1.73
ppm

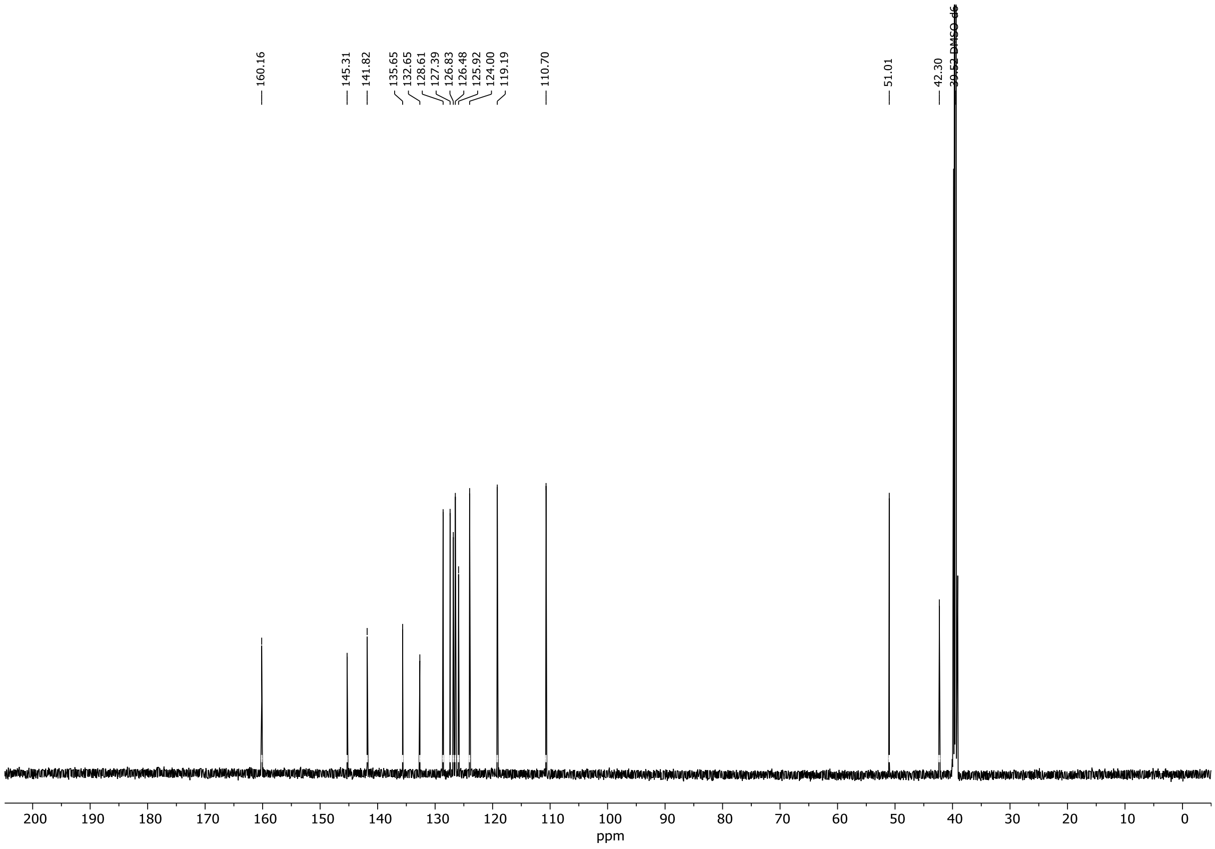
160.16
145.31
141.82
135.65
132.65
128.61
127.39
126.83
126.48
125.92
124.00
119.19
110.70
51.01
42.30
39.52 DMSO-d6
ppm

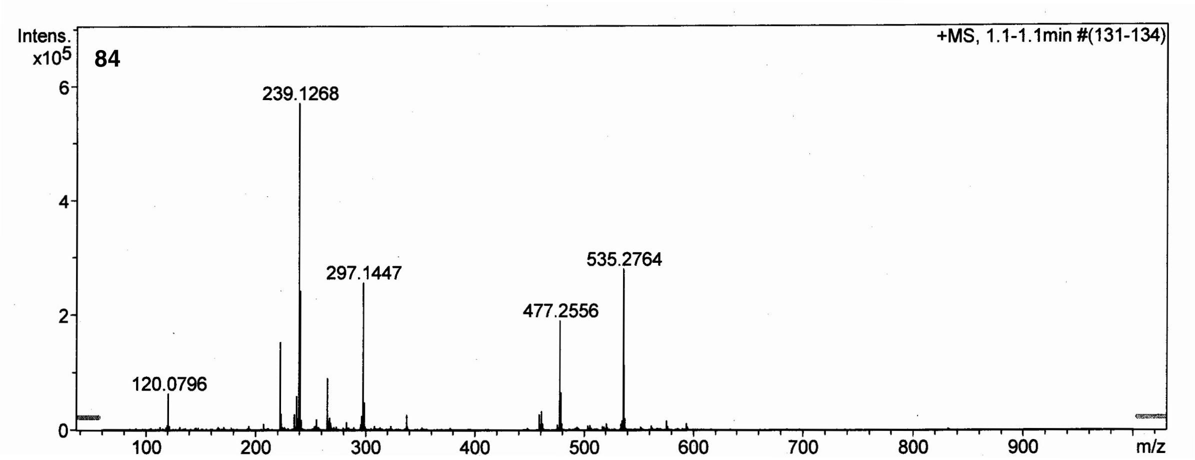
Intens.
x10^5
84
+MS, 1.1-1.1min #(131-134)
239.1268
297.1447
535.2764
477.2556
120.0796
m/z

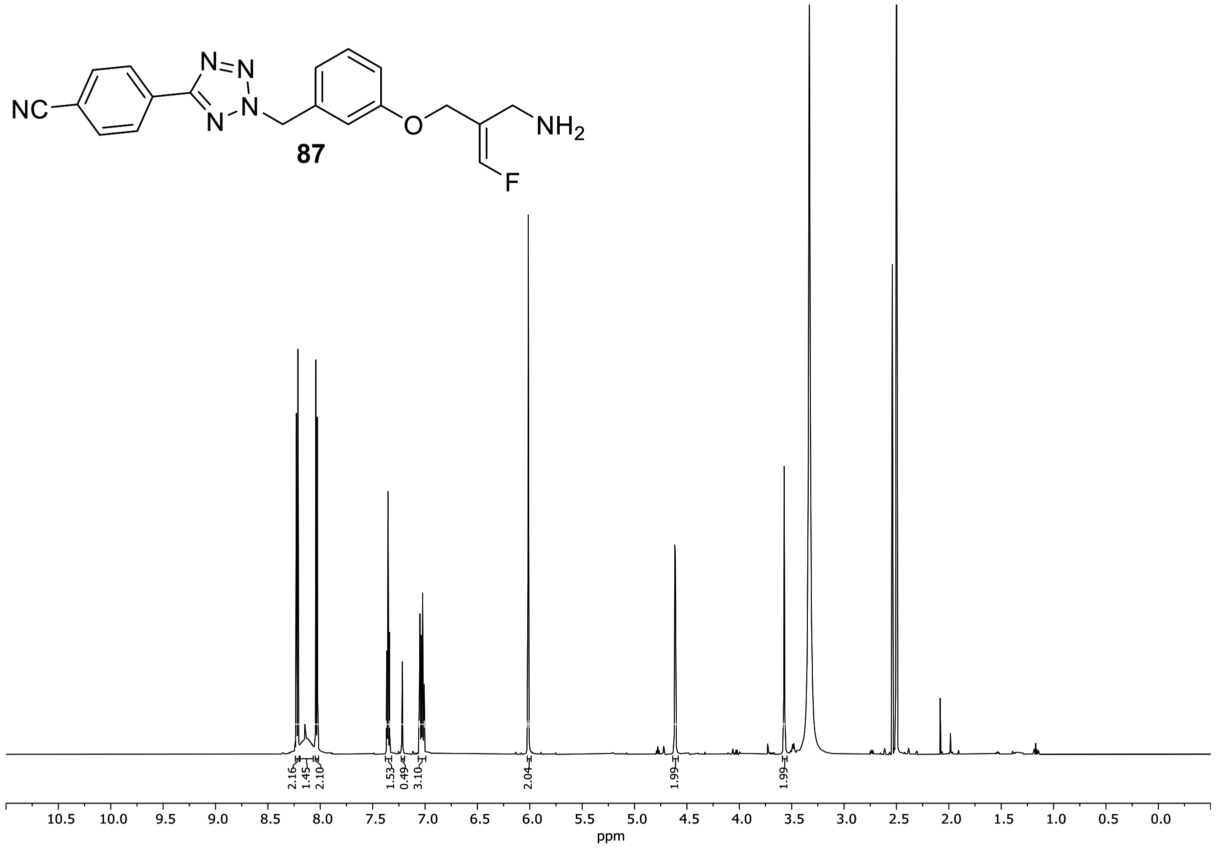
NC
N=N
N
N
NH2
O
F
87
2.16
1.45
2.10
1.53
0.49
3.10
2.04
1.99
1.99
10.5 10.0 9.5 9.0 8.5 8.0 7.5 7.0 6.5 6.0 5.5 5.0 4.5 4.0 3.5 3.0 2.5 2.0 1.5 1.0 0.5 0.0
ppm

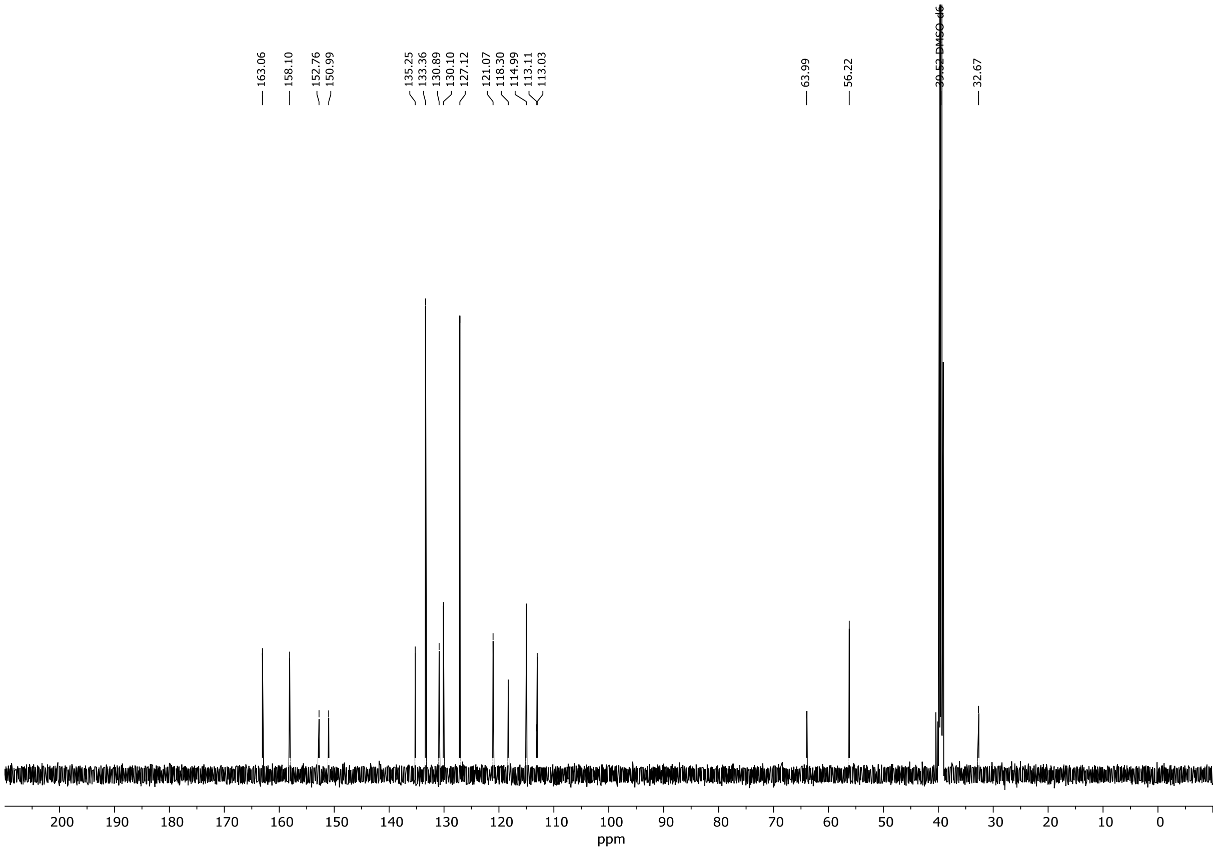
163.06
158.10
152.76
150.99
135.25
133.36
130.89
130.10
127.12
121.07
118.30
114.99
113.11
113.03
63.99
56.22
39.52 DMSO-d6
32.67
200 190 180 170 160 150 140 130 120 110 100 90 80 70 60 50 40 30 20 10 0
ppm

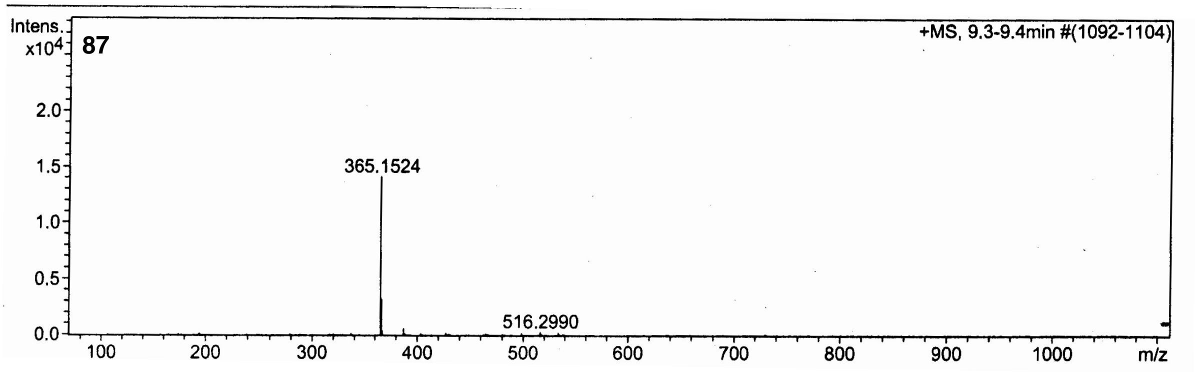
Intens.
x10^4
87
+MS, 9.3-9.4min #(1092-1104)
2.0
1.5
1.0
0.5
0.0
365.1524
516.2990
100
200
300
400
500
600
700
800
900
1000
m/z

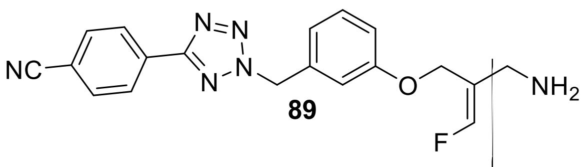
NC
N=N
N
N
O
NH2
F

89

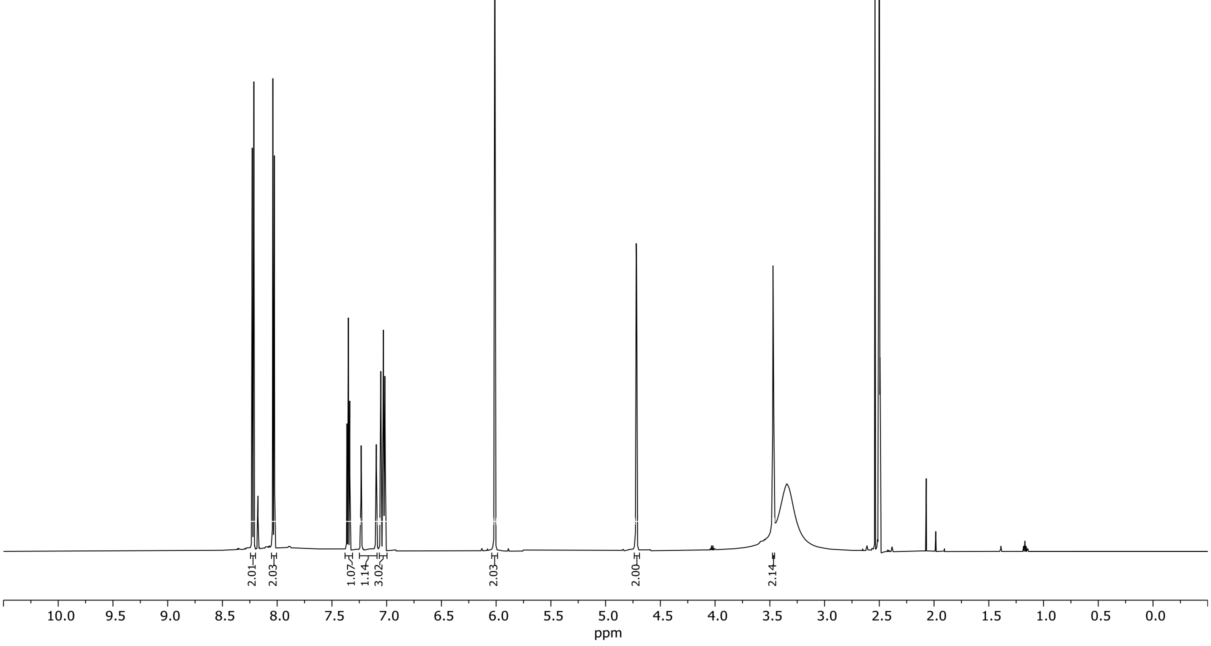
2.01
2.03
1.07
1.14
3.02
2.03
2.00
2.14
10.0 9.5 9.0 8.5 8.0 7.5 7.0 6.5 6.0 5.5 5.0 4.5 4.0 3.5 3.0 2.5 2.0 1.5 1.0 0.5 0.0
ppm

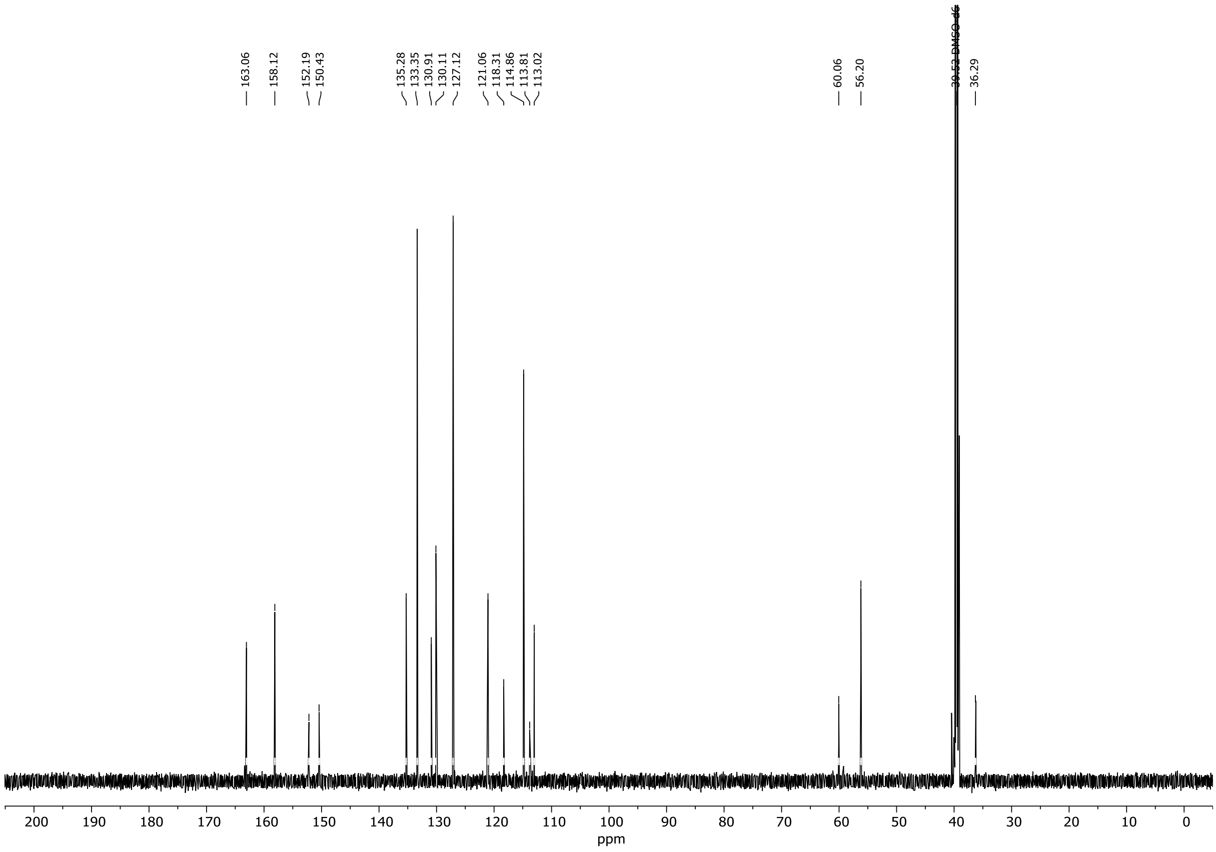
163.06
158.12
152.19
150.43
135.28
133.35
130.91
130.11
127.12
121.06
118.31
114.86
113.81
113.02
60.06
56.20
39.52 DMSO-d6
36.29
200 190 180 170 160 150 140 130 120 110 100 90 80 70 60 50 40 30 20 10 0
ppm

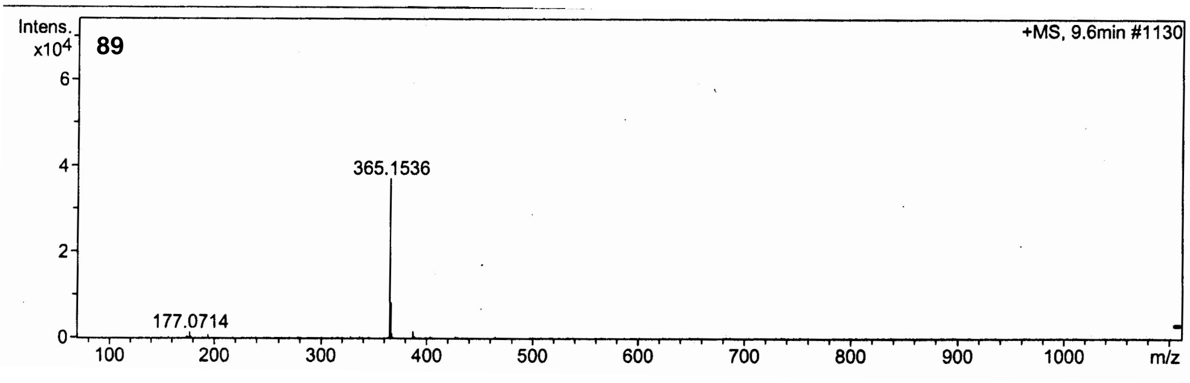
Intens.
x10^4
89
+MS, 9.6min #1130
365.1536
177.0714
0
2
4
6
100
200
300
400
500
600
700
800
900
1000
m/z

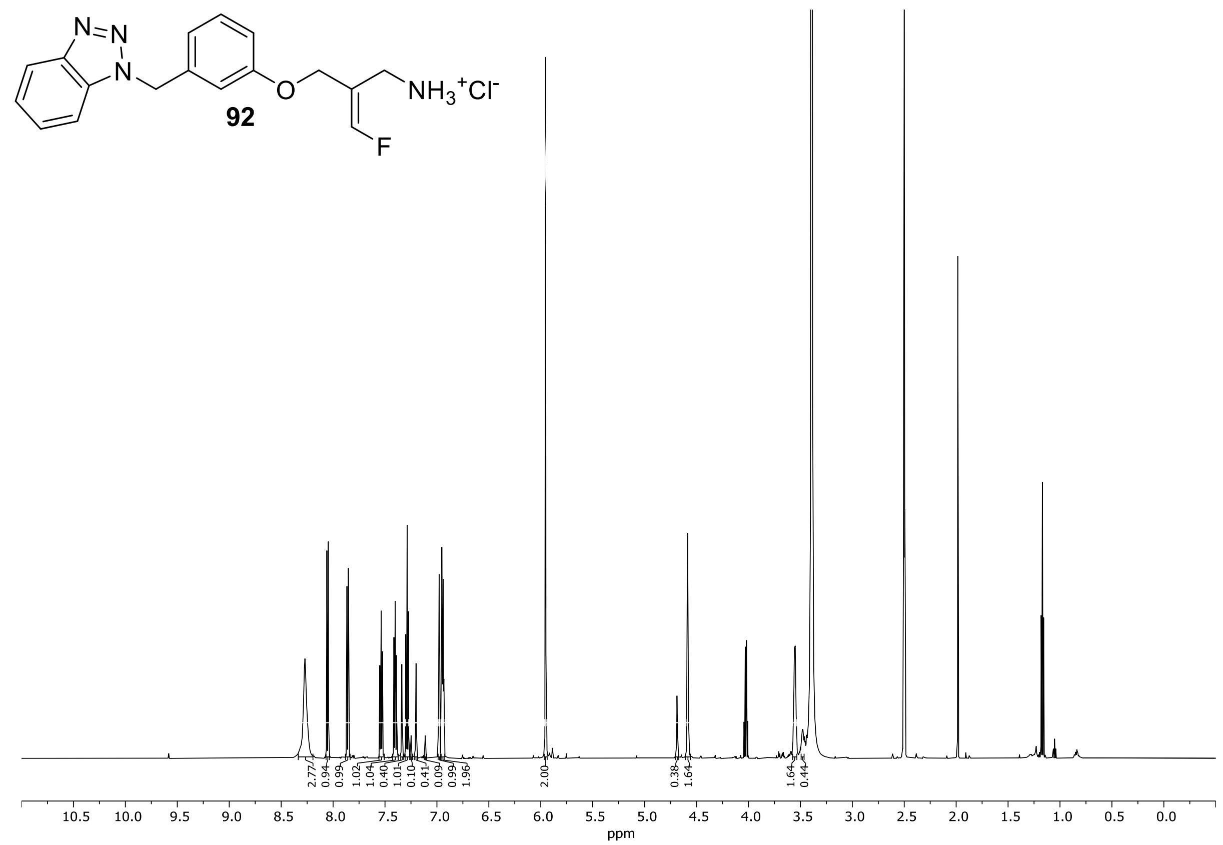

N=N
N
O
NH3+Cl-
F
92
2.77
0.94
0.99
1.02
1.04
0.40
1.01
0.10
0.41
0.09
0.99
1.96
2.00
0.38
1.64
1.64
0.44
ppm

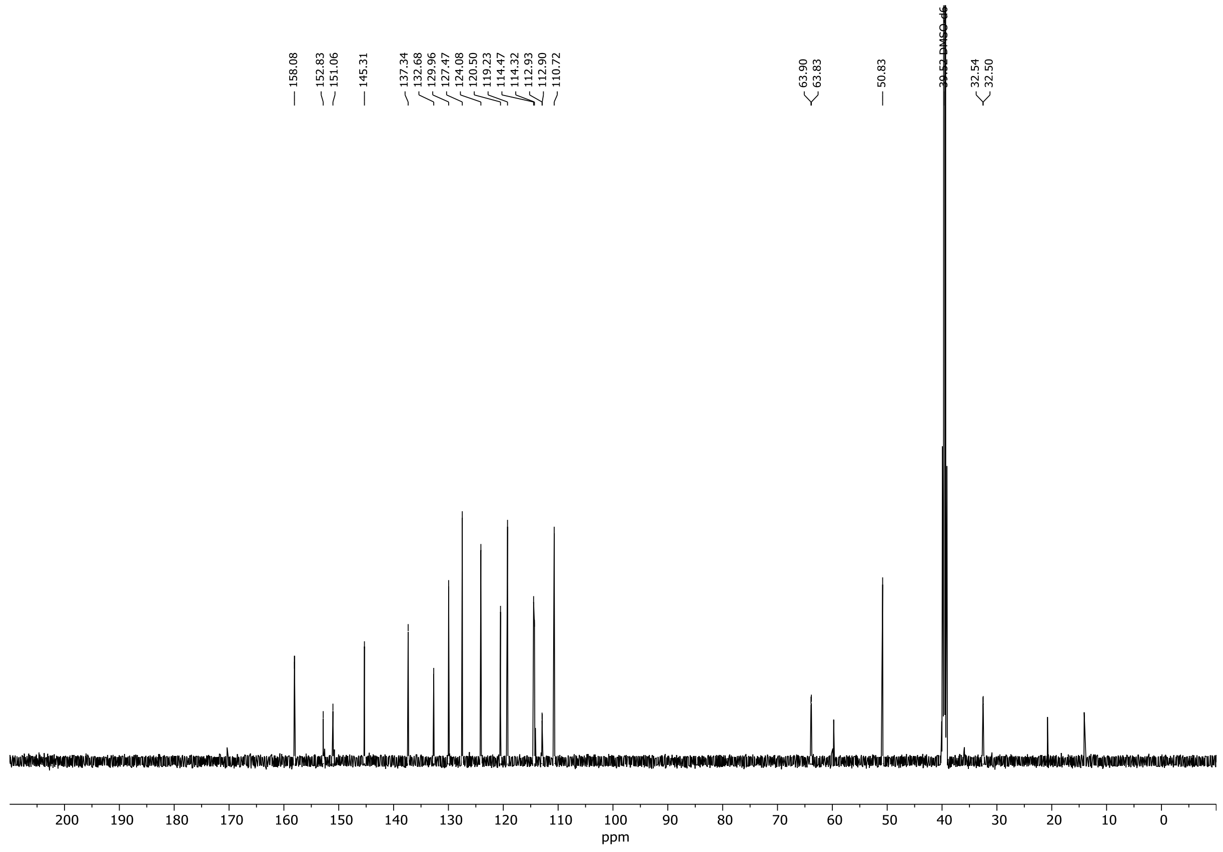

158.08
152.83
151.06
145.31
137.34
132.68
129.96
127.47
124.08
120.50
119.23
114.47
114.32
112.93
112.90
110.72
63.90
63.83
50.83
39.52 DMSO-d6
32.54
32.50
ppm

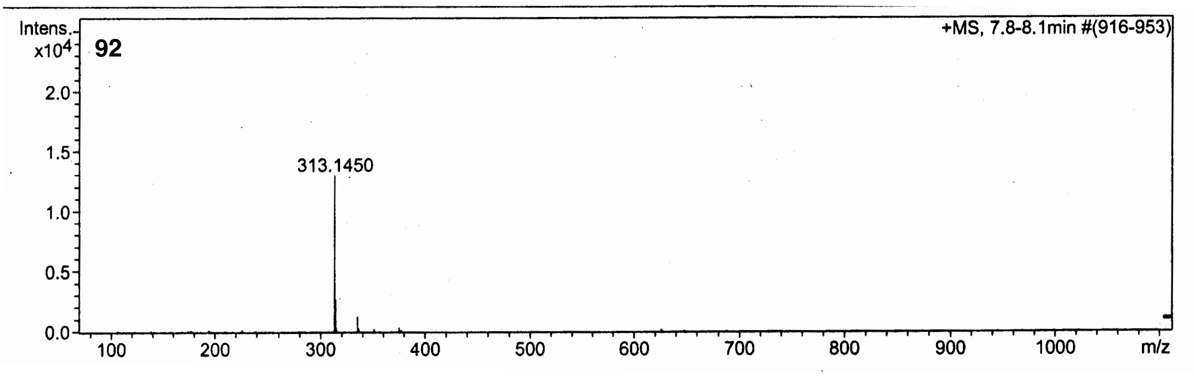
Intens.
x10^4
92
+MS, 7.8-8.1min #(916-953)
313.1450
2.0
1.5
1.0
0.5
0.0
100
200
300
400
500
600
700
800
900
1000
m/z

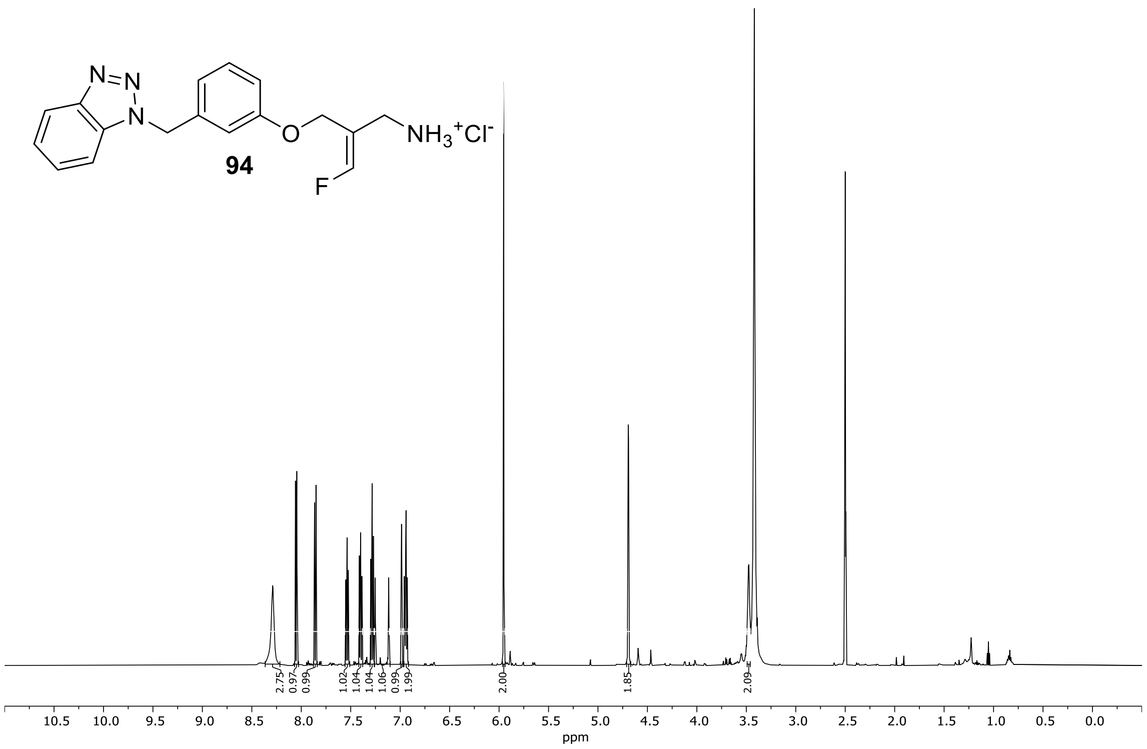

94
2.75
0.97
0.99
1.02
1.04
1.04
1.04
1.06
0.99
1.99
2.00
1.85
2.09
10.5 10.0 9.5 9.0 8.5 8.0 7.5 7.0 6.5 6.0 5.5 5.0 4.5 4.0 3.5 3.0 2.5 2.0 1.5 1.0 0.5 0.0
ppm

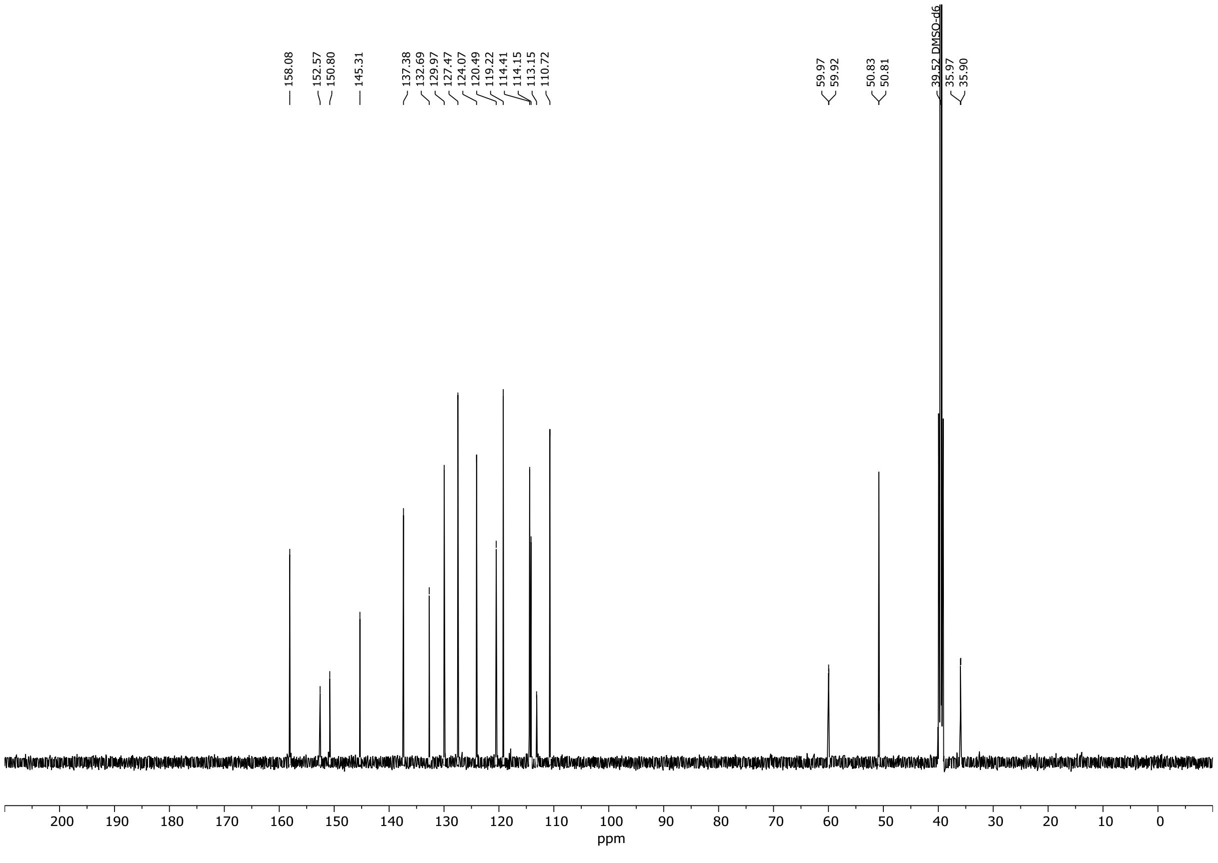
158.08
152.57
150.80
145.31
137.38
132.69
129.97
127.47
124.07
120.49
119.22
114.41
114.15
113.15
110.72
59.97
59.92
50.83
50.81
39.52 DMSO-d6
35.97
35.90
200 190 180 170 160 150 140 130 120 110 100 90 80 70 60 50 40 30 20 10 0
ppm

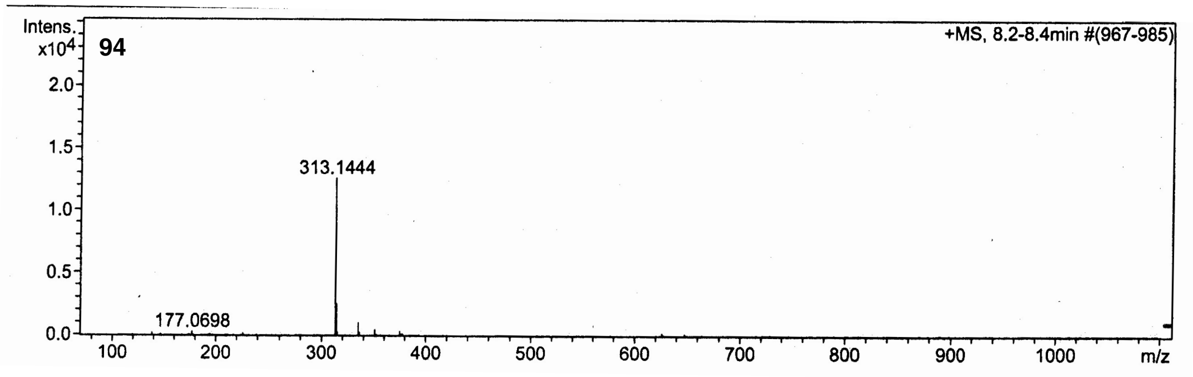
Intens.
x10^4
94
+MS, 8.2-8.4min #(967-985)
313.1444
177.0698
m/z

4. Means and standard deviations of the inhibition data listed in Table 4

Inhibition of the activity of VAP-1 in human plasma and sVAP-1 isolated from bovine plasma by selected compounds evaluated with a 15 min pre-incubation of enzyme and inhibitor

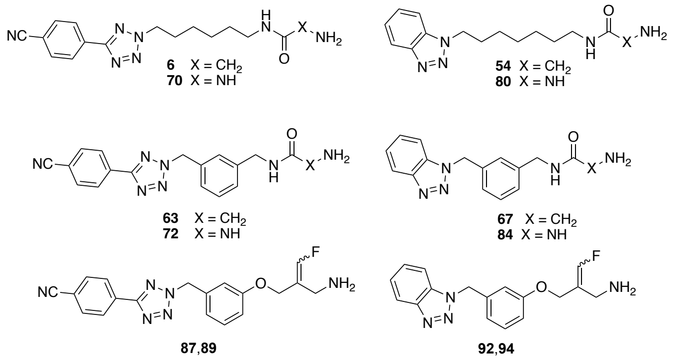

Cpd	Inhibition IC_{50} (µM)[a]	
	VAP-1 in human plasma	sVAP-1 from bovine plasma
**Glycine amides**		
**6**	39 ± 8	2.3 ± 0.35[b]
**54**	39 ± 13	1.7 ± 0.2
**63**	0.32 ± 0.04	0.13 ± 0.01
**67**	1.4 ± 0.3	0.36 ± 0.05
**Semicarbazides**		
**70**	1.3 ± 0.3	0.067 ± 0.001
**80**	0.40 ± 0.01	0.024 ± 0.002
**72**	0.17 ± 0.02	0.022 ± 0.004
**84**	0.62 ± 0.11	0.066 ± 0.008
**Fluoroallylamines**		
**87** (*E*:*Z* 95:5)	0.021 ± 0.008	0.045 ± 0.017
**89** (*E*:*Z* 0:100)	0.050 ± 0.011	0.040 ± 0.009
**92** (*E*:*Z* 80:20)	0.0074 ± 0.0011	0.030 ± 0.008
**94** (*E*:*Z* 8:92)	0.0040 ± 0.0003[c]	0.033 ± 0.008
**References**		
**1**	2.1 ± 0.2	0.37 ± 0.14[d]
**2** (PXS-4728)	0.0016 ± 0.0004	0.027 ± 0.003[e]
**4** (Mofegiline)	0.0119 ± 0.0001	not determined

[a] IC_{50} values are reported as means ± standard deviations (n = 2), unless otherwise indicated.
[b] n = 4;
[c] n = 3;
[d] n = 5;
[e] n = 4.
